# Flavin-Mediated Photocatalysis Provides a General Platform for Sulfide C–H Functionalization

**DOI:** 10.1021/acscatal.3c05785

**Published:** 2024-01-31

**Authors:** Alex S. Anderton, Oliver J. Knowles, James A. Rossi-Ashton, David J. Procter

**Affiliations:** Department of Chemistry, University of Manchester, Oxford Road, Manchester M13 9PL, U.K.

**Keywords:** flavin, sulfides, photocatalysis, radical, alpha functionalization

## Abstract

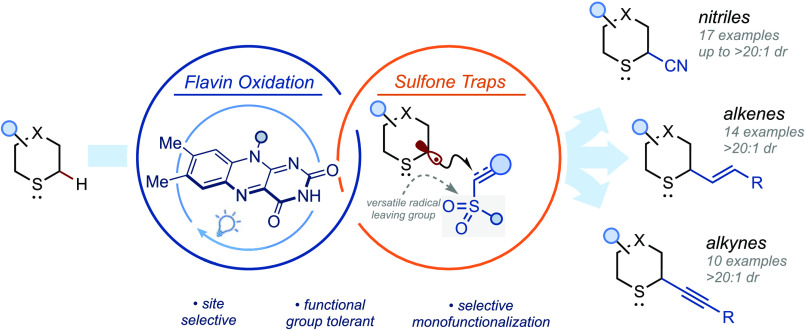

Functionalized sulfides are important in many areas of science, ranging from chemical biology through drug discovery to organic materials chemistry. Sulfides bearing pendant reactive groups in the α-position are particularly useful; however, methods for the selective valorization of simple sulfides or the late-stage functionalization of complex sulfides by the convenient addition of valuable functionality are underexplored. Here we exemplify a general reaction platform for sulfide functionalization by showcasing three modes of α-sulfur C–H functionalization; cyanation, alkenylation, and alkynylation. Using inexpensive and commercially available riboflavin tetraacetate and visible light, decoration of both feedstock and complex sulfides proceeds in a good yield and with high selectivity. Methionine-containing peptides can also be selectively functionalized and a tolerance screen using amino-acid dopants suggests that the platform is compatible with most amino-acid side chains and thus is a potential tool for bioconjugation.

## Introduction

**Figure 1:**
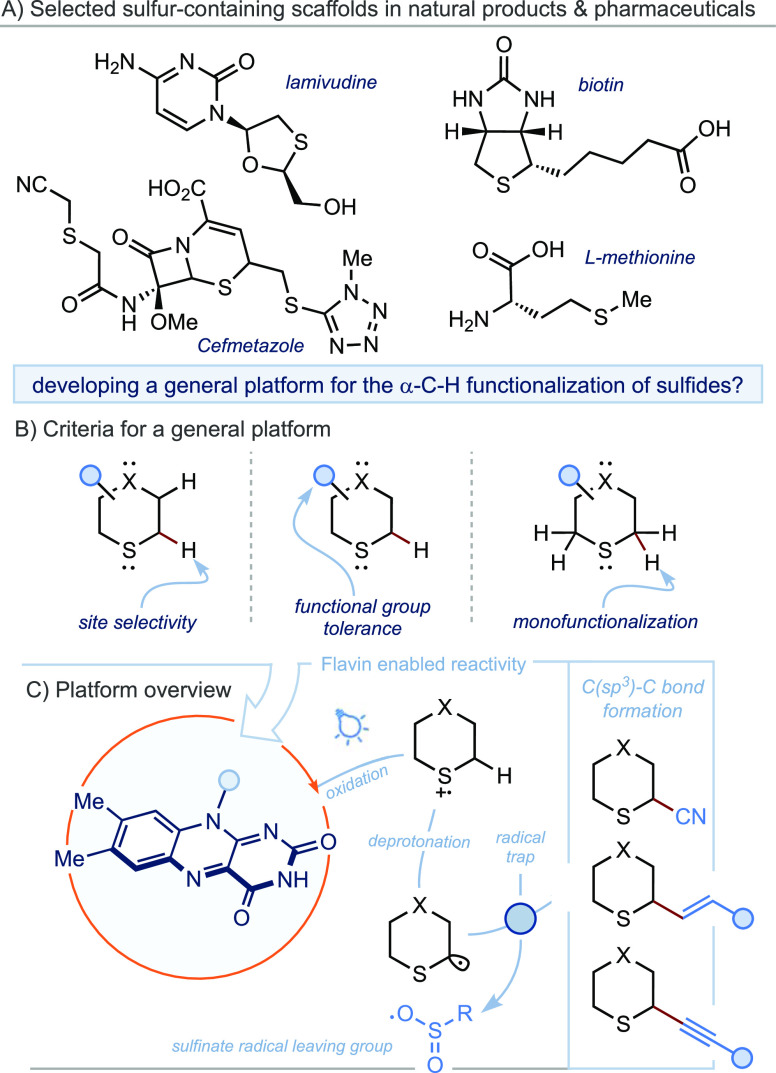
(A) Bioactive sulfide-containing molecules. (B) Essential criteria for a general approach to α-sulfur C–H functionalization; (C) flavin-mediated photocatalytic α-sulfur C–H functionalization; reaction platform overview.

Sulfur is a vital element for life and is a key component of bioactive molecules (Figure 1A).^1^ Most notably, its presence in the amino acids methionine and cysteine makes it important in many proteins, with sulfur–sulfur interactions determining a protein’s tertiary structure and thus its function.^2^ Sulfur is the fifth most abundant element in small-molecule drugs and is typically utilized in the S(II) (sulfide), S(IV) (sulfoxide), and S(VI) (sulfone, sulfoximine, etc.) oxidation states. These higher-oxidation-state sulfur motifs are typically afforded by late-stage oxidation of the corresponding sulfides,^3^ thus, convenient and general methods for the valorization of simple sulfides or late-stage modification of complex sulfides by selective functionalization enable rapid access to a range of sulfur-containing species and an important swathe of chemical space.

In terms of building and modifying sulfides, the very properties that shape sulfur’s function in biology—for example, its ability to bind metal ions—often impede the metal-catalyzed cross-coupling of organic fragments in its presence.^4^ Thus, a general, metal-free reaction platform that leverages sulfur’s innate reactivity and allows site-selective bond construction would provide convenient access to libraries of important sulfides for subsequent exploitation. We recently disclosed a riboflavin (RF)-catalyzed process for the synthesis of methionine analogues.^5^ In the study, RF was found to exhibit excellent site selectivity for the activation of C–H bonds α- to sulfur and the addition of α-sulfur radicals to electron-deficient alkenes including dehydroamino acids. If this selectivity could be harnessed in selective processes that afford a wide array of α-functionalized sulfides, then the approach could provide a general, metal-free, functionalization protocol for both simple and complex sulfides. In addition to exceptional site selectivity, we envisaged that a general reaction platform must exhibit wide functional group tolerance; although radical couplings α- to sulfur have been reported,^6^ they are typically isolated examples using unfunctionalized sulfide substrates.^7a^

Radical formation α- to sulfur can be achieved by direct HAT^6^ or by a single-electron transfer (SET) oxidation of sulfur/deprotonation sequence.^5,8^ For example, MacMillan has utilized the second mechanistic approach in a site-selective functionalization of methionine residues using lumiflavin as a photocatalyst.^8^ By employing a SET oxidation of sulfur/deprotonation approach, we postulated that the risk of overfunctionalization would be removed; in the functionalized products, the reactivity at sulfur is electronically/sterically downmodulated.

Here, we exemplify a general reaction platform for sulfide modification by showcasing three modes of selective sulfide α-C–H monofunctionalization: cyanation, alkenylation, and alkynylation. Using inexpensive and commercially available riboflavin tetraacetate (RFTA) and visible light, decoration of both feedstock and complex sulfides generates high-value sulfide products in good yield and with high selectivity (Figure 1C). Methionine-containing peptides can also be selectively functionalized using the platform and a tolerance screen using amino-acid dopants suggests that the platform is compatible with most amino-acid side chains and thus is a potential tool for bioconjugation.

## Results and Discussion

### Reaction Development

We initiated our investigation by exploring the flavin-mediated α-cyanation of sulfides, initially employing conditions reported for previous flavin-mediated processes (Table 1).^5^ However, reacting tetrahydrothiopyran **1a** with tosyl cyanide^7b^ under such conditions—10 mol % RF in a mixture of 1:19 DMF/H_2_O at 5 mM concentration—afforded only trace amounts of α-cyanated sulfide **2a** (entry 1). These conditions were believed to be unfit for cyanation due to the low reaction concentration and the insolubility of the reagents in the aqueous medium. Employing RF in MeCN at 100 mM gave sulfide **2a** in an increased 7% yield (entry 2). We then turned to RF derivatives with greater solubility in organic solvents and identified commercial and inexpensive RFTA as a promising flavin photocatalyst, affording sulfide **2a** in 21% yield (entry 3). Further optimization identified the use of 10 equiv of tosyl cyanide as optimal (entry 5). Pleasingly, the use of degassed acetone gave **2a** in an optimized 66% yield after an 18 h reaction time (entry 7) with no overreacted products or sulfoxide detected.^9^ Control reactions revealed that both light and flavin were crucial for reactivity (entries 8 and 9).

**Table 1:**
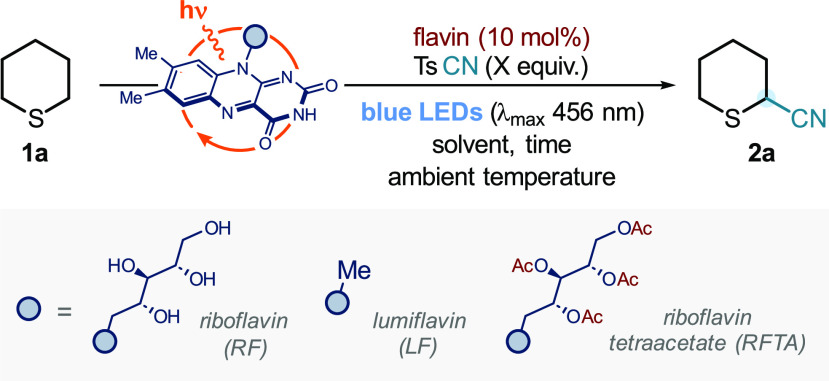
Optimization of α-C–H Cyanation of Sulfides

entry	flavin	solvent	conc. (mM)	time (h)	TsCN (equiv)	yield^a^ (%)
1	RF (10%)	DMF/H_2_O (1:19)	5	48	1.5	<5
2	RF (10%)	MeCN	100	18	1.5	7
3	RFTA (10%)	MeCN	100	18	1.5	21
4	LF (10%)	MeCN	100	18	1.5	14
5	RFTA (10%)	MeCN	50	18	10	40
6	RFTA (10%)	dioxane	50	18	10	54
7	RFTA (10%)	acetone	50	18	10	66
8^b^	RFTA (10%)	acetone	50	18	10	0
9		acetone	50	18	10	0

a Yield determined by ^1^H NMR spectroscopy using MeNO_2_ as the internal standard.

b Reaction performed in the dark. RF = riboflavin. RFTA = riboflavin tetraacetate. LF = lumiflavin.

### Substrate Scope

**Figure 2:**
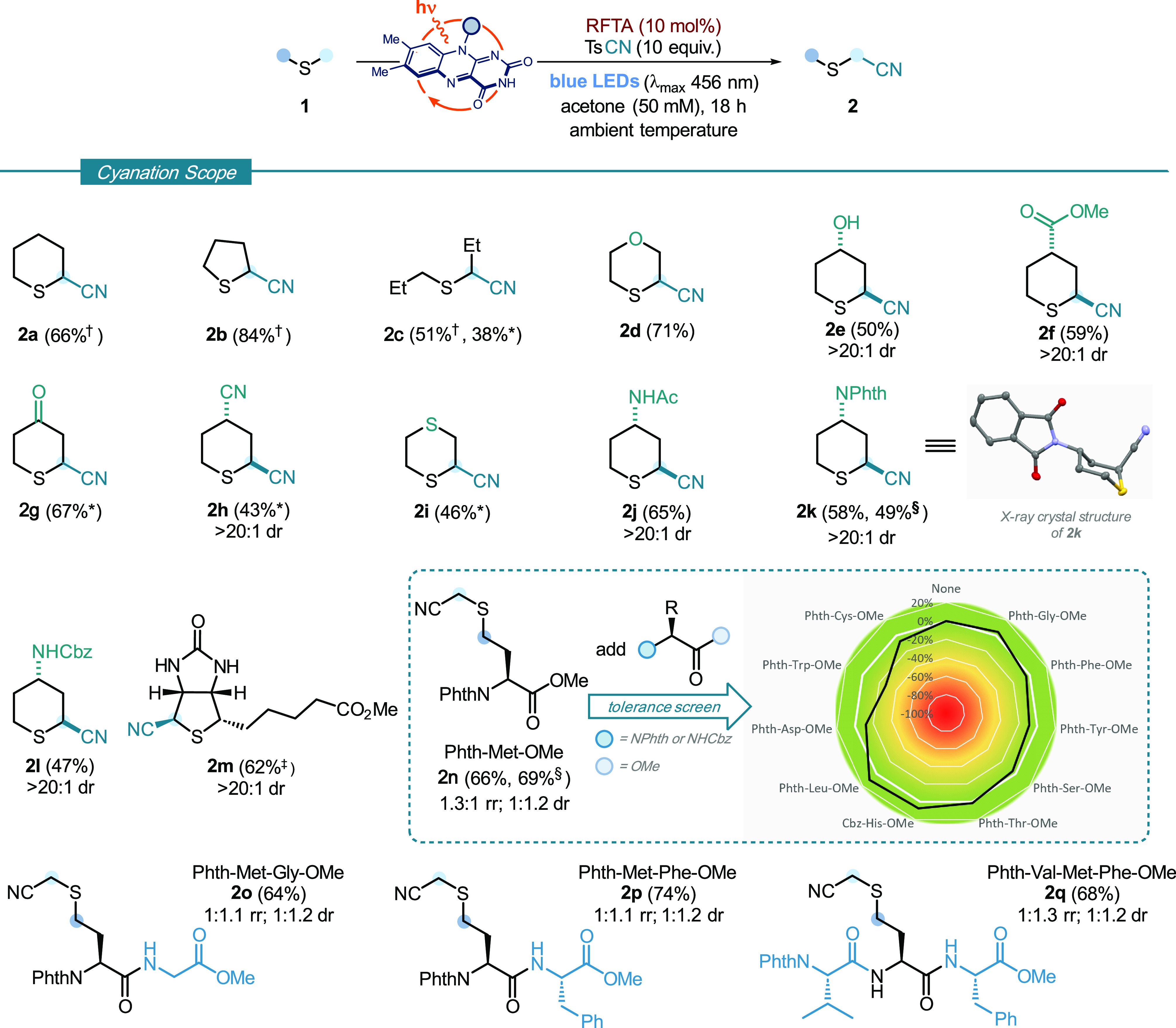
Scope of RFTA-photocatalyzed α-C–H functionalization of sulfides using tosyl cyanide. Reaction conditions: **1** (0.1 mmol, 1 equiv), tosyl cyanide (1.0 mmol, 10 equiv), and RFTA (0.01 mmol, 10 mol %), in acetone (50 mM with respect to **1**), at room temperature (∼25 °C, controlled by use of a fan) for 18 h, under irradiation by blue LEDs (λ_max_ 456 nm, maximum irradiance). ^†^ Yield determined by ^1^H NMR spectroscopy using MeNO_2_ as the internal standard. * Reaction run at 0.2 mmol scale with respect to sulfide **1**. ^§^ Reaction was run at 1.0 mmol scale with respect to sulfide **1**. ^‡^ 24 h reaction time. Ac = Acyl. Phth = Phthalimido. Cbz = Benzyloxy carbonyl. The tolerance screen of amino acids completed by doping the reaction conditions with 0.1 mmol (1 equiv) of *N*-Phth/Cbz protected amino acid methyl ester; percentage change in yields shown in the radar plot. (See Supporting Information for more details.).

We next explored the generality of the cyanation procedure (Figure 2). Simple aliphatic sulfides such as tetrahydrothiophene (**1b**) and di-*n*-propyl sulfide (**1c**) afforded the cyanated sulfides **2b** and **2c** in 84 and 51% yield, respectively. 1,4-Oxathiane (**1d**) and tetrahydrothiopyran-4-ol (**1e**) were cyanated to afford sulfides **2d** and **2e** in 71 and 50% with complete chemoselectivity for functionalization α- to sulfur. Modified sulfide **2e** was obtained in >20:1 dr. Sulfides containing ester, ketone, and cyano functional groups (**1f**–**1h**) underwent efficient C–H cyanation with yields between 43 and 67% and >20:1 dr (for **2f**, **2h**). In all cases, the process showed complete selectivity for monofunctionalization; even cyanation of **1i** which possesses four potential sites—and eight C–H bonds—for activation underwent selective monofunctionalization with no multicyanated products detected in the crude ^1^H NMR spectrum. Amines bearing common protecting groups underwent smooth cyanation; *N*-acetyl (**2j**), *N*-phthalimido (**2k**), and *N*-benzyloxy carbonyl (**2l**) protected products were isolated in 47–65% yield and with >20:1 dr. X-ray crystallography was used to confirm the *trans* relative stereochemistry of **2k**. The reaction was scaled up 10-fold, affording only a small decrease in the yield of **2k** (49% isolated yield, 133 mg). We propose that the high selectivity for formation of the *trans*-diastereoisomers arises from trapping of an axial α-sulfur radical on the face opposite to the C4 substituent, with the thiopyran ring adopting either a boat or chair conformation.

We next sought to apply our flavin-mediated photocatalytic α-sulfur C–H cyanation in the late-stage functionalization of a complex bioactive molecule. D-Biotin is a sulfide-containing biomolecule widely used in chemical biology (Figure 1A).^10^ Pleasingly, the potentially sensitive cyclic urea motif in biotin was well-tolerated and α-cyanation of D-biotin methyl ester gave an unprecedented biotin analogue (**2m**) in 62% yield as a single regioisomer and with >20:1 dr. NMR studies confirmed the stereochemistry of the product (see Supporting Information for more details); cyanation occurs on the less sterically hindered, convex face of the fused bicyclic core. Complete regioselectivity was also observed, with only C8 cyanation occurring and with the integrity of the C2 stereocenter intact. Biotin derivatives with C8 functionality have been used as linkers for conjugating antibodies.^11^ Typically, C8 activation of biotin has been achieved by traditional, forcing Pummerer rearrangement conditions;^12^ our new methodology allows mild and selective access to novel biotins with a potential site for linkage to an antibody- or protein-binding warhead.

Given the importance of functionalized peptides and noncanonical amino acids in medicine and biotechnology,^13^ we examined the functionalization of methionine and methionine-containing peptides. *N*-Phthalimidomethionine methyl ester **1n** underwent cyanation to give **2n** in 66% yield (1.3:1 rr, 1.2:1 dr for the minor regioisomer); a convenient 10-fold scale-up gave a slightly improved isolated yield of **2n** (69%, 228 mg). Dipeptide conjugates of methionine with glycine and phenylalanine also underwent smooth cyanation to give **2o** and **2p**, respectively, in 64 and 74% yield. Interestingly, flavins are known to photocatalyze the oxidation of benzylic positions in the presence of oxygen;^14^ however, utilizing degassed acetone allows for selective sulfide oxidation and no benzylic oxidation of the phenylalanine residue was observed. A tripeptide with an internal methionine residue was also a good substrate; **2q** was obtained in 68% yield.

To conveniently assess the likely compatibility of the flavin-mediated photocatalytic cyanation with the presence of other amino-acid residues containing reactive and potentially oxidizable side-chain functionality, a tolerance screen was carried out; the cyanation of **2n** was carried out in the presence of 1 equiv of an amino-acid dopant (Phth/Cbz-amino acid-OMe). Nonpolar side chains such as those in glycine and leucine had little impact on the yield of cyanated methionine **2n**. Similarly, the presence of phenylalanine and histidine in the reaction mixture had no effect on the cyanation and no products of dopant oxidation were detected. Pleasingly, the presence of tyrosine also had little impact on the flavin-mediated photocatalytic cyanation of methionine; although tyrosine is known to undergo phenolic oxidation by flavins,^15^ our process is selective for sulfur oxidation. The presence of alcohol-containing serine and threonine was also well-tolerated. Strikingly, the inclusion of cystine proved compatible, suggesting that oxidation of the disulfide motif by the photoexcited flavin does not occur or is not productive.

A lower yield of cyanated methionine **2n**—up to 27% below the benchmark yield—was observed when aspartic acid and tryptophan derivatives were added to the reaction mixture. The flavin photocatalyst can likely oxidize the side chains, with carboxylate^16a^ or indole oxidation taking place,^16b^ respectively. Overall, however, the flavin-mediated photocatalytic C–H cyanation of methionine showed a good overall tolerance to the presence of a wide range of amino-acid side chains.

**Figure 3:**
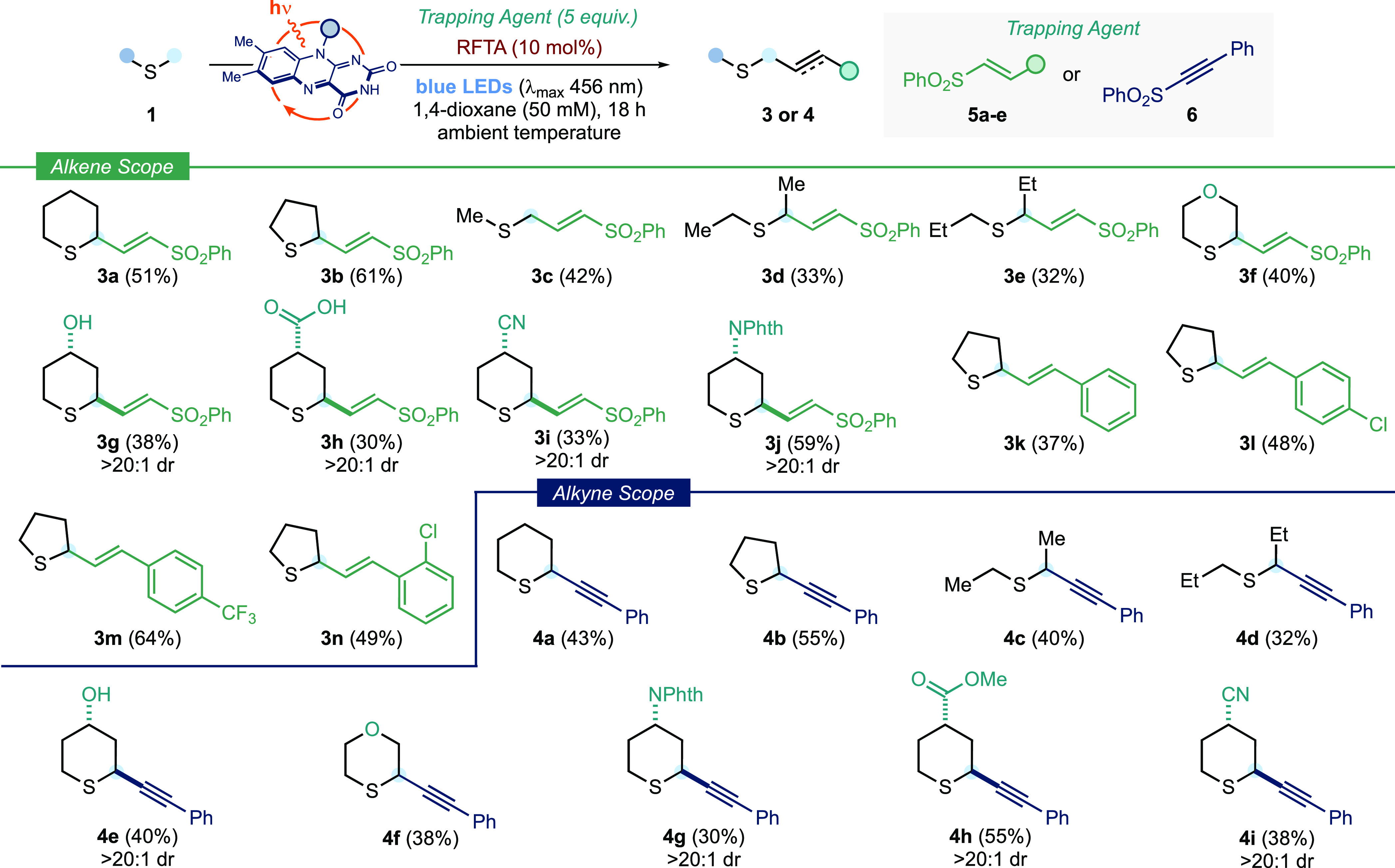
Scope of RFTA-photocatalyzed α-C–H functionalization of sulfides with sulfonyl alkenes and alkynes. Reaction conditions: **1** (0.1 mmol, 1 equiv), trapping agent (0.5 mmol, 5 equiv), and RFTA (0.01 mmol, 10 mol %), in 1,4-dioxane (50 mM with respect to **1**), at room temperature (∼25 °C, controlled by use of a fan) for 18 h, under irradiation by blue LEDs (λ_max_ 456 nm, maximum irradiance) for 18 h. ^†^ Yield determined by ^1^H NMR spectroscopy using MeNO_2_ as an internal standard. NPhth: *N*-Phthalimido.

We next extended the reaction platform for α-sulfur C–H functionalization by engaging alternative radical coupling partners. We chose to exploit alkenyl and alkynyl sulfones as coupling partners in flavin-mediated α-sulfur C–H alkenylation and alkynylation processes (Figure 3). We proposed that the use of 1,2-bis(phenylsulfonyl)ethylene as the coupling partner would allow alkenyl sulfone motifs to be introduced into sulfides; alkenyl sulfones are common motifs in covalent enzyme inhibitors;^17^ however, methods to install alkenyl sulfone units α- to sulfur are limited, requiring the use of toxic organotin reagents and harsh conditions^18^ or prefunctionalized starting materials.^19^

Adapting the reaction platform to accommodate the use of 1,2-bis(phenylsulfonyl)ethylene^20^ required only fine-tuning of the conditions; switching from acetone to dioxane as solvent and lowering the amount of radical trap [5 equiv of 1,2-bis(phenylsulfonyl)ethylene **5a**] gave alkenylated product **3a** in 51% isolated yield upon coupling to tetrahydrothiopyran **1a**. Exploring the scope, we found that simple cyclic and acyclic aliphatic sulfides were well-tolerated, with yields between 32 and 61% (**3b**–**3e**). In accordance with the cyanation protocol, alcohol- and ether-containing sulfides showed complete selectivity for functionalization α- to sulfur (**3f** and **3g**). The C–H alkenylation also tolerated the presence of carboxylic acid (**3h**), cyano (**3i**), and the *N*-phthalimido functionality (**3j**). The process was readily extended to a suite of styrenyl sulfones, prepared from the parent styrenes using a simple one-step procedure. Substitution on the styrene was tolerated in highly *trans*-selective (>20:1 dr) C–H cross-couplings with tetrahydrothiophene, including chloro (**3l** and **3n**) and trifluoromethyl (**3m**). Alkynyl sulfones were also compatible with the reaction platform. For example, phenylethynyl phenyl sulfone **6** underwent coupling under the metal-free photocatalytic conditions with simple cyclic and acyclic sulfides (**4a**–**4d**), and more complex sulfides containing hydroxyl (**4e**), ether (**4f**), protected amino (**4g**), ester (**4h**), and cyano (**4i**) functional groups, to give products of α-sulfur C–H alkynylation in moderate yield and with >20:1 diastereoselectivity (**4e**, **4g**–**i**). As for the cyanation protocol, only products of monoalkenylation/alkynylation were observed.

Crucially, all three product classes delivered by our general flavin-mediated photocatalytic reaction platform contain attractive handles for exploitation. For example, products of alkynylation are potential substrates for bioconjugation using click chemistry.^21^ Similarly, alkenylsulfonyl-containing sulfides are promising Michael acceptors that are also of potential use in bioconjugation.^22^ Furthermore, sulfide products bearing alkenylsulfonyl^23^ and cyano groups^24^ hold promise for the design of covalent inhibitors, for example, of cysteine and serine proteases.^25^

### Mechanistic Study

**Figure 4:**
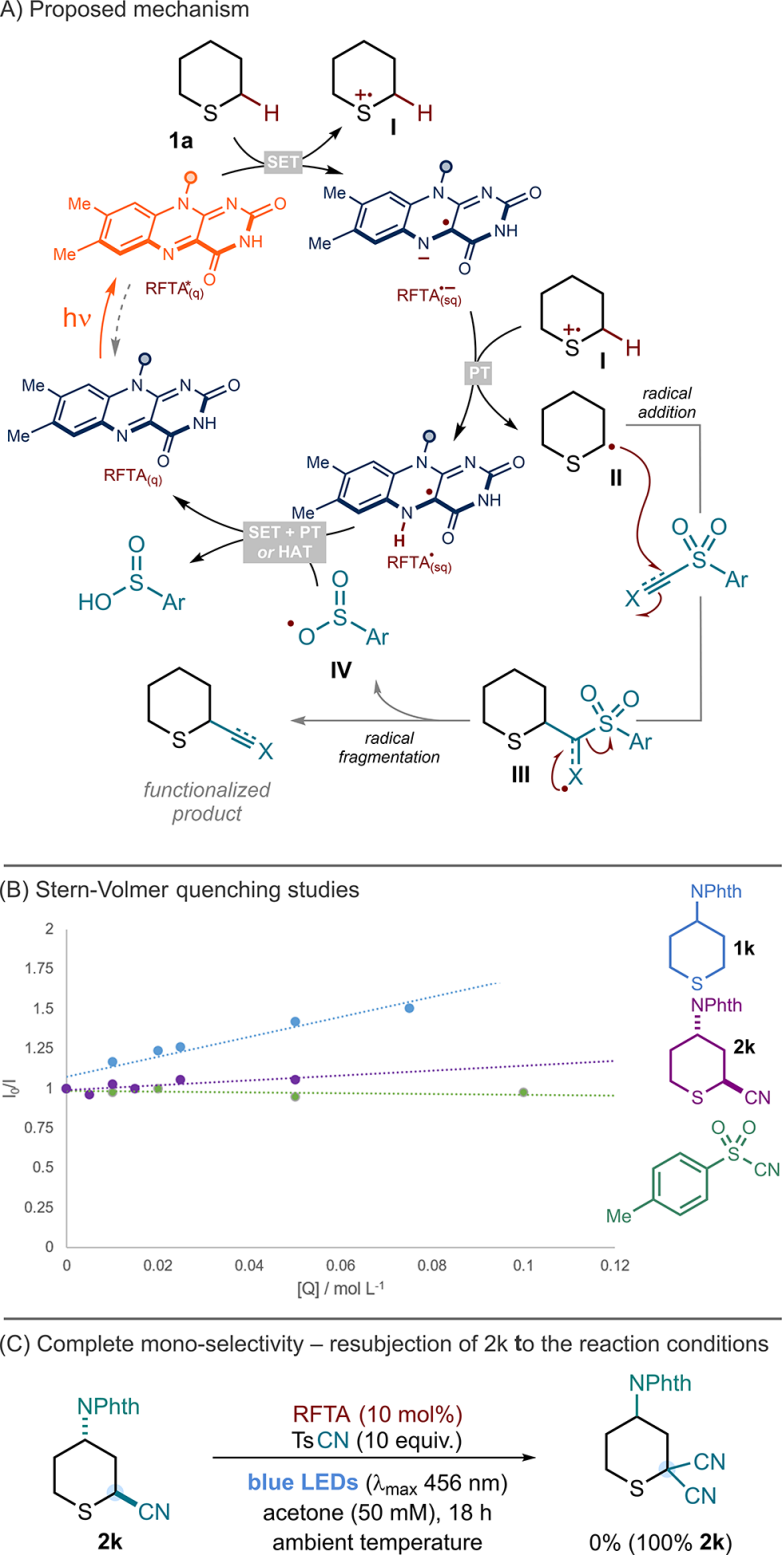
(A) Proposed mechanism for the general RFTA-photocatalyzed α-C–H functionalization of sulfides. X = N, CHSO_2_Ph, CHAr, or CAr. (B) Stern–Volmer quenching plots for **1k**, **2k**, and TsCN. (C) Reaction resubjecting the cyanated sulfide **2k** to the cyanation conditions.

In line with previous work,^5,7^ we propose that irradiation with visible light converts RFTA in the quinone ground state (RFTA_(q)_) to the corresponding singlet excited state. This excited state flavin carries out SET oxidation of sulfur, resulting in the generation of sulfur radical cations **I** and the flavin semiquinone radical anion (RFTA^•–^_(sq)_). After intersystem crossing to a triplet state, RFTA^•–^_(sq)_ then deprotonates **I** to generate nucleophilic α-sulfur radicals **II** which undergo radical addition into the π* orbital of the coupling partner to give radical intermediates **III**. Radical fragmentation then delivers functionalized products and sulfinate radicals **IV**. The cycle is closed by SET reduction and protonation of the sulfinate radical by RFTA_(sq)_ or by direct hydrogen-atom transfer from RFTA^•^_(sq)_ to the sulfinate radical (Figure 4A).

Intrigued by the high selectivity of the flavin-mediated α-sulfur C–H coupling platform for the monofunctionalized product, when there was clear potential for overfunctionalization, Stern–Volmer quenching studies were conducted on starting material **1k** and product **2k** (Figure 4B). While **1k** quenched the excited-state RFTA with a Stern–Volmer quenching constant (*K*_SV_) of 6.3, the cyanated product **2k** had a much lower *K*_SV_ value of 1.5. This implies that, while the product can undergo further oxidation by RFTA, the process is slow and does not produce bis-cyanated products. Stern–Volmer quenching studies confirmed that tosyl cyanide and dioxane do not quench the excited state of the flavin at any appreciable rate compared to the sulfide substrate. To underline the specificity of the platform for monofunctionalization, the sulfide product of cyanation **2k** was resubjected to the reaction conditions; no further cyanation was observed and 100% of **2k** was recovered after 18 h (Figure 4C).

## Conclusions

In summary, we have developed a general platform for the α-sulfur C–H functionalization of sulfides. The metal-free process is mediated by a readily available and inexpensive flavin photocatalyst and visible light and has been exemplified through the use of three different coupling partners in delivering products of cyanation, alkenylation, and alkynylation. The process shows good functional-group tolerance—for example, sulfur is selectively oxidized in the presence of ether and alcohol groups, is well-suited for complex sulfides such as biotin, and is selective for monofunctionalization. Small methionine-containing peptides were also functionalized in good yield, and a tolerance screen using amino-acid dopants in the cyanation suggests that the platform is compatible with many potentially reactive amino-acid side chains.

## Supporting Information Available

Additional experiments, experimental procedures, details of the reaction setup, characterization data, X-ray data, Stern–Volmer quenching studies, and NMR spectra for all new compounds (PDF)
