# Supplementary material for: Flavin-Mediated Photocatalysis Provides a General Platform for Sulfide C–H Functionalization
Source: ACS Catal. 2024 Jan 31;14(4):2395–401. doi: 10.1021/acscatal.3c05785 (PMC10877610; doi:10.1021/acscatal.3c05785)
Supplement: Supplementary file 1 — cs3c05785_si_001.pdf [file cs3c05785_si_001.pdf]

Supporting Information

Flavin-Mediated Photocatalysis Provides a General Platform for Sulfide C−H Functionalization

Alex S. Anderton†, Oliver J. Knowles†, James A. Rossi-Ashton, David J. Procter*

Department of Chemistry, University of Manchester, Oxford Road, Manchester, M13 9PL, UK.

E-mail: david.j.procter@manchester.ac.uk

† These authors contributed equally.

Table of Contents:

General Experimental 2
Pictures of Reaction Set-Up 3
Cyanation Protocol Optimization 4
  Catalyst Screen 4
  Stoichiometry and Concentration Screen 5
  Solvent Screen 6
  Alternative Cyanation Reagents 7
  Control Reactions 8
Alkenylation Protocol Optimization 9
Alkynylation Protocol Optimization 10
  Solvent Screen 10
  Alkynylating Agent Screen 11
Riboflavin Tetraacetate 12
Radical Traps 13
Starting Material Sulfides 19
Cyanation Scope 31
  Amino Acid Tolerance Experiments 48
  Possible Origin of Diastereoselectivity 55
  X-Ray Crystallography Data 56
Alkenylation Scope 58

Possible Origin of Trans-Selectivity in Alkenylated Products 68
Alkynylation Scope 69
Observed Byproducts 75
Scale-Up Procedure 76
Unsuccessful Examples 78
Mechanistic Studies 79
Stern-Volmer Quenching Studies 79
Resubjection of Product to Reaction Conditions 84
^{1}H and ^{13}C NMR Spectra 85
References 128

General Experimental

All experiments were performed under an atmosphere of nitrogen, using anhydrous solvents, unless stated otherwise. All solvents and reagents were purchased from commercial sources and used as supplied. Photochemical reactions were subjected to irradiation from a 34W Kessil blue LED lamp (λ_{max} = 456 nm), with the reaction tube placed approximately 4 cm from the lamp. ^{1}H NMR spectra were recorded on NMR spectrometers at 400 MHz and 500 MHz and ^{13}C NMR at 100 MHz and 125 MHz. ^{1}H NMR chemical shifts (δH) and 13C NMR chemical shifts (δC) are quoted in parts per million (ppm) downfield from trimethylsilane (TMS) and coupling constants (J) are quoted in Hertz (Hz). Abbreviations for NMR data are s (singlet), d (doublet), t (triplet), q (quartet), m (multiplet). Infrared (IR) spectra were recorded as evaporated films or neat on a FTIR spectrometer and mass spectra were obtained using positive or negative electrospray ionisation (ESI), atmospheric pressure chemical ionization (APCI), or atmospheric solid analysis probe (ASAP) techniques. Column chromatography was carried out using silica gel 60 Angstrom (Ǻ), 240-400 mesh. Thin layer chromatography (TLC) was performed on aluminium sheets pre-coated with silica gel, 0.20 mm (Macherey-Nagel, Polygram® Sil G/UV254). TLC plates were visualized by UV absorption, phosphomolybdic acid, vanillin or potassium permanganate solution and heating. Preparative TLC was performed on 20 × 20 cm glass plates 500 µm or 2000 µm thickness.

Pictures of Reaction Set-Up

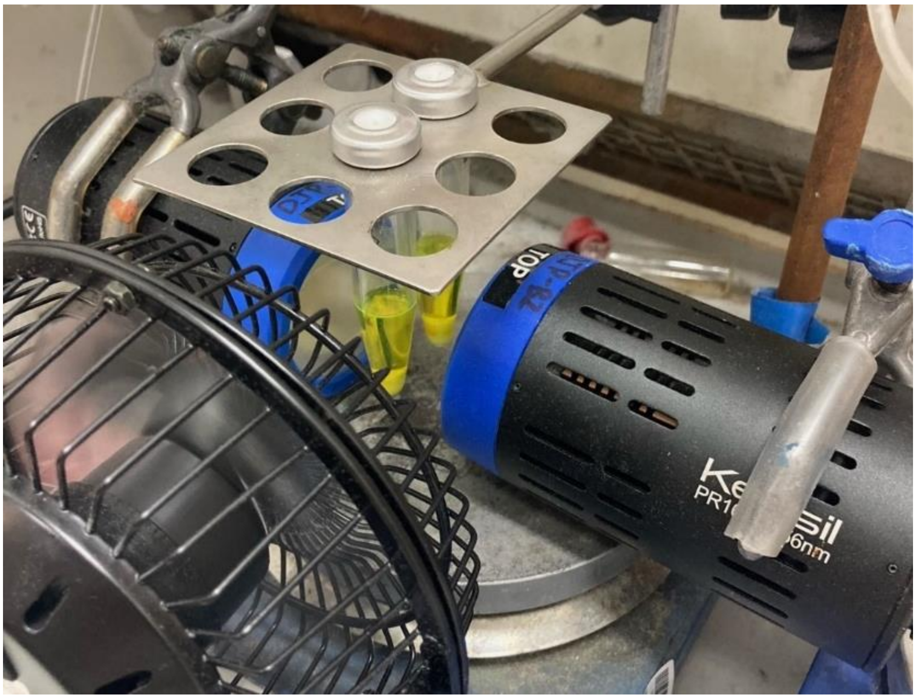

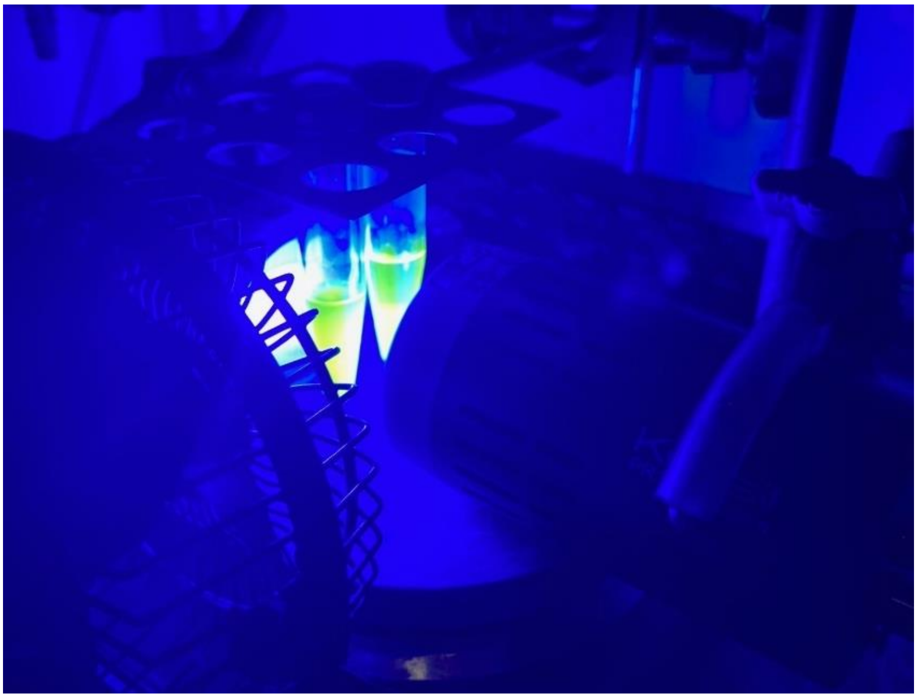

Cyanation Protocol Optimization

Catalyst Screen

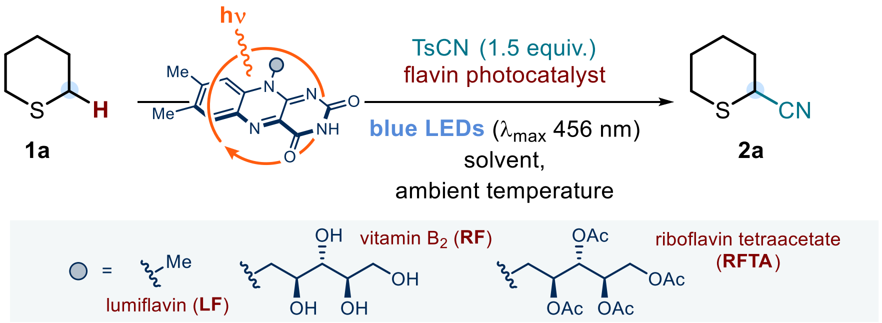

Entry	Flavin	Solvent	Conc. (mM)	Time (h)	Yield [a] (%)
1	RF (10 mol%)	5% DMF:H_2O	5	48	<5
2	RFTA (10 mol%)	5% DMF:H_2O	5	48	<5
3	RFTA (5 mol%)	MeCN	100	18	7
4	RF (10 mol%)	MeCN	100	18	7
5	RTFA (10 mol%)	MeCN	100	18	21
6	LF (10 mol%)	MeCN	100	18	14

[a] Yield determined by ^{1}H NMR using $MeNO_2$ as an internal standard.

Stoichiometry and Concentration Screen

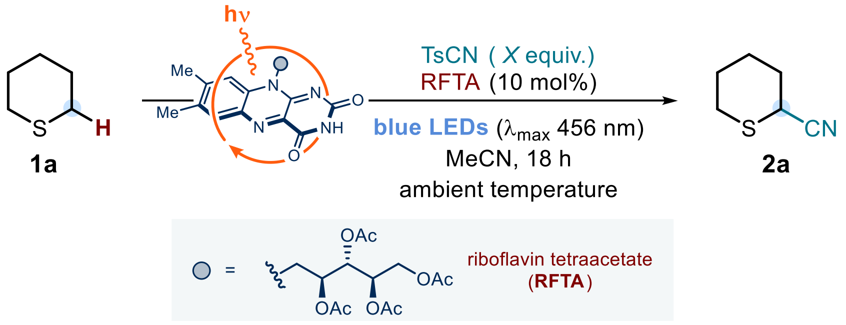

Entry	Conc. (mM)	**1a** (equiv.)	TsCN (equiv.)	Yield [a] (%)
1	10	1.0	1.5	21
2	50	1.0	1.5	24
3	25	1.0	1.5	13
4	15	1.0	1.5	10
5	50	1.0	2.0	18
6	50	1.0	3.0	23
7	50	1.0	5.0	33
8	50	1.0	10	40
9	50	1.0	1.0	6
10	50	3.0	1.0	11
11	50	5.0	1.0	12

[a] Yield determined by ^{1}H NMR using $MeNO_2$ as an internal standard.

Solvent Screen

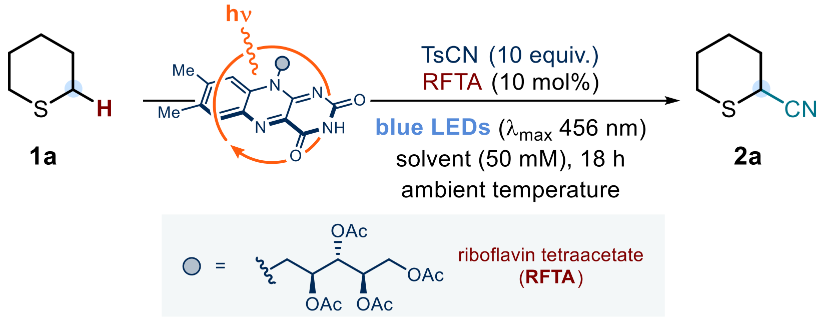

Entry	Solvent	Yield [a] (%)
1	MeCN	40
2	CH_2Cl_2	48
3	DMF	40
4	THF	30
5	1,4-Dioxane	54
6	DMA	38
7	1,2-DCE	55
8	EtOAc	35
9	DME	64
10	MeOH	34
11	EtOH	45
12	Acetone	66
13	DMSO	28
14	3:1 MeCN:H_2O	13
15	5% Acetone: H_2O	34

[a] Yield determined by ^{1}H NMR using $MeNO_2$ as an internal standard.

Alternative Cyanation Reagents

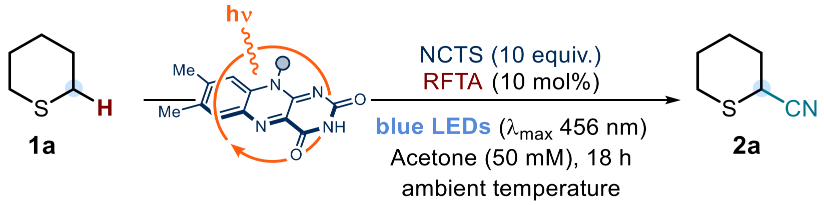

Alternate electrophilic CN sources: *N-cyano-N-phenyl-p-toluenesulfonamide (NCTS)*

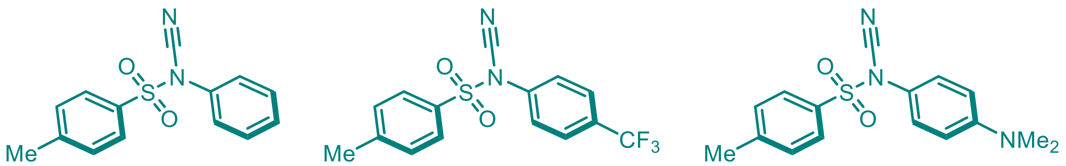

NCTS A *NCTS B* *NCTS C*

Entry	CN Source	Yield [a] (%)
1	NCTS A	<5
2	NCTS B	<5
3	NCTS C	<5

[a] Yield determined by ^{1}H NMR using $MeNO_2$ as an internal standard.

Control Reactions

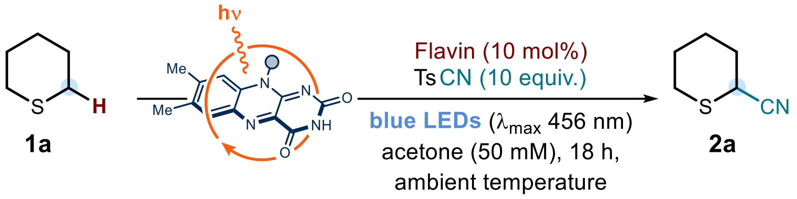

Entry	Flavin	Light	Yield [a] (%)
1	RFTA (10 mol%)	No light	0
2	No flavin	456 nm LEDs	0

[a] Yield determined by ^{1}H NMR using $MeNO_2$ as an internal standard.

Alkenylation Protocol Optimization

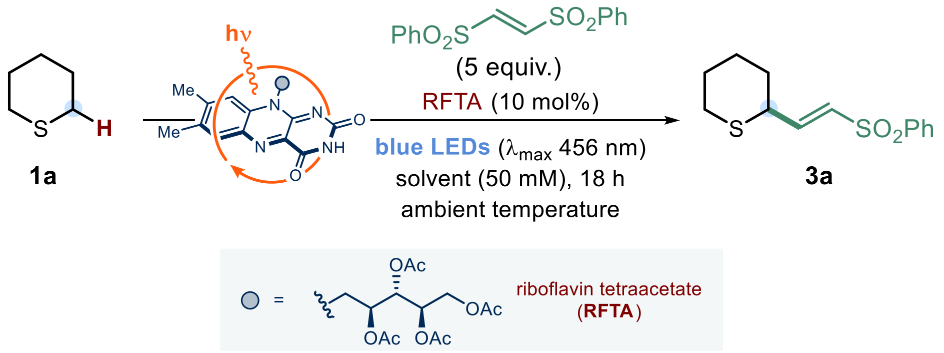

Entry	Solvent	Yield [a] (%)
1	Acetone	26
2	MeTHF	30
3	THP	5
4	DME	20
5	1,4-Dioxane	51
6	CH_2Cl_2	0
7	1,2-DCE	6
8	DMC	25
9	DEC	21
10	MeCN	0
11	DIPE	< 5
12	Et_2O	0

[a] Yield determined by ^{1}H NMR using $MeNO_2$ as an internal standard.

Alkynylation Protocol Optimization

Solvent Screen

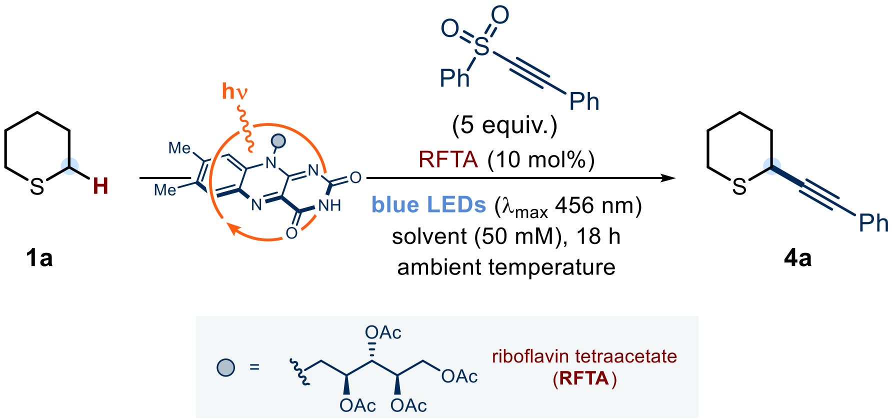

Entry	Solvent	Yield [a] (%)
1	Acetone	14
2	MeCN	14
3	CH_2Cl_2	16
4	DME	38
5	1,4-Dioxane	43
6	HFIP	15
7	1,2-DCE	28
8	DMC	28
9	DEC	34

[a] Yield determined by ^1H NMR using $MeNO_2$ as an internal standard.

Alkynylating Agent Screen

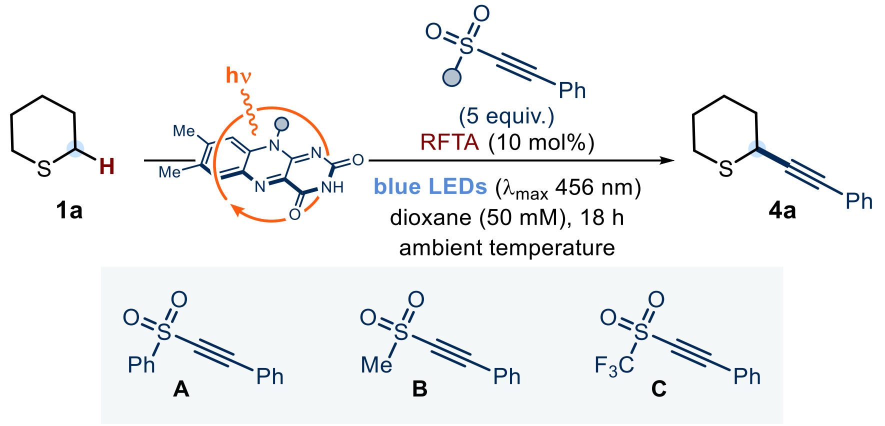

Entry	Alkynylating Agent	Yield [a] (%)
1	A	43
2	B	38
3	C	0

[a] Yield determined by ^{1}H NMR using $MeNO_2$ as an internal standard.

Riboflavin Tetraacetate

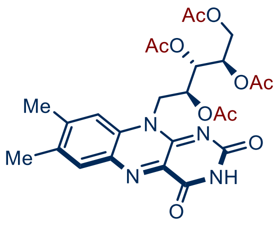

Riboflavin tetraacetate, RFTA

To a solution of pyridine (60 mL) and acetic anhydride (60 mL) was added riboflavin (5.00 g, 13.3 mmol) and the reaction stirred at 120 °C for 20 minutes. The solution was allowed to cool to room temperature, diluted with CH_2Cl_2 (150 mL), and poured into ice-cold 1M HCl (aq., 100 mL). The aqueous phase was separated and washed with CH_2Cl_2 (3 × 100 mL), before the combined organic phases were washed with 1M HCl (3 × 100 mL), and water (3 × 50 mL). The organic phase was then dried over $MgSO_4$, and concentrated *in vacuo*. The crude mixture was purified by column chromatography (silica; 2-4% MeOH in CH_2Cl_2) to afford the title compound (2.49 g, 4.57 mmol, 34%) as a yellow solid. **^{1}H NMR:** ^{1}H NMR (400 MHz, $CDCl_3$) δ 8.69 (s, 1H, N*H*), 8.01 (s, 1H, Ar *H*), 7.56 (s, 1H, Ar *H*), 5.70 – 5.62 (m, 1H, C*H*OAc), 5.49 – 5.36 (m, 2H, C*H*OAc), 4.89 (s, 2H, CH_2N), 4.42 (dd, *J* = 12.4, 2.8 Hz, 1H, CH_2OAc), 4.24 (dd, *J* = 12.4, 5.7 Hz, 1H, CH_2OAc), 2.56 (s, 3H, Ar CH_3), 2.44 (s, 3H, Ar CH_3), 2.28 (s, 3H, OAc), 2.21 (s, 3H, OAc), 2.07 (s, 3H, OAc), 1.75 (s, 3H, OAc). **^{13}C NMR:** ^{13}C NMR (101 MHz, $CDCl_3$) δ 170.8 (*C*O (OAc)), 170.4 (*C*O (OAc)), 170.0 (*C*O (OAc)), 169.9 (*C*O (OAc)), 159.5 (*C*O (imide)), 154.6 (*C*O (imide)), 150.8 (N*C*=N), 148.3 (Ar CCH_3), 137.1 (Ar CCH_3), 136.2 (N=*C*C=O), 134.8 (Ar *C*N), 133.1 (Ar *C*H), 131.4 (Ar *C*N), 115.7 (Ar *C*H), 70.6 9 (*C*HOAc), 69.6 (*C*HOAc), 69.1 (*C*HOAc), 62.0 (CH_2OAc), 45.2 (NCH_2), 21.6 9 (Ar CH_3), 21.2 (OAc), 20.9 (OAc), 20.8 (OAc), 20.5 (OAc), 19.6 (Ar CH_3). **HRMS** $C_{25}H_{27}O_{10}N_4$ $(M-H)^-$ Calculated 543.1733, found 543.1745.

The data are in accordance with the literature.[1]

Radical Traps

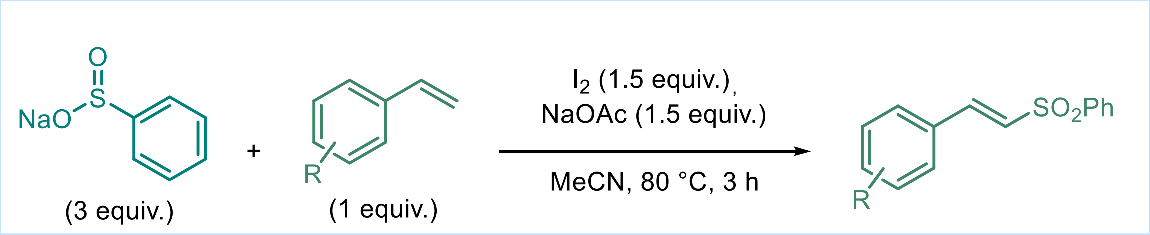

General Method A

Reactions were performed on a 2.0 mmol or 4.0 mmol scale. To a suspension of benzenesulfinic acid sodium salt (3.0 equiv) and sodium acetate (1.5 equiv) in MeCN (0.25 M), styrene derivative (1.0 equiv) was added followed by iodine (1.5 equiv). The mixture was heated under reflux for 3 h. The reaction mixture was allowed to cool to room temperature and excess iodine was quenched with a saturated aqueous sodium thiosulfate solution (5 mL). The reaction mixture was basified with saturated aqueous $NaHCO_3$ (5 mL), and the organic component extracted with EtOAc (3 × 10 mL). The combined organic extracts were washed with water (10 mL) and brine (10 mL), followed by drying over $MgSO_4$, and concentrated under reduced pressure. The crude product was purified by column chromatography.

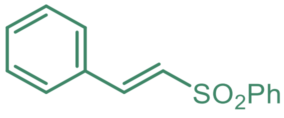

5b

(*E*)-(2-(Phenylsulfonyl)vinyl)benzene (5b)

Sulfone **5b** was prepared according to general method A. The crude product was purified using column chromatography (20% EtOAc in hexane) to give an off-white oil (874 mg, 3.56 mmol, 89%). **^{1}H NMR** (500 MHz, $CDCl_3$) δ 7.95 (dd, *J* = 7.4, 1.8 Hz, 2H, Ar C*H*), 7.69 (d, *J* = 15.4 Hz, 1H, PhC*H*=CH), 7.65 – 7.58 (m, 1H, Ar C*H*), 7.58 – 7.52 (m, 2H, Ar C*H*), 7.52 – 7.46 (m, 2H, Ar C*H*), 7.45 – 7.35 (m, 3H, Ar C*H*), 6.87 (d, *J* = 15.4 Hz, 1H, PhCH=C*H*). **^{13}C NMR** (126 MHz, $CDCl_3$) δ 142.4 (Ph*C*H=CH), 140.6 (Ar *C*), 133.3 (Ar *C*H), 132.2 (Ar *C*), 131.1 (Ar *C*H), 129.2 (Ar *C*H), 129.0 (Ar *C*H), 128.4 (Ar *C*H), 127.5 (Ar *C*H), 127.1 (PhCH=*C*H). **v_{max}**

(thin film/cm^{-1}): 1611, 1446, 1298, 1142, 1083, 969, 854, 816, 739, 716, 683, 616, 560, 534. **HRMS** $C_{14}H_{13}O_2S$ $(M+H)^+$ predicted 245.0631, found 245.0625.

The data are in accordance with the literature.[2]

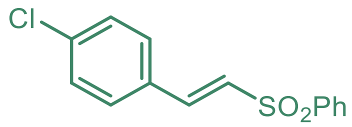

5c

(*E*)-1-Chloro-4-(2-(phenylsulfonyl)vinyl)benzene (5c)

Sulfone **5c** was prepared according to general method A. The crude product was purified using column chromatography (20% EtOAc in hexane) to give an off-white oil (522 mg, 1.87 mmol, 94%). **^{1}H NMR** (500 MHz, $CDCl_3$) δ 7.97 – 7.92 (m, 2H, Ar C*H*), 7.67 – 7.60 (m, 2H, Ar C*H* + ArC*H*=CH), 7.59 – 7.52 (m, 2H, Ar C*H*), 7.42 (d, *J* = 8.6 Hz, 2H, Ar C*H*), 7.36 (d, *J* = 8.6 Hz, 2H, Ar C*H*), 6.84 (d, *J* = 15.4 Hz, 1H, ArCH=C*H*). **^{13}C NMR** (126 MHz, $CDCl_3$) δ 140.9 (Ar*C*H=CH), 140.4 (Ar C), 137.2 (Ar *C*), 133.4 (Ar *C*H), 130.7 (Ar *C*), 129.6 (Ar *C*H), 129.3 (2 × Ar *C*H), 129.3 (Ar *C*H), 127.8 (ArCH=*C*H), 127.6 (Ar *C*H). **v_{max}** (thin film/cm^{-1}): 1611, 1485, 1446, 1305, 1186, 1142, 1081, 1010, 968, 858, 815, 785, 754, 719, 674, 581. **HRMS** $C_{14}H_{12}O_2SCl$ $(M+H)^+$ predicted 279.0241, found 279.0235

The data are in accordance with the literature.[2]

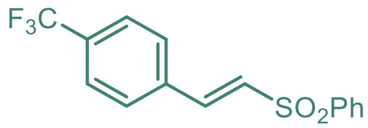

5d

(*E*)-1-(2-(Phenylsulfonyl)vinyl)-4-(trifluoromethyl)benzene (5d)

Sulfone **5d** was prepared according to general method A. The crude product was purified using column chromatography (20% EtOAc in hexane) to give a white solid (523 mg, 1.68 mmol, 84%). **^{1}H NMR** (400 MHz, $CDCl_3$) δ 7.99 – 7.93 (m, 2H, Ar C*H*), 7.71 (d, *J* = 15.5 Hz, 1H, ArC*H*=CH), 7.67 – 7.63 (m, 3H, Ar C*H*), 7.61 – 7.54 (m, 4H, Ar C*H*), 6.96 (d, *J* = 15.4 Hz, 1H, ArCH=C*H*). **^{13}C NMR** (101 MHz, $CDCl_3$) δ 140.4 (Ar*C*H=CH), 140.1 (Ar *C*), 135.7 (Ar *C*), 133.7 (Ar *C*H), 132.6 (q, *J* = 32.8 Hz, Ar *C*CF_3), 130.0 (Ar *C*H), 129.4 (Ar *C*H), 128.7 (Ar *C*H),

127.8 (ArCH=*C*H), 126.0 (q, *J* = 3.9 Hz, Ar*C*HCCF$_3$), 123.5 (q, *J* = 270.2 Hz, *C*F$_3$). **^{19}F NMR** (376 MHz, CDCl$_3$) δ -63.02. **ν_{max}** (thin film/cm^{-1}): 1616, 1412, 1304, 1142, 1110, 1082, 1062, 984, 861, 814, 732, 715, 570. **HRMS** $C_{15}H_{11}O_2SF_3$ $(M+H)^+$ predicted 312.0426, found 312.0421.

The data are in accordance with the literature.[4]

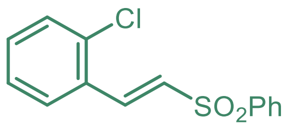

5e

(*E*)-1-Chloro-2-(2-(phenylsulfonyl)vinyl)benzene (5e)

Sulfone **5e** was prepared according to general method A. The crude product was purified using column chromatography (20% EtOAc in hexane) to give a white solid (518 mg, 1.86 mmol, 93%). **^{1}H NMR** (400 MHz, CDCl$_3$) δ 8.09 (d, *J* = 15.4 Hz, 1H, ArC*H*=CH), 8.01 – 7.93 (m, 2H, Ar CH), 7.66 – 7.61 (m, 1H, Ar CH), 7.60– 7.53 (m, 2H, Ar CH), 7.51 (dd, *J* = 7.8, 1.7 Hz, 1H, Ar CH), 7.43 (dd, *J* = 8.0, 1.4 Hz, 1H, Ar CH), 7.33 (td, *J* = 7.7, 1.7 Hz, 1H, Ar CH), 7.30 – 7.22 (m, 1H, Ar CH), 6.90 (d, *J* = 15.4 Hz, 1H, ArCH=C*H*). **^{13}C NMR** (101 MHz, CDCl$_3$) δ 140.2 (Ar *C*), 138.3 (Ar*C*H=CH), 135.2 (Ar *C*), 133.5 (Ar *C*H), 131.8 (Ar *C*H), 130.6 (Ar *C*), 130.3 (ArCH=*C*H), 129.9 (Ar *C*H), 129.3 (Ar *C*H), 128.2 (Ar *C*H), 127.7 (Ar *C*H), 127.1 (Ar *C*H). **ν_{max}** (thin film/cm^{-1}): 1605, 1447, 1301, 1143, 1084, 970, 853, 821, 744, 722, 685, 614, 565, 544, 461. **HRMS** $C_{14}H_{12}O_2SCl$ $(M+H)^+$ predicted 279.0241, found 279.0229.

The data are in accordance with the literature.[3]

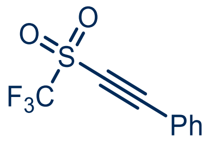

S1

(((Trifluoromethyl)sulfonyl)ethynyl)benzene (S1)

A 100 mL flask was charged with a solution of phenylacetylene (220 mg, 2.16 mmol) in dry Et_2O (20 mL) under nitrogen. The solution was cooled to −78 ºC and *n*BuLi (0.86 mL of 2.5 M in hexane, 1.08 mmol, 1.0 eq.) was added dropwise. The mixture was allowed to stir for 30 minutes. Trifluoromethylsulfonic anhydride (680 mg, 1.19 mmol) was added dropwise and the reaction stirred for a further 20 minutes. The reaction mixture was allowed to warm to room temperature, washed with saturated $NaHCO_3$ (20 mL), 1 M HCl (20 mL) then brine (20 mL), dried over $MgSO_4$ and concentrated under reduced pressure to yield the crude product. The residue was purified using column chromatography (10% EtOAc in hexane) to give a yellow oil (322 mg, 1.38 mmol, 64%). **^{1}H NMR** (500 MHz, $CDCl_3$) δ 7.73 – 7.67 (m, 2H, Ar CH), 7.67 – 7.59 (m, 1H, Ar CH), 7.52 – 7.46 (m, 2H, Ar CH). **^{13}C NMR** (126 MHz, $CDCl_3$) δ 133.7 (Ar CH), 133.3 (Ar CH), 129.0 (Ar CH), 119.1 (q, J = 323.2 Hz, CF_3), 115.7 (Ar C), 100.7 ($CCSO_2$), 77.3 ($CCSO_2$).**^{19}F NMR** (471 MHz, $CDCl_3$) δ -79.6 (CF_3). **v_{max}** (thin film/cm^{-1}): 2178, 1592, 1443, 1323, 1152, 1081, 847, 812, 757, 704, 635. **HRMS** $C_9H_5O_2SF_3$ $(M)^+$ predicted 233.9962, found 233.9965.

The data are in accordance with the literature.[5]

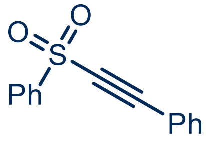

6

((Phenylethynyl)sulfonyl)benzene (6)

A mixture of phenylpropiolic acid (584 mg, 4.0 mmol), benzenesulfinic acid sodium salt (2.64 g, 16 mmol) and iodine (1.01 g, 4.0 mmol) in THF (32 mL) was stirred at room temperature for

10 minutes. Subsequently, *tert*-butylhydroperoxide (3.09 mL, 70 wt.% aq solution, 24 mmol) was added and the mixture was stirred at room temperature for a further 12 h. Upon completion, the reaction was quenched with $Na_2S_2O_3$ (aq, 20 mL), followed by extraction with EtOAc (3 × 50 mL). The organic layer was dried with anhydrous $MgSO_4$ and concentrated under reduced pressure. The crude product was purified using column chromatography (10% EtOAc in hexane) to give a yellow crystalline solid (445 mg, 1.83 mmol, 46%). **^{1}H NMR** (400 MHz, $CDCl_3$): δ 8.12 – 8.05 (m, 2H, Ar CH), 7.72 – 7.65 (m, 1H, Ar CH), 7.63 – 7.56 (m, 2H, Ar CH), 7.54 – 7.49 (m, 2H, Ar CH), 7.48 – 7.44 (m, 1H, Ar CH), 7.39 – 7.33 (m, 2H, Ar CH). **^{13}C NMR** (101 MHz, $CDCl_3$): δ 141.8 (Ar C), 134.2 (Ar CH), 132.8 (Ar CH), 131.6 (Ar CH), 129.4 (Ar CH), 128.7 (Ar CH), 127.4 (Ar CH), 117.9 (Ar C), 93.5 ($CCSO_2$), 85.3 ($CCSO_2$). **ν_{max}** (thin film/cm^{-1}): 2180, 1445, 1321, 1154, 1083, 850, 752, 724, 683, 654, 570, 539, 478. **MS** (ESI+): m/z 265.0306 $(M+Na)^+$

The data are in accordance with the literature.[6]

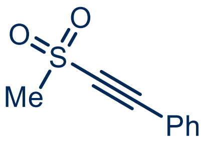

S2

((Methylsulfonyl)ethynyl)benzene (S2)

A mixture of sodium methanesulfinate (1.00 g, 10.0 mmol), phenylacetylene (0.537 mL, 4.90 mmol), and iodine (1.87 g, 7.35 mmol) in water (70 mL) was stirred at room temperature for 2 hours. The reaction mixture was then quenched with $Na_2S_2O_3$ (aq, 20 mL), followed by extraction with EtOAc (3 × 50 mL). The organic layer was dried with anhydrous $MgSO_4$ and concentrated under reduced pressure. The resulting residue was refluxed with K_2CO_3 (1.38 g, 10.0 mmol) in anhydrous acetone (40 mL) for 18 hours. The reaction mixture was washed with water (80 mL) and extracted with CH_2Cl_2 (3 × 30 mL). The combined organic extracts were washed with brine and dried over $MgSO_4$. The solvent was removed under reduced pressure and the residue was purified by column chromatography (CH_2Cl_2) to give an orange solid (387

mg, 2.15 mmol, 44%). **^{1}H NMR** (400 MHz, $CDCl_3$) δ 7.57 – 7.62 (m, 2H, Ar CH), 7.49 – 7.55 (m, 1H, Ar CH), 7.38 – 7.45 (m, 2H, Ar CH), 3.30 (s, 3H, SO_2CH_3). **^{13}C NMR** (101 MHz, $CDCl_3$) δ 132.8 (Ar *C*H), 131.7 (Ar *C*H), 128.7 (Ar *C*H), 117.4 (Ar *C*), 91.5 (*C*≡CSO_2), 84.3 (C≡*C*SO_2), 46.7 (SO_2*C*H_3). **v_{max}** (thin film/cm^{-1}): 2168, 1589, 1443, 1342, 1151, 1100, 837, 802, 761, 711, 630 **MS** (ESI^+): m/z 203.0140 $(M+Na)^+$

The data are in accordance with the literature.[7]

Starting Material Sulfides

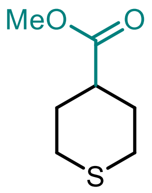

1f

Methyl tetrahydrothiopyran-4-carboxylate (1f)

To a stirred solution of tetrahydrothiopyran-4-carboxylic acid (146 mg, 1.0 mmol, 1.0 equiv.) in MeOH (5 mL) at 0 °C was added thionyl chloride (0.36 mL, 5.0 mmol, 5.0 equiv.) dropwise. After stirring for 18 hours, the reaction mixture quenched was with saturated Na_2CO_3 (5 mL) and extracted with EtOAc (3 × 10 mL). The combined organic phases were then washed with saturated Na_2CO_3 (3 × 5 mL), followed by brine (10 mL), dried over $MgSO_4$ and filtered before being concentrated *in vacuo*. The crude product was purified by column chromatography (50% EtOAc in hexane) to give a yellow oil (143 mg, 0.89 mmol, 89%). **^{1}H NMR** (500 MHz, $CDCl_3$) δ 3.63 (s, 3H, $OC\mathit{H}_3$), 2.66 – 2.55 (m, 4H, $S(C\mathit{H}_2)_2$), 2.34 (tt, *J* = 10.9, 3.4 Hz, 1H, $C\mathit{H}CO_2Me$), 2.20 – 2.11 (m, 2H, $(C\mathit{H_a}H_b)_2$), 1.88 – 1.74 (m, 2H, $(CH_a\mathit{H_b})_2$). **^{13}C NMR** (101 MHz, $CDCl_3$) δ 175.1 (*C*=O), 51.8 ($O\mathit{C}H_3$), 42.4 ($\mathit{C}HCO_2Me$), 29.8 ($SCH_2\mathit{C}H_2$), 27.6 ($S\mathit{C}H_2CH_2$). **HRMS** $C_7H_{11}O_2S$ $(M\text{-}H)^-$ Calculated 159.0485, found 158.9772.

The data are in accordance with the literature.[8]

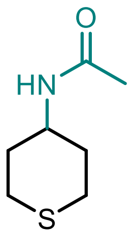

1j

***N*-Acetyltetrahydrothiopyran-4-amine (1j)**

To a stirred solution of 4-tetrahydrothiopyran amine (117 mg, 1.0 mmol, 1.0 equiv.) in pyridine (1 mL) was added acetic anhydride (0.11 mL, 1.1 mmol, 1.1 equiv.) The reaction was stirred

at room temperature for 4 hours before being quenched by slow addition of MeOH (2 mL). The resulting mixture was diluted with CH_2Cl_2 (5 mL) and the aqueous phase washed with CH_2Cl_2 (3 × 10 mL). The combined organic phases were then washed with brine, and dried over $MgSO_4$ before being concentrated *in vacuo*. The crude product was purified by column chromatography (5% MeOH in CH_2Cl_2) to give a white solid (111 mg, 0.70 mmol, 70%). **^{1}H NMR** (400 MHz, $CDCl_3$) δ 5.33 (bs, 1H, N*H*), 3.80 (tdt, *J* = 11.5, 7.9, 3.7 Hz, 1H, C*H*NH), 2.76 (ddd, *J* = 14.3, 11.8, 2.6 Hz, 2H, 2 × SC$H_a$$H_b$), 2.67 – 2.57 (m, 2H, 2 × SC$H_a$$H_b$), 2.21 (dq, *J* = 12.1, 3.7 Hz, 2H, 2 × $SCH_2$$H_a$$H_b$), 1.97 (s, 3H, Ac), 1.55 – 1.45 (m, 2H, 2 × $SCH_2$$H_a$$H_b$). **^{13}C NMR** (101 MHz, $CDCl_3$) δ 169.1 (*C*=O), 47.8 (*C*HNH), 34.4 (C(O)CH_3), 28.0 (SCH_2CH_2), 23.7 (SCH_2). **HRMS** $C_7H_{13}NOSNa$ $(M+Na)^+$ Calculated 182.0610, found 182.0611.

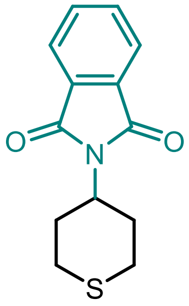

1k

2-(Tetrahydrothiopyran-4-yl)isoindoline-1,3-dione (1k)

To a solution tetrahydrothiopyran-4-amine (117 mg, 1.0 mmol, 1.0 equiv.) in $H_2O/CHCl_3$ (1:1, 4 mL) was added *N*-carbethoxyphthalimide (220 mg, 1.0 mmol, 1.0 equiv.) and Na_2CO_3 (106 mg, 1.0 mmol, 1.0 equiv.), and the reaction was stirred for 2 hours. The reaction mixture was quenched with 1M HCl (5 mL), and extracted with $CHCl_3$ (3 × 5 mL). The combined organic phases were washed with brine, and dried over $MgSO_4$ before concentration *in vacuo*. The crude product was purified by column chromatography (30% EtOAc in hexane) to give a white solid (146 mg, 0.58 mmol, 58%). **^{1}H NMR** (400 MHz, $CDCl_3$) δ 7.89 – 7.78 (m, 2H, Ar *H*), 7.78 – 7.66 (m, 2H, Ar *H*), 4.10 (tt, *J* = 12.2, 3.5 Hz, 1H, C*H*N), 2.89 – 2.79 (m, 2H, 2 × SC$H_a$$H_b$), 2.77 – 2.70 (m, 2H, 2 × SC$H_a$$H_b$), 2.62 (qd, *J* = 12.6, 3.4 Hz, 2H, 2 × CHCH_aCH_b), 2.07 – 1.97 (m, 2H, 2 × CHCH_aCH_b). **^{13}C NMR** (101 MHz, $CDCl_3$) δ 168.2 (*C*O), 134.1 (Ar *C*H), 132.0 (Ar

C), 123.4 (Ar *C*H), 50.3 (*C*HN), 31.6 (CH*C*H_2), 29.2 (S*C*H_2). **HRMS** $C_{13}H_{13}NO_2SNa$ $(M+Na)^+$ Calculated 270.0559, found 270.0563.

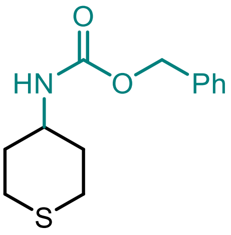

1l

Benzyl (tetrahydrothiopyran-4-yl)carbamate (1l)

To a solution of tetrahydrothiopyran-4-amine (117 mg, 1.0 mmol, 1.0 equiv.) and triethylamine (0.15 mL, 1.1 mmol, 1.1 equiv.) in THF (10 mL) was added benzylchloroformate (0.15 mL, 1.1 mmol, 1.1 equiv.). The reaction mixture was stirred for 18 hours, and quenched with a saturated aqueous solution of $NaHCO_3$ (10 mL). The reaction mixture was extracted with CH_2Cl_2 (3 × 10 mL), and the organic phase washed with brine (10 mL), followed by drying over $MgSO_4$. The crude product was purified by column chromatography (20% EtOAc in hexane) to give a white solid (171 mg, 0.69 mmol, 69%) **^{1}H NMR** (400 MHz, $CDCl_3$) δ 7.41 – 7.30 (m, 5H, Ar *H*), 5.09 (s, 2H, CH_2Ph), 4.68 (s, 1H, N*H*), 3.61 – 3.40 (m, 1H, C*H*N), 2.81 – 2.57 (m, 4H, 2 × SCH_2), 2.24 (dq, *J* = 12.5, 3.7 Hz, 2H, 2 × CH_aH$_b$CHN), 1.62 – 1.48 (m, 4H, 2 × CH$_a$$H_b$CHN + H_2O). **^{13}C NMR** (101 MHz, $CDCl_3$) δ 155.5 (*C*O), 136.6 (Ar *C*), 128.7 (Ar *C*H x 2), 128.3 (Ar *C*H), 66.9 (O*C*H$_2$Ph), 49.5 (*C*HNCbz), 34.6 ((*C*H$_2$)$_2$CHN), 27.9 (S(*C*H$_2$)$_2$). **HRMS** $C_{13}H_{17}NO_2SNa$ $(M+Na)^+$ Calculated 274.0872, found 274.0873.

The data are in accordance with the literature.[9]

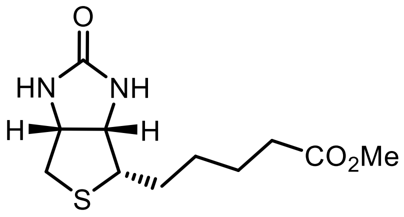

1m

D-Biotin methyl ester (1m)

Acetyl chloride (0.29 mL, 3.3 mmol, 3.3 equiv.) was stirred in MeOH (2 mL) at room temperature for 10 minutes. The resulting solution was added slowly to a suspension of D-biotin (244 mg, 1.0 mmol, 1.0 equiv.) in MeOH (6 mL) and the reaction allowed to stir for 2 hours. The reaction mixture was concentrated *in vacuo*, and partitioned between saturated $NaHCO_3$ (10 mL) and 5% MeOH in CH_2Cl_2 (10 mL). The aqueous phase was washed with 5% MeOH in CH_2Cl_2 (4 × 10 mL) and the organic phase was washed with brine, filtered over $MgSO_4$, and evaporated to dryness. The crude product was purified by column chromatography (10% MeOH in CH_2Cl_2) to give a white solid (225 mg, 0.87 mmol, 87%). **^{1}H NMR** (500 MHz, $CDCl_3$) δ 5.36 – 5.32 (m, 1H, N*H*), 4.99 (s, 1H, N*H*), 4.52 (ddt, *J* = 7.7, 5.0, 1.3 Hz, 1H, C*H*NH), 4.37 – 4.29 (m, 1H, C*H*NH), 3.71 – 3.65 (m, 3H, OCH_3), 3.16 (ddd, *J* = 8.1, 6.4, 4.5 Hz, 1H, SC*H*), 2.97 – 2.89 (m, 1H, SC$H_a$$H_b$), 2.74 (d, *J* = 12.8 Hz, 1H, SC$H_a$$H_b$), 2.34 (t, *J* = 7.4 Hz, 2H, C$H_2$$CO_2$Me), 1.76 – 1.65 (m, 4H, CH_2), 1.46 (tt, *J* = 10.2, 4.3 Hz, 2H, CH_2). **^{13}C NMR** (126 MHz, $CDCl_3$) δ 174.2 (*C*O_2Me), 163.2 (N*C*(O)N), 62.0 (*C*HN), 60.2 (*C*HN), 55.4 (S*C*H), 51.7 (O*C*H_3), 40.7 (S*C*H_2), 33.8 (*C*H_2), 28.4 (*C*H_2), 28.4 (*C*H_2), 24.9 (*C*H_2). **HRMS** $C_{11}H_{18}N_2O_3SNa$ $(M+Na)^+$ Calculated 281.0930, found 281.0933.

The data are in accordance with the literature.[10]

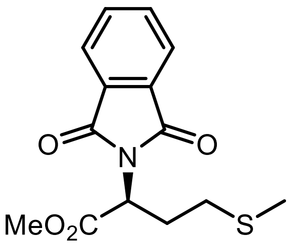

1n

Methyl (*S*)-2-(1,3-dioxoisoindolin-2-yl)-4-(methylthio)butanoate (1n)

To a mixture of methionine methyl ester hydrochloride salt (1.40 g, 7.0 mmol, 1.0 equiv.) in $CHCl_3$ and water (1:1, 20 mL) was added Na_2CO_3 (0.74 g, 7.0 mmol, 1.0 equiv.) and *N*-ethoxycarbonylphthalimide (1.53 g, 7.0 mmol, 1.0 equiv.). The mixture was stirred for 2 hours before addition of 1 M HCl (30 mL). The phases was separated, and the aqueous phase washed with CH_2Cl_2 (3 × 20 mL). The combined organic phases were then washed with 1 M HCl (3 × 20 mL), brine (20 mL), and dried over $MgSO_4$ before being concentrated *in vacuo*. The crude product was then purified by column chromatography (30% EtOAc in hexane) to give a colourless oil (849 mg, 3.1 mmol, 41%) that solidified to an amorphous solid over time. **^{1}H NMR** (400 MHz, $CDCl_3$) δ 7.87 (dd, *J* = 5.5, 3.1 Hz, 2H, Ar *H*), 7.75 (dd, *J* = 5.4, 3.1 Hz, 2H, Ar *H*), 5.10 (dd, *J* = 8.1, 6.0 Hz, 1H, C*H*N), 3.73 (s, 3H, OCH_3), 2.61 – 2.43 (m, 4H, SCH_2 + SCH$_2$CH_2), 2.08 (s, 3H, SCH_3). **^{13}C NMR** (101 MHz, $CDCl_3$) δ 169.7 (*C*O_2), 167.8 (N*C*O), 134.4 (Ar *C*H), 131.9 (Ar *C*), 123.8 (Ar *C*H), 53.0 (O*C*H_3), 51.0 (*C*HN), 31.0 (S*C*H_2), 28.2 (SCH$_2$*C*H_2), 15.5 (S*C*H_3). **HRMS** $C_{14}H_{15}NO_4SNa$ $(M+Na)^+$ Calculated 316.0619, found 316.0623.

The data are in accordance with the literature.[11]

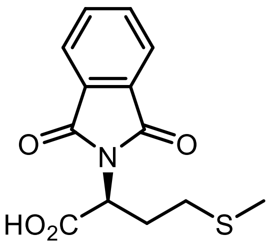

S3

(*S*)-2-(1,3-Dioxoisoindolin-2-yl)-4-(methylthio)butanoic acid (S3)

To a mixture of methionine (2.24 g, 15.0 mmol, 1.0 equiv.) in $CHCl_3$ and water (1:1, 20 mL) was added Na_2CO_3 (1.59 g, 15.0 mmol, 1.0 equiv.) and *N*-ethoxycarbonylphthalimide (3.29 g, 15.0 mmol, 1.0 equiv.). The mixture was stirred for 2 hours before addition of 1 M HCl (30 mL). The phases was separated, and the aqueous phase washed with CH_2Cl_2 (3 × 20 mL). The combined organic phases were then washed with 1 M HCl (3 × 20 mL), and dried over $MgSO_4$ before being concentrated *in vacuo*. The crude product was then purified by column chromatography (49% EtOAc, 1% AcOH in hexane) to give a white solid (4.04 g, 14.5 mmol, 96%). **^{1}H NMR** (400 MHz, $CDCl_3$) δ 7.91 – 7.80 (m, 2H, Ar C*H*), 7.74 (dd, *J* = 5.5, 3.1 Hz, 2H, Ar C*H*), 5.19 – 5.11 (m, 1H, NC*H*), 2.64 – 2.41 (m, 4H, SCH_2 + SCH_2CH_2), 2.07 (s, 3H, SCH_3). **^{13}C NMR** (101 MHz, $CDCl_3$) δ 174.1 (CO_2H), 167.8 (*C*(O)N*C*(O)), 134.4 (Ar *C*H), 131.9 (Ar *C*), 123.8 (Ar *C*H), 50.8 (N*C*H), 31.0 (SCH_2CH_2), 28.0 (SCH_2), 15.4 (SCH_3). **HRMS** $C_{13}H_{13}NO_4S$ $(M-H)^-$ Calculated 278.0574, found 278.0503.

The data are in accordance with the literature.[12]

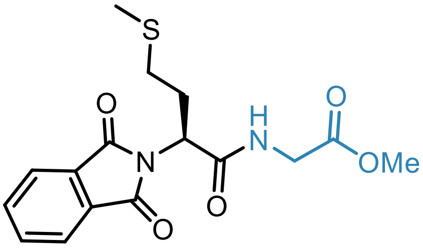

1o

Methyl (*S*)-(2-(1,3-dioxoisoindolin-2-yl)-4-(methylthio)butanoyl)glycinate (Phth-Met-Gly-OMe, 1o)

To a solution of (*S*)-2-(1,3-dioxoisoindolin-2-yl)-4-(methylthio)butanoic acid (838 mg, 3.0 mmol, 1.0 equiv.) in CH_2Cl_2 (20 mL) was added EDC.HCl (690 mg, 3.3 mmol, 1.2 equiv.), HOBT (446 mg, 3.3 mmol, 1.1 equiv.) and NEt_3 (1.5 mL, 9.0 mmol, 3.0 equiv.) and the mixture was allowed to stir for 10 minutes before portionwise addition of glycine methyl ester hydrochloride salt (414 mg, 3.3 mmol, 1.1 equiv.). The reaction was stirred overnight at room temperature, and quenched by addition of 1M HCl (20 mL) after 18 hours. The phases were separated, and the organic phases were washed with 1M HCl (3 × 20 mL), followed by saturated $NaHCO_3$ (3 × 20 mL), and brine (20 mL) before being dried over $MgSO_4$. The crude product was purified by column chromatography (40-60% EtOAc in hexane) to give a colourless oil (263 mg, 0.75 mmol, 25%). **^{1}H NMR** (400 MHz, $CDCl_3$) δ 7.88 (dd, *J* = 5.5, 3.0 Hz, 2H, Ar *H*), 7.76 (dd, *J* = 5.5, 3.1 Hz, 2H, Ar *H*), 6.67 (t, *J* = 5.1 Hz, 1H, N*H*), 5.10 – 5.01 (m, 1H, C*H*N), 4.05 (d, *J* = 5.0 Hz, 2H, CH_2N), 3.73 (s, 3H, OCH_3), 2.64 – 2.46 (m, 4H, SCH_2 + SCH$_2$CH_2), 2.08 (s, 3H, SCH_3). **^{13}C NMR** (101 MHz, $CDCl_3$) δ 170.1 (CO_2), 168.9 (*C*(O)NH), 168.2 (*C*(O)N*C*(O)), 134.6 (Ar *C*H), 131.7 (Ar *C*), 123.9 (Ar*C*H), 53.5 (N*C*H), 52.6 (O*C*H_3), 41.6 (N*C*H_2), 31.1 (SCH$_2$*C*H$_2$), 28.1 (S*C*H$_2$), 15.6 (S*C*H$_3$). **HRMS** $C_{16}H_{18}N_2O_4SNa$ $(M+Na)^+$ Calculated 373.0834, found 373.0838.

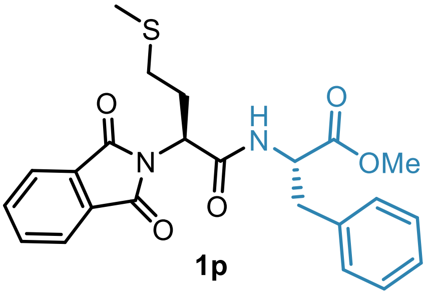

1p

Methyl ((*S*)-2-(1,3-dioxoisoindolin-2-yl)-4-(methylthio)butanoyl)-L-phenylalaninate (Phth-Met-Phe-OMe) (1p)

To a solution of (*S*)-2-(1,3-dioxoisoindolin-2-yl)-4-(methylthio)butanoic acid (139 mg, 0.5 mmol, 1.0 equiv.) in CH_2Cl_2 (20 mL) was added HBTU (238 mg, 0.6 mmol, 1.2 equiv.), and DIPEA (0.25 mL, 1.5 mmol, 3.0 equiv.) and the mixture was allowed to stir for 10 minutes before portionwise addition of phenylalanine methyl ester hydrochloride salt (108 mg, 0.5 mmol, 1.0 equiv.). The reaction was stirred overnight at room temperature, and quenched by addition of H_2O (20 mL) after 18 hours. The phases were separated, and the aqueous phase was washed with CH_2Cl_2 (3 × 10 mL). The organic phase was then washed with brine (20 mL) before being dried over $MgSO_4$. The crude product was concentrated *in vacuo* and purified by column chromatography (40-60% EtOAc in hexane) to give a colourless oil (142 mg, 0.32 mmol, 65%). **^{1}H NMR** (400 MHz, $CDCl_3$) δ 7.87 (dd, *J* = 5.5, 3.1 Hz, 2H, Ar CH_{Phth}), 7.80 – 7.70 (m, 2H, Ar CH_{Phth}), 7.16 – 7.04 (m, 3H, Ar CH_{phenyl}), 7.03 – 6.99 (m, 2H, Ar CH_{phenyl}), 6.44 (d, *J* = 7.7 Hz, 1H, N*H*), 5.01 – 4.91 (m, 1H, NC*H*), 4.86 (dt, *J* = 7.8, 5.9 Hz, 1H, NHC*H*), 3.70 (s, 3H, OCH_3), 3.13 (dd, *J* = 13.9, 5.7 Hz, 1H, CH_aH$_b$Ph), 3.06 (dd, *J* = 13.9, 6.2 Hz, 1H, CH$_a$$H_b$Ph) 2.56 – 2.36 (m, 4H, SCH_2 + SCH$_2$CH_2), 2.03 (s, 3H, SCH_3). **^{13}C NMR** (101 MHz, $CDCl_3$) δ 171.7 (*C*O_2), 168.1 (*C*(O)NH), 168.0 (*C*(O)N*C*(O)), 135.6 (Ar C_{phenyl}), 134.5 (Ar CH_{Phth}), 131.7 (Ar C_{Phth}), 129.3 (Ar CH_{phenyl}), 128.6 (Ar CH_{phenyl}), 127.2 (Ar CH_{phenyl}), 123.8 (Ar CH_{Phth}), 53.4 (NH*C*H), 53.3 (N*C*H), 52.6 (O*C*H_3), 37.8 (*C*H_2Ph), 31.0 (SCH$_2$*C*H_2), 27.8 (S*C*H_2), 15.5 (S*C*H_3). **HRMS** $C_{23}H_{24}N_2O_5SNa$ $(M+Na)^+$ Calculated 463.1298, found 463.1305.

The data are in accordance with the literature.[11]

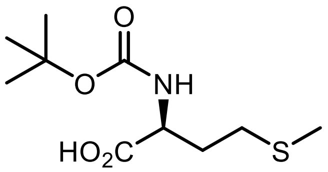

S4

(*tert*-Butoxycarbonyl)-L-methionine (Boc-Met-OH) (S4)

To a solution of L-methionine (448 mg, 3.0 mmol, 1.0 equiv.) in THF (5 mL) and 1M aqueous NaOH (5 mL) was added Boc_2O (786 mg, 3.6 mmol, 1.2 equiv.) and the reaction stirred at room temperature for 18 hours. The pH of the reaction was adjusted to pH 2 with 0.5M HCl and extracted with CH_2Cl_2. The combined organic extracts were dried over $MgSO_4$ and concentrated *in vacuo*. The crude product was then purified by column chromatography (10% MeOH in CH_2Cl_2) to give a colourless oil (587 mg, 2.34 mmol, 78%). **^{1}H NMR** (400 MHz, CD_3CN) δ 5.68 – 5.57 (m, 1H, N*H*), 4.21 (dt, *J* = 13.6, 6.5 Hz, 1H, C*H*), 2.59 – 2.42 (m, 2H, SC*H$_2$*), 2.11 – 1.97 (s, 4H, $SCH_2CH_aH_b$ + SC*H$_3$*), 1.91 – 1.82 (m, 1H, $SCH_2CH_aH_b$), 1.40 (s, 9H, C(C*H$_3$*)$_3$). **^{13}C NMR** (101 MHz, CD_3CN) δ 173.8 (*C*O$_2$H), 156.4 (N*C*(O)O), 79.6 (*C*(CH$_3$)$_3$), 53.0 (*C*H), 31.4 (SCH$_2$*C*H$_2$), 30.4 (S*C*H$_2$), 28.1 (C(*C*H$_3$)$_3$), 14.9 (S*C*H$_3$).

The data are in accordance with the literature.[13]

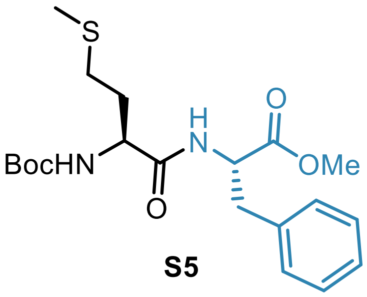

S5

Methyl (*tert*-butoxycarbonyl)-L-methionyl-L-phenylalaninate (Boc-Met-Phe-OMe) (S5)

To a solution of (*tert*-butoxycarbonyl)-L-methionine (374 mg, 1.5 mmol, 1.0 equiv.) in CH_2Cl_2 (30 mL) was added HBTU (683 mg, 1.8 mmol, 1.2 equiv.), and DIPEA (0.75 mL, 4.5 mmol, 3.0 equiv.) and the mixture was allowed to stir for 10 minutes before portionwise addition of phenylalanine methyl ester hydrochloride salt (356 mg, 1.65 mmol, 1.1 equiv.). The reaction

was stirred overnight at room temperature, and quenched by addition of H_2O (20 mL) after 18 hours. The phases were separated, and the aqueous phase was washed with CH_2Cl_2 (3 × 10 mL), and the organic phase washed with brine (20 mL) before being dried over $MgSO_4$. The crude product was concentrated *in vacuo* and purified by column chromatography (50% EtOAc in hexane) to give an off white amorphous solid (364 mg, 0.89 mmol, 59%). **^{1}H NMR** (400 MHz, $CDCl_3$) δ 7.36 – 7.22 (m, 3H, Ar C*H*), 7.17 – 7.10 (m, 2H, Ar C*H*), 6.63 (d, *J* = 7.9 Hz, 1H, N*H*), 5.18 (s, 1H, N*H*), 4.87 (dt, *J* = 7.9, 6.0 Hz, 1H, C(O)NHC*H*), 4.32 – 4.23 (m, 1H, BocNHC*H*), 3.74 (s, 3H, OCH_3), 3.21 – 3.06 (m, 2H, CH_2Ph), 2.55 (t, *J* = 7.1 Hz, 2H, SCH_2), 2.12 – 1.80 (m, 5H, SCH_3 + SCH_2CH_2), 1.46 (s, 9H, $C(CH_3)_3$). **^{13}C NMR** (101 MHz, $CDCl_3$) δ 171.7 (CO_2), 171.2 (*C*(O)N), 155.5 (O*C*(O)N), 135.7 (Ar C_{phenyl}), 129.4 (Ar CH_{phenyl}), 128.8 (Ar CH_{phenyl}), 127.3 (Ar CH_{phenyl}), 80.24 ($OC(CH_3)_3$), 53.4 (NH*C*H), 53.3 (BocNH*C*H), 52.5 (OCH_3), 38.0 (CH_2Ph), 31.7 (SCH_2CH_2), 30.2 (SCH_2), 28.4 ($OC(CH_3)_3$), 15.2 (SCH_3).

The data are in accordance with the literature.[14]

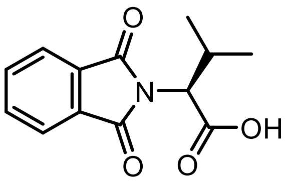

S6

(*S*)-2-(1,3-Dioxoisoindolin-2-yl)-3-methylbutanoic acid (Phth-Val-OH) (S6)

To a solution of L-valine (235 mg, 2.0 mmol, 1.0 equiv.) and *N*-ethoxyphthalimide (440 mg, 2.0 mmol, 1.0 mmol) in $CHCl_3/H_2O$ (1:1, 7 mL) was added Na_2CO_3 and stirred for 3 hours. The reaction mixture was partitioned between CH_2Cl_2 (10 mL) and H_2O (10 mL), the aqueous phase washed with CH_2Cl_2 (2 × 10 mL), and the pH of the aqueous phase adjusted to pH 2 with 1M HCl and extracted with CH_2Cl_2 (3 × 10 mL). The organic phases were then dried over $MgSO_4$, and evaporated to dryness to give an off-white oil (240 mg, 0.97 mmol, 49%) which was carried forward without further purification. **^{1}H NMR** (400 MHz, MeOD) δ 7.91 – 7.87 (m, 2H, Ar C*H*), 7.86 – 7.82 (m, 2H, Ar C*H*), 4.52 (d, *J* = 8.2 Hz, 1H, NC*H*), 2.69 (dh, *J* = 8.2, 6.8 Hz, 1H, C*H*$(CH_3)_2$), 1.15 (d, *J* = 6.7 Hz, 3H, CH_3), 0.89 (d, *J* = 6.8 Hz, 3H, CH_3). **^{13}C NMR** (101

MHz, MeOD) δ 172.0 (CO_2H), 169.3 (*C*(O)N*C*(O)), 135.7 (Ar *C*H), 132.9 (Ar *C*), 124.4 (Ar *C*H), 58.7 (N*C*H), 29.7 (*C*H(CH_3)$_2$), 21.5 (*C*H_3), 19.9 (*C*H_3). **HRMS** $C_{13}H_{13}NO_4Na$ $(M+Na)^+$ Calculated 270.0739, found 270.0737.

The data are in accordance with the literature.[15]

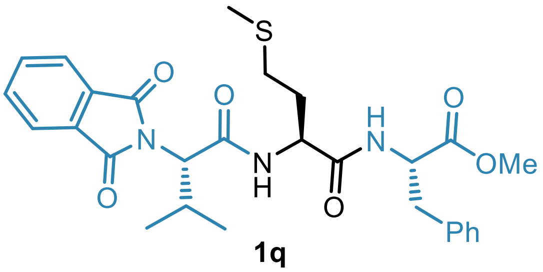

1q

Methyl ((*S*)-2-(1,3-dioxoisoindolin-2-yl)-3-methylbutanoyl)-L-methionyl-L-phenylalaninate (Phth-Val-Met-Phe-OMe) (1q)

To a solution of methyl (*tert*-butoxycarbonyl)-L-methionyl-L-phenylalaninate (364 mg, 0.89 mmol, 1.0 equiv.) in CH_2Cl_2 (5 mL) was added trifluoroacetic acid (5 mL) slowly over 5 minutes. The mixture was stirred until no starting material was detected by TLC (roughly 2-3 hours). The volatiles were removed *in vacuo* to give an off-white oil. To a solution of (*S*)-2-(1,3-dioxoisoindolin-2-yl)-3-methylbutanoic acid (240 mg, 1.0 mmol, 1.1 equiv.) and DIPEA (0.5 mL, 3.0 mmol, 3.0 equiv.) in CH_2Cl_2 (15 mL) at 0 °C was added HATU (418 mg, 1.2 mmol, 1.2 equiv.) and the mixture was allowed to stir for 10 minutes before portionwise addition of the previously deprotected amine. The reaction was stirred overnight at room temperature and quenched by addition of H_2O (20 mL) after 18 hours. The phases were separated, and the aqueous phase was washed with CH_2Cl_2 (3 × 10 mL), and the organic phase washed with brine (20 mL) before being dried over $MgSO_4$. The crude product was concentrated *in vacuo* and purified by column chromatography (30-50% EtOAc in hexane) to give an off white amorphous solid (208 mg, 0.38 mmol, 43%). **^{1}H NMR** (400 MHz, $CDCl_3$) δ 7.86 (dd, J = 5.5, 3.1 Hz, 2H, Ar CH_{Phth}), 7.75 (dd, J = 5.4, 3.1 Hz, 2H, Ar CH_{Phth}), 7.48 (d, J = 7.6 Hz, 1H, N*H*),

7.28 – 7.13 (m, 3H, Ar C*H*$_{phenyl}$), 7.11 – 7.04 (m, 2H, Ar C*H*$_{phenyl}$), 6.65 (d, *J* = 8.0 Hz, 1H, N*H*), 4.82 – 4.73 (m, 1H, NHC*H*$_{Phe}$), 4.56 (q, *J* = 6.8 Hz, 1H, NHC*H*$_{Met}$), 4.43 (d, *J* = 10.9 Hz, 1H, NC*H*$_{Val}$), 3.63 (s, 3H, OCH_3), 3.10 (dd, *J* = 14.0, 5.6 Hz, 1H, CH_aH$_b$Ph), 3.03 (dd, *J* = 13.9, 6.7 Hz, 1H, CH$_a$$H_b$Ph), 2.89 – 2.75 (m, 1H, C*H*(CH_3)$_2$), 2.53 (t, *J* = 7.0 Hz, 2H, SCH_2), 2.10 – 1.88 (m, 5H, SCH_3 + SCH_2CH_2), 1.09 (d, *J* = 6.6 Hz, 3H, CHCH_3), 0.86 (d, *J* = 6.6 Hz, 3H, CHCH_3). **^{13}C NMR** (101 MHz, $CDCl_3$) δ 171.6 (*C*O_2), 170.4 (*C*(O)NH$_{Met}$), 168.6 (*C*(O)NH$_{Val}$), 168.5 (*C*(O)N*C*(O)), 135.7 (Ar *C*$_{phenyl}$), 134.6 (Ar *C*H$_{Phth}$), 131.6 (Ar *C*$_{Phth}$), 129.3 (Ar *C*H$_{phenyl}$), 128.8 (Ar *C*H$_{phenyl}$), 127.3 (Ar *C*H$_{phenyl}$), 123.1 (Ar *C*H$_{Phth}$), 62.4 (N*C*H$_{Val}$), 53.4 (NH*C*H$_{Phe}$), 52.5 (NH*C*H$_{Met}$), 52.4 (O*C*H_3), 37.8 (*C*H_2Ph), 30.8 (SCH_2*C*H_2), 30.1 (S*C*H_2), 27.9 (*C*H(CH_3)$_2$), 20.2 (CH(*C*H_3)), 19.6 (CH(*C*H_3)), 15.1 (SCH_3). **HRMS** $C_{28}H_{33}N_3O_6SNa$ $(M+Na)^+$ Calculated 562.1982, found 562.1996.

Cyanation Scope

General Method B: Cyanation of sulfides with 4-tosyl cyanide:

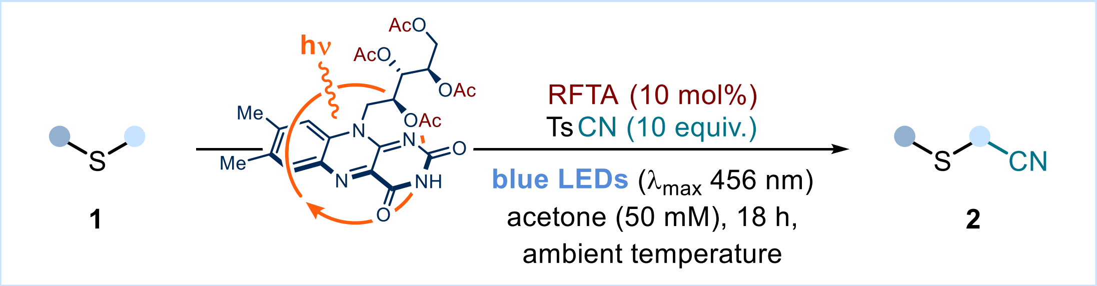

A 6 mL microwave vial with a magnetic stirrer bar was charged with riboflavin tetraacetate (5.4 mg, 0.01 mmol, 0.1 equiv.), 4-tosyl cyanide (181 mg, 1.0 mmol, 10 equiv.), and sulfide (0.1 mmol, 1 equiv.) and placed under a nitrogen atmosphere. The reagents were dissolved in degassed acetone (2 mL, 50 mM) and irradiated with blue LEDs (456 nm) for 18 h with fan cooling. After completion of the reaction, the reaction mixture was concentrated *in vacuo* and the crude residue was purified by column chromatography.

NMR Yield Procedure: The reaction was evaporated in a 35°C water bath, and dissolved in 1 mL of a 33 mM solution of $MeNO_2$ in $CDCl_3$. NMR yields were obtained by comparing the $MeNO_2$ peak at 4.3 ppm and the α-cyano peak, typically a dd falling between 3.3 – 4.0 ppm.

Typically, between 5.3 and 7.0 equivalents of unreacted TsCN were observed in the crude NMR, giving a recoverable yield of 59% and 78%.

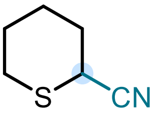

2a

Tetrahydrothiopyran-2-carbonitrile (2a)

Nitrile **2a** was prepared according to general method B using tetrahydrothiopyran (10 mg, 0.1 mmol, 1.0 equiv.) and 4-tosyl cyanide (181 mg, 1.0 mmol, 10.0 equiv.). The crude product mixture was analysed by ^{1}H NMR due to volatility of the product, indicating a yield of 66%. **^{1}H NMR** (400 MHz, $CDCl_3$) δ 3.55 (t, J = 4.0 Hz, 1H, SC*H*CN), 3.02 – 2.91 (m, 1H, SC$H_a$$H_b$), 2.60

– 2.49 (m, 1H, SCH$_a$*H$_b$*), 2.15 – 2.03 (m, 1H, SCH$_2$C*H$_a$*H$_b$), 1.98 – 1.86 (m, 2H, SCH$_2$CH$_2$C*H$_a$*H$_b$ + SCHCH$_a$*H$_b$*), 1.79 – 1.60 (m, 3H, SCH$_2$CH$_2$CH$_a$*H$_b$* + SCH$_2$C*H$_2$*).

The data was in accordance with the literature.[16]

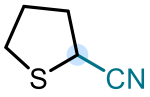

2b

Tetrahydrothiophene-2-carbonitrile (2b)

Nitrile **2b** was prepared according to general method B using tetrahydrothiophene (8.9 mg, 0.1 mmol, 1.0 equiv.) and 4-tosyl cyanide (181 mg, 1.0 mmol, 10.0 equiv.). The crude product mixture was analysed by ^{1}H NMR due to volatility of the product, indicating a yield of 84%. **^{1}H NMR** (500 MHz, CDCl$_3$) δ 3.97 (t, *J* = 5.1 Hz, 1H, SC*H*CN), 3.16 – 3.07 (m, 1H, SC*H$_a$*H$_b$), 2.95 (dt, *J* = 9.9, 7.0 Hz, 1H, SCH$_a$*H$_b$*), 2.25 – 2.12 (m, 3H, SCH(CN)C*H$_2$*). (Multiplet peak at 2.39-2.35 covered).

The data was in accordance with the literature.[17]

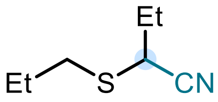

2c

2-(Propylthio)butanenitrile (2c)

Nitrile **2c** was prepared according to general method B using di-*n*-propyl sulfide (24 mg, 0.2 mmol, 1 equiv.) and 4-tosyl cyanide (362 mg, 2.0 mmol, 10.0 equiv.), and RFTA (10.8 mg, 0.02 mmol, 0.1 equiv.). The crude product mixture was analysed by ^{1}H NMR due to volatility of the product, indicating a yield of 51%. Column chromatography provided a sample of the product for characterisation (0-5% EtOAc in hexane) as an off white oil (11 mg, 0.076 mmol, 38%). **^{1}H NMR** (400 MHz, CDCl$_3$) δ 3.45 (dd, *J* = 7.7, 6.5 Hz, 1H, SC*H*CN), 2.82 – 2.64 (m, 2H, SC*H$_2$*), 1.99 – 1.79 (m, 2H, SCH(CN)C*H$_2$*), 1.75 – 1.59 (m, 2H, SCH$_2$C*H$_2$*), 1.15 (t, *J* = 7.4 Hz, 3H, SCH(CN)CH$_2$C*H$_3$*), 1.03 (t, *J* = 7.4 Hz, 3H, SCH$_2$CH$_2$C*H$_3$*). **^{13}C NMR** (101 MHz, CDCl$_3$)

δ 119.3 (*C*N), 34.3 (S*C*HCN), 33.9 (S*C*H_2), 26.2 (SCH(CN)*C*H_2), 22.4 (SCH_2*C*H_2), 13.4 (SCH(CN)CH_2*C*H_3), 11.8 (SCH_2CH_2*C*H_3). **v_{max}** (thin film/cm^{-1}): 2966, 2930, 2874, 2235, 1721. **HRMS** $C_7H_{13}NSNa$ (M+Na)$^+$ Calculated 166.0661, found 166.0658.

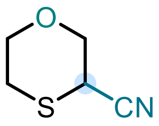

2d

1,4-Oxathiane-2-carbonitrile (2d)

Nitrile **2d** was prepared according to general method B using 1,4-oxathiane (9.4 μL, 0.1 mmol, 1.0 equiv.) and 4-tosyl cyanide (181 mg, 1.0 mmol, 10.0 equiv.). The crude product mixture was purified by column chromatography (5% EtOAc in toluene) to give a white solid (9.2 mg, 0.071 mmol, 71%). **^{1}H NMR** (400 MHz, $CDCl_3$) δ 4.22 (ddd, *J* = 11.9, 3.6, 0.8 Hz, 1H, SCH(CN)CH_aH_bO), 4.17 (dddd, *J* = 11.9, 3.8, 3.0, 0.7 Hz, 1H, OCH_aH_b), 3.93 (dd, *J* = 11.8, 2.4 Hz, 1H, SCH(CN)CH_aH_bO), 3.82 (ddd, *J* = 12.2, 10.2, 2.2 Hz, 1H, OCH_aH_b), 3.48 – 3.45 (m, 1H, SC*H*CN), 3.23 (dddd, *J* = 13.9, 10.3, 3.0, 0.5 Hz, 1H, SCH_aH_b), 2.52 (dddd, *J* = 14.1, 4.5, 2.2, 0.9 Hz, 1H, SCH_aH_b). **^{13}C NMR** (101 MHz, $CDCl_3$) δ 117.7 (*C*N), 69.7 (SCH(CN)*C*H_2O), 68.6 (SCH_2*C*H_2O), 26.9 (S*C*H(CN)), 25.4 (S*C*H_2). **v_{max}** (thin film/cm^{-1}): 2954, 2923, 2853, 2188, 1774, 1715, 1656, 1618, 1597, 1492. **HRMS** C_5H_8ONS (M+H)$^+$ Calculated 130.0321, found 130.0319.

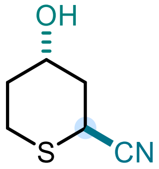

2e

***rac-2S,4S*-4-Hydroxytetrahydro-2H-thiopyran-2-carbonitrile (2e)**

Nitrile **2e** was prepared according to general method B using tetrahydrothiopyran-4-ol (12 mg, 0.1 mmol, 1.0 equiv.) and 4-tosyl cyanide (181 mg, 1.0 mmol, 10.0 equiv.). The crude product mixture was purified by column chromatography (50% EtOAc in hexane) to give an off-white

oil (7.0 mg, 0.05 mmol, 50%, dr >20:1). **^{1}H NMR** (400 MHz, $CDCl_3$) δ 4.00 (tt, *J* = 9.8, 3.6 Hz, 1H, C*H*OH), 3.89 (ddd, *J* = 5.3, 3.7, 1.4 Hz, 1H, SC*H*CN), 2.99 (ddd, *J* = 13.9, 10.8, 2.9 Hz, 1H, SCH_aH$_b$), 2.87 (dddd, *J* = 14.3, 5.3, 3.5, 1.5 Hz, 1H, SCH$_a$$H_b$), 2.41 (dddd, *J* = 13.2, 5.1, 3.6, 1.2 Hz, 1H, SCH(CN)CH_aH$_b$), 2.31 – 2.20 (m, 1H, SCH$_2$CH_aH$_b$), 1.93 (ddd, *J* = 13.4, 9.9, 3.8 Hz, 1H, SCH(CN)CH$_a$$H_b$), 1.79 – 1.71 (m, 1H, SCH$_2$CH$_a$$H_b$). **^{13}C NMR** (101 MHz, $CDCl_3$) δ 118.9 (*C*N), 65.8 (*C*HOH), 38.2 (SCH(CN)*C*H2), 35.4 (SCH$_2$*C*H$_2$), 27.7 (S*C*H(CN)), 25.72 (S*C*H$_2$). **v_{max}** (thin film/cm^{-1}): 3430, 2923, 2853, 2234, 1718, 1670. **HRMS** C_6H_9NSNa (M+Na)$^+$ Calculated 166.0297, found 166.0294.

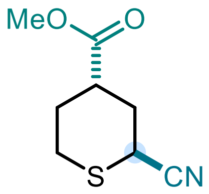

2f

rac-2S,4S-Methyl 2-cyanotetrahydrothiopyran-4-carboxylate (2f)

Nitrile **2f** was prepared according to general method B using methyl tetrahydrothiopyran-4-carboxylate (16 mg, 0.1 mmol, 1.0 equiv.) and 4-tosyl cyanide (181 mg, 1.0 mmol, 10.0 equiv.). The crude product mixture was purified by column chromatography (20% EtOAc in hexane) to give a clear oil (10.7 mg, 0.059 mmol, 59%, dr >20:1). **^{1}H NMR** (400 MHz, $CDCl_3$) δ 3.81 – 3.76 (m, 1H, SC*H*CN), 3.71 (s, 3H, OCH_3), 3.10 (ddd, *J* = 14.6, 12.3, 2.6 Hz, 1H, SCH_aH$_b$), 2.86 – 2.69 (m, 2H, SCH$_a$$H_b$ + C*H*CO$_2$Me), 2.51 – 2.44 (m, 1H, SCH(CN)CH_aH$_b$), 2.38 (dq, *J* = 13.9, 3.6 Hz, 1H, SCH$_2$CH_aH$_b$), 2.06 (ddd, *J* = 13.9, 12.2, 4.0 Hz, 1H, SCH(CN)CH$_a$$H_b$), 1.81 (dtd, *J* = 13.7, 12.1, 3.3 Hz, 1H, SCH$_2$CH$_a$$H_b$). **^{13}C NMR** (101 MHz, $CDCl_3$) δ 173.9 (*C*O$_2$CH$_3$), 118.6 (*C*N), 52.3 (O*C*H$_3$), 38.9 (*C*HCO$_2$CH$_3$), 32.0 (SCH(CN)*C*H$_2$), 29.2 (SCH$_2$*C*H$_2$), 27.8 (S*C*HCN), 26.2 (S*C*H$_2$). **v_{max}** (thin film/cm^{-1}): 2953, 2361, 2343, 1794, 1733. **HRMS** $C_8H_{11}O_2NS$ (M)$^+$ Calculated 185.0505, found 185.0505

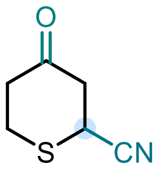

2g

2-Cyanotetrahydrothiopyran-4-one (2g)

Nitrile **2g** was prepared according to general method B using tetrahydrothiopyran-4-one (23 mg, 0.2 mmol, 1 equiv.) and 4-tosyl cyanide (362 mg, 2.0 mmol, 10.0 equiv.), and RFTA (10.8 mg, 0.02 mmol, 0.1 equiv.). The crude product mixture was purified by column chromatography (20-30% EtOAc in hexane) to give an off white oil (19 mg, 0.135 mmol, 67%). **^{1}H NMR** (500 MHz, $CDCl_3$) δ 4.08 (td, *J* = 5.0, 1.9 Hz, 1H, SC*H*CN), 3.38 (ddd, *J* = 14.5, 11.0, 3.6 Hz, 1H, SCH_aH$_b$), 3.07 (dtd, *J* = 14.4, 5.1, 1.9 Hz, 1H, SCH$_a$$H_b$), 2.98 – 2.82 (m, 3H, SCH(CN)CH_2 + SCH$_2$CH_aH$_b$), 2.72 (ddd, *J* = 15.0, 11.0, 5.2 Hz, 1H, SCH$_2$CH$_a$$H_b$). **^{13}C NMR** (126 MHz, $CDCl_3$) δ 202.6 (*C*=O), 117.3 (*C*N), 45.9 (SCH(CN)*C*H$_2$), 43.0 (SCH$_2$*C*H$_2$), 30.7 (S*C*HCN), 27.9 (S*C*H$_2$). **ν_{max}** (thin film/cm^{-1}): 2923, 2852, 2360, 2236, 1457, 1417, 1378. **HRMS** C_6H_8ONS $(M+H)^+$ Calculated 142.0321, found 142.0316.

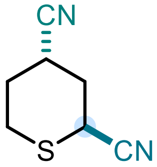

2h

***rac-2S,4S*-Tetrahydrothiopyran-2,4-dicarbonitrile (2h)**

Nitrile **2h** was prepared according to general method B using tetrahydrothiopyran-4-carbonitrile (25.4 mg, 0.2 mmol, 1.0 equiv.) and 4-tosyl cyanide (362 mg, 2.0 mmol, 10.0 equiv.), riboflavin tetraacetate (10.8 mg, 0.02 mmol, 0.1 equiv.) in acetone (2 mL). Crude product was purified by column chromatography (20% EtOAc in hexane) to give a pale yellow amorphous solid (13.2 mg, 0.087 mmol, 43%, dr >20:1). **^{1}H NMR** (400 MHz, $CDCl_3$) δ 3.86 – 3.76 (m, 1H, SC*H*CN), 3.10 – 2.96 (m, 2H, SCH_aH$_b$ + C*H*CN), 2.81 (dddd, *J* = 14.6, 4.8, 3.3,

1.2 Hz, 1H, SCH_a*H_b*), 2.57 – 2.47 (m, 1H, SCH(CN)C*H_a*H_b), 2.40 (ddt, *J* = 13.9, 5.6, 3.1 Hz, 1H, SCH_2C*H_a*H_b), 2.25 (ddd, *J* = 13.8, 11.3, 3.7 Hz, 1H, SCH(CN)CH_a*H_b*), 2.03 (dtd, *J* = 14.3, 11.2, 3.3 Hz, 1H, SCH_2CH_a*H_b*). **^{13}C NMR** (101 MHz, $CDCl_3$) δ 120.1 ($(CH_2)_2$CHCN), 117.5 (SCHCN), 32.9 (SCH*C*H_2), 29.7 (SCH_2*C*H_2), 27.1 (S*C*HCN), 25.5 ($(CH_2)_2$*C*HCN), 25.4 (S*C*H_2). **ν_{max}** (thin film/cm^{-1}): 2927, 2241, 1427, 1267. **HRMS** $C_7H_9N_2S$ $(M+H)^+$ Calculated 153.0481, found 153.0478.

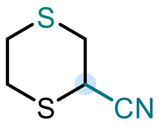

2i

1,4-Dithiane-2-carbonitrile (2i)

Nitrile **2i** was prepared according to general method B using 1,4-dithiane (24 mg, 0.2 mmol, 1.0 equiv.) and 4-tosyl cyanide (362 mg, 2.0 mmol, 10.0 equiv.), and riboflavin tetraacetate (10.8 mg, 0.02 mmol, 0.1 equiv.) in acetone (2 mL). The crude product mixture was purified by column chromatography (5-15% EtOAc in hexane) to give a white solid (13.2 mg, 0.091 mmol, 46%). **^{1}H NMR** (400 MHz, $CDCl_3$) δ 3.84 (dd, *J* = 5.5, 2.7 Hz, 1H, SC*H*CN), 3.43 – 3.31 (m, 1H, SC*H_a*H_b), 3.25 (dd, *J* = 14.1, 2.7 Hz, 1H, SCH(CN)C*H_a*H_b), 3.09 – 2.93 (m, 2H, SCH_2C*H_a*H_b + SCH(CN)CH_a*H_b*), 2.91 – 2.77 (m, 2H, SCH_a*H_b* + SCH_2CH_a*H_b*). **^{13}C NMR** (101 MHz, $CDCl_3$) δ 117.6 (*C*N), 31.6 (SCH(CN)*C*H_2), 28.5 (SCH_2*C*H_2), 28.2 (S*C*HCN), 27.5 (S*C*H_2). **ν_{max}** (thin film/cm^{-1}): 2911, 2853, 2231, 1743, 1407, 1369. **HRMS** $C_5H_8NS_2$ $(M+H)^+$ Calculated 146.0093, found 146.0093.

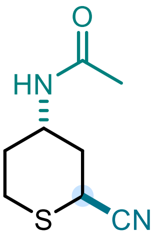

2j

***rac*-2*S*,4*S*-*N*-(2-Cyanotetrahydrothiopyran-4-yl)acetamide (2j)**

Nitrile **2j** was prepared according to general method B using *N*-(tetrahydro-2H-thiopyran-4-yl)acetamide (15.9 mg, 0.1 mmol, 1 equiv.) and 4-tosyl cyanide (181 mg, 1.0 mmol, 10.0 equiv.). The crude product mixture was purified by column chromatography (5% MeOH in CH_2Cl_2) to give a brown solid (11.7 mg, 0.065 mmol, 65%, dr >20:1). **^{1}H NMR** (400 MHz, $CDCl_3$) δ 5.41 (brs, 1H, N*H*), 4.14 (tdt, *J* = 11.6, 7.7, 3.8 Hz, 1H, C*H*NH), 3.79 (td, *J* = 4.0, 1.6 Hz, 1H, SC*H*CN), 3.19 (ddd, *J* = 14.7, 12.3, 2.5 Hz, 1H, SCH_aH_b), 2.75 (dddd, *J* = 14.5, 4.9, 3.6, 1.7 Hz, 1H, SCH_aH_b), 2.49 – 2.41 (m, 1H, SCH(CN)CH_aH_b), 2.41 – 2.31 (m, 1H, $SCH_2CH_aH_b$), 1.97 (s, 3H, C(O)CH_3), 1.79 (ddd, *J* = 13.2, 11.9, 4.1 Hz, 1H, SCH(CN)CH_aH_b), 1.65 – 1.51 (m, 1H, $SCH_2CH_aH_b$). **^{13}C NMR** (101 MHz, $CDCl_3$) δ 169.2 (*C*O), 118.3 (*C*N), 44.7 (*C*HN), 35.9 (SCH(CN)CH_2), 33.5 (SCH_2CH_2), 28.5 (S*C*H(CN)), 26.4 (SCH_2), 23.6 (C(O)CH_3). **ν_{max}** (thin film/cm^{-1}): 3330, 2936, 2360, 2235, 1776, 1746, 1645, 1529, 1434. **HRMS** $C_8H_{12}ON_2NaS$ $(M+Na)^+$ Calculated 207.0563, found 207.0569.

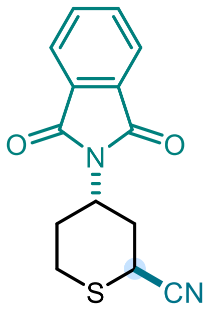

2k

***rac*-*2S,4S*-4-(1,3-Dioxoisoindolin-2-yl)tetrahydrothiopyran-2-carbonitrile (2k)**

Nitrile **2k** was prepared according to general procedure B using 2-(tetrahydrothiopyran-4-yl)isoindoline-1,3-dione (25 mg, 0.1 mmol, 1 equiv.) and 4-tosyl cyanide (181 mg, 1.0 mmol, 10 equiv.). The crude product mixture was purified by column chromatography (30% EtOAc in hexane) to give a white solid (15.8 mg, 0.058 mmol, 58%, dr >20:1). **^{1}H NMR** (400 MHz, $CDCl_3$) δ 7.85 (dd, *J* = 5.5, 3.1 Hz, 2H, Ar *H*), 7.79 – 7.69 (m, 2H, Ar *H*), 4.53 (tt, *J* = 12.5, 3.6 Hz, 1H, C*H*N), 3.90 (td, *J* = 3.9, 1.6 Hz, 1H, SC*H*CN), 3.26 (ddd, *J* = 15.0, 12.9, 2.5 Hz, 1H, SC$H_a$$H_b$), 2.98 (td, *J* = 12.9, 4.2 Hz, 1H, SCH(CN)C$H_a$$H_b$), 2.84 (dtd, *J* = 14.2, 3.7, 1.7 Hz, 1H, SC$H_a$$H_b$), 2.59 (qd, *J* = 12.8, 3.5 Hz, 1H, $SCH_2C$$H_a$$H_b$), 2.26 (dtd, *J* = 13.3, 3.5, 1.2 Hz, 1H, SCH(CN)C$H_a$$H_b$), 2.13 (dd, *J* = 13.2, 3.5 Hz, 1H, $SCH_2C$$H_a$$H_b$). **^{13}C NMR** (101 MHz, $CDCl_3$) δ 167.7 (*C*O), 134.3 (Ar *C*H), 131.7 (Ar *C*), 123.4 (Ar *C*H), 118.2 (*C*N), 46.0 (*C*HN), 32.9 (SCH(CN)CH_2), 30.7 (SCH_2CH_2), 29.1 (S*C*HCN), 26.9 (SCH_2). **ν_{max}** (thin film/cm^{-1}): 3057, 2926, 2362, 2235, 1775, 1614, 1468, 1449. **HRMS** $C_{14}H_{12}O_2N_2NaS$ $(M+Na)^+$ Calculated 295.0512, found 295.0520. **XRD** Single crystal analysis confirms the structure drawn, and the relative stereochemistry.

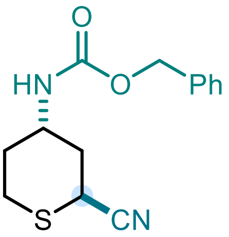

2l

***rac-2S,4S*-Benzyl (2-cyanotetrahydrothiopyran-4-yl)carbamate (2l)**

Nitrile **2l** was prepared according the general procedure B using benzyl (tetrahydrothiopyran-4-yl)carbamate (24.7 mg, 0.1 mmol, 1 equiv.) and 4-tosyl cyanide (181 mg, 1.0 mmol, 10 equiv.). The crude product mixture was purified by column chromatography (30% EtOAc in hexane) to give an amorphous white solid (12.9 mg, 0.047 mmol, 47%, dr >20:1). **^{1}H NMR** (400 MHz, $CDCl_3$) δ 7.41 – 7.29 (m, 5H, Ar *H*), 5.17 – 5.05 (m, 2H, $OC\mathit{H}_2Ph$), 4.71 – 4.66 (m, 1H, N*H*), 3.89 (ddd, *J* = 11.6, 7.7, 3.8 Hz, 1H, C*H*N), 3.78 (td, *J* = 3.9, 1.6 Hz, 1H, SC*H*CN), 3.16 (ddd, *J* = 14.7, 12.3, 2.5 Hz, 1H, $SC\mathit{H}_a H_b$), 2.81 – 2.70 (m, 1H, $SCH_a \mathit{H}_b$), 2.53 – 2.46 (m, 1H, $SCH(CN)C\mathit{H}_a H_b$), 2.39 – 2.31 (m, 1H, $SCH_2C\mathit{H}_a H_b$), 1.79 (td, *J* = 12.5, 3.9 Hz, 1H, $SCH(CN)CH_a \mathit{H}_b$), 1.67 – 1.52 (m, 3H, $SCH_2C\mathit{H}_a H_b$ + H_2O). **^{13}C NMR** (101 MHz, $CDCl_3$) δ 155.1 (*C*O), 136.2 (Ar *C*), 128.6 (Ar *C*H), 128.3 (2 × Ar *C*H), 118.2 (*C*N), 67.0 ($O\mathit{C}H_2Ph$), 46.2 (*C*HN), 36.1 ($SCH(CN)\mathit{C}H_2$), 33.6 ($SCH_2\mathit{C}H_2$), 28.4 (S*C*HCN), 26.3 ($S\mathit{C}H_2$). **ν_{max}** (thin film/cm^{-1}): 3326, 3033, 2944, 2231, 1696, 1527. **HRMS** $C_{14}H_{16}O_2N_2NaS$ $(M+Na)^+$ Calculated 299.0825, found 299.0832.

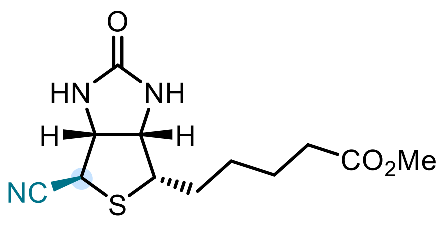

2m

Methyl 5-((3a*R*,4*R*,6*R*,6a*S*)-6-cyano-2-oxooctahydrocyclopenta[d]imidazol-4-yl) pentanoate (2m)

Biotin derivative **2m** was prepared according to general procedure B using biotin methyl ester (12.9 mg, 0.05 mmol, 1 equiv.) and 4-tosyl cyanide (90 mg, 0.5 mmol, 10.0 equiv.) and stirred for 24 hours. The crude product mixture was purified by column chromatography (7% MeOH in CH_2Cl_2) to give an off white solid (8.7 mg, 0.031 mmol, 62%, rr >50:1, dr >20:1). **^{1}H NMR** (400 MHz, MeOD) δ 4.74 (d, *J* = 7.6 Hz, 1H, SCH(CN)C*H*), 4.55 (dd, *J* = 7.7, 4.3 Hz, 1H, SCHC*H*), 4.04 (s, 1H, SC*H*CN), 3.69 – 3.61 (m, 4H, OCH_3 + SC*H*), 2.37 (t, *J* = 7.4 Hz, 2H, CH_2CO$_2$), 1.81 (ddt, *J* = 13.6, 10.0, 6.0 Hz, 1H, SCHCH_aH$_b$), 1.73 – 1.60 (m, 3H, SCHCH$_a$$H_b$ + CH_2), 1.55 – 1.42 (m, 2H, CH_2). **^{13}C NMR** (101 MHz, MeOD) δ 175.8 (*C*O$_2$), 165.2 (N*C*(O)N), 119.9 (*C*N), 66.3 (SCH(CN)*C*H), 63.2 (SCH*C*H), 57.0 (S*C*H), 52.1 (O*C*H$_3$), 43.2 (S*C*HCN), 34.4 (*C*H$_2$CO$_2$), 29.5 (*C*H$_2$), 28.9 (*C*H$_2$), 25.8 (*C*H$_2$). **ν_{max}** (thin film/cm^{-1}): 3369, 3246, 2931, 2360, 2342, 1708, 1595, 1456, 1437. $[\alpha]_D^{26}$ +4.85 ° (c = 2.8 g/100 ml, CH_2Cl_2). **HRMS** $C_{12}H_{18}O_3N_3S$ $(M+H)^+$ Calculated 284.1063, found 284.1056.

The proton alpha to the CN group appears as a singlet in the spectrum, indicating − by reference to the Karplus equation – that the proton has a dihedral angle with the adjacent proton of ~90°. This is only possible if the C8 centre has the *R* configuration.

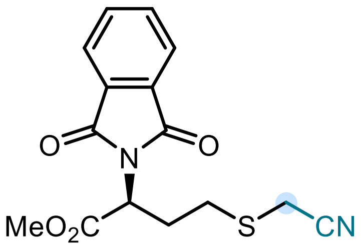

2na

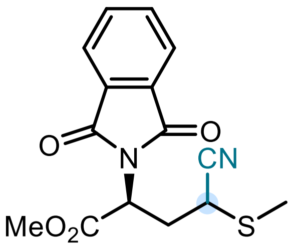

2nb
1.2:1 dr

Methyl (*S*)-4-((cyanomethyl)thio)-2-(1,3-dioxoisoindolin-2-yl)butanoate (2na) and methyl (2*S*)-4-cyano-2-(1,3-dioxoisoindolin-2-yl)-4-(methylthio)butanoate (2nb)

Methionine derivatives **2na** and **2nb** were prepared according to general procedure B using *N*-phthalimido-L-methionine methyl ester (29 mg, 0.1 mmol, 1.0 equiv.) and 4-tosyl cyanide (181 mg, 1.0 mmol, 10 equiv.). The crude product mixture was purified by column chromatography (30% EtOAc in hexane) to give off white oils (**2na**: 12.2 mg, 0.037 mmol, 37%; **2nb**: 9.5 mg, 0.029 mmol, 29%; total: 66%). The regioisomers were separated (**2na**:**2nb**, 1.3:1 regioisomeric ratio), and **2nb** was obtained in a 1.2:1 diastereoisomeric ratio.

2na:

^{1}H NMR (400 MHz, $CDCl_3$) δ 7.90 – 7.85 (m, 2H, Ar C*H*), 7.80 – 7.72 (m, 2H, Ar C*H*), 5.06 (dd, *J* = 9.3, 5.5 Hz, 1H, $CH_α$), 3.75 (s, 3H, OCH_3), 3.31 (s, 2H, SCH_2CN), 2.86 (ddd, *J* = 13.5, 7.4, 6.1 Hz, 1H, SC$H_a$$H_b$$CH_2$), 2.74 (dt, *J* = 13.5, 7.5 Hz, 1H, SC$H_a$$H_b$$CH_2$), 2.64 – 2.50 (m, 2H S$CH_2CH_2$). **^{13}C NMR** (101 MHz, $CDCl_3$) δ 169.2 (CO_2Me), 167.7 ($N(CO)_2$), 134.6 (Ar *C*H), 131.80 (Ar *C*), 123.9 (Ar C*H*), 116.2 (*C*N), 53.2 (OCH_3), 50.6 ($CH_α$), 29.3 (SCH_2), 28.1 (S$CH_2$$CH_2$), 17.0 (S$CH_2$CN). **$ν_{max}$** (thin film/cm^{-1}): 2955, 2361, 2244, 1775, 1743, 1712, 1612, 1468. $[\alpha]_D^{26}$ −114.7 ° (c = 3.4 g/100 ml, CH_2Cl_2). **HRMS** $C_{15}H_{14}N_2O_4S$ $(M)^+$ Calculated 341.0566, found 341.0581.

2nb:

^{1}H NMR (400 MHz, $CDCl_3$) δ 7.90 (dt, *J* = 5.4, 2.7 Hz, 5H, Ar *H* (3 H D1 + 2H D2)), 7.78 (dd, *J* = 5.5, 3.1 Hz, 5.25 H, Ar *H* (3H D1 + 2H D2)), 5.19 (ddd, *J* = 11.9, 9.3, 5.4 Hz, 2.48 H, C*H*N

(1.48 H D1 + 1 H D2)), 3.79 – 3.70 (m, 9.2 H, OCH_3 (4.2 H D1 + 3 H D2) + 1.4 H CHCN (D1)), 3.52 (dd, J = 8.4, 6.9 Hz, 1H, C*H*CN (D2)), 2.99 – 2.71 (m, 3.5H, SCH(CN)C*H₂* (1.5 H D1 + 2H D2), 2.62 – 2.48 (m, 1.8 H, SCH(CN)CH_2 (1.4 H D1)), 2.29 (s, 3H, SCH_3 (D2)), 2.28 (s, 4.2 H, SCH_3 (D1)). **^{13}C NMR** (101 MHz, $CDCl_3$) δ 168.6 (*C*O_2Me (D1)),168.6 (*C*O_2Me (D2)), 167.6 (*C*ON (D2)), 167.5 (*C*ON (D1)), 134.7 (Ar *C*H (D2)), 134.7 (Ar *C*H (D1)), 131.8 (Ar *C* (D1)), 131.8 (Ar *C* (D1)), 124.0 (Ar *C*H (D1 + D2)), 117.8 (*C*N (D2)), 117.8 (*C*N (D1)), 53.4 (O*C*H_3 (D2)), 53.4 (O*C*H_3 (D1)), 49.6 (*C*HN (D2)), 49.1 (*C*HN (D1)), 31.6 (SCH(CN)*C*H_2 (D1)), 31.4 (SCH(CN)*C*H_2 (D2), 31.4 (*C*HCN (D1 + D2)), 14.0 (S*C*H_3 (D1)), 13.8 (S*C*H_3 (D1)). **ν_{max}** (thin film/cm^{-1}): 2955, 2924, 2362, 1777, 1745, 1713, 1595, 1438. **HRMS** $C_{15}H_{14}N_2NaO_4S$ $(M+Na)^+$ Calculated 341.0566, found 341.0574.

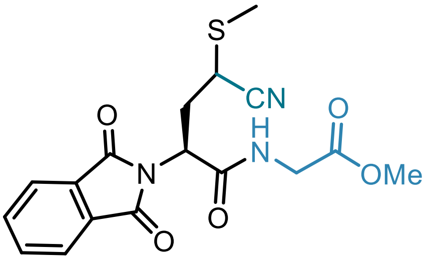

2oa

1:1.2 dr

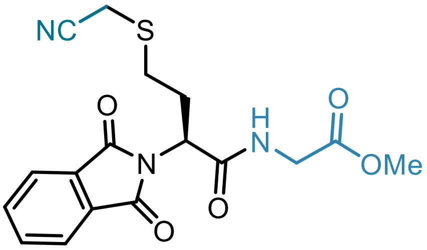

2ob

Methyl ((2*S*)-4-cyano-2-(1,3-dioxoisoindolin-2-yl)-4-(methylthio)butanoyl)glycinate (2oa) and methyl (*S*)-(4-((cyanomethyl)thio)-2-(1,3-dioxoisoindolin-2-yl)butanoyl)glycinate (2ob)

Peptide derivatives **2oa** and **2ob** were prepared according to general method B using methyl (*S*)-(2-(1,3-dioxoisoindolin-2-yl)-4-(methylthio)butanoyl)glycinate (35 mg, 0.1 mmol, 1.0 equiv.) and 4-tosyl cyanide (181 mg, 1.0 mmol, 10.0 equiv.). The crude product mixture was purified by column chromatography (60% EtOAc in hexane) to give off white oils (**2oa**: 12.5 mg, 0.033 mmol 33%; **2ob**: 11.6 mg, 0.31 mmol, 31%; total: 64%). The regioisomers were separated (2oa:2ob, 1:1.1 regioisomeric ratio), and **2oa** was obtained in a 1:1.2 ratio diastereoisomeric.

2oa:

^{1}H NMR (400 MHz, $CDCl_3$) δ 7.90 (ddd, *J* = 5.5, 3.0, 0.9 Hz, 4.4H, Ar C*H* (2.4H D1 + 2H D2)), 7.78 (dd, *J* = 5.5, 3.0 Hz, 4.9H, Ar C*H* (2.9H D1 + 2H D2)), 6.52 (dd, *J* = 10.3, 5.3 Hz, 2.2H, N*H* (1.2H D1 + 1H D2)), 5.19 (dd, *J* = 8.2, 6.6 Hz, 1.2H, C*H*N (D1)), 5.13 (t, *J* = 7.4 Hz, 1H, C*H*N (D2)), 4.03 (dt, *J* = 5.3, 2.7 Hz, 4.4H, CH_2N (2.4H D1 + 2H D2), 3.74 – 3.70 (m, 7.9H, SC*H*CN (1.3H D1) + OCH_3 (3.6 H D1 + 3H D2), 3.59 (t, *J* = 7.7 Hz, 1H, C*H*CN (D2)), 2.99 (dt, *J* = 14.6, 6.7 Hz, 1.2H, SCH(CN)CH_aH_b (D1)), 2.82 (t, *J* = 7.5 Hz, 2H, SCH(CN)CH_2 (D2)), 2.64 – 2.56 (m, 1.3H, SCH(CN)CH_aH_b), 2.28 (s, 2.7H, SCH_3 (D2)), 2.27 (s, 3.3H, SCH_3 (D1)). **^{13}C NMR** (101 MHz, $CDCl_3$) δ 169.9 (CO_2 (D1 + D2)), 167.8 (*C*ON (D2)), 167.7 (*C*ON (D1)), 167.7 (*C*(O)N*C*(O) (D1 + D2)), 134.9 (Ar *C*H (D2)), 134.9 (Ar *C*H (D1), 131.6 ((Ar *C* (D1)), 131.6 ((Ar *C* (D2)), 124.2 ((Ar *C*H (D2)), 124.1 ((Ar *C*H (D1)), 117.9 (*C*N (D2)), 117.8 (*C*N (D1)), 52.7 (OCH_3 (D1 + D2)), 51.6 (*C*HN (D2)), 50.8 (*C*HN (D1)), 41.6 (CH_2N (D2)), 41.6 (CH_2N (D2)), 31.5 (S*C*HCN (D1)), 31.5 (S*C*HCN (D2)), 31.2 (SCH(CN)CH_2 (D2)), 30.9 (SCH(CN)CH_2 (D1)), 14.0 (SCH_3 (D2)), 14.0 (SCH_3 (D2)). **v_{max}** (thin film/cm^{-1}): 3351, 2953, 2362, 2236, 1776, 1714, 1683. **HRMS** $C_{17}H_{18}O_5N_3S$ $(M+H)^+$ Calculated 376.0962, found 376.0950.

2ob:

^{1}H NMR (400 MHz, $CDCl_3$) δ 7.89 (td, *J* = 5.7, 2.8 Hz, 2H, Ar C*H*), 7.82 – 7.74 (m, 2H, Ar C*H*), 6.63 (s, 1H, N*H*), 5.07 (t, *J* = 7.7 Hz, 1H, C*H*N), 4.07 – 4.01 (dd, *J* = 9.6, 5.1 Hz, 2H, CH_2N), 3.73 (s, 3H, OCH_3), 3.39 – 3.25 (m, 2H, SCH_2CN), 2.91 – 2.71 (m, 2H, SCH_2), 2.64 (m, 2H, SCH_2CH_2). **^{13}C NMR** (101 MHz, $CDCl_3$) δ 170.0 (CO_2), 168.5 (*C*ON), 168.1 (*C*(O)N*C*(O)), 134.8 (Ar *C*H), 130.1 (Ar *C*), 127.3 (Ar *C*H), 116.6 (*C*N), 53.0 (*C*HN), 52.7 (OCH_3), 41.6 (CH_2N), 29.6 (SCH_2), 27.9 (SCH_2CH_2), 17.1 (SCH_2CN). **v_{max}** (thin film/cm^{-1}): 3370, 3058, 2954, 2361, 2249, 1774, 1713, 1685. **HRMS** $C_{17}H_{18}O_5N_3S$ $(M+H)^+$ Calculated 376.0962, found 376.0950.

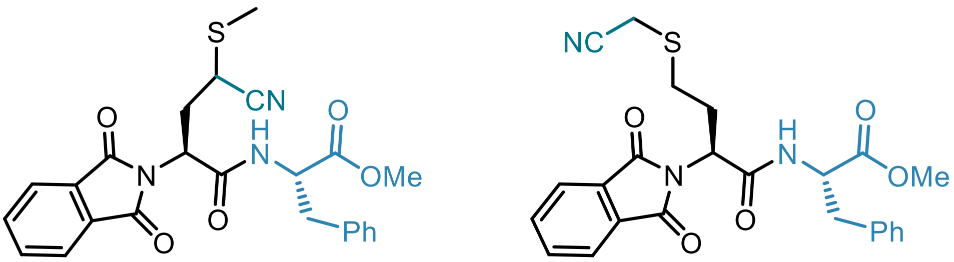

2pa

1:1.3 dr

2pb

Methyl ((2*S*)-4-cyano-2-(1,3-dioxoisoindolin-2-yl)-4-(methylthio)butanoyl) phenylalaninate (2pa) and methyl (*S*)-(4-((cyanomethyl)thio)-2-(1,3-dioxoisoindolin-2-yl)butanoyl)phenylalaninate (2pb)

Peptide derivatives **2pa** and **2pb** were prepared according to general method B using (*S*)-(2-(1,3-dioxoisoindolin-2-yl)-4-(methylthio)butanoyl)phenylalaninate (44 mg, 0.1 mmol, 1 equiv.) and 4-tosyl cyanide (181 mg, 1.0 mmol, 10.0 equiv.). The crude product mixture was purified by column chromatography (40-60% EtOAc in hexane) to give off white foams (**2pa**: 17.6 mg, 0.039 mmol, 39%; **2pb**: 16.9 mg, 0.036 mmol, 36%; total: 75%). The regioisomers were separated (**2pa**:**2pb**, 1.1:1 regioisomeric ratio), and **2pa** was obtained in a 1:1.3 diastereoisomeric ratio.

2pa:

^{1}H NMR (400 MHz, $CDCl_3$) δ 7.82 (dt, *J* = 5.0, 2.5 Hz, 5H, Ar CH_{Phth} (2.6H D1, 2H D2), 7.74 (dd, *J* = 5.5, 3.1 Hz, 4.6H, Ar CH_{Phth} (2.6 H D1 + 2 H D2)), 7.05 – 6.82 (m, 11H, Ar CH_{phenyl}, 5.6.5H D1 + 5H D2), 6.17 (d, *J* = 7.8 Hz, 2.3H, N*H* (1.3H D1 + 1H D2)), 5.02 (dd, *J* = 8.0, 6.9 Hz, 1.3H, NC*H* (D1)), 4.95 (dd, *J* = 8.2, 6.5 Hz, 1H, NC*H* (D2)), 4.78 (ddd, *J* = 8.3, 5.8, 2.5 Hz, 2.5H, NHC*H* (1.3H D1 +1H D2)), 3.68 – 3.58 (m, 9.9H, OCH_3 (D1 + D2) + C*H*CN (D1)), 3.43 (t, *J* = 7.7 Hz, 1H, C*H*CN (D2)), 3.07 – 2.93 (m, 4.6H, CH_2Ph (2.6H D1 + 2H D1)), 2.88 (dt, *J* = 14.5, 6.7 Hz, 1.3H, SCH(CN)CH_aH_b (D1)), 2.74 – 2.62 (m, 2H, SCH(CN)CH_2 (D2)), 2.49 – 2.33 (m, 1.4H, SCH(CN)CH_aH_b (D1)), 2.18 (s, 3H, SCH_3 (D2)), 2.16 (s, 3.6H, SCH_3 (D1)). **^{13}C NMR** (101 MHz, $CDCl_3$) δ 171.6 (CO_2 (D1)), 171.5 (CO_2CH_3 (D2)), 167.5 (*C*(O)N*C*(O) (D2)), 167.5 (*C*(O)N*C*(O) (D1)), 166.9 (*C*(O)N (D1)), 166.9 (*C*(O)N (D2)), 135.3 (Ar C_{phenyl} (D1 + D2)),

134.9 (Ar *C*H$_{Phth}$ (D2)), 134.8 (Ar *C*H$_{Phth}$ (D1)), 131.6 (Ar *C*$_{Phth}$ (D1)), 131.5 (Ar *C*$_{Phth}$ (D2)), 129.2 (Ar C*H*$_{phenyl}$), 128.6 (Ar C*H*$_{phenyl}$), 128.6 (Ar C*H*$_{phenyl}$ (D2)), 127.3 (Ar C*H*$_{phenyl}$ (D1)), 127.3 (Ar C*H*$_{phenyl}$ (D2)), 124.1 (Ar *C*H$_{Phth}$ (D2)), 124.1 (Ar *C*H$_{Phth}$ (D1)), 117.9 (*C*N (D1 + D2)), 53.3 (NH*C*H (D2)), 53.3 (NH*C*H (D1)), 52.7 (O*C*H$_3$ (D1 + D2)), 51.4 (N*C*H (D2)), 50.6 (N*C*H (D1)), 37.7 (*C*H$_2$Ph (D1)), 37.7 (*C*H$_2$Ph (D1)), 31.5 (*C*HCN (D1)), 31.4 (*C*HCN (D2)), 30.8 (SCH(CN)*C*H$_2$ (D2)), 30.6 (SCH(CN)*C*H$_2$ (D1)), 14.0 (S*C*H$_3$ (D1 + D2). **v$_{max}$** (thin film/cm^{-1}): 3352, 3030, 2952, 2361, 2342, 1776, 1717, 1686, 1611, 1498, 1381. **HRMS** $C_{24}H_{23}N_3O_5SNa$ (M+Na)$^+$ Calculated 488.1256, found 488.1265.

2pb:

^{1}H NMR (400 MHz, $CDCl_3$) δ 7.81 (dd, *J* = 5.5, 3.0 Hz, 2H, Ar C*H*$_{Phth}$), 7.72 (dt, *J* = 5.5, 2.9 Hz, 2H, Ar C*H*$_{Phth}$), 7.04 – 6.97 (m, 3H, Ar C*H*$_{phenyl}$), 6.93 (dd, *J* = 6.6, 2.9 Hz, 2H, Ar C*H*$_{phenyl}$), 6.31 (d, *J* = 7.8 Hz, 1H, N*H*), 4.88 (t, *J* = 7.6 Hz, 1H, NC*H*), 4.81 – 4.71 (m, 1H, NHC*H*), 3.65 (s, 3H, OC*H$_3$*), 3.28 – 3.13 (m, 2H, C*H$_2$*CN), 3.06 (dd, *J* = 13.9, 5.6, 1H, C*H$_a$*H$_b$Ph), 2.98 (dd, *J* = 13.8, 6.4 Hz, 1H, CH$_a$*H$_b$*Ph), 2.75 – 2.54 (m, 2H, SC*H$_2$*), 2.48 (q, *J* = 7.1 Hz, 2H, SCH$_2$C*H$_2$*). **^{13}C NMR** (101 MHz, $CDCl_3$) δ 171.7 (*C*O$_2$), 167.9 (*C*(O)N*C*(O)), 167.7 (*C*(O)N), 135.6 (Ar *C*$_{phenyl}$), 134.7 (Ar *C*H$_{Phth}$), 131.6 (Ar *C*$_{Phth}$), 129.3 (Ar *C*H$_{phenyl}$), 128.6 (Ar *C*H$_{phenyl}$), 127.2 (Ar *C*H$_{phenyl}$), 124.0 (Ar *C*H$_{Phth}$), 116.4 (*C*N), 53.4 (NH*C*H), 52.8 (N*C*H), 52.6 (O*C*H$_3$), 37.8 (*C*H$_2$Ph), 29.4 (S*C*H$_2$), 27.5 (SCH$_2$*C*H$_2$), 17.0 (S*C*H$_2$CN). **v$_{max}$** (thin film/cm^{-1}): 3367, 3057, 2954, 2360, 1776, 1716, 1686, 1611. **HRMS** $C_{24}H_{23}N_3O_5SNa$ (M+Na)$^+$ Calculated 488.1256, found 488.1267.

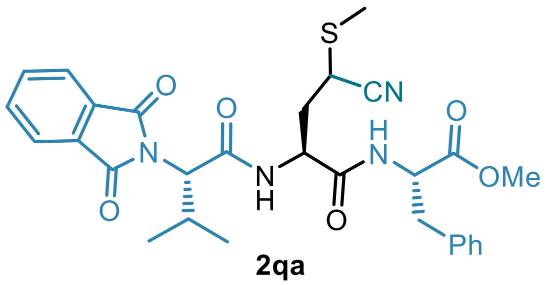

2qa

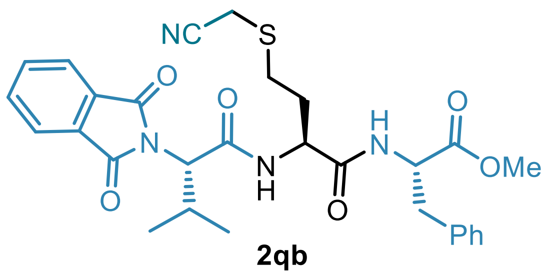

2qb

Methyl (4-cyano-2-((*S*)-2-(1,3-dioxoisoindolin-2-yl)-3-methylbutanamido)-4-(methylthio)butanoyl)-L-phenylalaninate (2qa) and methyl *S*-(cyanomethyl)-*N*-((*S*)-2-(1,3-dioxoisoindolin-2-yl)-3-methylbutanoyl)-L-homocysteinyl-L-phenylalaninate (2qb)

Peptide derivatives **2qa** and **2qb** were prepared according to general method B using methyl ((*S*)-2-(1,3-dioxoisoindolin-2-yl)-3-methylbutanoyl)-L-methionyl-L-phenylalaninate (54 mg, 0.1 mmol, 1.0 equiv.) and 4-tosyl cyanide (181 mg, 1.0 mmol, 10.0 equiv.). The crude product mixture was purified by column chromatography (30-50% EtOAc/hexane) to give off white oils (**2qa**: 22 mg, 0.039 mmol, 39%; **2qb**: 16.7 mg, 0.029 mmol, 29%; total: 68%). The regioisomers were separated (**2qa**:**2qb**, 1.3:1 regioisomeric ratio), and **2qa** was obtained in a 1:1.2 diastereoisomeric ratio.

2qa:

^{1}H NMR (400 MHz, $CDCl_3$) δ 7.87 (dq, *J* = 4.8, 2.5 Hz, 2H, Ar CH_{Phth} D1 + D2), 7.76 (ddd, *J* = 6.7, 3.6, 1.8 Hz, 2H, Ar CH_{Phth} (D1 + D2)), 7.46 (app. t, *J* = 7.7 Hz, 1H, N*H* (D1 + D2)), 7.28 – 7.16 (m, 3H, Ar CH_{phenyl} (D1 + D2)), 7.11 – 7.03 (m, 2H, Ar CH_{phenyl} (D1 + D2)), 6.60 (app. dd, *J* = 19.8, 7.9 Hz, 1H, N*H*), 4.76 (dddd, *J* = 11.7, 10.2, 8.2, 5.5 Hz, 1H, $NHCH_{Phe}$ (D1 + D2)), 4.64 (ddt, *J* = 21.5, 7.8, 6.3 Hz, 1H, $NHCH_{Met}$ (D1 + D2)), 4.44 (dd, *J* = 10.8, 4.7 Hz, 1H, NCH_{Val} (D1 + D2)), 3.80 – 3.59 (m, 5H, SC*H*CN + OCH_3 (D1 + D2)), 3.18 – 2.97 (m, 2H, CH_2Ph (D1 + D2)), 2.88 – 2.72 (m, 1H, C*H*$(CH_3)_2$ (D1 + D2)), 2.29 – 2.15 (m, 5H, SCH_3 + SCH(CN)CH_2), 1.08 (app. d, *J* = 6.6 Hz, 3H, CH_3 (D1 + D2)), 0.86 (app. dd, *J* = 6.6, 1.6 Hz, 3H, CH_3). **^{13}C NMR** (101 MHz, $CDCl_3$) δ 171.5 (CO_2 (D1)), 171.3 (CO_2 (D2)), 169.2 (*C*(O)NH (D1)), 169.0 (*C*(O)NH (D1)), 168.9 (*C*(O)NH (D2)), 168.6 (*C*(O)NH (D2)), 168.4 (*C*(O)N*C*(O) (D1)), 168.3(*C*(O)N*C*(O) (D2)), 135.5 (Ar C_{phenyl} (D2)), 135.2 (Ar C_{phenyl} (D1)), 134.6 (Ar CH_{Phth} (D2)),

134.5 (Ar *C*H_{Phth} (D1)), 131.3 (Ar *C*$_{Phth}$ (D1 + D2)), 129.1 (Ar CH_{phenyl} (D2)), 129.1 (Ar CH_{phenyl} (D1)), 128.9 (Ar CH_{phenyl} (D1)), 128.7 (Ar CH_{phenyl} (D2)), 127.4 (Ar CH_{phenyl} (D1)), 127.3 (Ar CH_{phenyl} (D2)), 123.9 (Ar *C*H_{Phth} (D1 + D2)), 118.3 (*C*N (D2)), 118.0 (*C*N (D1)), 62.1 (N*C*H_{Val} (D1)), 61.9 (N*C*H_{Val} (D2)), 53.4 (NH*C*H_{Phe} (D1)), 53.2 (NH*C*H_{Phe} (D2)), 52.6 (O*C*H_3 (D1)), 52.5 (O*C*H_3 (D2)), 51.2 (NH*C*H_{Met} (D2)), 50.5 (NH*C*H_{Met} (D1)), 37.5 (*C*H_2Ph (D2)), 37.3 (*C*H_2Ph (D1)), 34.4 (SCH(CN)*C*H_2 (D2)), 33.7 (SCH(CN)*C*H_2 (D1)), 30.9 (S*C*HCN (D2)), 30.1 (S*C*HCN (D1)), 27.8 (*C*H(CH_3)$_3$ (D2)), 27.7 (*C*H(CH_3)$_3$ (D1)), 20.2 (CH*C*H_3 (D2)), 20.1 (CH*C*H_3 (D1)), 19.4 (CH*C*H_3 (D1)), 19.4 (CH*C*H_3 (D2)), 13.6, (S*C*H_3 (D2)), 13.2 (S*C*H_3 (D1)). **v_{max}** (thin film/cm^{-1}): 3290, 2961, 2362, 2342, 1710, 1686, 1609, 1541. **HRMS** $C_{29}H_{32}N_4O_6SNa$ $(M+Na)^+$ Calculated 587.1935, found 587.1952.

2qb:

^{1}H NMR (400 MHz, $CDCl_3$) δ 7.87 (dd, *J* = 5.5, 3.0 Hz, 2H, Ar C*H*$_{Phth}$), 7.75 (dd, *J* = 5.5, 3.1 Hz, 2H, Ar C*H*$_{Phth}$), 7.40 (d, *J* = 7.6 Hz, 1H, N*H*), 7.27 – 7.13 (m, 3H, Ar C*H*$_{phenyl}$), 7.09 – 7.02 (m, 2H, Ar C*H*$_{phenyl}$), 6.49 (d, *J* = 7.9 Hz, 1H, N*H*), 4.77 (ddd, *J* = 7.9, 6.6, 5.6 Hz, 1H, NHC*H*$_{Phe}$), 4.57 (q, *J* = 6.8 Hz, 1H, NHC*H*$_{Met}$), 4.43 (d, *J* = 10.8 Hz, 1H, NC*H*$_{Val}$), 3.66 (s, 3H, OC*H*$_3$), 3.35 – 3.20 (m, 2H, SC*H*$_2$CN), 3.09 (dd, *J* = 14.0, 5.6 Hz, 1H, C*H*$_a$H$_b$Ph), 3.03 (dd, *J* = 14.0, 6.7 Hz, 1H, CH$_a$*H*$_b$Ph), 2.90 – 2.70 (m, 3H, SC*H*$_2$ + C*H*(CH_3)$_2$), 2.16 – 2.08 (m, 1H, SCH_2C*H*$_a$H$_b$), 2.03 – 1.95 (m, 1H, SCH_2CH$_a$*H*$_b$), 1.08 (d, *J* = 6.7 Hz, 3H, CH(C*H*$_3$)), 0.85 (d, *J* = 6.6 Hz, 3H, CH(C*H*$_3$)). **^{13}C NMR** (101 MHz, $CDCl_3$) δ 171.7 (*C*O_2), 170.0 (*C*(O)NH), 168.9 (*C*(O)NH), 168.5 (*C*(O)N*C*(O)), 135.6 (Ar *C*$_{phenyl}$), 134.6 (Ar *C*H_{Phth}), 131.5 (Ar *C*$_{Phth}$), 129.3 (Ar *C*H_{phenyl}), 128.9 (Ar *C*H_{phenyl}), 127.4 (Ar *C*H_{phenyl}), 124.0 (Ar *C*H_{Phth}), 116.6 (*C*N), 62.4 (N*C*H_{Val}), 53.4 (NH*C*H_{Phe}), 52.6 (O*C*H_3), 51.9 (NH*C*H_{Met}), 37.6 (*C*H_2Ph), 31.1 (SCH_2*C*H_2), 28.6 (S*C*H_2), 28.0 (*C*H(CH_3)$_2$), 20.2 (CH*C*H_3), 19.6 (CH*C*H_3), 16.7 (S*C*H_2CN). **v_{max}** (thin film/cm^{-1}): 3307, 3064, 2964, 2360, 1713, 1656, 1527. **HRMS** $C_{29}H_{32}N_4O_6SNa$ $(M+Na)^+$ Calculated 587.1935, found 587.1952.

Amino Acid Tolerance Experiments

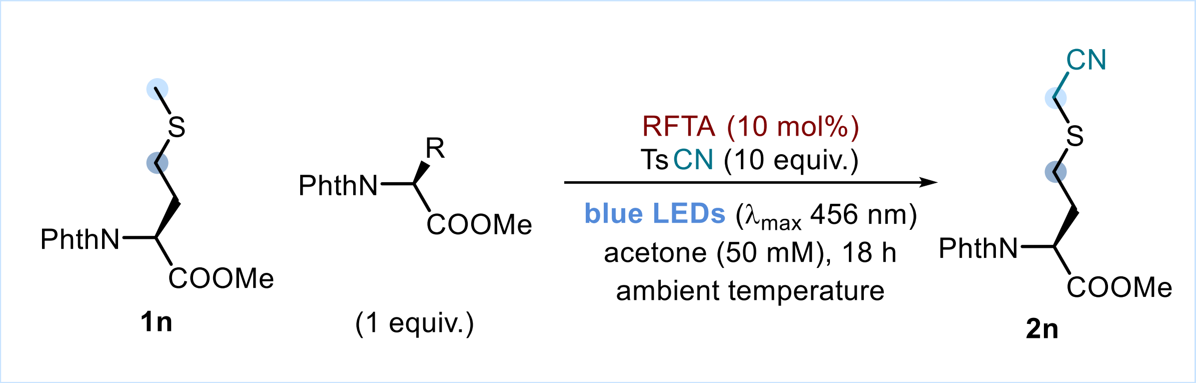

General Procedure:

A 6 mL microwave vial with a magnetic stirrer bar was charged with riboflavin tetraacetate (2.7 mg, 0.005 mmol, 0.1 equiv.), 4-tosyl cyanide (91 mg, 0.5 mmol, 10 equiv.), methyl (*S*)-2-(1,3-dioxoisoindolin-2-yl)-4-(methylthio)butanoate (**1n**, 14.6 mg, 0.05 mmol, 1 equiv.), and 1 equivalent of amino acid additive (0.05 mmol, 1 equiv.) and placed under a nitrogen atmosphere. The reagents were dissolved in degassed acetone (1 mL, 50 mM) and irradiated with blue LEDs (456 nm) for 18 h with fan cooling. After completion of the reaction, the reaction mixture was concentrated *in vacuo* and analysed by ^{1}H NMR.

NMR Yield Procedure: The reaction was evaporated in a 35°C water bath before the addition of 0.5 mL of a 33 mM solution of $MeNO_2$ in $CDCl_3$, with up to 0.5 mL $CDCl_3$ used to solubilise any additional undissolved solid. NMR yields were obtained by comparing the $MeNO_2$ peak at 4.3 ppm and the peak for the proton alpha to the amide group in the methionine products. If the signals of the additive obscured or overlapped with the peak for the proton alpha to the amide group in methionine product, the peak for the proton alpha to the cyano group in the methionine product was instead used for calculating an NMR yield. Peaks used to assign NMR yield are highlighted on the spectrum and reaction scheme.

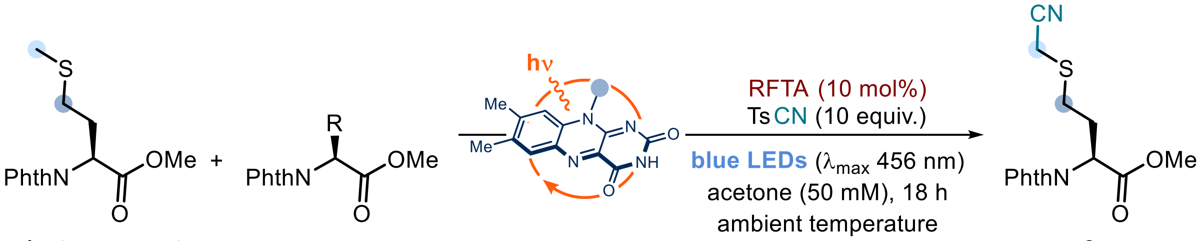

1n (0.05 mmol) 1.0 equiv. **2n**

Additive:

none

68%

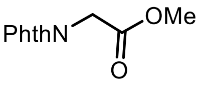

Phth-Gly-OMe
71%

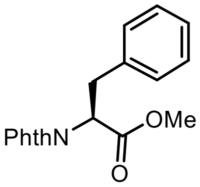

Phth-Phe-OMe
65%

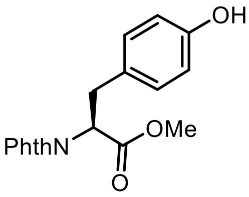

Phth-Tyr-OMe
62%

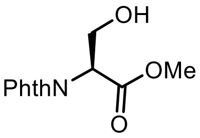

Phth-Ser-OMe
65%

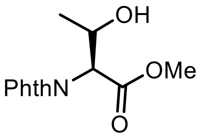

Phth-Thr-OMe
69%

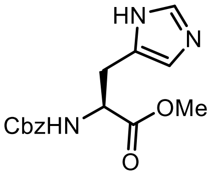

Cbz-His-OMe
73%

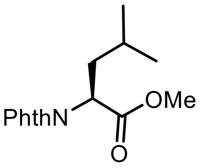

Phth-Leu-OMe
75%

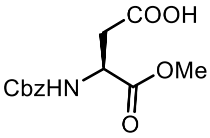

Cbz-Asp-OMe
60%

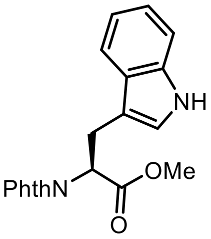

Phth-Trp-OMe
49%

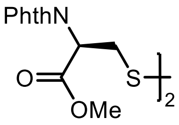

Phth-Cys-OMe dimer
63%

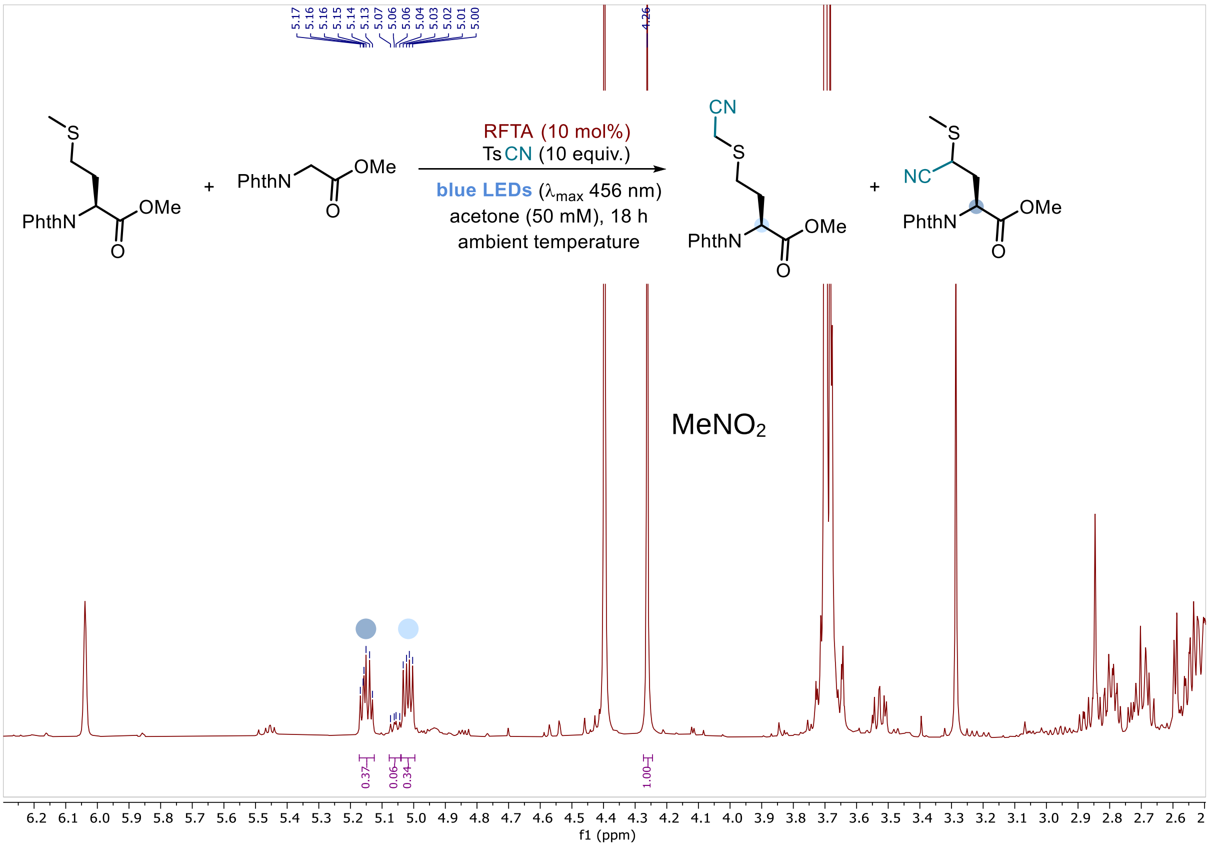
RFTA (10 mol%)
TsCN (10 equiv.)
blue LEDs (λmax 456 nm)
acetone (50 mM), 18 h
ambient temperature
PhthN
OMe
MeNO2
f1 (ppm)

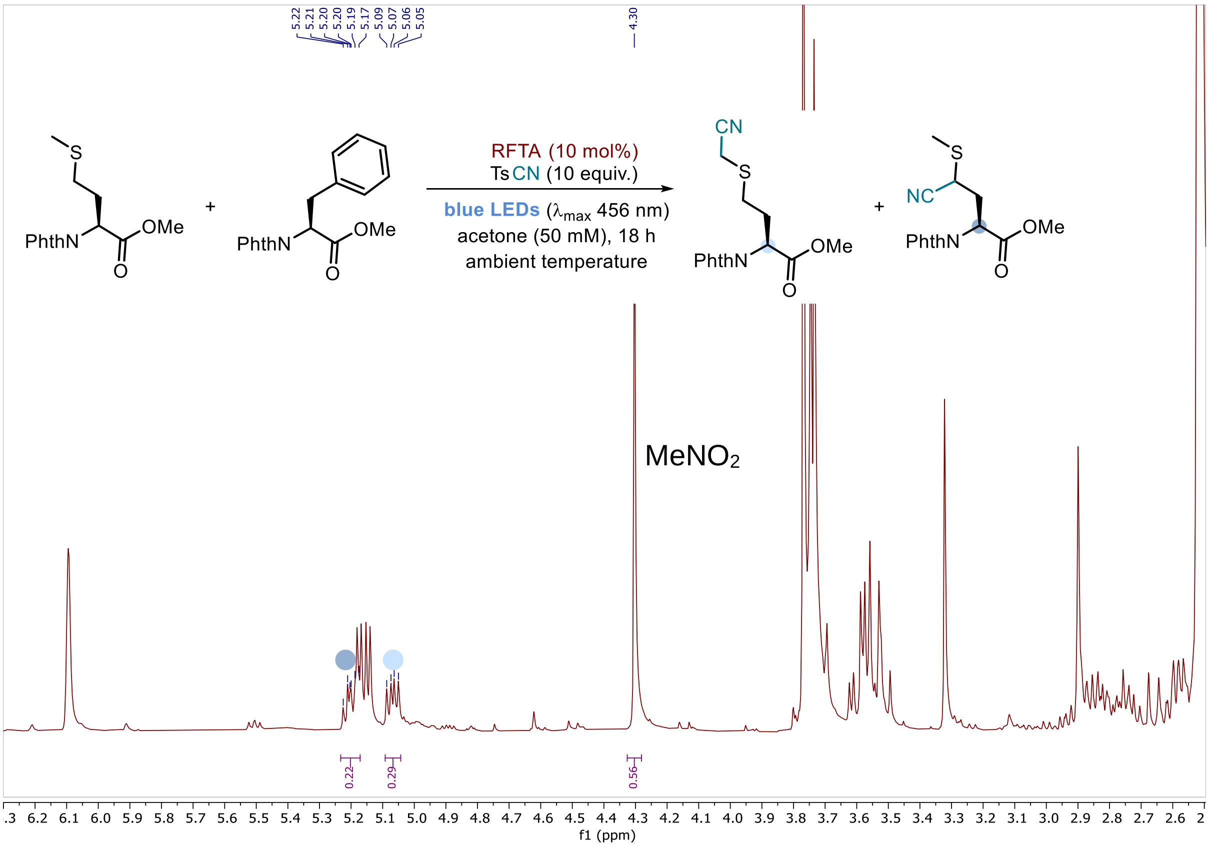
RFTA (10 mol%)
TsCN (10 equiv.)
blue LEDs (λmax 456 nm)
acetone (50 mM), 18 h
ambient temperature
PhthN
OMe
MeNO2
f1 (ppm)

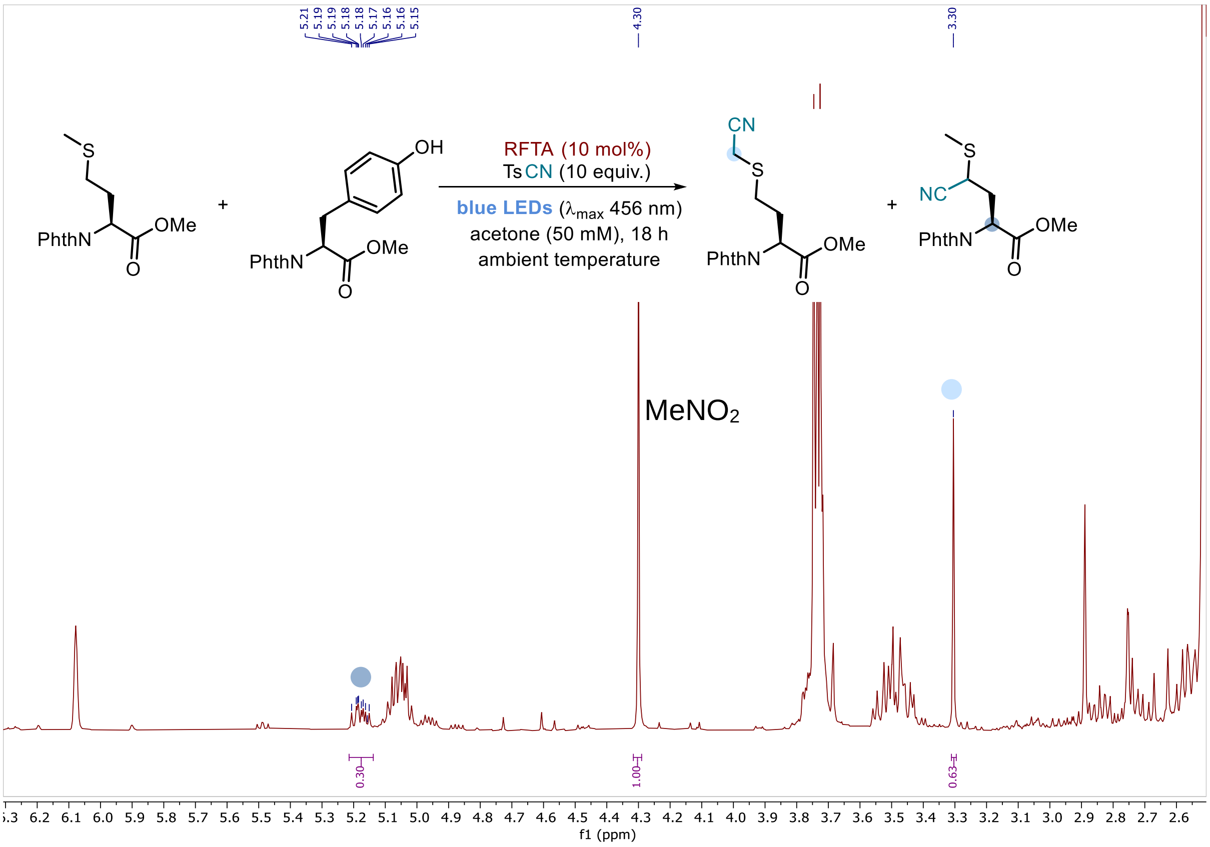
RFTA (10 mol%)
TsCN (10 equiv.)
blue LEDs (λ_{max} 456 nm)
acetone (50 mM), 18 h
ambient temperature
PhthN
OMe
OH
CN
NC
MeNO$_2$
0.30
1.00
0.63
f1 (ppm)

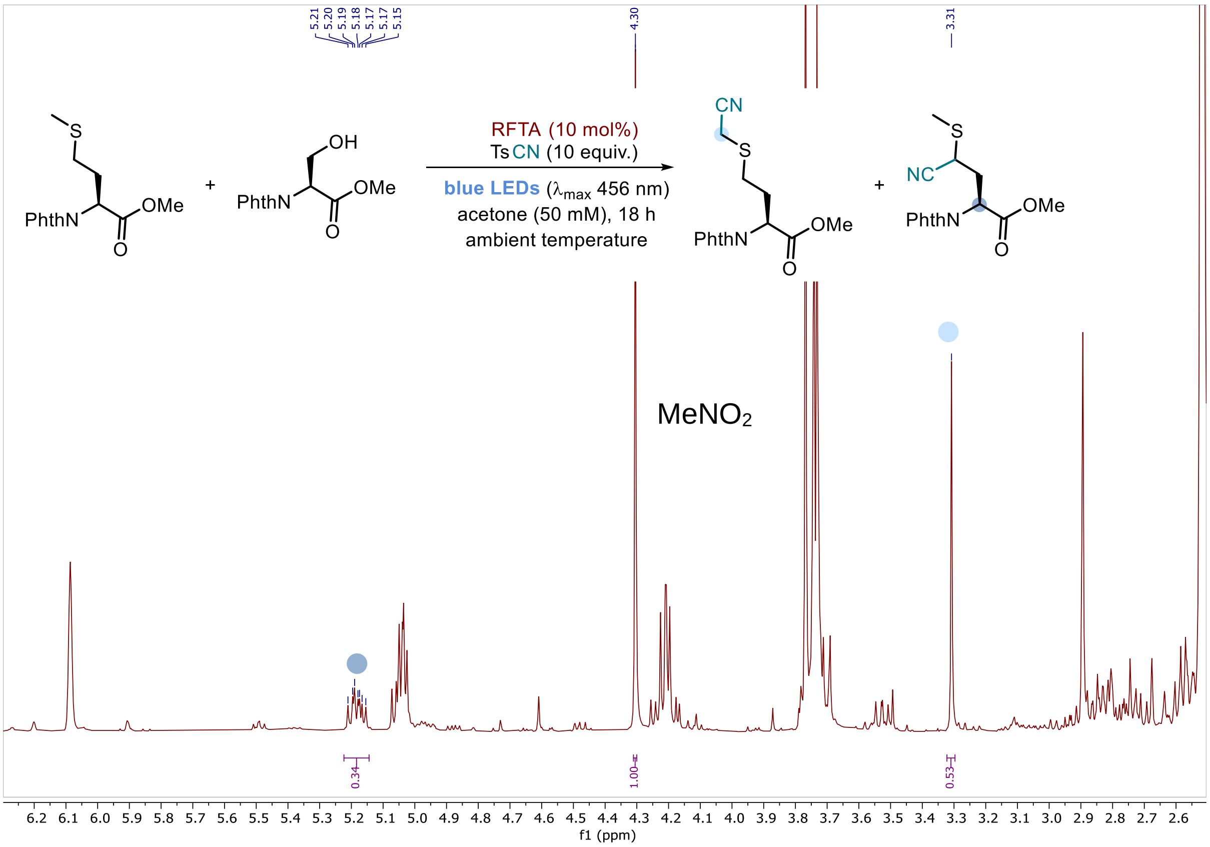
RFTA (10 mol%)
TsCN (10 equiv.)
blue LEDs (λ_{max} 456 nm)
acetone (50 mM), 18 h
ambient temperature
PhthN
OMe
OH
CN
NC
MeNO$_2$
0.34
1.00
0.53
f1 (ppm)

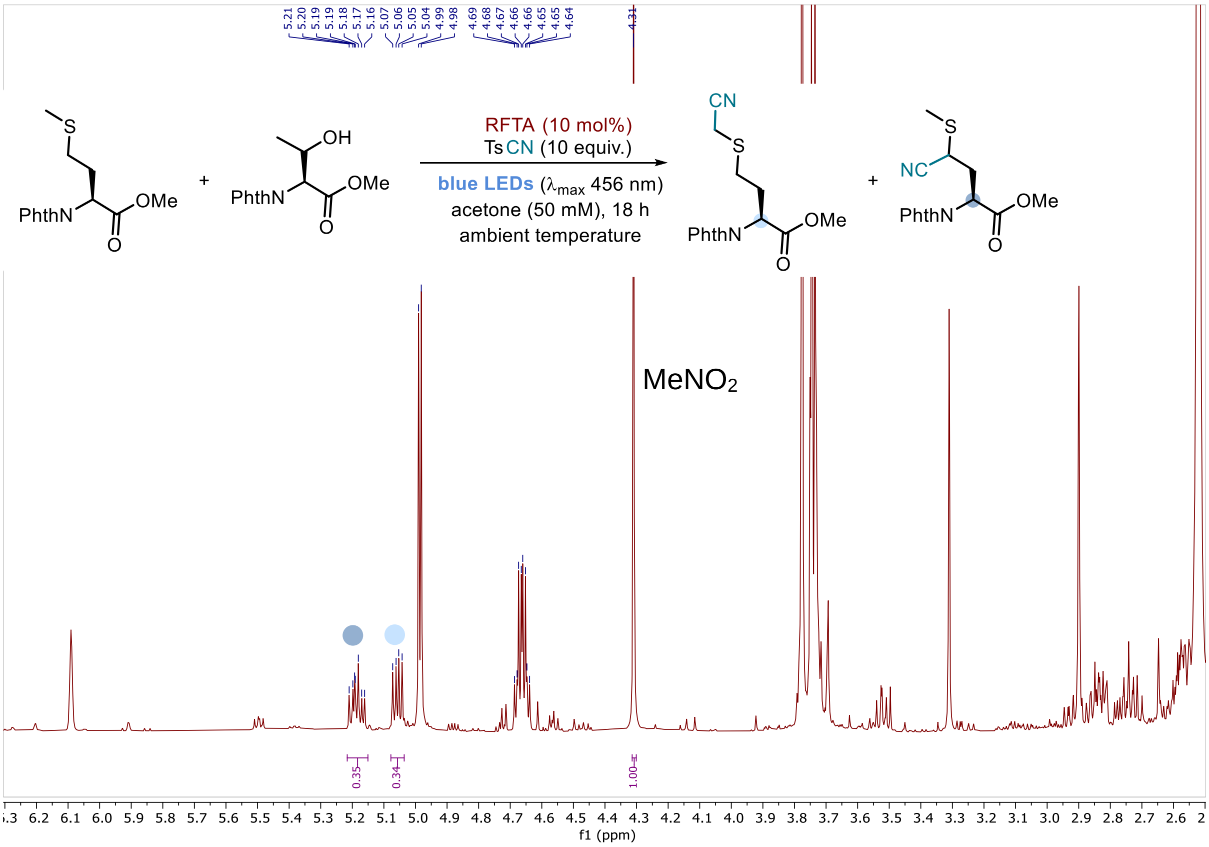
RFTA (10 mol%)
TsCN (10 equiv.)
blue LEDs (λmax 456 nm)
acetone (50 mM), 18 h
ambient temperature
PhthN
OMe
OH
MeNO2
f1 (ppm)

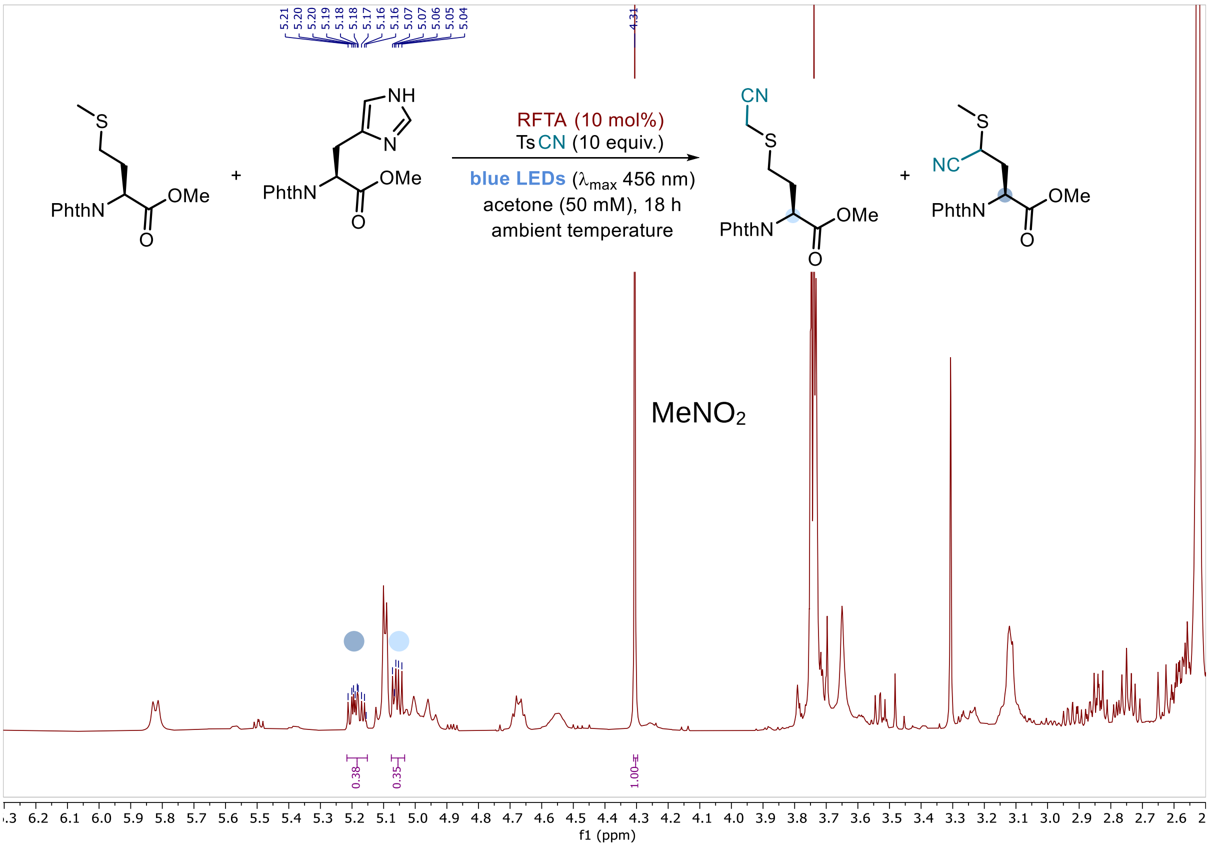
RFTA (10 mol%)
TsCN (10 equiv.)
blue LEDs (λmax 456 nm)
acetone (50 mM), 18 h
ambient temperature
PhthN
OMe
NH
MeNO2
f1 (ppm)

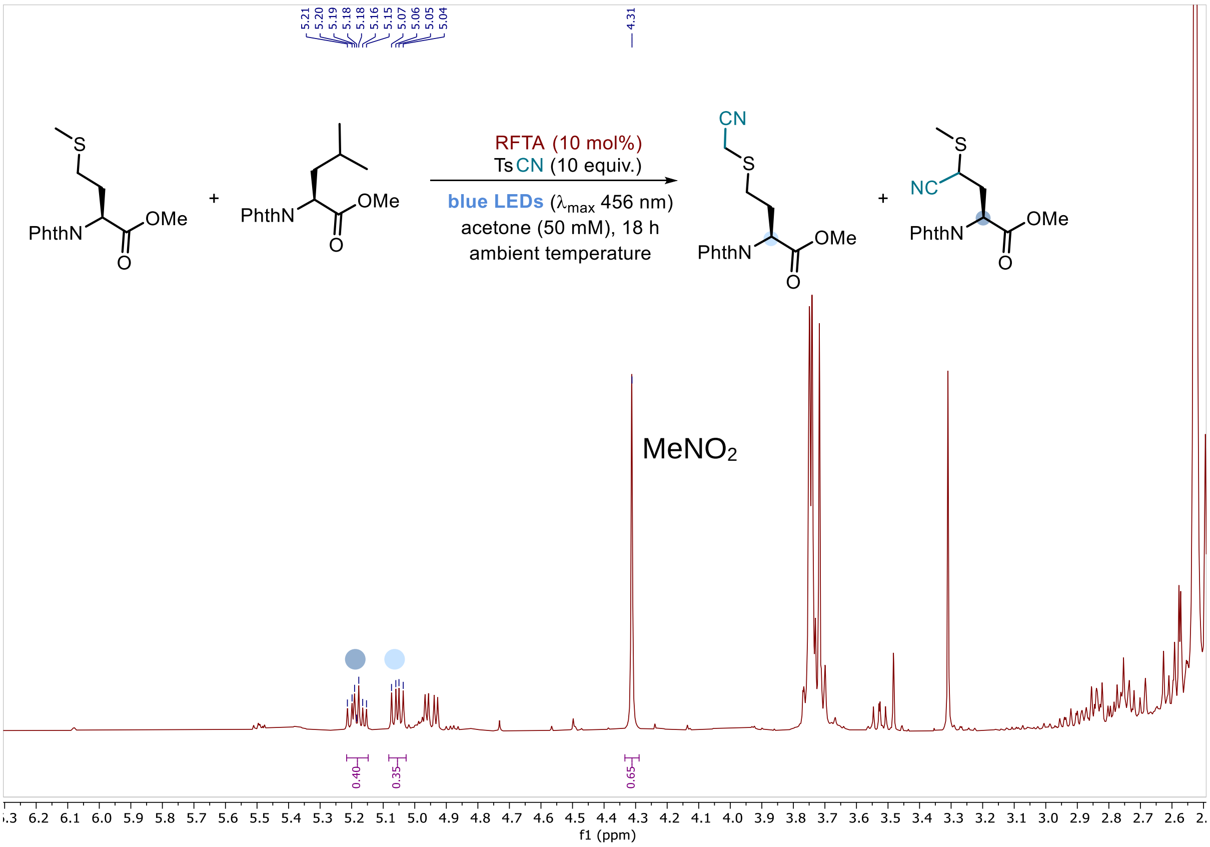
RFTA (10 mol%)
TsCN (10 equiv.)
blue LEDs (λmax 456 nm)
acetone (50 mM), 18 h
ambient temperature
PhthN
OMe
PhthN
OMe
CN
NC
MeNO2
0.40
0.35
0.65
f1 (ppm)

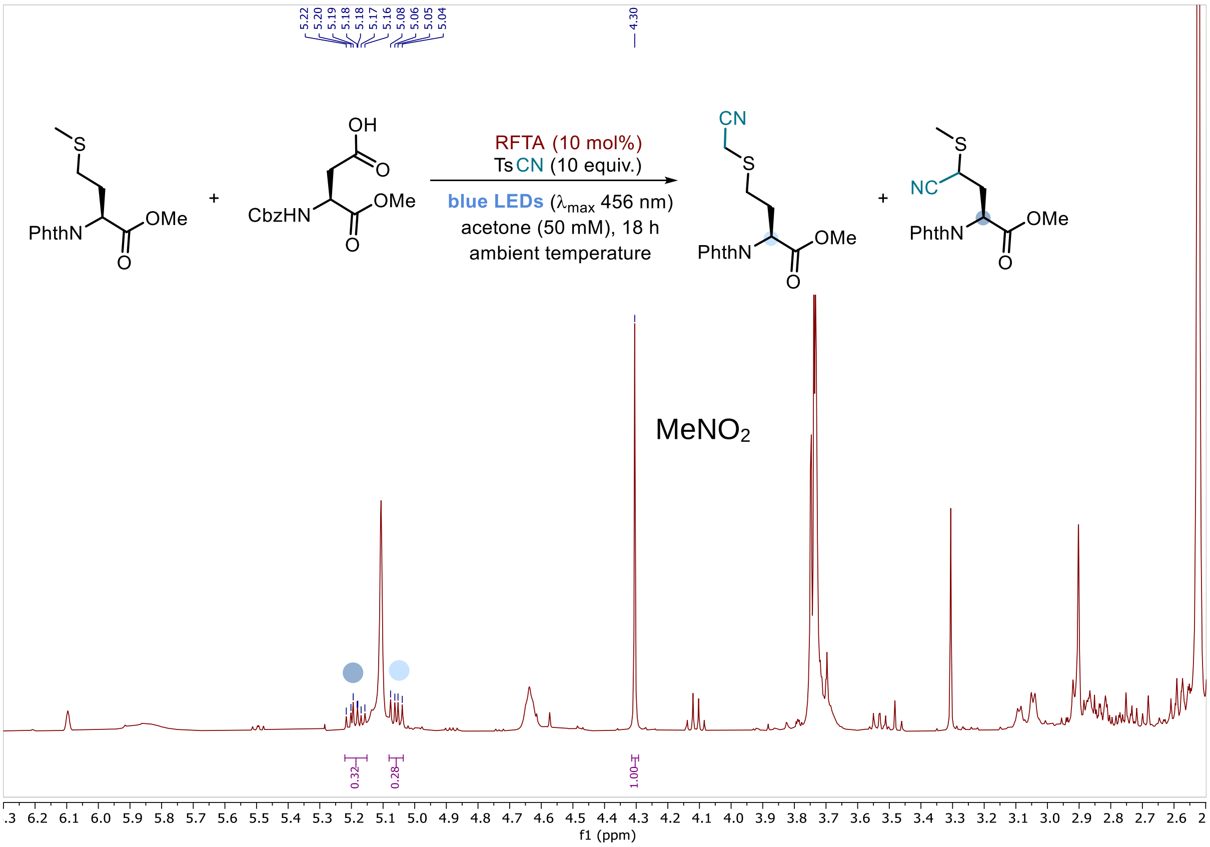
RFTA (10 mol%)
TsCN (10 equiv.)
blue LEDs (λmax 456 nm)
acetone (50 mM), 18 h
ambient temperature
PhthN
CbzHN
OH
OMe
CN
NC
MeNO2
0.32
0.28
1.00
f1 (ppm)

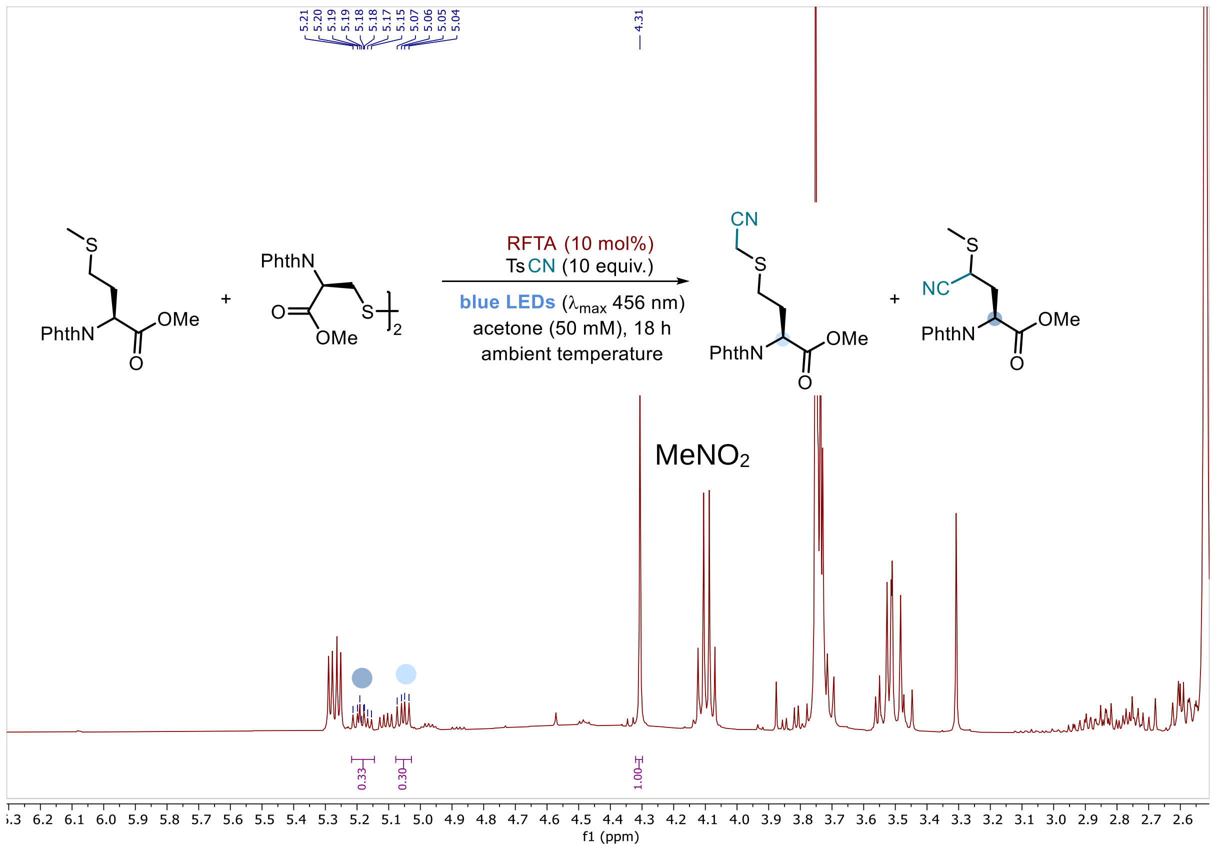
RFTA (10 mol%)
TsCN (10 equiv.)
blue LEDs (λ_{max} 456 nm)
acetone (50 mM), 18 h
ambient temperature
MeNO2
f1 (ppm)

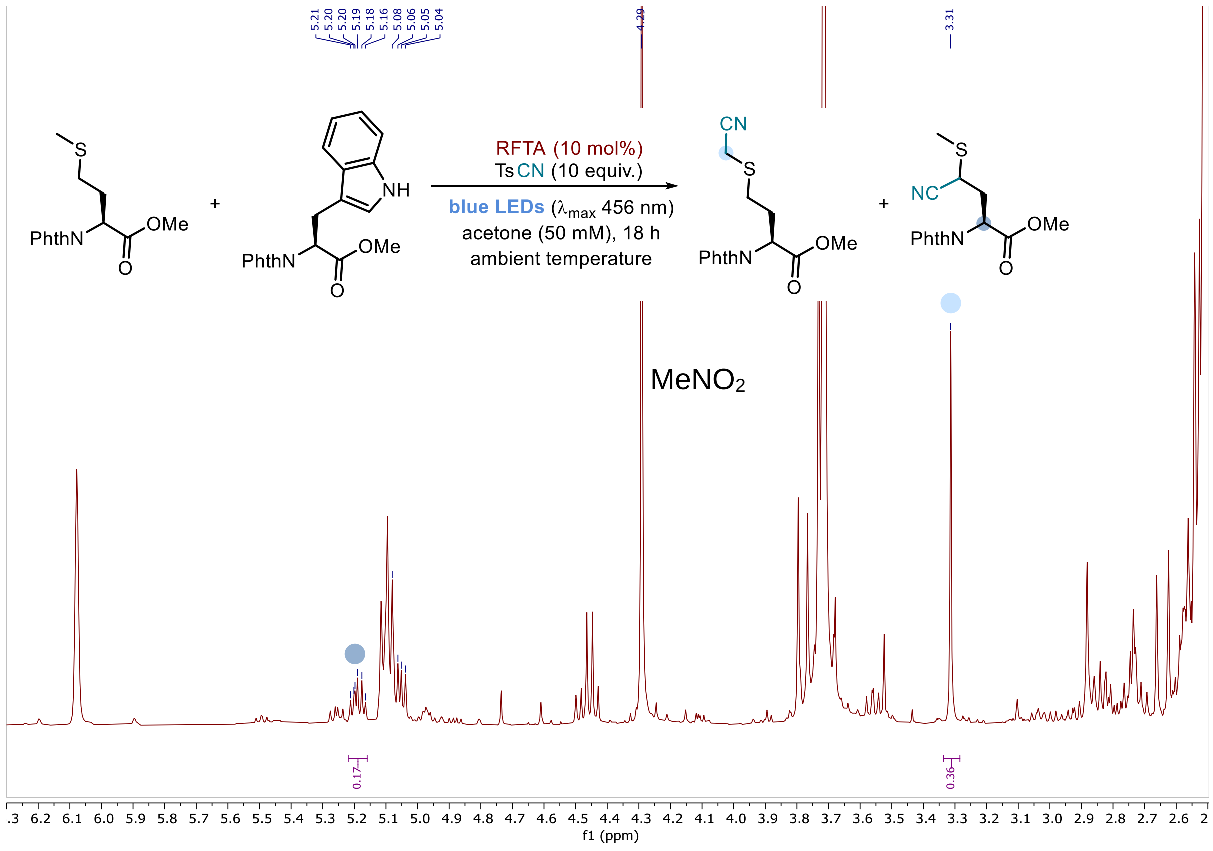
RFTA (10 mol%)
TsCN (10 equiv.)
blue LEDs (λ_{max} 456 nm)
acetone (50 mM), 18 h
ambient temperature
MeNO2
f1 (ppm)

Possible Origin of Diastereoselectivity

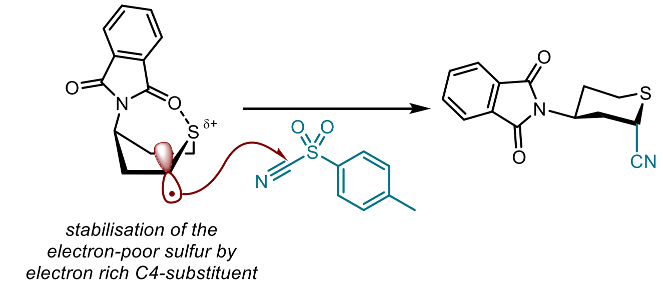

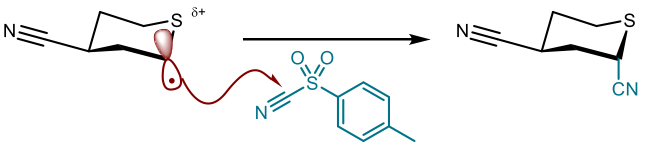

The excellent diastereoselectivity observed when using 4-substituted thiopyrans likely arises during the radical addition step. The sulfur atom in the α-sulfur radical is electron-deficient, and a transannular stabilization of the partial charge on sulfur may occur in a boat conformation with the electron-rich C4-substituent interacting with sulfur. The interaction would block the top face of the molecule, leading to radical addition anti to the C4 substituent.

For C4-substituents that are unable to interact with the electron-poor S atom, it is believed that the C4-substituent adopts an equatorial orientation, with radical addition anti to the C4 substituent.

X-Ray Crystallography Data

All data collection, crystal structure determination, and refinement was completed by the X-ray crystallography service (Dr George Whitehead) at The University of Manchester.

Data collection

X-ray data was collected at a temperature of 100K on a Rigaku FR-X DW rotating anode diffractometer using CuKα radiation, (λ = 1.54184 Å) with an AFC-11 RINC goniometer and a Rigaku Hypix 6000 HE photon counting detector. The diffractometer was equipped with an Oxford Cryosystems Cryostream 800 plus nitrogen flow gas system.

Crystal structure determinations and refinements

X-ray data were processed and reduced using CrysAlisPro suite of programs. The crystal structures were solved and refined against all F2 values using the SHELX and Olex 2 suite of programs. All the non-hydrogen atoms were refined anisotropically. Hydrogen atoms were placed in a calculated position refined using idealised geometries (riding model) and assigned fixed isotropic displacement parameters. Some carbon atoms were found disordered and modelled over two positions were possible. In such cases, C-C bond distances were restrained using FIX and SADI commands. The atomic displacement parameters (adp) of the disordered atoms have been restrained using RIGU command.

Data availability

Crystallographic data for **2k** has been deposited in the Cambridge Crystallographic Data Centre, with deposition number CCDC 2305964, and are available free of charge via www.ccdc.camac.uk/data_request/cif.

***rac*-2*S*,4*S*-4-(1,3-Dioxoisoindolin-2-yl)tetrahydrothiopyran-2-carbonitrile** (**2k**)

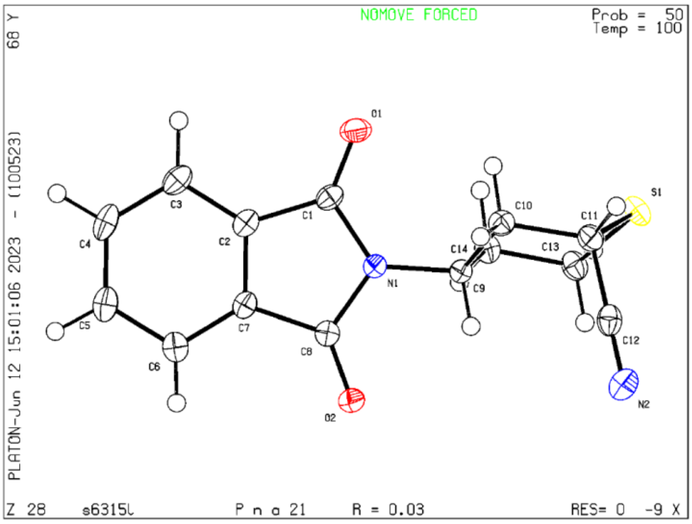

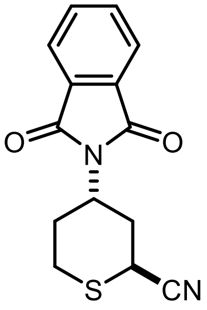

2k

Table S1: Crystal data and structure refinement for **2k**.

Empirical Formula	$C_{14}H_{12}N_2O_2S$
Temperature / K	100
Crystal System	orthorhombic
Space Group	P n a 21
a / Å	7.1005(1)
b / Å	16.6816(2)
c / Å	10.7309(2)
α / °	90
β / °	90
γ / °	90
Volume / Å^3	1271.05(3)
Z	4
F(000)	568
Radiation	CuKα
2Θ Range for Data Collection / °	9.800 to 152.664
Index Ranges	$-8 \leq h \leq 8$, $-20 \leq k \leq 19$, $-13 \leq l \leq 12$

Goodness-of-fit on F2	1.058
Final R Indexes [I>=2σ (I)]	R1 = 0.0259 wR2 = 0.0660
Final R Indexes [all data]	R1 = 0.0262 wR2 = 0.0662
Absolute Structure Parameter	−0.008(7)

Alkenylation Scope

4.2.7 Alkenylation Scope

General Method C:

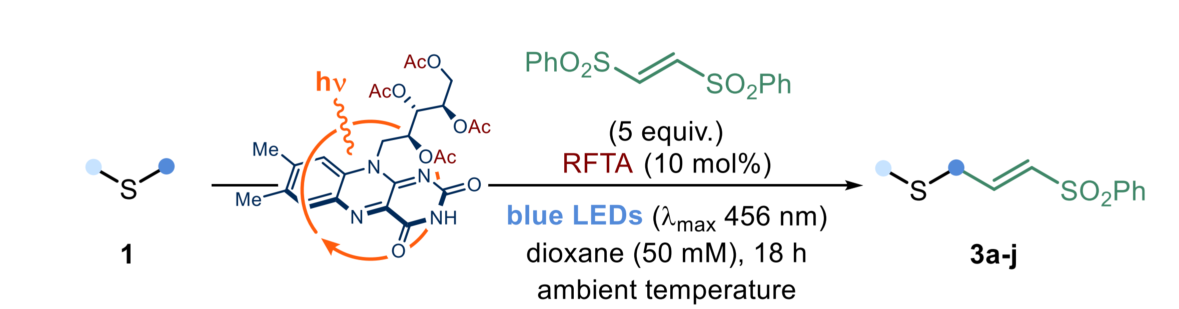

A 6 mL microwave vial with a magnetic stirrer bar was charged with riboflavin tetraacetate (5.4 mg, 10 mol%), trans-1,2-bis(phenylsulfonyl)ethylene (154 mg, 0.5 mmol). and sulfide (0.1 mmol, 1 equiv.) and placed under a nitrogen atmosphere. The reagents were dissolved in degassed 1,4-dioxane (2 mL, 50 mM) and irradiated with blue LEDs (456 nm) for 18 h with fan cooling. After completion of the reaction, the crude product mixture was concentrated *in vacuo* and purified by column chromatography.

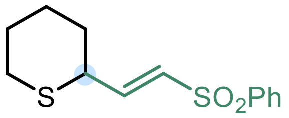

3a

(*E*)-2-(2-(Phenylsulfonyl)vinyl)tetrahydro-2*H*-thiopyran (3a)

Sulfone **3a** was prepared according to general method C, using tetrahydrothiopyran (10.1 μL, 0.1 mmol) and *trans*-1,2-bis(phenylsulfonyl)ethylene (154 mg, 0.5 mmol). The crude product

mixture was purified using column chromatography (20% EtOAc in hexane) to give an off-white oil (13.6 mg, 0.051 mmol, 51%). **^{1}H NMR** (400 MHz, $CDCl_3$) δ 7.91 – 7.85 (m, 2H, Ar C*H*), 7.65 – 7.59 (m, 1H, Ar C*H*), 7.58 – 7.51 (m, 2H, Ar C*H*), 6.98 (dd, *J* = 15.0, 6.6 Hz, 1H, C*H*=CHSO$_2$Ph), 6.48 (dd, *J* = 15.0, 1.5 Hz, 1H, CH=C*H*SO$_2$Ph), 3.57 – 3.44 (m, 1H, SC*H*), 2.71 – 2.59 (m, 2H, SC*H$_2$*), 2.13 – 2.07 (m, 1H, SCHCH$_a$*H$_b$*), 1.95 – 1.73, m, 2H, SCH$_2$CH$_a$*H$_b$* + SCH$_2$CH$_2$CH$_a$*H$_b$*), 1.74 – 1.70 (m, 2H, SCHCH$_a$*H$_b$* + SCH$_2$C*H$_a$*H$_b$), 1.51 – 1.38 (m, 1H, SCH$_2$CH$_2$C*H$_a$*H$_b$). **^{13}C NMR** (101 MHz, $CDCl_3$) δ 145.7 (CH=*C*HSO$_2$Ph), 140.2 (Ar *C*), 133.4 (Ar *C*H), 131.3 (*C*H=CHSO$_2$Ph), 129.3 (Ar *C*H), 127.7 (Ar *C*H), 41.4 (S*C*H), 32.3 (SCH*C*H$_2$), 28.4 (S*C*H$_2$), 26.4 (SCH$_2$*C*H$_2$), 24.5 (SCH$_2$CH$_2$*C*H$_2$). **ν_{max}** (thin film/cm^{-1}): 2924, 1597, 1490, 1443, 1406, 1265, 1008, 757, 664, 554, 519. **HRMS** $C_{13}H_{17}O_2S_2$ $(M+H)^+$ Calculated 269.0664, found 269.0662.

The data are in accordance with the literature.[18]

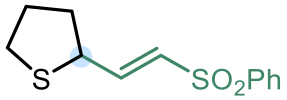

3b

(*E*)-2-(2-(Phenylsulfonyl)vinyl)tetrahydrothiophene (3b)

Sulfone **3b** was prepared according to general method C, using tetrahydrothiophene (8.8 µL, 0.1 mmol) and *trans*-1,2-bis(phenylsulfonyl)ethylene (154 mg, 0.5 mmol). The crude product mixture was purified using column chromatography (5% EtOAc in toluene) to give an off-white oil (15.4 mg, 0.061 mmol, 61%). **^{1}H NMR** (400 MHz, $CDCl_3$) δ 7.93 – 7.84 (m, 2H, ArC*H*), 7.68 – 7.59 (m, 1H, ArC*H*), 7.59 – 7.50 (m, 2H, ArC*H*), 6.91 (dd, *J* = 14.8, 8.6 Hz, 1H, C*H*=CHSO$_2$Ph), 6.40 (dd, J = 14.8, 0.9 Hz, 1H, CH=C*H*SO$_2$Ph), 4.05 – 3.96 (m, 1H, SC*H*), 3.01 – 2.93 (m, 1H, SCH$_a$*H$_b$*), 2.93 – 2.85 (m, 1H, SC*H$_a$*H$_b$), 2.25 – 2.13 (m, 1H, SCHCH$_a$*H$_b$*), 2.13 –1.94 (m, 2H, SCH$_2$C*H$_2$*), 1.89 – 1.78 (m, 1H, SCHC*H$_a$*H$_b$). **^{13}C NMR** (101 MHz, $CDCl_3$) δ 146.7 (*C*H=CHSO$_2$Ph), 140.3 (Ar *C*), 133.3 (Ar *C*H), 129.7 (CH=*C*HSO$_2$Ph), 129.3 (Ar *C*H), 127.6 (Ar *C*H), 47.7 (S*C*H), 36.8 (SCH*C*H$_2$), 33.2 (S*C*H$_2$), 30.4 (SCH$_2$*C*H$_2$). **ν_{max}** (thin film/cm$^-$

[1]): 2275, 2207, 2152, 1598, 585, 498, 422. **HRMS** $C_{12}H_{15}S_2O_2$ $(M+H)^+$ Calculated 255.0508, found 255.0506.

The data are in accordance with the literature.[18]

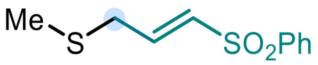

3c

(*E*)-Methyl(3-(phenylsulfonyl)allyl)sulfane (3c)

Sulfone **3c** was prepared according to general method C, using dimethyl sulfide (7.4 μL, 0.1 mmol) and *trans*-1,2-bis(phenylsulfonyl)ethylene (155 mg, 0.5 mmol). The crude product mixture was purified using column chromatography (20% EtOAc in hexane) to give a colourless oil (9.6 mg, 0.042 mmol, 42%). **^{1}H NMR** (400 MHz, $CDCl_3$) δ 7.93 – 7.88 (m, 2H, Ar C*H*), 7.67 – 7.61 (m, 1H, Ar C*H*), 7.59 – 7.53 (m, 2H, Ar C*H*), 6.91 (dd, *J* = 14.7, 7.3 Hz, 1H, SO_2CH=C*H*), 6.43 (dt, *J* = 14.9, 1.3 Hz, 1H, SO_2C*H*=CH), 3.21 (dd, *J* = 7.1, 1.4 Hz, 2H, SC*H*$_2$CH), 1.98 (s, 3H, C*H*$_3$S). **^{13}C NMR** (101 MHz, $CDCl_3$) δ 141.3 (SO_2CH=*C*H), 133.5 (Ar *C*H), 132.2 (Ar *C*H), 129.5 (Ar *C*H), 129.4 (Ar *C*), 127.7 (SO_2*C*H=CH), 34.0 (S*C*H$_2$), 14.9 (S*C*H$_3$). **ν_{max}** (thin film/cm^{-1}): 2915, 1309, 1145, 756, 654, 598, 551, 528, 484, 457, 445, 431. **HRMS** $C_{10}H_{13}S_2O_2$ $(M+H)^+$ Calculated 229.0351, found 229.0343.

The data are in accordance with the literature. [19]

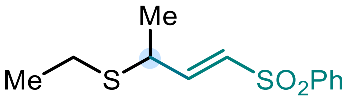

3d

(*E*)-Ethyl(4-(phenylsulfonyl)but-3-en-2-yl)sulfane (3d)

Sulfone **3d** was prepared according to general method C, using diethyl sulfide (10.8 μL, 0.1 mmol) and *trans*-1,2-bis(phenylsulfonyl)ethylene (155 mg, 0.5 mmol). The crude product mixture was purified using column chromatography (5% EtOAc in toluene) to give an off-white oil (8.3 mg, 0.033 mmol, 33%). **^{1}H NMR** (400 MHz, $CDCl_3$) δ 7.95 – 7.98 (m, 2H, Ar C*H*), 7.66 – 7.59 (m, 1H, Ar C*H*), 7.57 – 7.50 (m, 2H, Ar C*H*), 6.84 (dd, *J* = 14.9, 8.6 Hz, 1H, SCHC*H*=CH), 6.30 (dd, *J* = 14.9, 0.9 Hz, 1H, SCHCH=C*H*), 3.50 – 3.38 (m, 1H, SC*H*), 2.36

(q, J = 7.4 Hz, 2H, SCH_2CH$_3$), 1.37 (d, J = 6.9 Hz, 3H, SCHCH_3), 1.15 (t, J = 7.4 Hz, 3H, SCH$_2$CH_3). **^{13}C NMR** (101 MHz, CDCl$_3$) δ 146.9 (SCHCH=CH), 140.6 (Ar *C*), 133.4 (Ar *C*H), 129.3 (SCHCH=*C*H), 129.2 (Ar *C*H), 127.6 (Ar *C*H), 39.8 (S*C*H), 24.7 (S*C*H$_2$CH$_3$), 19.2 (SCH*C*H$_3$), 14.4 (SCH$_2$*C*H$_3$). **v_{max}** (thin film/cm^{-1}): 2198, 2036, 2010, 1144, 528, 484, 456, 429. **HRMS** $C_{12}H_{17}S_2O_2$ $(M+H)^+$ Calculated 257.0664, found 257.0669.

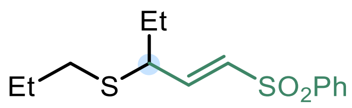

3e

(*E*)-(1-(Phenylsulfonyl)pent-1-en-3-yl)(propyl)sulfane (3e)

Sulfone **3e** was prepared according to general method C, using di-*n*-propyl sulfide (14.1 μL, 0.1 mmol) and *trans*-1,2-bis(phenylsulfonyl)ethylene (155 mg, 0.5 mmol). The crude product mixture was purified using column chromatography (5% EtOAc in toluene) to give an off-white oil (9.1 mg, 0.032 mmol, 32%). **^{1}H NMR** (400 MHz, CDCl$_3$) δ 7.94 – 7.88 (m, 2H, Ar C*H*), 7.67 – 7.59 (m, 1H, Ar C*H*), 7.59 – 7.48 (m, 2H, Ar C*H*), 6.78 (dd, J = 14.9, 9.5 Hz, 1H, SCHC*H*=CH), 6.29 (dd, J = 14.9, 0.8 Hz, 1H, SCHCH=C*H*), 3.22 – 3.12 (m, 1H, SC*H*), 2.30 – 2.16 (m, 2H, SCH_2CH$_2$CH$_3$), 1.77 – 1.59 (m, 2H, SCHCH_2), 1.52 – 1.37 (m, 2H, SCH$_2$CH_2CH$_3$), 0.97 (t, J = 7.4 Hz, 3H, SCHCH$_2$CH_3), 0.85 (t, J = 7.3 Hz, 3H, SCH$_2$CH$_2$CH_3). **^{13}C NMR** (101 MHz, CDCl$_3$) δ 146.2 (SCH*C*H=CH), 140.8 (Ar *C*), 133.3 (Ar *CH*), 130.0 (SCHCH=*C*H), 129.3 (Ar *CH*), 127.5 (Ar *CH*), 47.4 9 (S*C*H), 32.6 (S*C*H$_2$), 26.6 (SCH*C*H$_2$), 22.7 (SCH$_2$*C*H$_2$CH$_3$), 13.3 (SCH$_2$CH$_2$*C*H$_3$), 11.7 (SCHCH$_2$*C*H$_3$). **v_{max}** (thin film/cm^{-1}): 2964, 1446, 1319, 1145, 1085, 688, 599, 427. **HRMS** $C_{14}H_{21}S_2O_2$ $(M+H)^+$ Calculated 285.0977, found 258.0981.

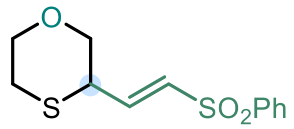

3f

(*E*)-3-(2-(Phenylsulfonyl)vinyl)-1,4-oxathiane (3f)

Sulfone **3f** was prepared according to general method C, using 1,4-oxathiane (9.5 μL, 0.1 mmol) and *trans*-1,2-bis(phenylsulfonyl)ethylene (155 mg, 0.5 mmol). The crude product

mixture was purified using column chromatography (10% MeCN in toluene) to give a colourless oil (10.8 mg, 0.040 mmol, 40%). ^{1}**H NMR** (400 MHz, $CDCl_3$) δ 7.93 – 7.82 (m, 2H, Ar C*H*), 7.66 – 7.58 (m, 1H, Ar C*H*), 7.57 – 7.50 (m, 2H, Ar C*H*), 7.01 (dd, *J* = 15.1, 6.7 Hz, 1H, SCHC*H*=CH), 6.63 (dd, *J* = 15.1, 1.4 Hz, 1H, SCHCH=C*H*), 4.04 (dd, *J* = 12.0, 2.9 Hz, 1H, $SCHCH_aH_bO$), 3.89 – 3.79 (m, 3H, SCH_2CH_2O + $SCHCH_aH_bO$), 3.43 – 3.36 (m, 1H, SC*H*), 2.72 – 2.63 (m, 1H, SCH_aH_b), 2.56 – 2.46 (m, 1H, SCH_aH_b). ^{13}**C NMR** (101 MHz, $CDCl_3$) δ 142.1 (SCH*C*H=CH), 140.3 (Ar *C*), 133.7 (Ar *C*H), 132.7 (SCHCH=*C*H), 129.5 (Ar *C*H), 127.9 (Ar *C*H), 71.1 (OCH_2CHS), 68.3 (OCH_2CH_2S), 37.9 (S*C*H), 25.1 (SCH_2). **ν_{max}** (thin film/cm^{-1}): 1584, 1446, 1308, 1145, 1085, 752, 686, 574, 429. **HRMS** $C_{12}H_{14}S_2O_3Na$ $(M+Na)^+$ Calculated 293.0282, found 293.0288.

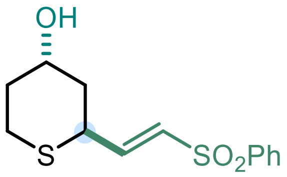

3g

***rac-2S,4S*-(*E*)-2-(2-(phenylsulfonyl)vinyl)tetrahydrothiopyran-4-ol (3g)**

Sulfone **3g** was prepared according to general method C, using tetrahydrothiopyran-4-ol (11.8 mg, 0.1 mmol) and *trans*-1,2-bis(phenylsulfonyl)ethylene (155 mg, 0.5 mmol). The crude product mixture was purified using column chromatography (4% MeOH in CH_2Cl_2) to give a pale yellow oil (10.8 mg, 0.038 mmol, 38%, dr >20:1). ^{1}**H NMR** (500 MHz, $CDCl_3$) δ 7.89 – 7.86 (m, 2H, Ar C*H*), 7.64 – 7.60 (m, 1H, Ar C*H*), 7.56 – 7.52 (m, 2H, Ar C*H*), 6.95 (dd, *J* = 15.0, 6.4 Hz, 1H, $PhO_2SCH=CH$), 6.50 (dd, *J* = 15.0, 1.6 Hz, 1H, $PhO_2SCH=CH$), 4.11 – 4.04 (m, 1H, C*H*OH), 3.96 – 3.90 (m, 1H, SC*H*), 3.00 (ddd, *J* = 13.4, 9.7, 3.5 Hz, 1H, $SCH_2CH_aH_b$), 2.55 – 2.46 (m, 1H, $SCH_2CH_aH_b$), 2.11 – 2.04 (m, 1H, $SCHCH_aH_b$), 1.99 – 1.86 (m, 3H, SCH_2 + $SCHCH_aH_b$). ^{13}**C NMR** (126 MHz, $CDCl_3$) δ 145.5 ($PhO_2SCH=CH$), 140.3 (Ar *C*), 133.5 (Ar *C*H), 131.7 ($PhO_2SCH=CH$), 129.4 (Ar *C*H), 127.9 (Ar *C*H), 65.1 (*C*HOH), 39.5 ($SCHCH_2$), 37.5 (S*C*H), 34.1 (SCH_2), 23.6 (SCH_2CH_2). **ν_{max}** (thin film/cm^{-1}): 3009, 1558, 1308, 1080, 937,

857, 765, 704, 585, 503, 417. **HRMS** $C_{13}H_{16}S_2O_3Na$ $(M+Na)^+$ Calculated 307.0439, found 307.0444.

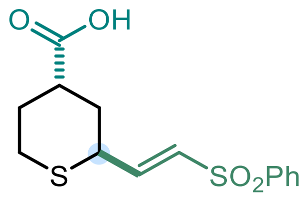

3h

***rac*-*2S,4S*-(*E*)-2-(2-(Phenylsulfonyl)vinyl)tetrahydrothiopyran-4-carboxylic acid (3h)**

Sulfone **3h** was prepared according to general method C, using tetrahydrothiopyran-4-carboxylic acid (14.6 mg, 0.1 mmol) and *trans*-1,2-bis(phenylsulfonyl)ethylene (155 mg, 0.5 mmol). The crude product mixture was purified using column chromatography (1:1 hexane in EtOAc, 1% AcOH) to give a pale yellow oil (9.4 mg, 0.030 mmol, 30%, dr >20:1). **^{1}H NMR** (400 MHz, $CDCl_3$) δ 7.91 – 7.86 (m, 2H, Ar C*H*), 7.67 – 7.62 (m, 1H, Ar C*H*), 7.58 – 7.54 (m, 2H, Ar C*H*), 7.00 (dd, *J* = 15.0, 5.9 Hz, 1H, $PhO_2SCH{=}CH$), 6.59 (dd, *J* = 15.0, 1.7 Hz, 1H, $PhO_2SCH{=}CH$), 3.74 – 3.69 (m, 1H, SC*H*), 2.73 – 2.63 (m, 3H, SCH_2 + C*H*COOH), 2.37 – 2.26 (m, 1H, $SCHCH_aH_b$), 2.14 – 2.05 (m, 4H, $SCHCH_aH_b$ + COO*H* + SCH_2CH_2). **^{13}C NMR** (101 MHz, $CDCl_3$) δ 178.2 (C=O), 144.8 ($PhO_2SCH{=}CH$), 140.1 (Ar *C*), 133.6 (Ar *C*H), 132.4 ($PhO_2SCH{=}CH$), 129.4 (Ar *C*H), 127.7 (Ar *C*H), 38.1 (S*C*H), 37.9 (*C*HCOOH), 33.1 ($SCHCH_2$), 29.5 (SCH_2CH_2), 27.5 (SCH_2). **v_{max}** (thin film/cm^{-1}): 3008, 1701, 1524, 1458, 1308, 1145, 1085, 763, 705, 554, 521, 422. **HRMS** $C_{14}H_{16}S_2O_4$ $(M+Na)^+$ Calculated 335.0388, found 335.0392.

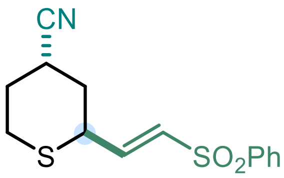

3i

***rac*-*2S,4S*-(*E*)-2-(2-(Phenylsulfonyl)vinyl)tetrahydro-2*H*-thiopyran-4-carbonitrile (3i)**

Sulfone **3i** was prepared according to general method C, using tetrahydrothiopyran-4-carbonitrile (12.7 mg, 0.1 mmol) and trans-1,2-bis(phenylsulfonyl)ethylene (155 mg, 0.5 mmol). The crude product mixture was purified using column chromatography (1% MeOH in

CH_2Cl_2) to give a colourless oil (9.7 mg, 0.033 mmol, 33%, dr >20:1). **^{1}H NMR** (400 MHz, $CDCl_3$) δ 7.91 – 7.86 (m, 2H, Ar C*H*), 7.86 – 7.61 (m, 1H, Ar C*H*), 7.59 – 7.53 (m, 2H, Ar C*H*), 6.89 (dd, *J* = 15.1, 6.7 Hz, 1H, PhO_2SCH=C*H*), 6.55 (dd, *J* = 15.1, 1.4 Hz, 1H, PhO_2SC*H*=CH), 3.91 – 3.76 (m, 1H, SC*H*), 3.15 – 3.07 (m, 1H, C*H*CN), 3.07 – 2.97 (m, 1H, SCH_aH_b), 2.69 – 2.62 (m, 1H, SCH_aH_b), 2.36 – 2.30 (m, 1H, $SCHCH_aH_b$), 2.25 – 2.15 (m, 1H, $SCH_2CH_aH_b$), 2.02 – 1.91 (m, 2H, $SCH_2CH_aH_b$ + $SCHCH_aH_b$). **^{13}C NMR** (101 MHz, $CDCl_3$) δ 143.0 (PhO_2SCH=*C*H), 139.8 (Ar *C*), 133.8 (Ar *C*H), 133.1 (PhO_2S*C*H=CH), 129.5 (Ar *C*H), 127.8 (Ar *C*H), 120.2 (*C*N), 38.0 (S*C*H), 34.3 (SCH*C*H_2), 28.7 (SCH_2*C*H_2), 26.6 (*C*HCN), 24.9 (S*C*H_2). **v_{max}** (thin film/cm^{-1}): 1523, 1308, 858, 765, 704, 585, 520, 418. $C_{14}H_{16}NS_2O_2$ $(M+H)^+$ Calculated 294.0617, found 294.0611.

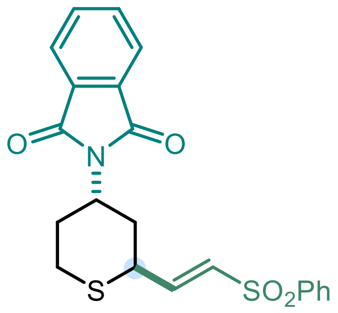

3j

***rac*-2*S*,4*S*-(*E*)-2-(2-(2-(phenylsulfonyl)vinyl)tetrahydrothiopyran-4-yl)isoindoline-1,3-dione (3j)**

Sulfone **3j** was prepared according to general method C, using 2-(tetrahydrothiopyran-4-yl)isoindoline-1,3-dione (24.7 mg, 0.1 mmol) and *trans*-1,2-bis(phenylsulfonyl)ethylene (155 mg, 0.5 mmol). The crude product mixture was purified using column chromatography (10% MeCN in toluene) to give a white amorphous solid (24.4 mg, 0.059 mmol, 59%, dr >20:1). **^{1}H NMR** (500 MHz, $CDCl_3$) δ 7.99 – 7.95 (m, 2H, Ar C*H*), 7.86 – 7.81 (m, 2H, Ar C*H*), 7.74 – 7.70 (m, 2H, Ar C*H*), 7.66 – 7.62 (m, 1H, Ar C*H*), 7.61 – 7.57 (m, 2H, Ar C*H*), 7.17 (dd, *J* = 15.0, 5.1 Hz, 1H, PhO_2SCH=*C*H), 6.75 (dd, *J* = 15.0, 1.9 Hz, 1H, PhO_2S*C*H=CH), 4.18 (tt, *J* = 12.4, 3.4 Hz, 1H, C*H*N), 3.81 – 3.75 (m, 1H, SC*H*), 3.01 (td, *J* = 13.0, 4.9 Hz, 1H, $SCHCH_aH_b$), 2.86 – 2.71 (m, 1H, SCH_aH_b), 2.70 – 2.54 (m, 2H, $SCH_2CH_aH_b$ + SCH_aH_b), 2.15 – 2.06 (m, 1H,

SCHCH_aH$_b$), 2.04 – 1.96 (m, 1H, SCH$_2$CH_aH$_b$). **^{13}C NMR** (126 MHz, $CDCl_3$) δ 167.9 (*C*O), 145.0 (PhO$_2$SCH=*C*H), 140.3 (Ar *C*), 134.2 (Ar *C*H), 133.6 (Ar *C*H), 132.8 (PhO$_2$S*C*H=CH), 131.7 (Ar *C*), 129.5 (Ar *C*H), 127.8 (Ar *C*H), 123.4 (Ar *C*H), 45.6 (*C*HNH), 39.8 (S*C*H), 34.4 (SCH*C*H$_2$), 30.8 (SCH$_2$*C*H$_2$), 25.3 (S*C*H$_2$). **v_{max}** (thin film/cm^{-1}): 1708, 1375, 1307, 1085, 719. **HRMS** $C_{21}H_{20}S_2O_4N$ (M+H)$^+$ Calculated 414.0828, found 414.0816.

General Method D:

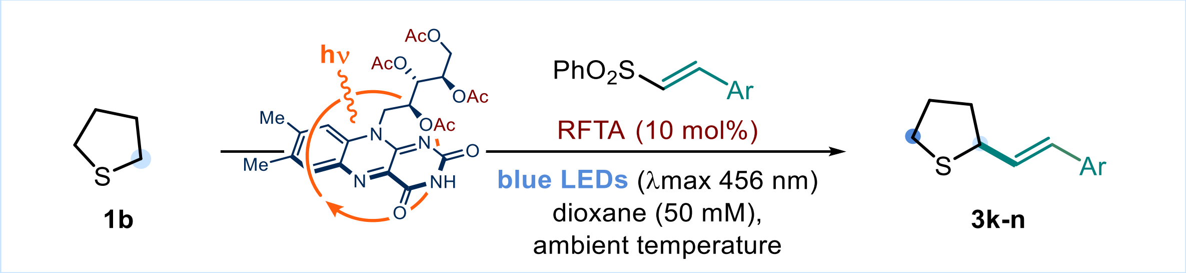

A 6 mL microwave vial with a magnetic stirrer bar was charged with riboflavin tetraacetate (5.4 mg, 10 mol%), styrene derivative (0.5 mmol), and tetrahydrothiophene (8.8 µL, 0.1 mmol), and placed under a nitrogen atmosphere. The reagents were dissolved in degassed 1,4-dioxane (2 mL, 50 mM) and irradiated with blue LEDs (456 nm) for 18 h with fan cooling. After completion of the reaction, the crude product mixture was concentrated *in vacuo* and purified by column chromatography.

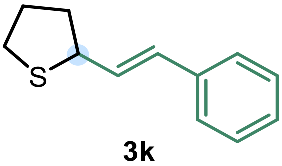

3k

(*E*)-2-Styryltetrahydrothiophene (3k)

Alkenyl sulfide **3k** was prepared according to general method D, using tetrahydrothiophene (8.8 µL, 0.1 mmol) and (*E*)-(2-(phenylsulfonyl)vinyl)benzene (122 mg, 0.5 mmol). The crude product mixture was purified using column chromatography (5% EtOAc in hexane) to give an off-white oil (7.0 mg, 0.037 mmol, 37%). **^{1}H NMR** (400 MHz, $CDCl_3$) δ 7.31 – 7.27 (m, 2H, Ar C*H*), 7.25 – 7.20 (m, 2H, Ar C*H*), 7.16 – 7.11 (m, 1H, Ar C*H*), 6.39 (d, *J* = 15.6 Hz, 1H, SCHC*H*=CHPh), 6.10 (dd, *J* = 15.6, 9.0 Hz, 1H, SCHCH=C*H*Ph), 4.05 (td, *J* = 8.4, 5.8 Hz, 1H,

SC*H*), 3.00 – 2.91 (m, 1H, SC$H_a$$H_b$), 2.90 – 2.80 (m, 1H, SC$H_a$$H_b$), 2.20 – 2.06 (m, 2H, $SCH_2C$$H_a$$H_b$ + SCHC$H_a$$H_b$), 1.96 – 1.83 (m, 1H, $SCH_2CH_a$$H_b$), 1.77 – 1.62 (m, 1H, SCHC$H_a$$H_b$). **^{13}C NMR** (101 MHz, $CDCl_3$) δ 134.2 (Ar *C*), 131.6 (SCHCH=*C*HPh), 129.7 (SCH*C*H=CHPh), 128.5 (Ar *C*H), 127.3 (Ar *C*H), 126.3 (Ar *C*H), 51.2 (S*C*H), 38.2 (SCH*C*H_2), 33.2 (S*C*H_2), 30.9 (SCH_2*C*H_2). **v_{max}** (thin film/cm^{-1}): 2231, 2144, 1978, 527, 498, 485, 458, 414. **HRMS** $C_{12}H_{15}S$ $(M+H)^+$ Calculated 191.0889, found 191.0892.

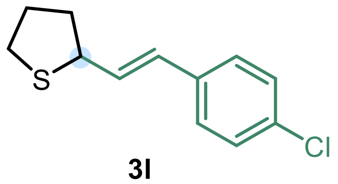

3l

(*E*)-2-(4-Chlorostyryl)tetrahydrothiophene (3l)

Alkenyl sulfide **3l** was prepared according to general method D, using tetrahydrothiophene (8.8 µL, 0.1 mmol) and (*E*)-1-chloro-4-(2-(phenylsulfonyl)vinyl)benzene (139 mg, 0.5 mmol). The crude product mixture was purified using column chromatography (5% EtOAc in hexane) to give an off-white oil (10.8 mg, 0.048 mmol, 48%). **^{1}H NMR** (400 MHz, $CDCl_3$) δ 7.28 – 7.25 (m, 4H, Ar C*H*), 6.41 (d, *J* = 15.6 Hz, 1H, SCHCH=C*H*Ar), 6.14 (dd, *J* = 15.6, 9.0 Hz, 1H, SCHC*H*=CHAr), 4.10 (td, *J* = 8.4, 5.8 Hz, 1H, SC*H*), 3.05 – 2.97 (m, 1H, SC$H_a$$H_b$), 2.96 – 2.88 (m, 1H, SC$H_a$$H_b$), 2.27 – 2.12 (m, 2H, $SCH_2C$$H_a$$H_b$ + SCHC$H_a$$H_b$), 2.02 – 1.90 (m, 1H, $SCH_2CH_a$$H_b$), 1.83 – 1.73 (m, 1H, SCHC$H_a$$H_b$). **^{13}C NMR** (101 MHz, $CDCl_3$) δ 135.3 (Ar C), 132.9 (Ar C), 132.3 (SCHCH=CHAr), 128.6 (Ar *C*H), 128.5 (SCHCH=*C*HAr), 127.5 (Ar *C*H), 51.0 (S*C*H), 38.1 (SCH*C*H_2), 33.2 (S*C*H_2), 30.9 (SCH_2*C*H_2). **v_{max}** (thin film/cm^{-1}): 2927, 1270, 983, 755, 692, 665, 607, 652. **HRMS** $C_{12}H_{14}SCl$ $(M+H)^+$ Calculated 225.0499, found 225.0504.

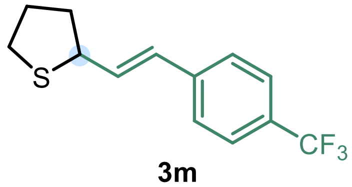

3m

(*E*)-2-(4-(Trifluoromethyl)styryl)tetrahydrothiophene (3m)

Alkenyl sulfide **3m** was prepared according to general method D, using tetrahydrothiophene (8.8 µL, 0.1 mmol) and (*E*)-1-(2-(phenylsulfonyl)vinyl)-4-(trifluoromethyl)benzene (156 mg, 0.5

mmol). The crude product mixture was purified using column chromatography (5% EtOAc in hexane) to give an off-white oil (16.5 mg, 0.064 mmol, 64%). **^{1}H NMR** (400 MHz, $CDCl_3$) δ 7.54 (d, *J* = 8.3 Hz, 2H, Ar C*H*), 7.44 (d, *J* = 8.2 Hz, 2H, Ar C*H*), 6.48 (d, *J* = 15.6 Hz, 1H, SCHCH=C*H*), 6.26 (dd, *J* = 15.6, 9.0 Hz, 1H, SCHC*H*=CH), 4.17 – 4.07 (m, 1H, SC*H*), 3.08 – 2.98 (m, 1H, SCH_aH_b), 2.97 – 2.90 (m, 1H, SCH_aH_b), 2.27 – 2.15 (m, 2H, $SCHCH_aH_b$ + $SCH_2CH_aH_b$), 2.05 –1.93 (m, 1H, $SCH_2CH_aH_b$), 1.86 – 1.95 (m, 1H, $SCHCH_aH_b$). **^{13}C NMR** (101 MHz, $CDCl_3$) δ 140.4 (Ar *C*), 134.4 (SCH*C*H=CH), 129.2 (q, *J* = 37.6 Hz, Ar *C*CF_3), 128.4 (SCHCH=*C*H), 126.5 (Ar *C*H), 125.5 (q, *J* = 3.9 Hz, Ar *C*HCCF_3), 124.2 (q, *J* = 270.1, *C*F_3), 50.9 (S*C*H), 38.1 (SCH_2CH_2), 33.3 (SCH_2), 30.9 (SCHCH_2). **^{19}F NMR** (376 MHz, $CDCl_3$) δ -62.46. **v_{max}** (thin film/cm^{-1}): 1324, 1164, 1122, 1067. **HRMS** $C_{13}H_{14}SF_3$ $(M+H)^+$ Calculated 259.0763, found 259.0751.

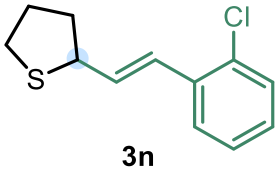

3n

(*E*)-2-(2-Chlorostyryl)tetrahydrothiophene (3n)

Alkenyl sulfide **3n** was prepared according to general method D, using tetrahydrothiophene (8.8 µL, 0.1 mmol) and (*E*)-1-chloro-2-(2-(phenylsulfonyl)vinyl)benzene (139 mg, 0.5 mmol). The crude product mixture was purified using column chromatography (5% EtOAc in hexane) to give an off-white oil (11.0 mg, 0.049 mmol, 49%). **^{1}H NMR** (400 MHz, $CDCl_3$) δ 7.54 (dd, *J* = 7.7, 1.8 Hz, 1H, Ar C*H*), 7.33 (dd, *J* = 7.7, 1.5 Hz, 1H, Ar C*H*), 7.21 – 7.13 (m, 2H, Ar C*H*), 6.84 (d, *J* = 15.6 Hz, 1H, SCHCH=C*H*), 6.16 (dd, *J* = 15.6, 9.0 Hz, 1H, SCHC*H*=CH), 4.22 – 4.11 (m, 1H, SC*H*), 3.07 – 2.97 (m, 1H, SCH_aH_b), 2.96 – 2.88 (m, 1H, SCH_aH_b), 2.30 – 2.12 (m, 2H, $SCHCH_aH_b$ + $SCH_2CH_aH_b$), 2.04 – 1.93 (m, 1H, $SCH_2CH_aH_b$), 1.86 – 1.73 (m, 1H, $SCHCH_aH_b$). **^{13}C NMR** (101 MHz, $CDCl_3$) δ 134.9 (Ar *C*), 134.4 (SCH*C*H=CH), 132.8 (Ar *C*Cl), 129.7 (Ar *C*H), 128.4 (Ar *C*H), 126.8 (Ar *C*H), 126.8 (Ar *C*H), 125.7 (SCHCH=*C*H), 51.1 (S*C*H), 38.2 (SCHCH_2), 33.3 (SCH_2), 30.9 (SCH_2CH_2). **v_{max}** (thin film/cm^{-1}): 2927, 1470, 1439, 1034, 753, 527, 458. **HRMS** $C_{12}H_{14}SCl$ $(M+H)^+$ Calculated 225.0499, found 225.0504.

Possible Origin of Trans-Selectivity in Alkenylated Products

We propose that the *trans*-selectivity of the radical alkenylation arises from a preferred conformation for the radical intermediate. For sulfinate elimination, the singly occupied p-orbital must sit *anti* to the leaving group. Radical elimination through the least sterically hindered conformation, leads to the *trans*-alkene product.

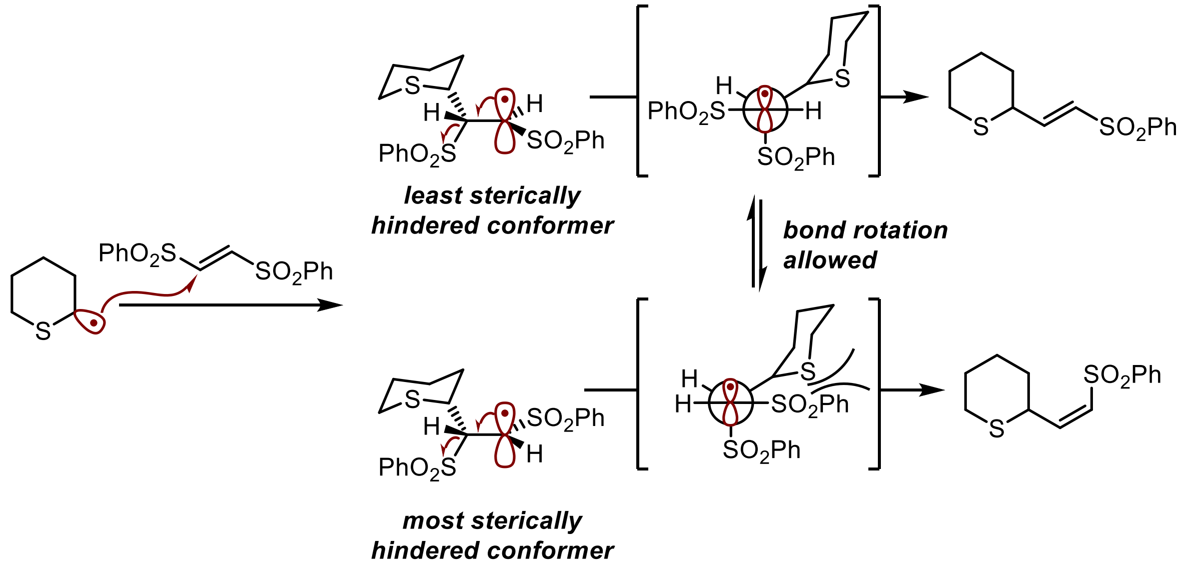

Alkynylation Scope

General Method E:

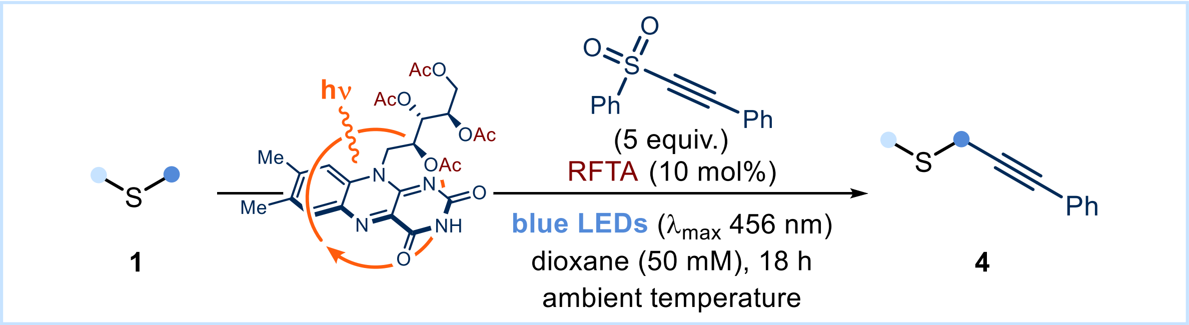

A 6 mL microwave vial with a magnetic stirrer bar was charged with riboflavin tetraacetate (5.4 mg, 10 mol%), 1-methyl-4-((phenylethynyl)sulfonyl)benzene (128 mg, 0.5 mmol), and sulfide (0.1 mmol, 1 equiv.), and placed under a nitrogen atmosphere. The reagents were dissolved in degassed 1,4-dioxane (2 mL, 50 mM) and irradiated with blue LEDs (456 nm) for 18 h with fan cooling. After completion of the reaction, the crude product mixture was concentrated *in vacuo* and purified by column chromatography.

The data are in accordance with the literature.[20]

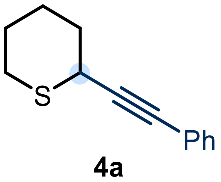

4a

2-(Phenylethynyl)tetrahydrothiopyran (4a)

Alkynyl sulfide **4a** was prepared according to general method E, using tetrahydrothiopyran (10.3 μL, 0.1 mmol) and 1-methyl-4-((phenylethynyl)sulfonyl)benzene (128 mg, 0.5 mmol). The crude product mixture was purified using column chromatography (10% EtOAc in hexane) to give an off-white oil (8.7 mg, 0.043 mmol, 43%). **^{1}H NMR** (400 MHz, $CDCl_3$) δ 7.47 – 7.40 (m, 2H, ArC*H*), 7.32 – 7.27 (m, 3H, Ar C*H*), 3.79 (dd, *J* = 7.0, 3.4 Hz, 1H, SC*H*), 2.99 – 2.88 (m, 1H, SCH_aH_b), 2.68 – 2.58 (m, 1H, SCH_aH_b), 2.20 – 2.08 (m, 1H, $SCHCH_aH_b$), 2.07 –1.90 (m, 2H, $SCH_2CH_2CH_aH_b$ + $SCHCH_aH_b$), 1.89 – 1.77 (m, 2H, SCH_2CH_2), 1.63 – 1.56 (m, 1H, $SCH_2CH_2CH_aH_b$).**^{13}C NMR** (101 MHz, $CDCl_3$) δ 131.7 (Ar *C*H), 128.1 (Ar *C*H), 128.0 (Ar *C*H),

123.2 (Ar *C*), 89.1 (SCH*C*CPh), 83.3 (SCHC*C*Ph), 33.7 (SCH*C*H_2), 31.3 (S*C*H), 27.8 (S*C*H_2), 26.9 (SCH_2*C*H_2), 23.5 (SCH_2CH_2*C*H_2). **v_{max}** (thin film/cm^{-1}): 2925, 1446, 1307, 1145, 1085, 754, 721, 687, 599. **HRMS** $C_{13}H_{15}S$ $(M+H)^+$ Calculated 203.0889, found 203.0888.

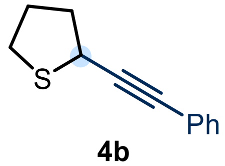

4b

2-(Phenylethynyl)tetrahydrothiophene (4b)

Alkynyl sulfide **4b** was prepared according to general method E, using tetrahydrothiophene (8.8 µL, 0.1 mmol) and 1-methyl-4-((phenylethynyl)sulfonyl)benzene (128 mg, 0.5 mmol). The crude product mixture was purified using column chromatography (10% EtOAc in hexane) to give an off-white oil (10.3 mg, 0.055 mmol, 55%). **^{1}H NMR** (400 MHz, $CDCl_3$) δ 7.44 – 7.36 (m, 2H, Ar C*H*), 7.30 – 7.26 (m, 3H, Ar C*H*), 4.27 (t, *J* = 5.7 Hz, 1H, SC*H*), 3.19 – 3.06 (m, 1H, SCH_aH_b), 2.99 – 2.89 (m, 1H, SCH_aH_b), 2.30 – 2.02 (m, 4H, SCHCH_2, SCH_2CH_2). **^{13}C NMR** (101 MHz, $CDCl_3$) δ 131.6 (Ar *C*H), 128.2 (Ar *C*H), 128.0 (Ar *C*H), 123.3 (Ar *C*), 90.7 (SCHC*C*), 82.8 (SCHC*C*), 39.0 (SCHC*H*$_2$), 37.0 (S*C*H), 32.9 (S*C*H_2), 30.5 (SCH_2*C*H_2). **v_{max}** (thin film/cm^{-1}): 2926, 1669, 1596, 1489, 1447, 1262, 1001, 758, 691, 529, 456. **HRMS** $C_{12}H_{13}S$ $(M+H)^+$ Calculated 189.0732 found 189.0731.

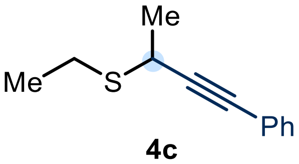

4c

Ethyl(4-phenylbut-3-yn-2-yl)sulfane (4c)

Alkynyl sulfide **4c** was prepared according to general method E, using diethyl sulfide (10.8 µL, 0.1 mmol) and 1-methyl-4-((phenylethynyl)sulfonyl)benzene (128 mg, 0.5 mmol). The crude product mixture was purified using column chromatography (5% EtOAc in hexane) to give an off-white oil (7.6 mg, 0.040 mmol, 40%). **^{1}H NMR** (400 MHz, $CDCl_3$) δ 7.47 – 7.36 (m, 2H, Ar C*H*), 7.33 – 7.25 (m, 3H, Ar C*H*), 3.89 (q, *J* = 7.0 Hz, 1H, SC*H*), 2.92 – 2.64 (m, 2H, SCH_2), 1.58 (d, *J* = 7.0 Hz, 3H, SCHCH_3), 1.33 (t, *J* = 7.4 Hz, 3H, SCH_2CH_3). **^{13}C NMR** (101 MHz,

$CDCl_3$) δ 131.6 (Ar *C*H), 128.2 (Ar *C*H), 128.0 (Ar *C*H), 123.1 (Ar *C*), 90.1 (SCHC*C*Ph), 82.9 (SCH*C*CPh), 29.4 (S*C*H), 25.4 (S*C*H_2), 21.6 (SCH*C*H_3), 14.6 (SCH_2*C*H_3). **ν_{max}** (thin film/cm^{-1}): 2929, 1669, 1489, 1447, 1314, 1019, 758, 551, 491. **HRMS** $C_{12}H_{15}S$ $(M+H)^+$ Calculated 191.0889, found 191.0894.

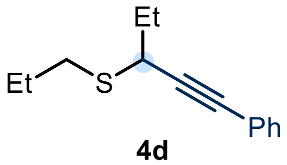

4d

(1-Phenylpent-1-yn-3-yl)(propyl)sulfane (4d)

Alkynyl sulfide **4d** was prepared according to general method E, using dipropyl sulfide (14.1 μL, 0.1 mmol) and 1-methyl-4-((phenylethynyl)sulfonyl)benzene (128 mg, 0.5 mmol). The crude product mixture was purified using column chromatography (10% CH_2Cl_2 in hexane) to give an off-white oil (7.0 mg, 0.032 mmol, 32%). **^{1}H NMR** (400 MHz, $CDCl_3$) δ 7.29 – 7.25 (m, 2H, Ar C*H*), 7.24 – 7.20 (m, 3H, Ar C*H*), 3.62 (t, *J* = 6.9 Hz, 1H, SC*H*), 2.78 – 2.68 (m, 1H, SCH_aH_b), 2.65 – 2.56 (m, 1H, SCH_aH_b), 1.82 – 1.70 (m, 2H, SCHCH_2), 1.70 – 1.51 (m, 2H, SCH_2CH_2), 1.07 (t, *J* = 7.4 Hz, 3H, $SCHCH_2CH_3$), 0.95 (t, *J* = 7.4 Hz, 3H, $SCH_2CH_2CH_3$). **^{13}C NMR** (101 MHz, $CDCl_3$) δ 131.4 (Ar *C*H), 128.5 (Ar *C*), 128.3 (Ar *C*H), 128.2 (Ar *C*H), 92.8 (SCHC*C*Ph), 89.3 (SCH*C*CPh), 36.9 (S*C*H), 33.3 (S*C*H_2), 28.8 (SCH*C*H_2), 22.7 (SCH_2*C*H_2), 13.7 (*C*H_3), 12.0 (*C*H_3). **ν_{max}** (thin film/cm^{-1}): 1966, 1489, 1444, 1368, 1297, 1152, 1025, 756, 690, 552. **HRMS** $C_{14}H_{19}S$ $(M+H)^+$ Calculated 219.1202, found 219.1196.

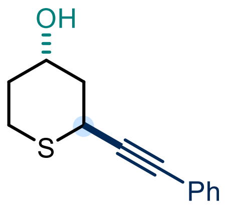

4e

***rac*-2*S*,4*S*-2-(Phenylethynyl)tetrahydrothiopyran-4-ol (4e)**

Alkynyl sulfide **4e** was prepared according to general method E, using tetrahydrothiopyran-4-ol (11.8 mg, 0.1 mmol) and 1-methyl-4-((phenylethynyl)sulfonyl)benzene (128 mg, 0.5 mmol). The crude product mixture was purified using column chromatography (40% EtOAc in hexane)

to give an off-white oil (8.7 mg, 0.040 mmol, 40%, dr >20:1). ^{1}**H NMR** (400 MHz, $CDCl_3$) δ 7.44 – 7.40 (m, 2H, Ar C*H*), 7.32 – 7.28 (m, 3H, Ar C*H*), 4.19 – 4.05 (m, 2H, C*H*OH + SC*H*), 3.03 – 2.91 (m, 1H, SCH_aH_b), 2.91 – 2.83 (m, 1H, SCH_aH_b), 2.40 – 2.31 (m, 1H, $SCHCH_aH_b$), 2.25 – 2.17 (m, 1H, $SCH_2CH_aH_b$), 2.01 (ddd, *J* = 12.8, 9.2, 3.7 Hz, 1H, $SCHCH_aH_b$), 1.82 – 1.71 (m, 1H, $SCH_2CH_aH_b$), 1.43 (d, *J* = 4.4 Hz, 1H, O*H*). ^{13}**C NMR** (101 MHz, $CDCl_3$) δ 131.6 (Ar *C*H), 129.0 (Ar *C*), 128.2 (Ar *C*H), 128.1 (Ar *C*H), 88.5 (SCHC*C*Ph), 83.5 (SCH*C*CPh), 66.1 (*C*HOH), 41.3 (SCH*C*H_2), 35.6 (SCH_2*C*H_2), 29.8 (S*C*H), 24.8 (S*C*H_2). $\mathbf{v_{max}}$ (thin film/cm^{-1}): 3806, 2090, 1128, 1041, 759, 661, 591, 574, 554, 532, 525, 491. **HRMS** $C_{13}H_{15}OS$ $(M+H)^+$ Calculated 219.0838, found 219.0843.

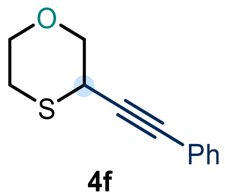

4f

***rac*-*2S,4S*-3-(Phenylethynyl)-1,4-oxathiane (4f)**

Alkynyl sulfide **4f** was prepared according to general method E, using 1,4-oxathiane (9.5 μL, 0.1 mmol) and 1-methyl-4-((phenylethynyl)sulfonyl)benzene (128 mg, 0.5 mmol). The crude product mixture was purified using column chromatography (10% EtOAc in hexane) to give an off-white oil (7.8 mg, 0.038 mmol, 38%). ^{1}**H NMR** (400 MHz, $CDCl_3$) δ 7.48 – 7.39 (m, 2H, Ar C*H*), 7.34 – 7.27 (m, 3H, Ar C*H*), 4.23 – 4.17 (m, 1H, $SCHCH_aH_bO$), 4.05 (ddd, *J* = 11.8, 4.9, 3.0 Hz, 1H, $SCH_2CH_aH_bO$), 3.92 – 3.77 (m, 3H, SC*H* + $SCH_2CH_aH_bO$ + $SCHCH_aH_bO$), 2.91 – 2.80 (m, 1H, SCH_aH_b), 2.77 – 2.67 (m, 1H, SCH_aH_b). ^{13}**C NMR** (101 MHz, $CDCl_3$) δ 131.8 (Ar *C*H), 128.4 (Ar *C*H), 128.2 (Ar *C*H), 122.5 (Ar *C*), 85.2 (SCHC*C*Ph), 84.4 (SCH*C*CPh), 72.6 (SCH*C*H_2O), 68.3 (SCH_2*C*H_2O), 30.2 (S*C*H), 26.9 (S*C*H_2), $\mathbf{v_{max}}$ (thin film/cm^{-1}): 2924, 1490, 1100, 1051, 758, 691, 470, 450. **HRMS** $C_{12}H_{13}SO$ $(M+H)^+$ Calculated 205.0682, found 205.0680.The data are in accordance with the literature.[20]

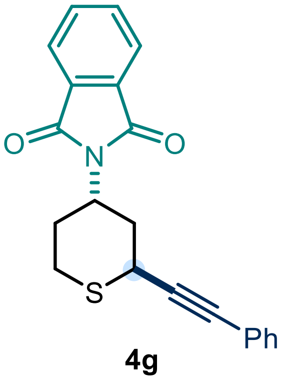

4g

***rac-2S,4S*-2-(2-(phenylethynyl)tetrahydrothiopyran-4-yl)isoindoline-1,3-dione (4g)**

Alkynyl sulfide **4g** was prepared according to general method E, using 2-(tetrahydrothiopyran-4-yl)isoindoline-1,3-dione (24.7 mg, 0.1 mmol) and 1-methyl-4-((phenylethynyl)sulfonyl)benzene (128 mg, 0.5 mmol). The crude product mixture was purified using column chromatography (CH_2Cl_2) to give a yellow oil (10.4 mg, 0.030 mmol, 30%, dr >20:1). **^{1}H NMR** (400 MHz, $CDCl_3$) δ 7.86 – 7.83 (m, 2H, Ar C*H*), 7.74 – 7.71 (m, 2H, Ar C*H*), 7.58 – 7.51 (m, 2H, Ar C*H*), 7.42 – 7.37 (m, 1H, Ar C*H*), 7.35 – 7.32 (m, 2H, Ar C*H*), 4.82 (tt, *J* = 12.4, 3.4 Hz, 1H, C*H*N), 4.13 – 4.05 (m, 1H, SC*H*), 3.36 (ddd, *J* = 13.7, 12.8, 2.6 Hz, 1H, SCH_aH_b), 3.05 – 2.91 (m, 1H, $SCHCH_aH_b$), 2.80 – 2.71 (m, 1H, SCH_aH_b), 2.69 – 2.57 (m, 1H, $SCH_2CH_aH_b$), 2.25 – 2.16 (m, 1H, $SCHCH_aH_b$), 2.13 – 2.06 (m, 1H, $SCH_2CH_aH_b$). **^{13}C NMR** (101 MHz, $CDCl_3$) δ 168.1 (*C*O), 141.1 (Ar *C*), 134.0 (Ar *C*H), 131.8 (Ar *C*H), 129.0 (Ar *C*), 128.3 (Ar *C*H), 128.2 (Ar *C*H), 123.2 (Ar *C*H), 87.8 (SCH*C*CPh), 85.2 (SCHC*C*Ph), 46.2 (*C*HN), 36.4 (SCH*C*H_2), 31.6 (S*C*H), 31.4 (SCH_2*C*H_2), 26.2 (S*C*H_2). **v_{max}** (thin film/cm^{-1}): 2147, 1711, 1374, 720, 543, 462, 449, 428. **HRMS** $C_{21}H_{18}NO_2S$ $(M+H)^+$ Calculated 348.1044, found 348.1045.

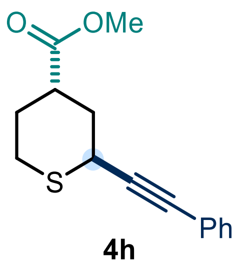

4h

***rac*-2*S*,4*S*-Methyl 2-(phenylethynyl)tetrahydrothiopyran-4-carboxylate (4h)**

Alkynyl sulfide **4i** was prepared according to general method E, using methyl tetrahydrothiopyran-4-carboxylate (16.0 μL, 0.1 mmol) and 1-methyl-4-((phenylethynyl)sulfonyl)benzene (128 mg, 0.5 mmol). The crude product mixture was purified using column chromatography (20% EtOAc in hexane) to give a yellow oil (14.3 mg, 0.055 mmol, 55%, dr >20:1). **^{1}H NMR** (400 MHz, $CDCl_3$) δ 7.48 – 7.41 (m, 2H, Ar C*H*), 7.34 – 7.27 (m, 3H, Ar C*H*), 3.96 (t, *J* = 4.1 Hz, 1H, SC*H*), 3.70 (s, 3H, OCH_3), 3.15 (ddd, *J* = 14.3, 11.9, 2.7 Hz, 1H, SCH$_a$$H_b$), 2.96 (tt, *J* = 11.5, 3.4 Hz, 1H, C*H*CO), 2.71 – 2.62 (m, 1H, SCH_aH$_b$), 2.45 – 2.37 (m, 1H, SCHCH$_a$$H_b$), 2.34 – 2.25 (m, 1H, SCH$_2$CH$_a$$H_b$), 2.18 – 2.09 (m, 1H, SCHCH_aH$_b$), 1.92 – 1.81 (m, 1H, SCH$_2$CH_aH$_b$). **^{13}C NMR** (101 MHz, $CDCl_3$) δ 175.1 (*C*O), 131.7 (Ar *C*H), 128.2 (Ar *C*H), 128.2 (Ar *C*H), 122.9 (Ar *C*), 88.1 (SCHC*C*Ph), 84.4 (SCH*C*CPh), 51.8 (O*C*H$_3$), 38.6 (*C*HCO), 34.9 (SCH*C*H$_2$), 29.8 (S*C*H), 29.6 (SCH$_2$*C*H$_2$), 25.3 (S*C*H$_2$), **ν$_{max}$** (thin film/cm^{-1}): 2926, 1731, 758, 463, 450, 429, 415. **HRMS** $C_{15}H_{17}SO_2$ (M+H)$^+$ Calculated 261.0944, found 261.0946.

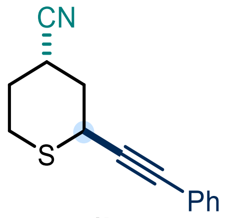

4i

***rac*-2*S*,4*S*-2-(Phenylethynyl)tetrahydrothiopyran-4-carbonitrile (4i)**

Alkynyl sulfide **4j** was prepared according to general method E, using tetrahydrothiopyran-4-carbonitrile (12.7 μL, 0.1 mmol) and 1-methyl-4-((phenylethynyl)sulfonyl)benzene (128 mg, 0.5 mmol). The crude product mixture was purified using column chromatography (20% EtOAc in hexane) to give an off-white oil (8.6 mg, 0.038 mmol, 38%, dr >20:1). **^{1}H NMR** (400 MHz,

$CDCl_3$) δ 7.46 – 7.40 (m, 2H, Ar C*H*), 7.35 – 7.29 (m, 3H, Ar C*H*), 4.03 – 3.97 (m, 1H, SC*H*), 3.19 (tt, *J* = 9.6, 3.7 Hz, 1H, SC$H_a$$H_b$), 3.03 (ddd, *J* = 14.2, 10.1, 2.9 Hz, 1H, SC$H_a$$H_b$), 2.82 – 2.75 (m, 1H, CNC*H*), 2.41 – 2.22 (m, 3H, $SCH_2CH_aH_b$ + SCHCH_2), 2.18 – 1.99 (m, 1H, $SCH_2CH_aH_b$). **^{13}C NMR** (101 MHz, $CDCl_3$) δ 131.8 (Ar *C*H), 130.7 (Ar *C*), 128.6 (Ar *C*H), 128.4 (Ar *C*H), 122.3 (*C*N), 94.1 (SCH*C*CPh), 85.1 (SCHC*C*Ph), 35.8 (SCH*C*H_2), 30.0 (SCH_2CH_2), 29.3 (S*C*H), 25.5 (CN*C*H), 24.9 (S*C*H_2). **v_{max}** (thin film/cm^{-1}): 2919, 2236, 2106, 652, 576, 525, 491, 470, 436, 417. **HRMS** $C_{14}H_{16}NS_2O_2$ $(M+H)^+$ Calculated 228.0847, found 228.0844.

Observed Byproducts

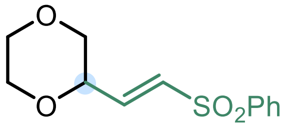

S7

(*E*)-2-(2-(phenylsulfonyl)vinyl)-1,4-dioxane (S7)

S7 was identified as a side product, arising from general method C, using tetrahydrothiopyran-4-one (11.6 mg, 0.1 mmol) and 1,2-bis(phenylsulfonyl)ethylene (155 mg, 0.5 mmol). The crude product mixture was purified using column chromatography (30% EtOAc in hexane) to give an off-white oil (5.6 mg, 0.022 mmol, 4% [yield with respect to limiting reagent, which in this case is the alkene]). **^{1}H NMR** (400 MHz, $CDCl_3$) δ 7.50 – 7.42 (m, 2H, Ar C*H*), 7.36 – 7.28 (m, 3H, Ar C*H*), 4.57 (dd, *J* = 8.5, 2.9 Hz, 1H, OC*H*), 4.00 – 3.89 (m, 2H, $OCHCH_aH_b$ + $OCH_aH_bCH_2O$), 3.80 – 3.62 (m, 4H, $OCHCH_aH_b$ + $OCH_aH_bCH_2O$ + OCH_2CH_2). **^{13}C NMR**. (101 MHz, $CDCl_3$) δ 131.9 (Ar *C*H), 128.7 (Ar *C*H), 128.3 (Ar *C*H), 121.9 (Ar *C*), 86.6 (OCH*C*CPh), 84.3 (OCHC*C*Ph), 70.4 (OCH*C*H_2O), 66.5 (O*C*H_2CH_2O), 66.4 (O*C*H), 65.8 (OCH_2CH_2O), **v_{max}** (thin film/cm^{-1}): 2919, 1489, 1456, 1287, 1119, 1091, 903, 874, 758, 694, 593, 575, 533. **HRMS** $C_{12}H_{13}O_2$ $(M+H)^+$ Calculated 189.0910, found 189.0910.

The data are in accordance with the literature.[19]

Scale-Up Procedure

General Scale-up Procedure

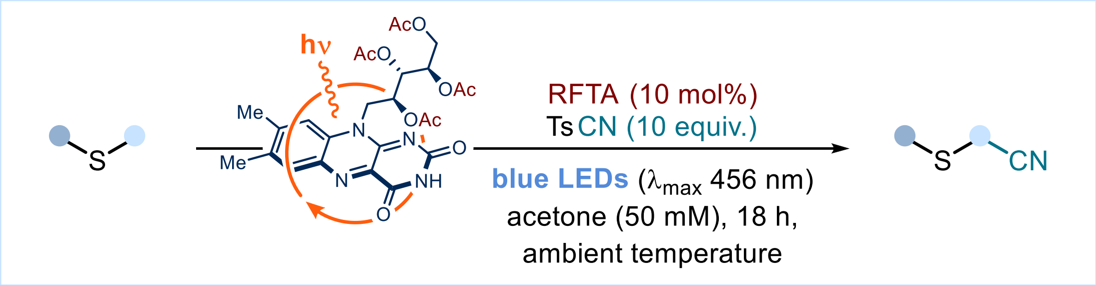

A 100 mL round-bottomed flask with a magnetic stirrer bar was charged with riboflavin tetraacetate (54.4 mg, 0.1 mmol, 0.1 equiv.), 4-tosyl cyanide (1.81 g, 10.0 mmol, 10 equiv.), sulfide (1.0 mmol, 1 equiv.) and placed under a nitrogen atmosphere. The reagents were dissolved in degassed acetone (20 mL, 50 mM) and irradiated with blue LEDs (4 × 34W Kessil 456 nm lamps) for 18 h with fan cooling. After completion of the reaction, the resulting mixture was concentrated *in vacuo* and the crude product mixture was purified by column chromatography.

Pictures of reaction set-up

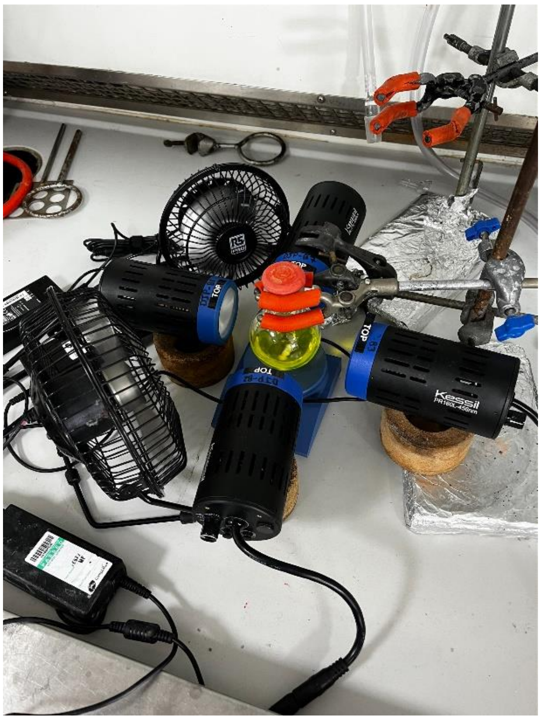

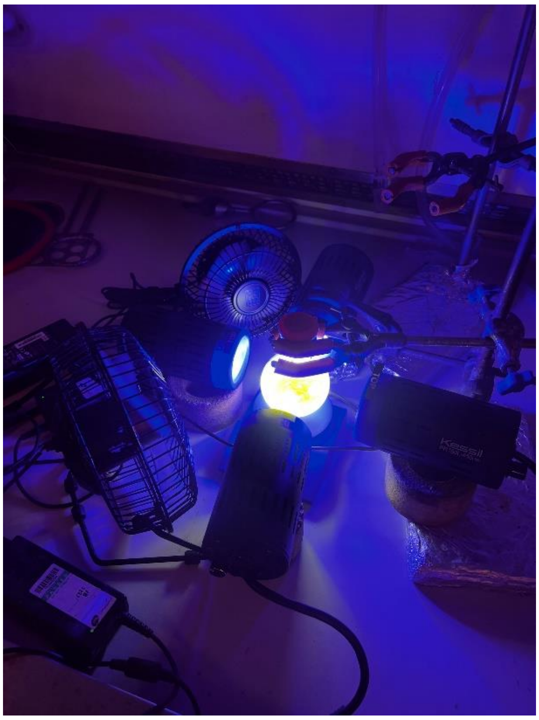

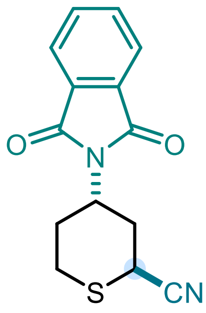

2k

4-(1,3-Dioxoisoindolin-2-yl)tetrahydrothiopyran-2-carbonitrile (2k)

Nitrile **2k** was prepared according to general procedure A using 2-(tetrahydrothiopyran-4-yl)isoindoline-1,3-dione (251 mg, 1.0 mmol, 1 equiv.), 4-tosyl cyanide (1.81 g, 10.0 mmol, 10 equiv.). The crude product mixture was purified by column chromatography (30% EtOAc in hexane) to give a white solid (133 mg, 0.49 mmol, 49%). NMR data matched with previously described data.

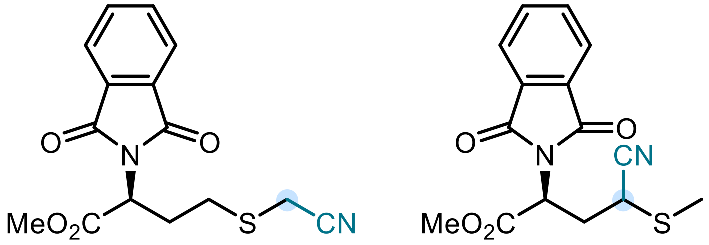

2na **2nb**

Methyl (*S*)-4-((cyanomethyl)thio)-2-(1,3-dioxoisoindolin-2-yl)butanoate (2na) and methyl (2*S*)-4-cyano-2-(1,3-dioxoisoindolin-2-yl)-4-(methylthio)butanoate (2nb)

Methionine derivatives **2na** and **2nb** were prepared according to general procedure A using *N*-phthalimido-L-methionine methyl ester (290 mg, 1.0 mmol, 1.0 equiv.), 4-tosyl cyanide (1.81 g, 10.0 mmol, 10 equiv.). The crude product mixture was purified by column chromatography (30% EtOAc in hexane) to give off white oils (**2na**: 77 mg, 0.23 mmol, 23%; **2nb**: 151 mg, 0.45 mmol, 45%; total: 68%). The regioisomers were separated (**2na:2nb**, 1:2 regioisomeric ratio), and **2nb** was produced in a 1.1:1 diastereoisomeric ratio. NMR data matched with previously described data.

Unsuccessful Examples

Unsuccessful Cyanation Substrates

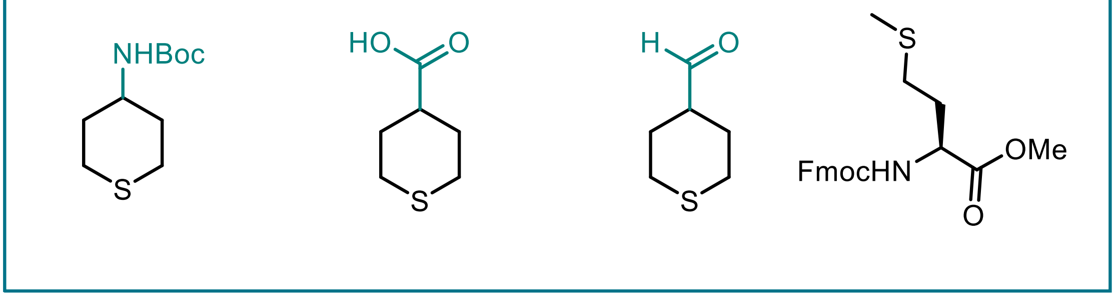

Unsuccessful Alkenylation Substrates

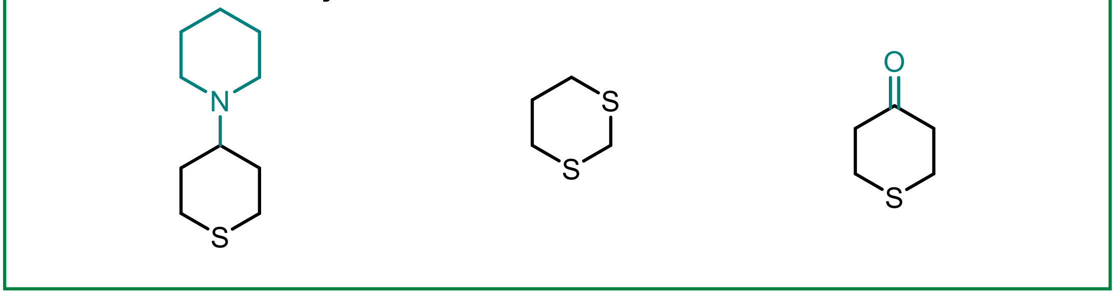

Unsuccessful Alkynylation Substrates

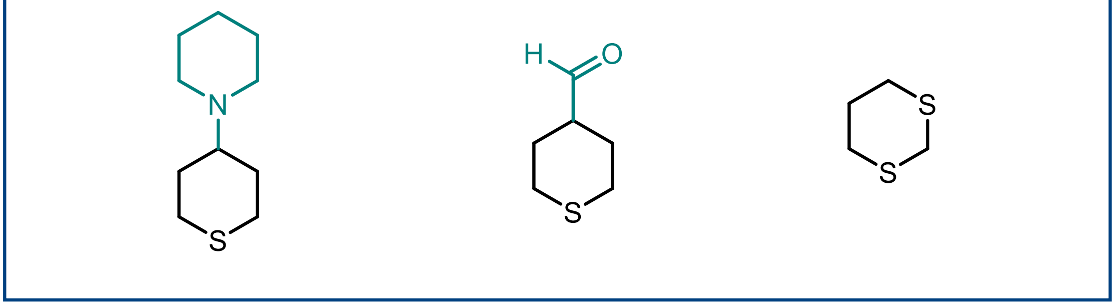

Mechanistic Studies

Stern-Volmer Quenching Studies

Experiments were performed on an Edinburgh Instruments FLS1000 fluorescence spectrometer. Stern-Volmer experiments were conducted to track the quenching of the fluorescence of riboflavin tetraacetate using both 2-(tetrahydrothiopyran-4-yl)isoindoline-1,3-dione (**1k**), 4-(1,3-dioxoisoindolin-2-yl)tetrahydrothiopyran-2-carbonitrile (**2k**), 1,4-dioxane, and tosyl cyanide. Degassed stock solutions of each component were prepared prior to each set of experiments. In a typical experiment, 1 mL of a 0.05 mM solution of riboflavin tetraacetate in acetone was added to 1 mL of the substrate in a screwtop 1.0 cm quartz cuvette, giving a final riboflavin tetraacetate concentration of 0.025 mM. Solutions were excited at a fixed wavelength of 450 nm (incident light slit set to 1.0 mm) and the emission light was acquired from 460 nm to 750 nm (max emission intensity at 530 nm) over three scans. Each measurement was then repeated three times and averaged.

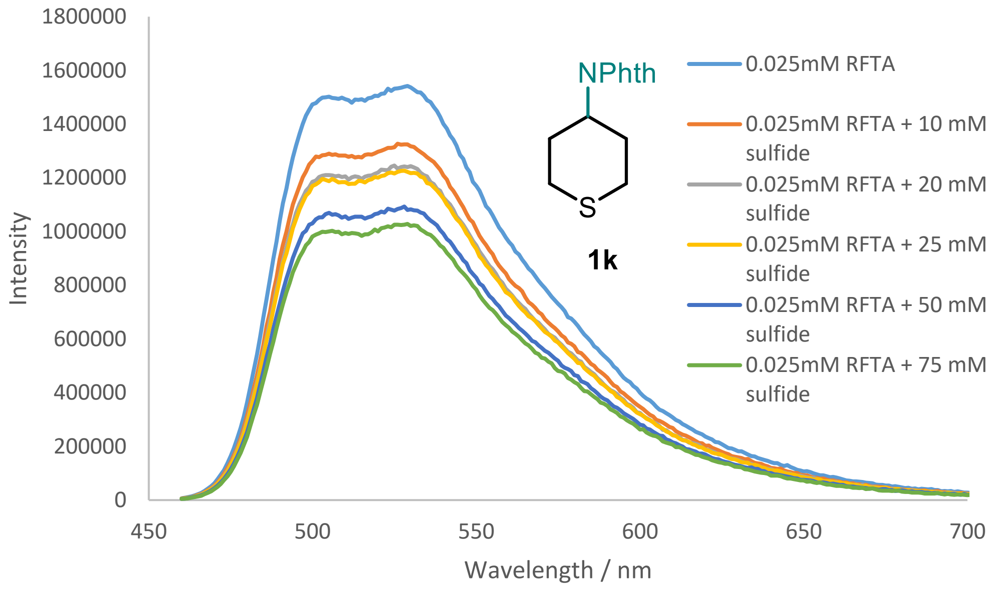

Figure S1: Quenching of riboflavin tetraacetate emission (0.025 mM in acetone) in the presence of 2-(tetrahydrothiopyran-4-yl)isoindoline-1,3-dione (**1k**).

The results shown in Figure S1 suggest that the sulfide quenches the excited state of RFTA and its emission.

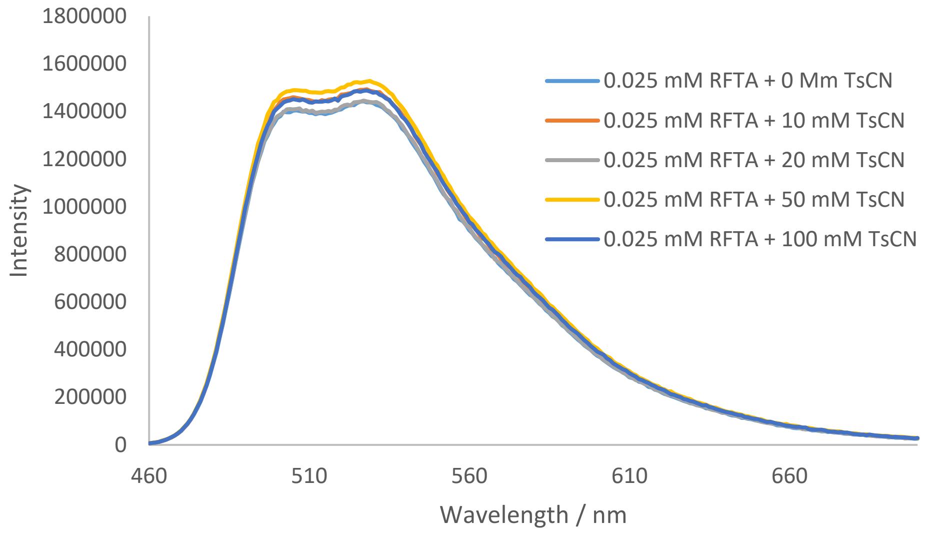

Figure S2: Quenching of riboflavin tetraacetate emission (0.025 mM in acetone) in the presence of 4-tosyl cyanide.

The results shown in Figure S2 suggest that 4-tosyl cyanide does not quench the excited state of RFTA and its emission.

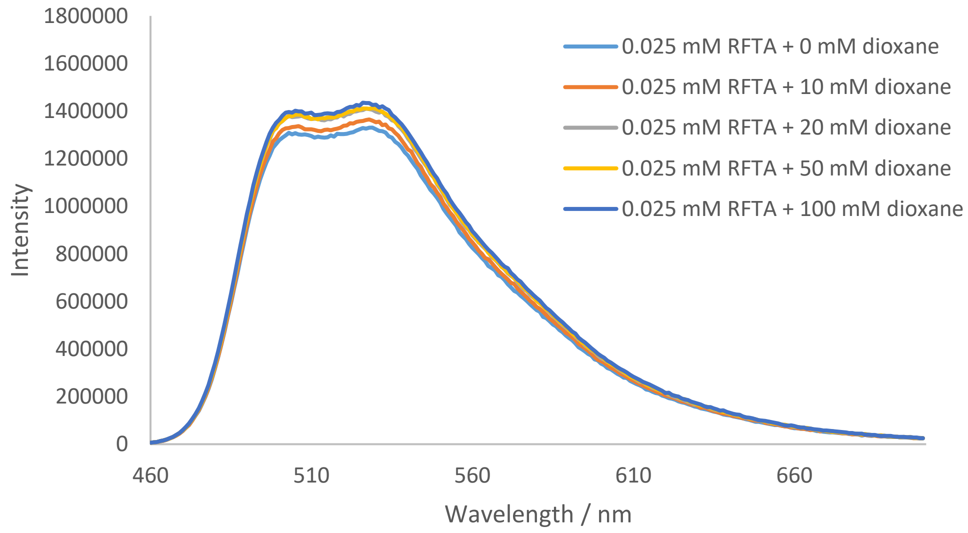

Figure S3: Quenching of riboflavin tetraacetate emission (0.025 mM in acetone) in the presence of dioxane.

The results shown in Figure S3 suggest that dioxane does not readily quench the excited state of RFTA and its emission. This means that the solvent-derived byproduct of alkenylation **S7** is likely not formed from flavin-mediated O-oxidation, rather from HAT α-oxygen in dioxane.

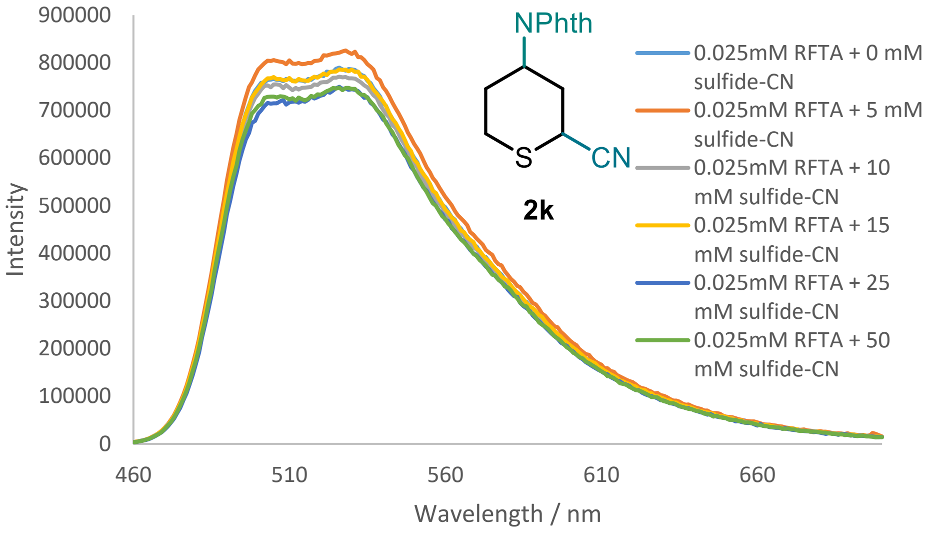

Figure S4: Quenching of riboflavin tetraacetate emission (0.025 mM in acetone) in the presence of dioxane.

The results shown in Figure S4 suggest that **2k** is weakly capable of quenching the excited state of RFTA and its emission.

The Stern-Volmer plot (Figure S1) shows a linear correlation between the increasing amounts of quencher and the ratio I_0/I. Based on the Stern-Volmer relationship (Eq. 1), it is possible to calculate the Stern-Volmer constant (K_{SV}).

$$\frac{I_0}{I} = 1 + K_{SV}[Q] \quad \textit{[Eq. 1]}$$

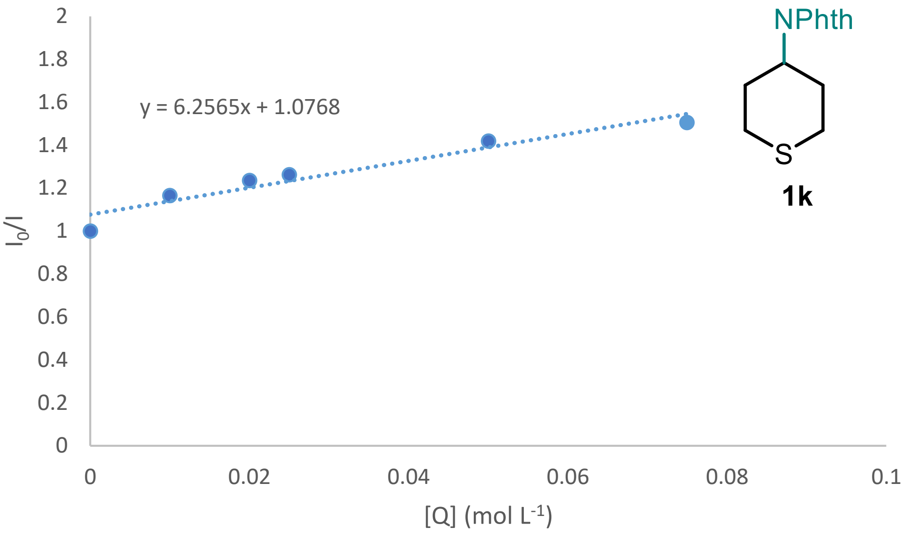

Figure S5: Stern-Volmer quenching plot of 2-(tetrahydrothiopyran-4-yl)isoindoline-1,3-dione (**1k**).

A Stern-Volmer quenching constant of 6.3 M^{-1} can be extracted from Figure S5.

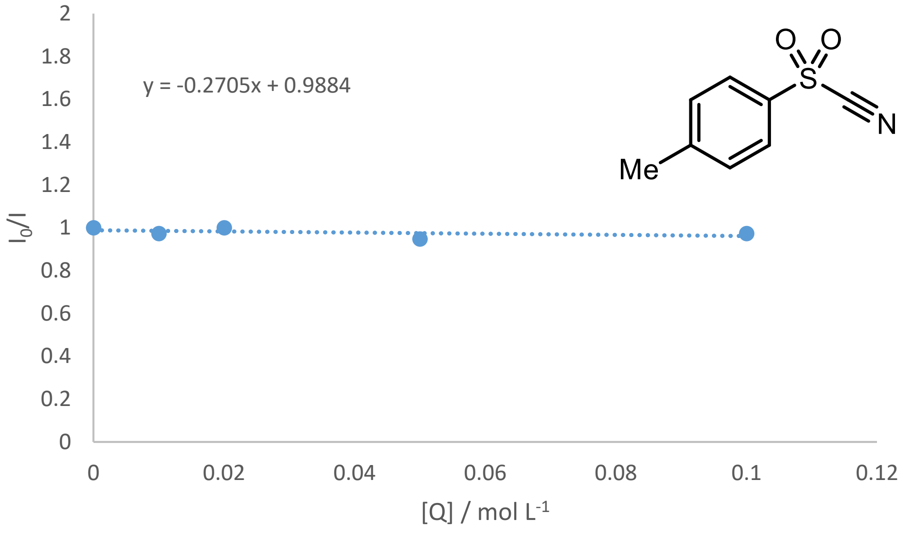

Figure S6: Stern-Volmer quenching plot of 4-tosyl cyanide.

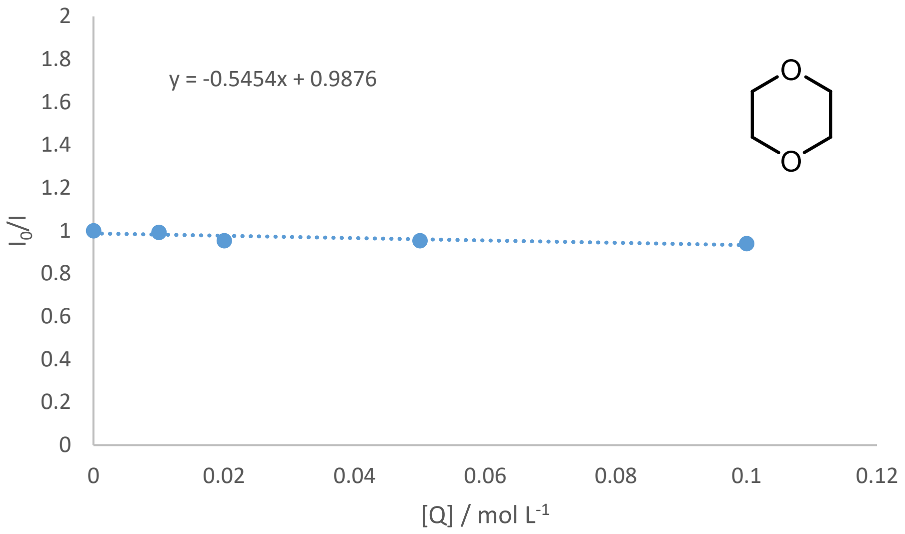

Figure S7: Stern-Volmer quenching plot of 1,4-dioxane.

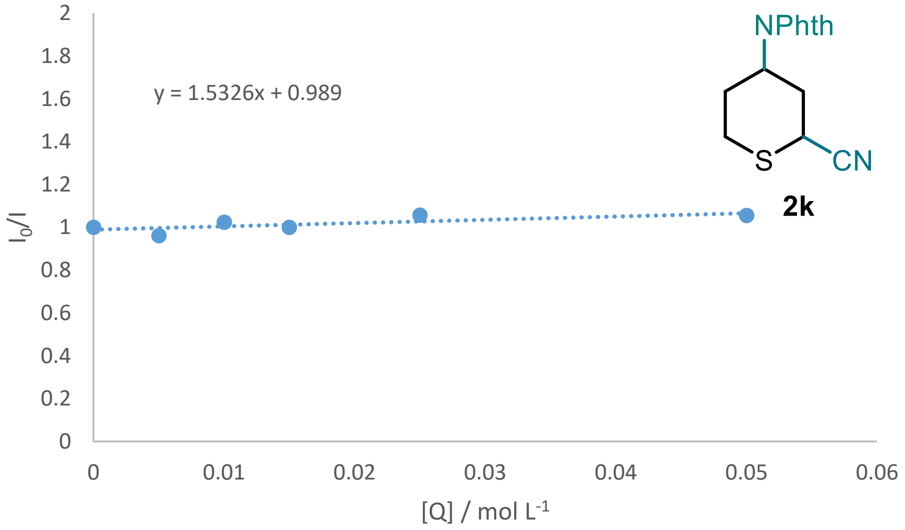

Figure S8: Stern-Volmer quenching plot of 4-(1,3-dioxoisoindolin-2-yl)tetrahydrothiopyran-2-carbonitrile (**2k**).

A Stern-Volmer quenching constant of 1.5 M^{-1} can be extracted from Figure S8, meaning that the C−H functionalised product is a poor quencher of the excited state flavin. The presence of the electron-withdrawing group at the C2 position sufficiently alters the redox potential of the sulfide so that further oxidation-trapping with RFTA and tosyl cyanide to generate

bisfunctionalized products is not feasible. This same selectivity for monofunctionalization extends to the α- to sulfur C−H alkenylation and alkynylation processes.

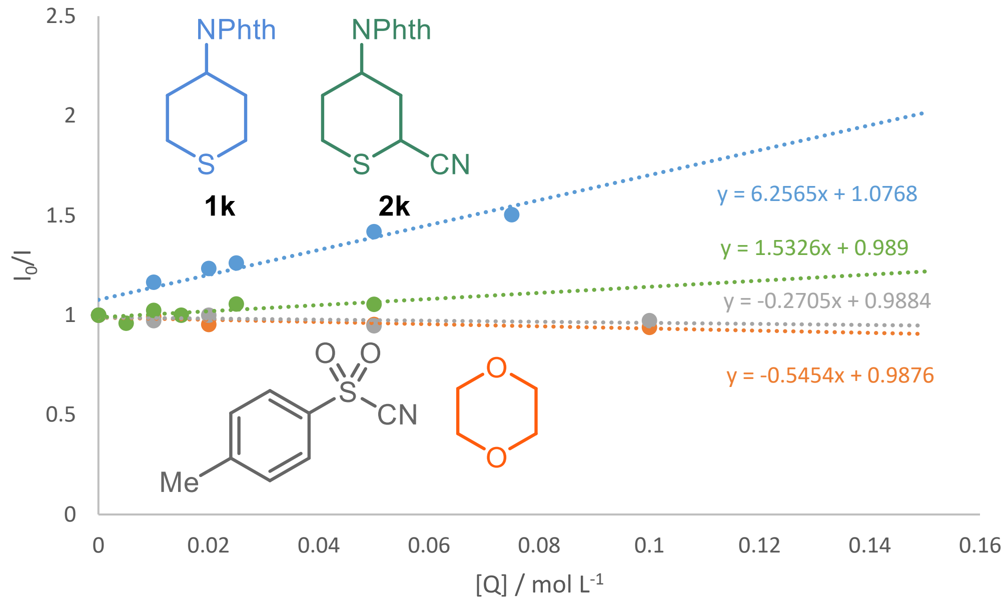

Figure S9: Stern-Volmer plots for all 4 reaction components.

Resubjection of Product to Reaction Conditions

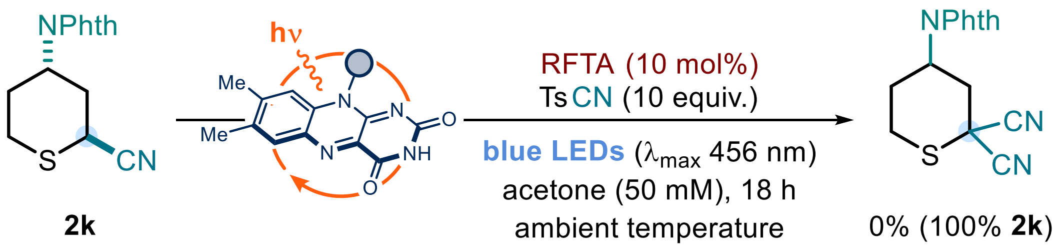

Following general method B using *rac-2S,4S*-4-(1,3-dioxoisoindolin-2-yl)tetrahydrothiopyran-2-carbonitrile (**2k**) (12.6 mg, 0.1 mmol, 1 equiv.) and 4-tosyl cyanide (181 mg, 1.0 mmol, 10 equiv.), the biscyanated product was not detected by ^{1}H NMR, with **2k** recovered in 100% yield; suggesting that the cyanated product is unable to undergo further oxidation by riboflavin tetraacetate.

^{1}H and ^{13}C NMR Spectra

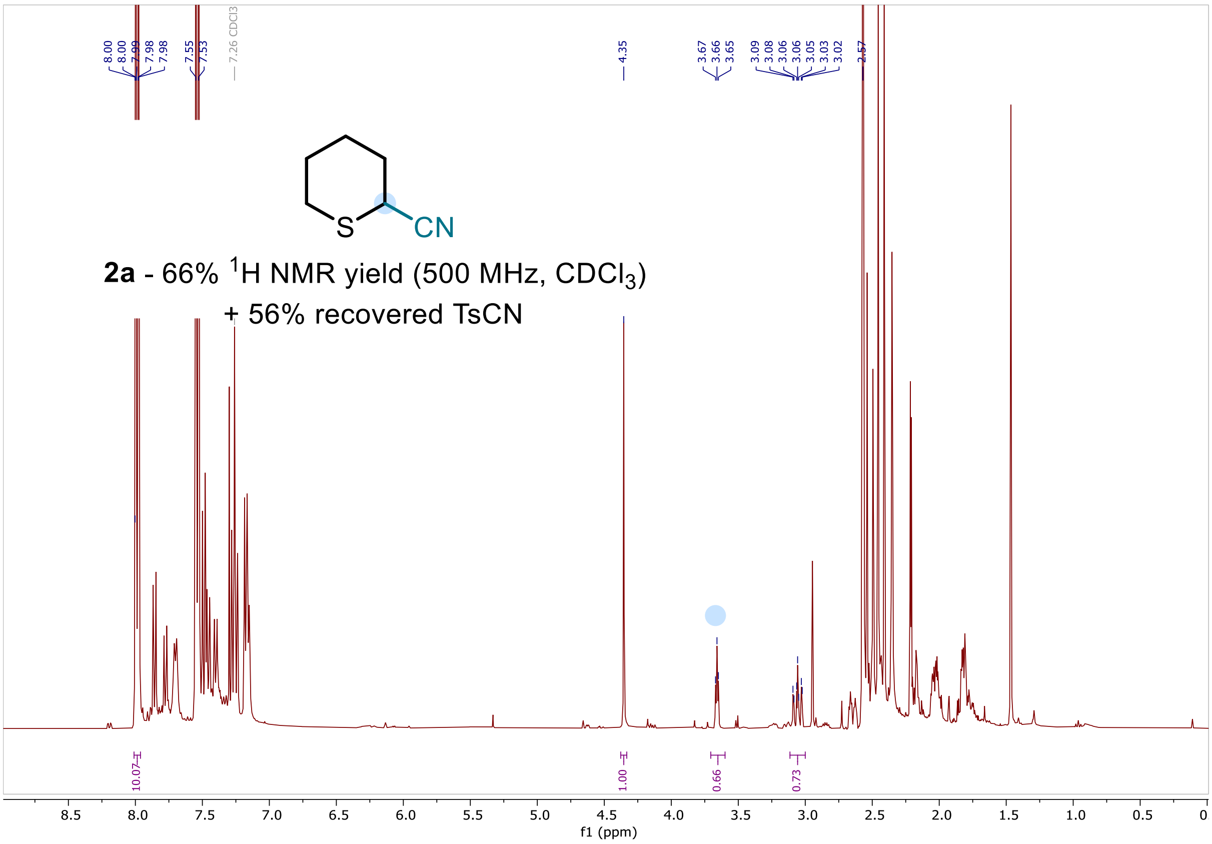

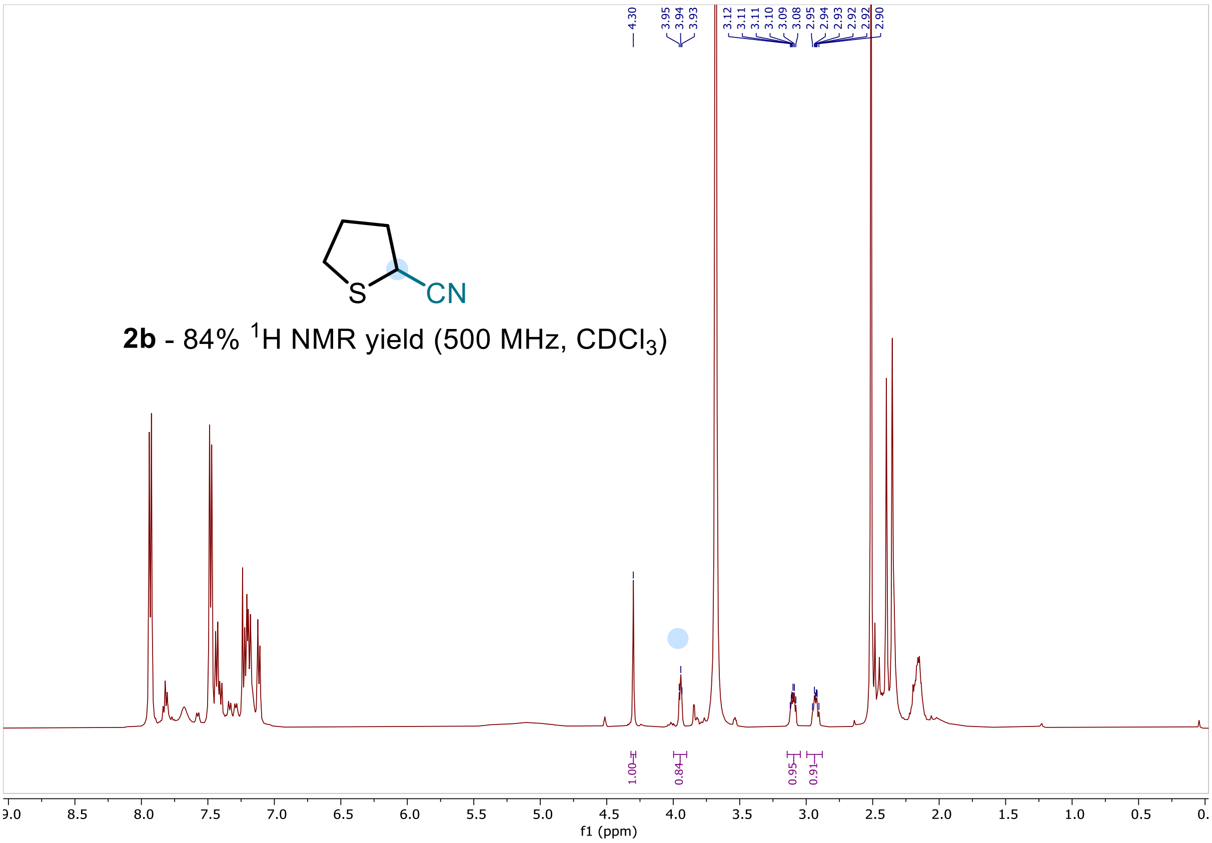

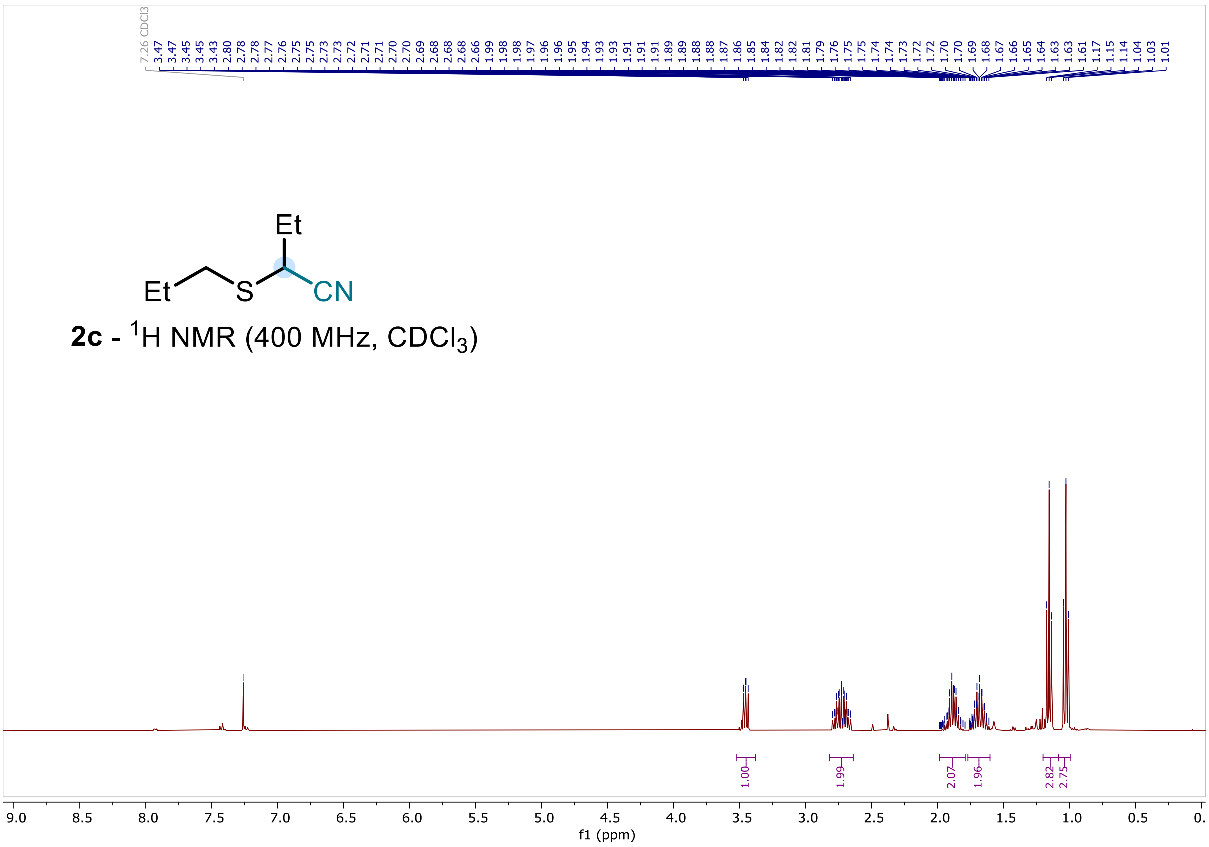

2c - ^{1}H NMR (400 MHz, $CDCl_3$)

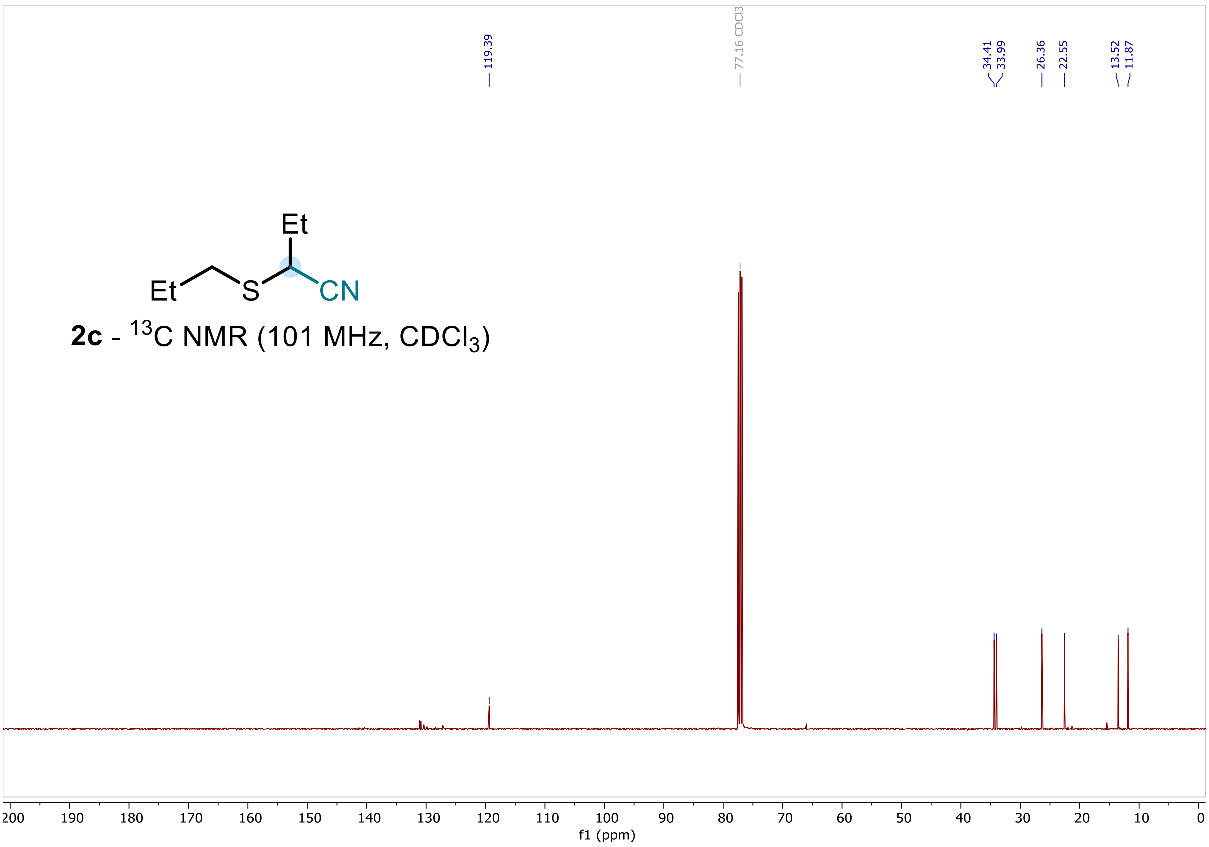

2c - ^{13}C NMR (101 MHz, $CDCl_3$)

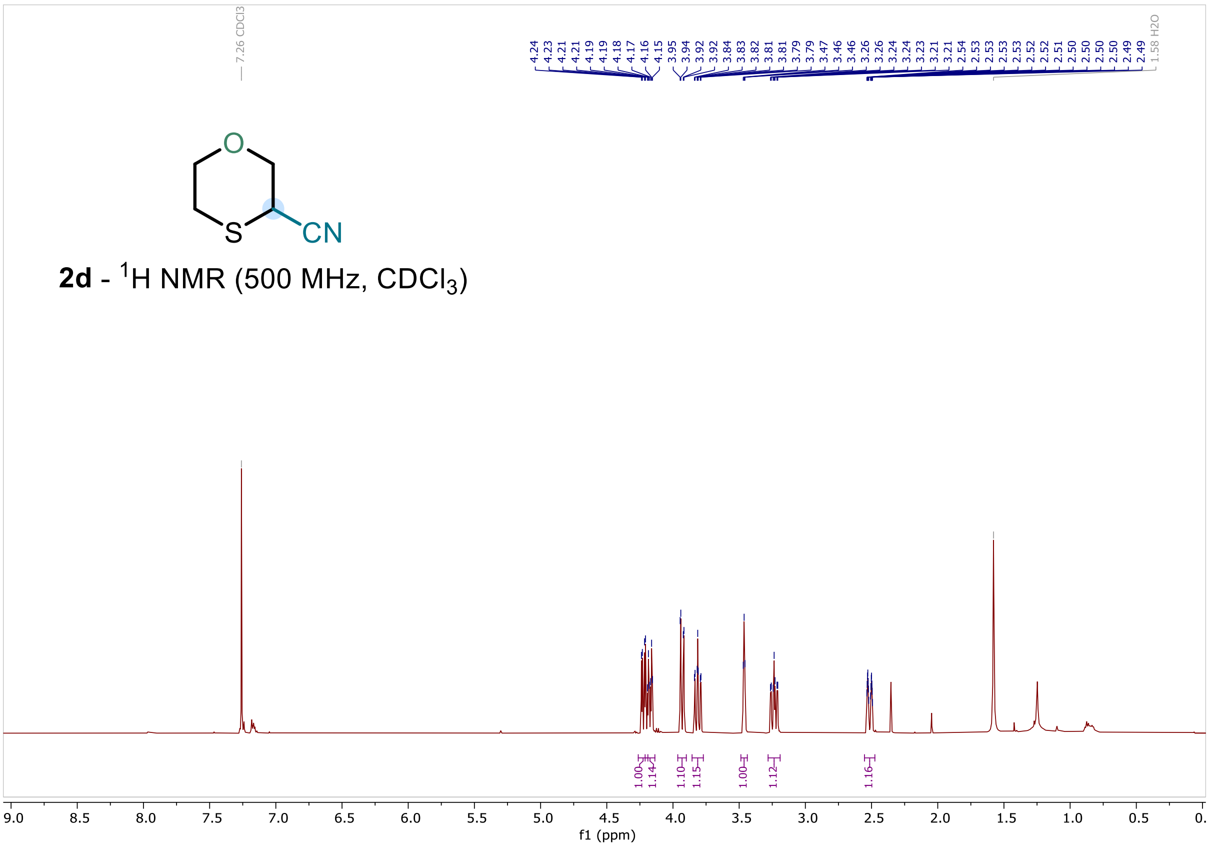
2d - 1H NMR (500 MHz, CDCl3)
7.26 CDCl3
4.24 4.23 4.21 4.21 4.19 4.19 4.18 4.17 4.16 4.15 3.95 3.94 3.92 3.92 3.84 3.83 3.82 3.81 3.81 3.79 3.47 3.46 3.46 3.26 3.26 3.24 3.24 3.23 3.21 3.21 2.54 2.53 2.53 2.53 2.53 2.52 2.52 2.51 2.50 2.50 2.50 2.50 2.49 2.49
1.58 H2O
1.00 1.14 1.10 1.15 1.00 1.12 1.16
f1 (ppm)

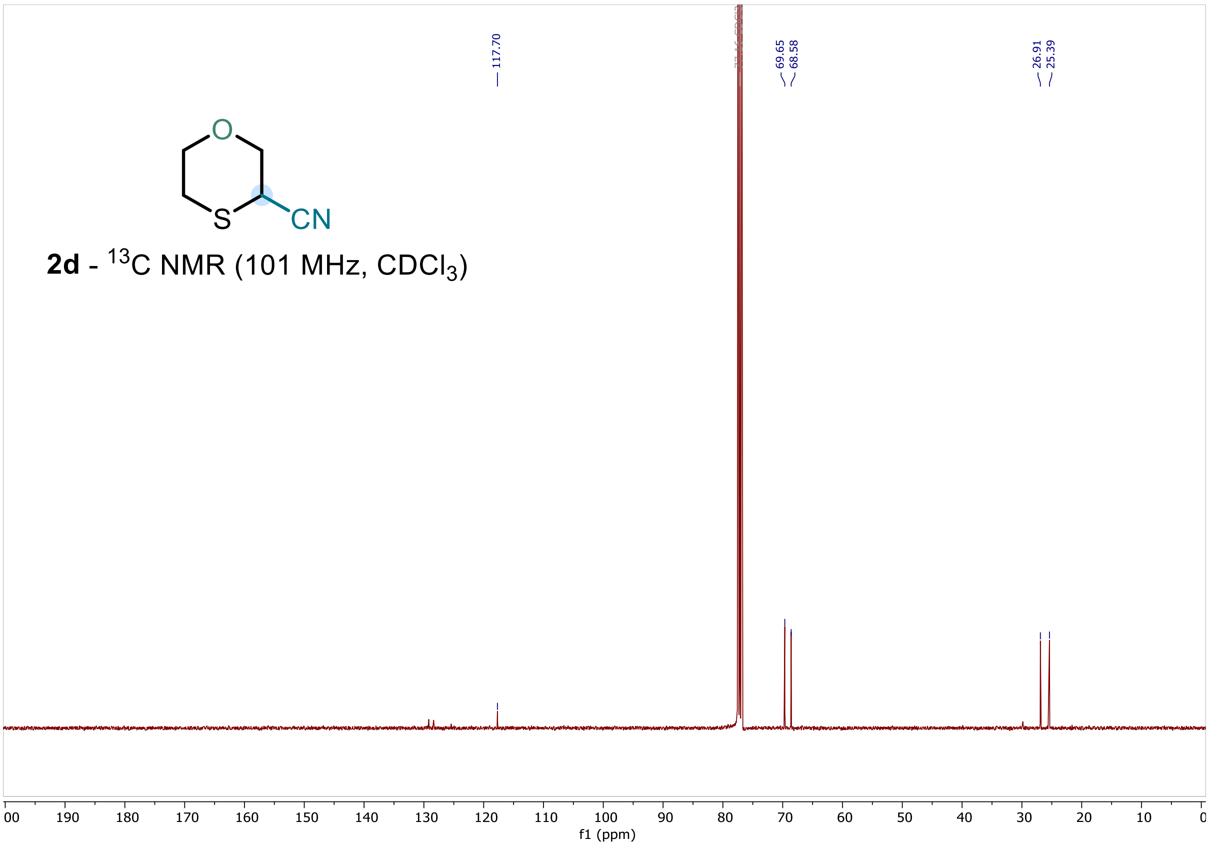
2d - 13C NMR (101 MHz, CDCl3)
117.70
69.65
68.58
26.91
25.39
f1 (ppm)

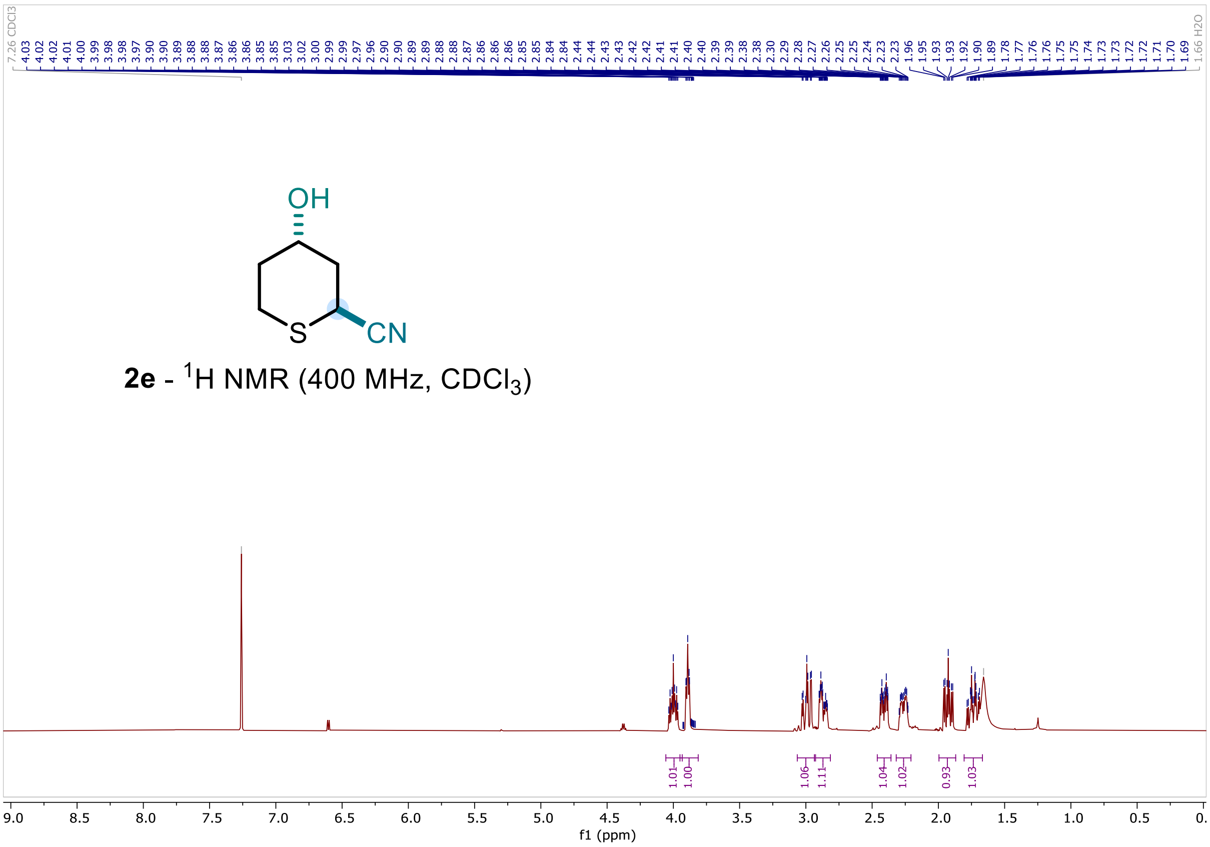
OH
S
CN
2e - 1H NMR (400 MHz, CDCl3)
f1 (ppm)

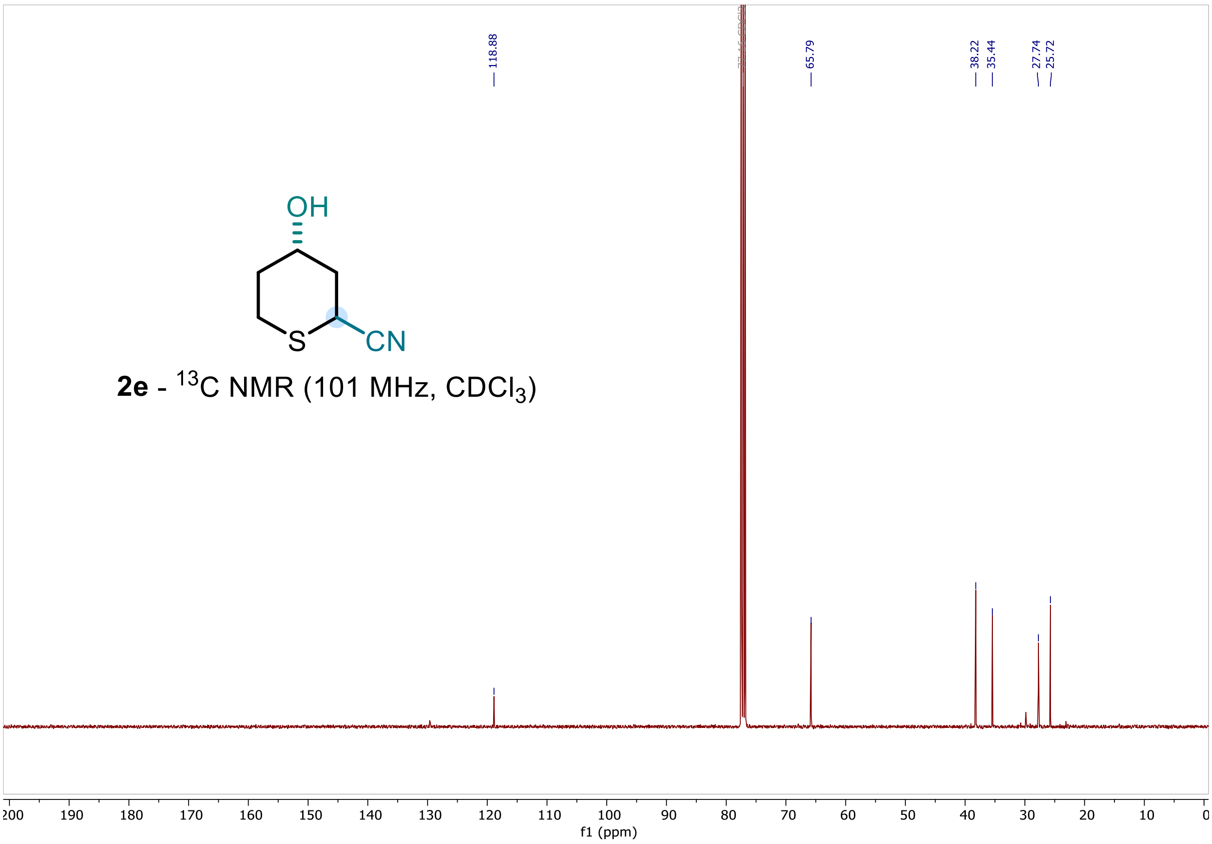
118.88
65.79
38.22
35.44
27.74
25.72
OH
S
CN
2e - 13C NMR (101 MHz, CDCl3)
f1 (ppm)

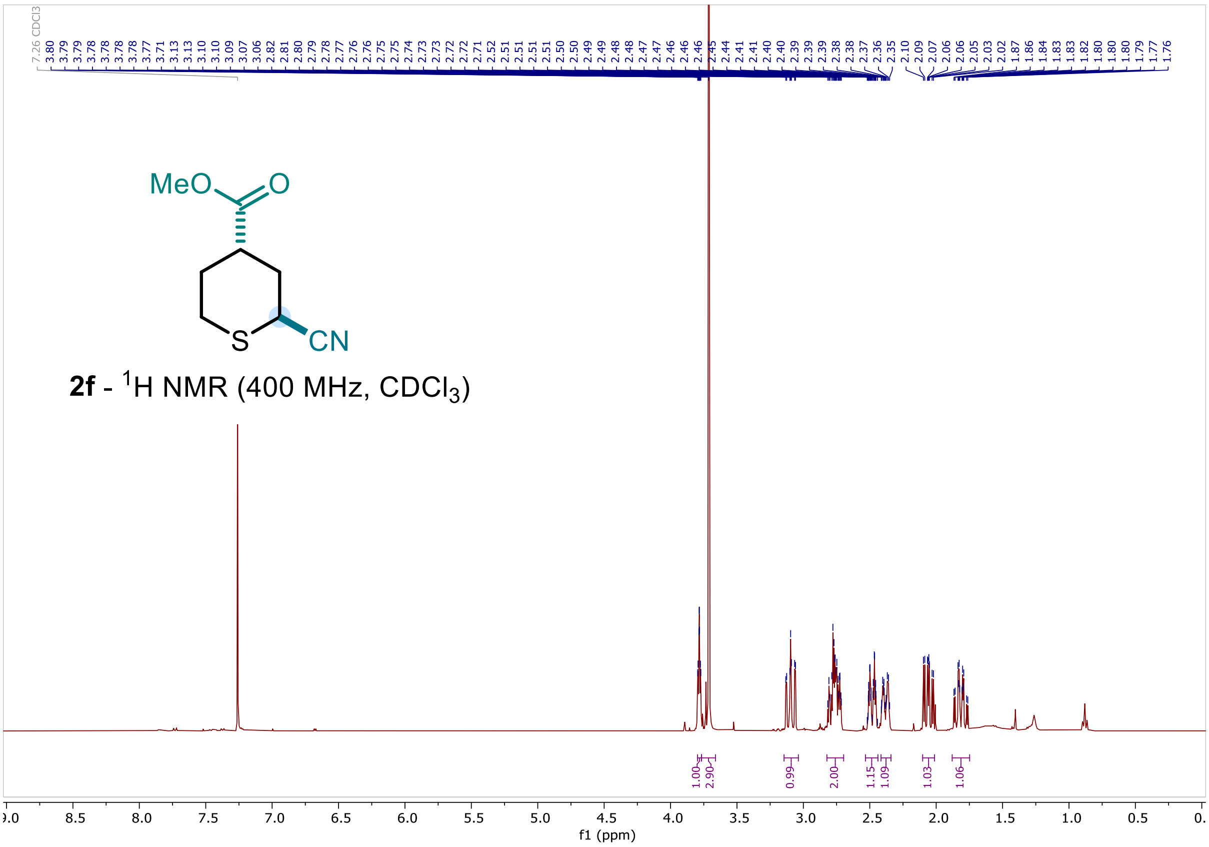
MeO
O
S
CN
2f - ¹H NMR (400 MHz, CDCl₃)
f1 (ppm)

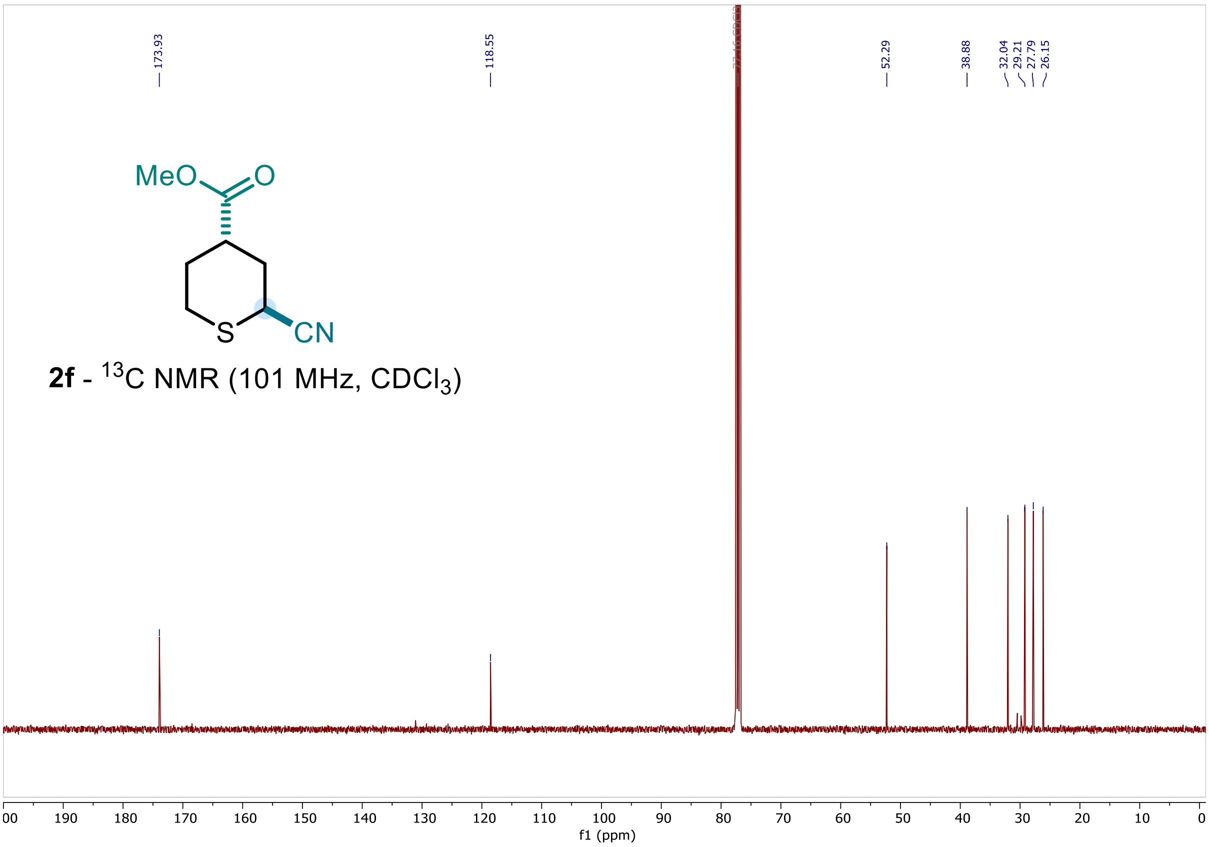
173.93
118.55
52.29
38.88
32.04
29.21
27.79
26.15
MeO
O
S
CN
2f - ¹³C NMR (101 MHz, CDCl₃)
f1 (ppm)

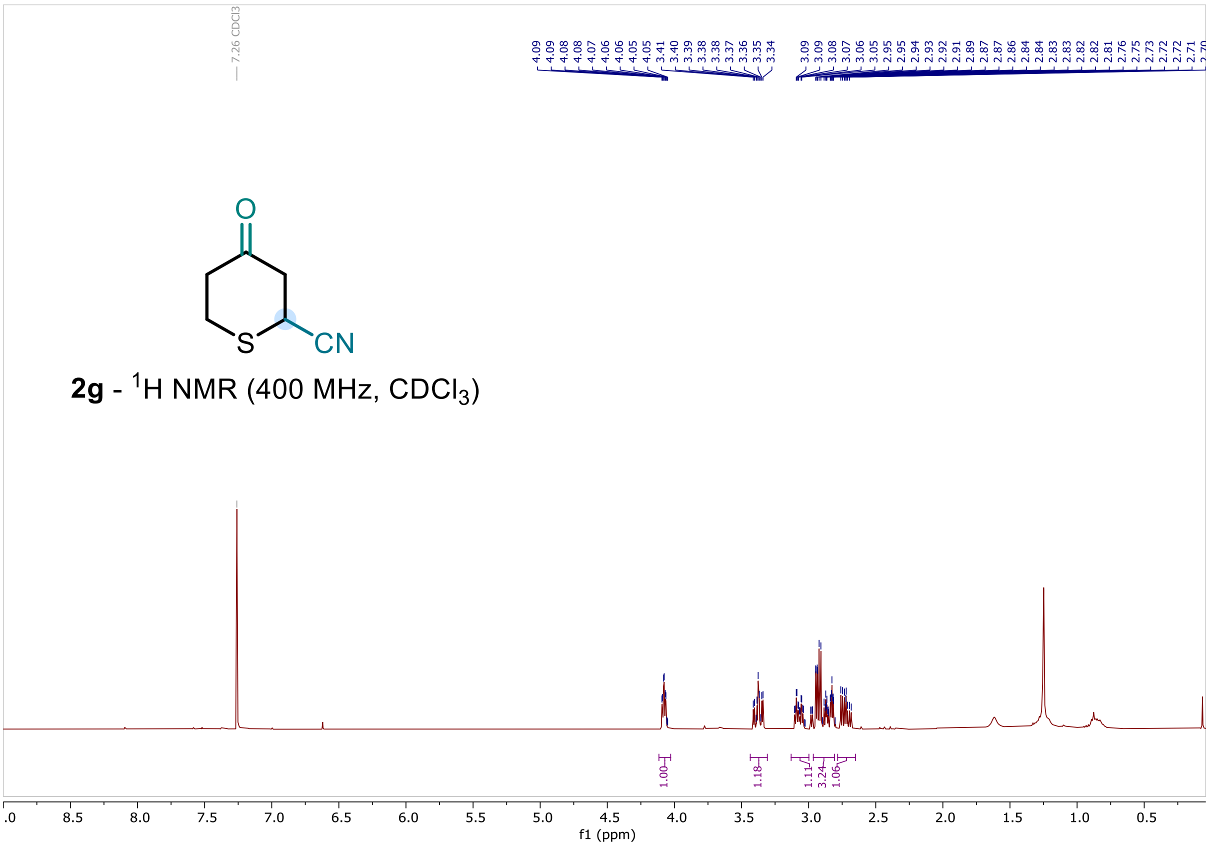
O
S
CN
2g - 1H NMR (400 MHz, CDCl3)
7.26 CDCl3
f1 (ppm)

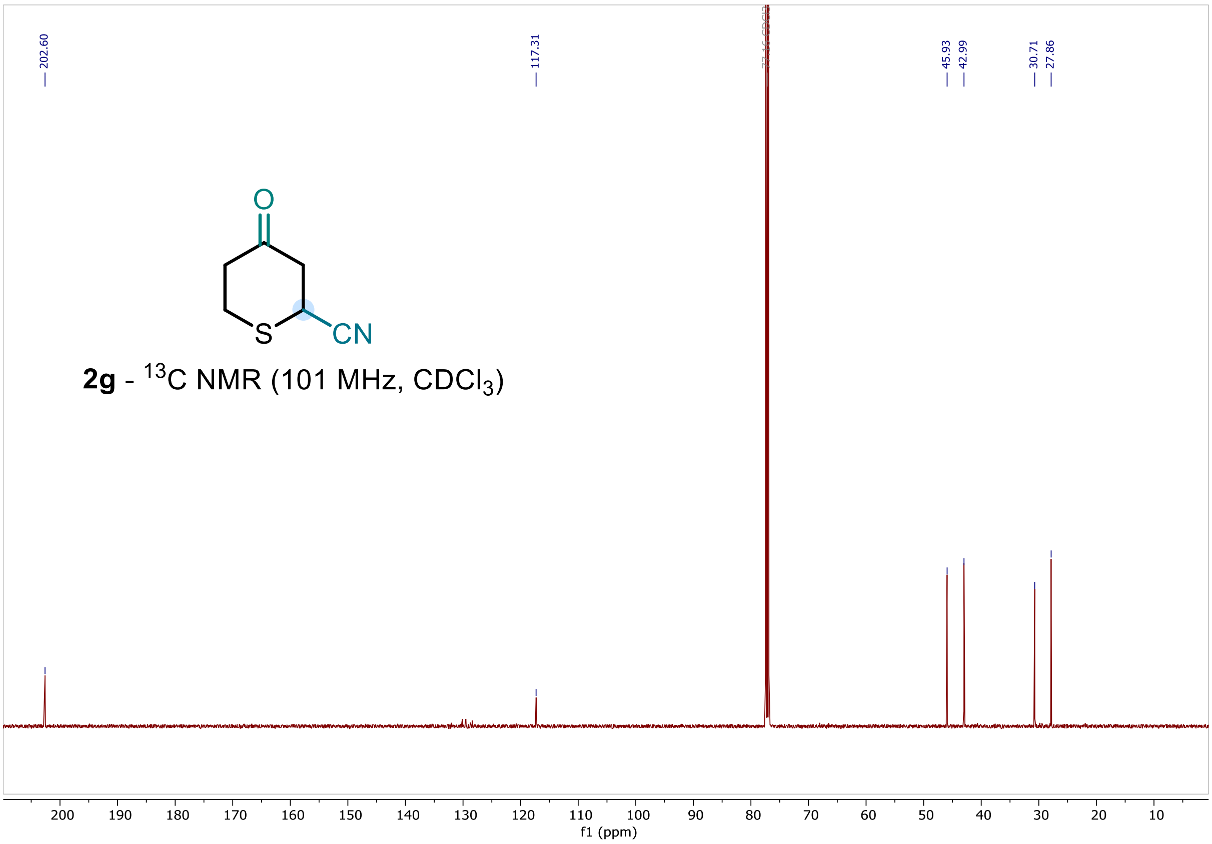
O
S
CN
2g - 13C NMR (101 MHz, CDCl3)
202.60
117.31
45.93
42.99
30.71
27.86
f1 (ppm)

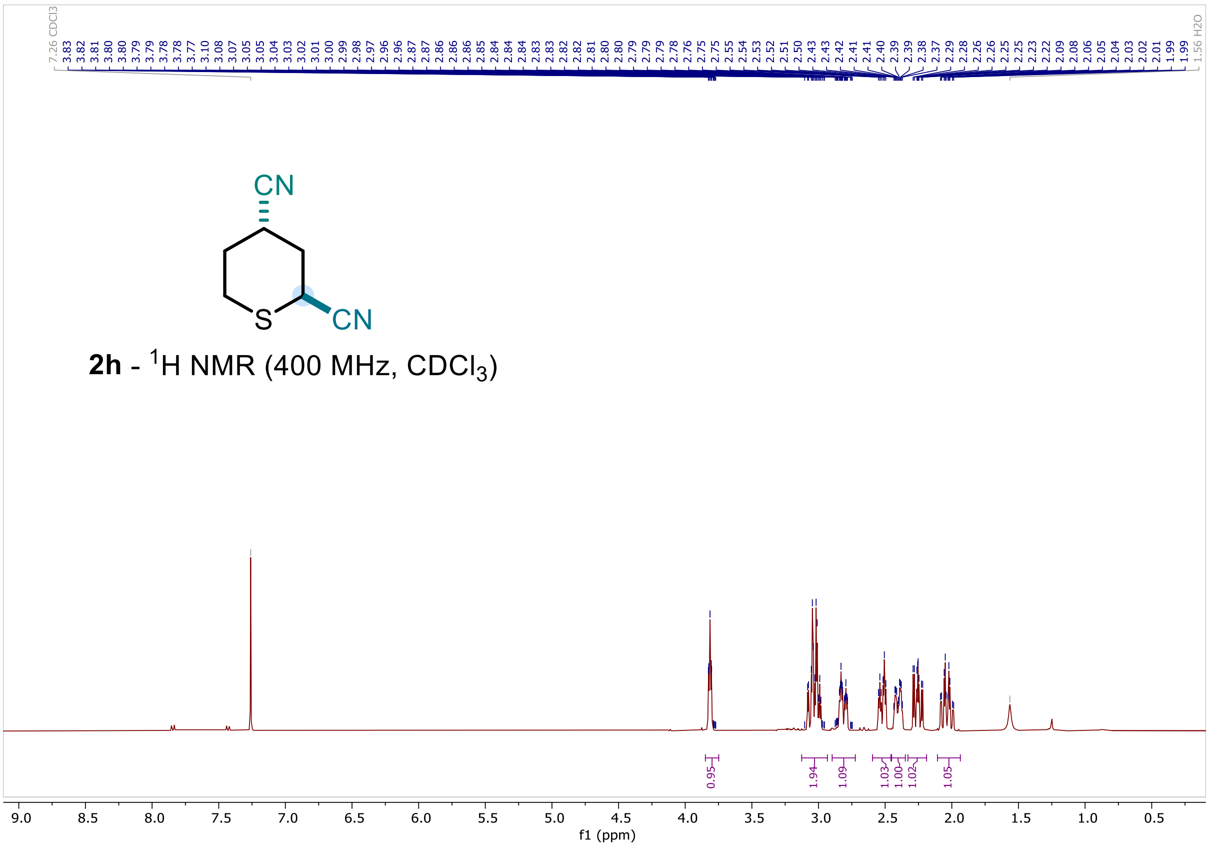
7.26 CDCl3
3.83 3.82 3.81 3.80 3.80 3.79 3.79 3.78 3.78 3.77 3.10 3.08 3.07 3.05 3.05 3.04 3.03 3.02 3.01 3.00 2.99 2.98 2.97 2.96 2.96 2.87 2.87 2.86 2.86 2.86 2.85 2.84 2.84 2.84 2.83 2.83 2.82 2.82 2.81 2.80 2.80 2.79 2.79 2.79 2.78 2.76 2.75 2.75 2.55 2.54 2.53 2.52 2.51 2.50 2.43 2.43 2.42 2.41 2.40 2.39 2.39 2.38 2.37 2.29 2.28 2.26 2.26 2.25 2.25 2.23 2.22 2.09 2.08 2.06 2.05 2.04 2.03 2.02 2.01 1.99 1.99
1.56 H2O
CN
S
CN
2h - 1H NMR (400 MHz, CDCl3)
0.95
1.94
1.09
1.03
1.00
1.02
1.05
9.0 8.5 8.0 7.5 7.0 6.5 6.0 5.5 5.0 4.5 4.0 3.5 3.0 2.5 2.0 1.5 1.0 0.5
f1 (ppm)

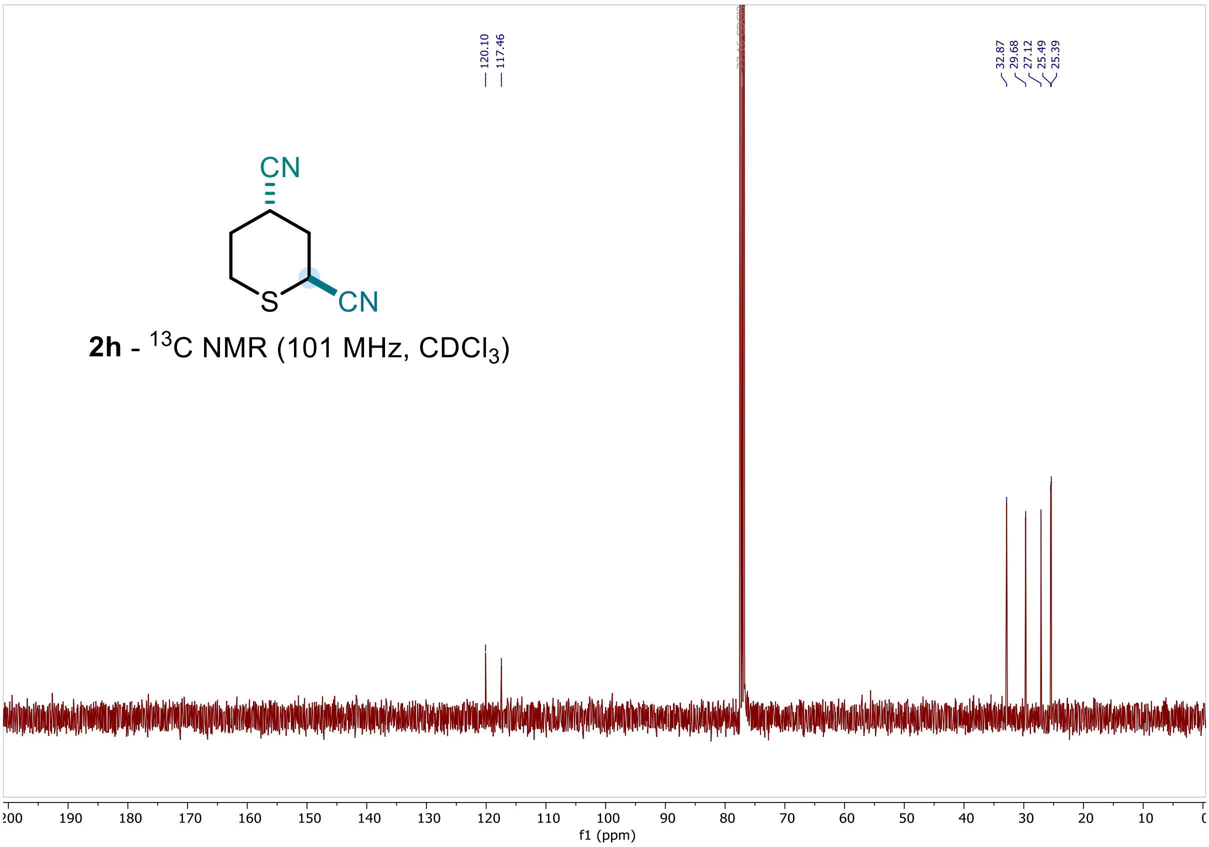
120.10
117.46
32.87
29.68
27.12
25.49
25.39
CN
S
CN
2h - 13C NMR (101 MHz, CDCl3)
200 190 180 170 160 150 140 130 120 110 100 90 80 70 60 50 40 30 20 10 0
f1 (ppm)

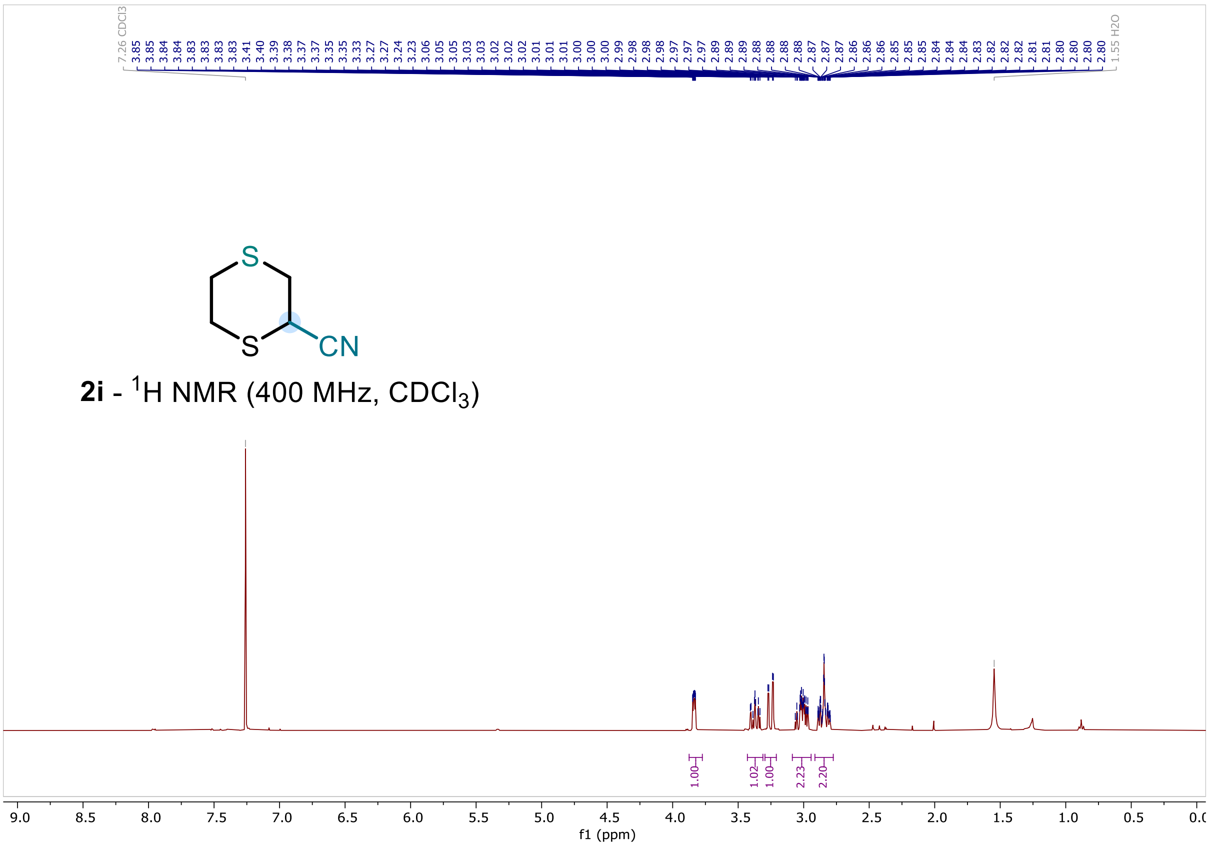
2i - ^{1}H NMR (400 MHz, $CDCl_3$)
f1 (ppm)

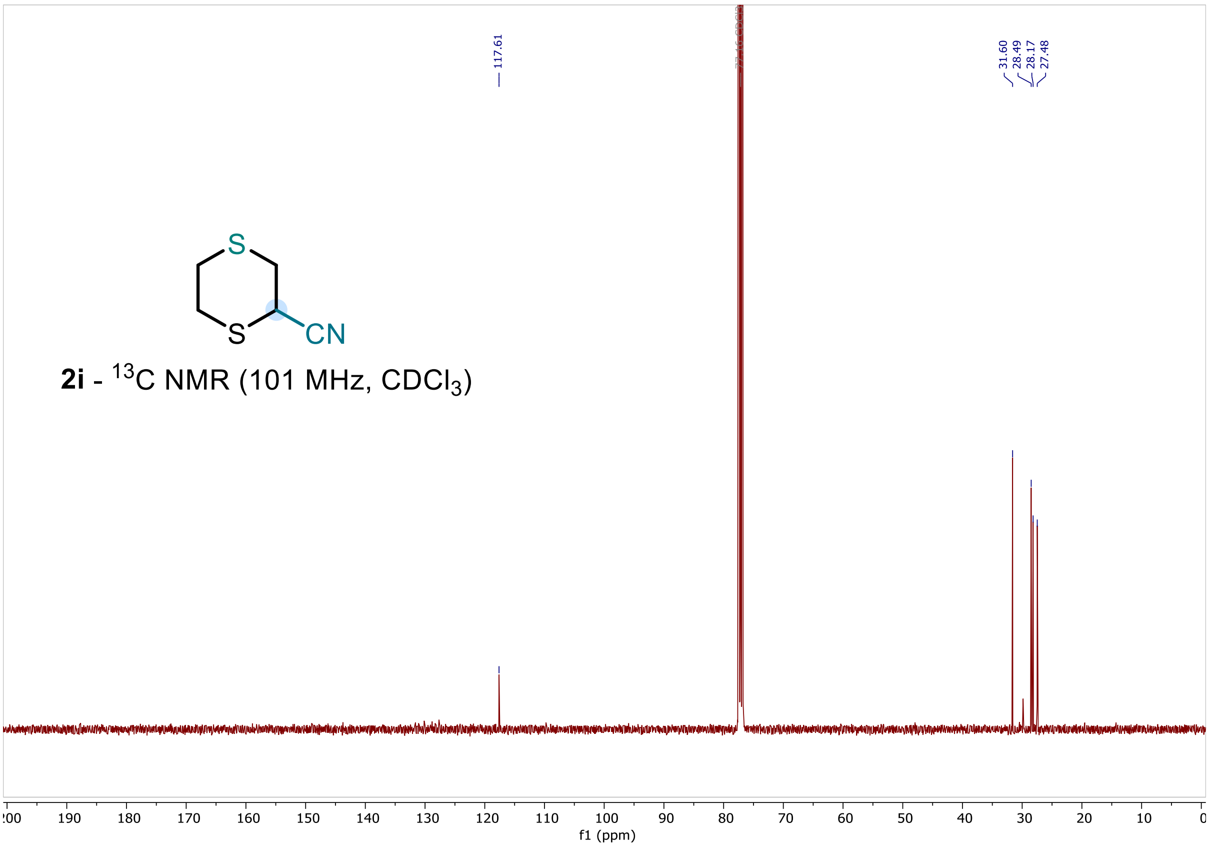
2i - ^{13}C NMR (101 MHz, $CDCl_3$)
117.61
31.60
28.49
28.17
27.48
f1 (ppm)

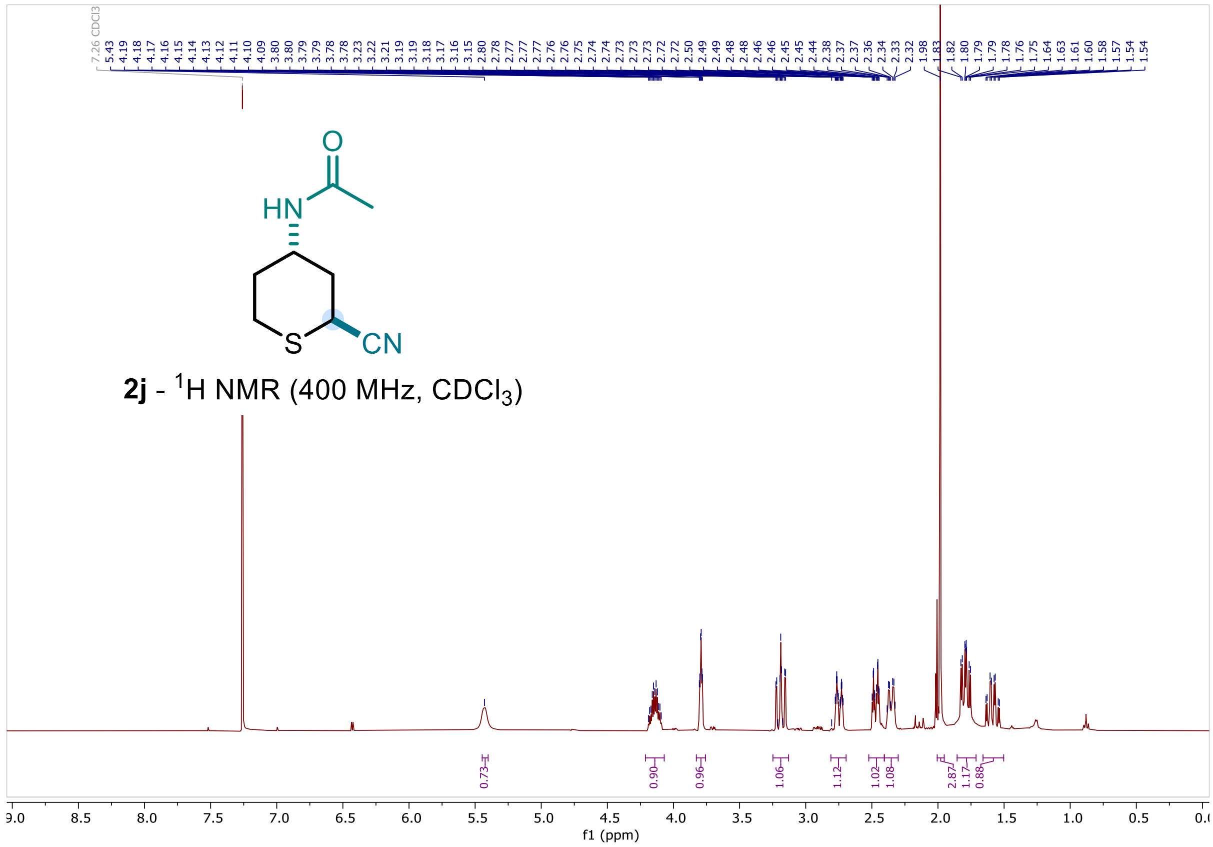

2j - ^{1}H NMR (400 MHz, CDCl$_3$)
HN
O
S
CN
7.26 CDCl3
5.43
0.73
0.90
0.96
1.06
1.12
1.02
1.08
2.87
1.17
0.88
f1 (ppm)

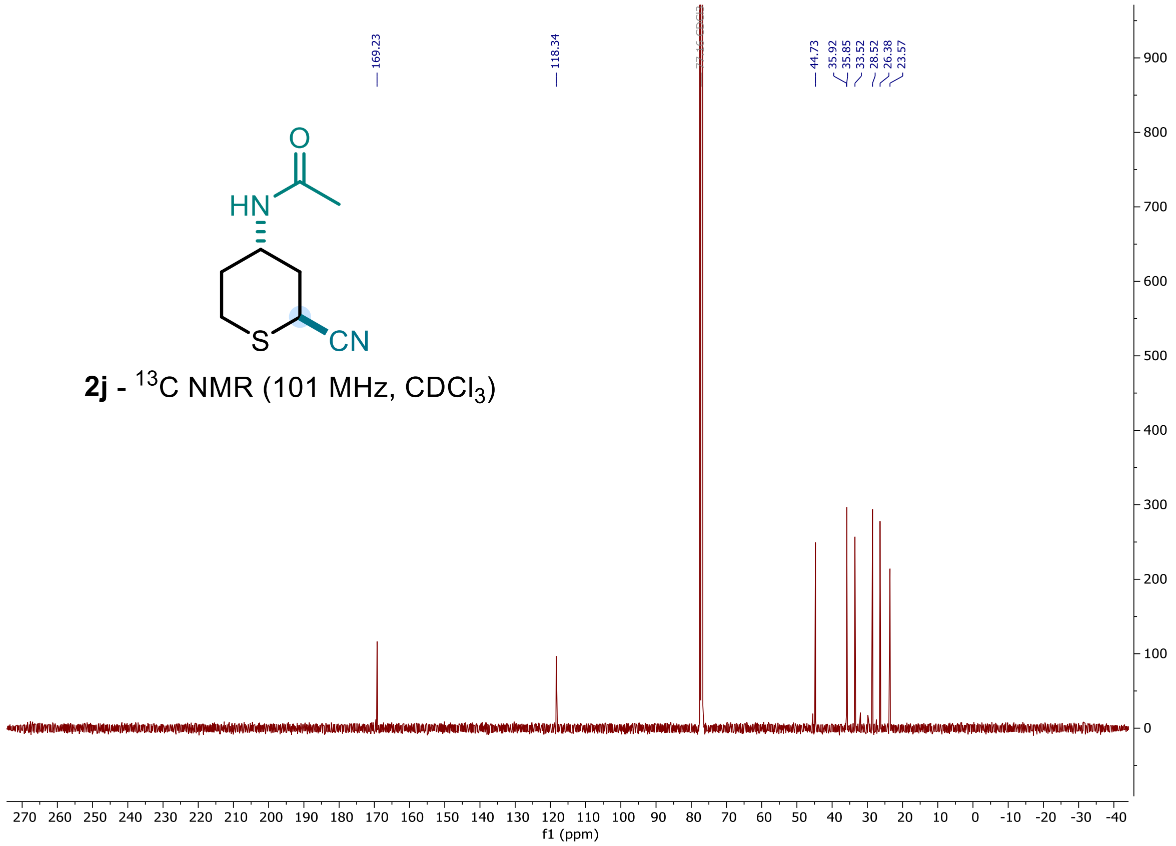

2j - ^{13}C NMR (101 MHz, CDCl$_3$)
HN
O
S
CN
169.23
118.34
44.73
35.92
35.85
33.52
28.52
26.38
23.57
f1 (ppm)

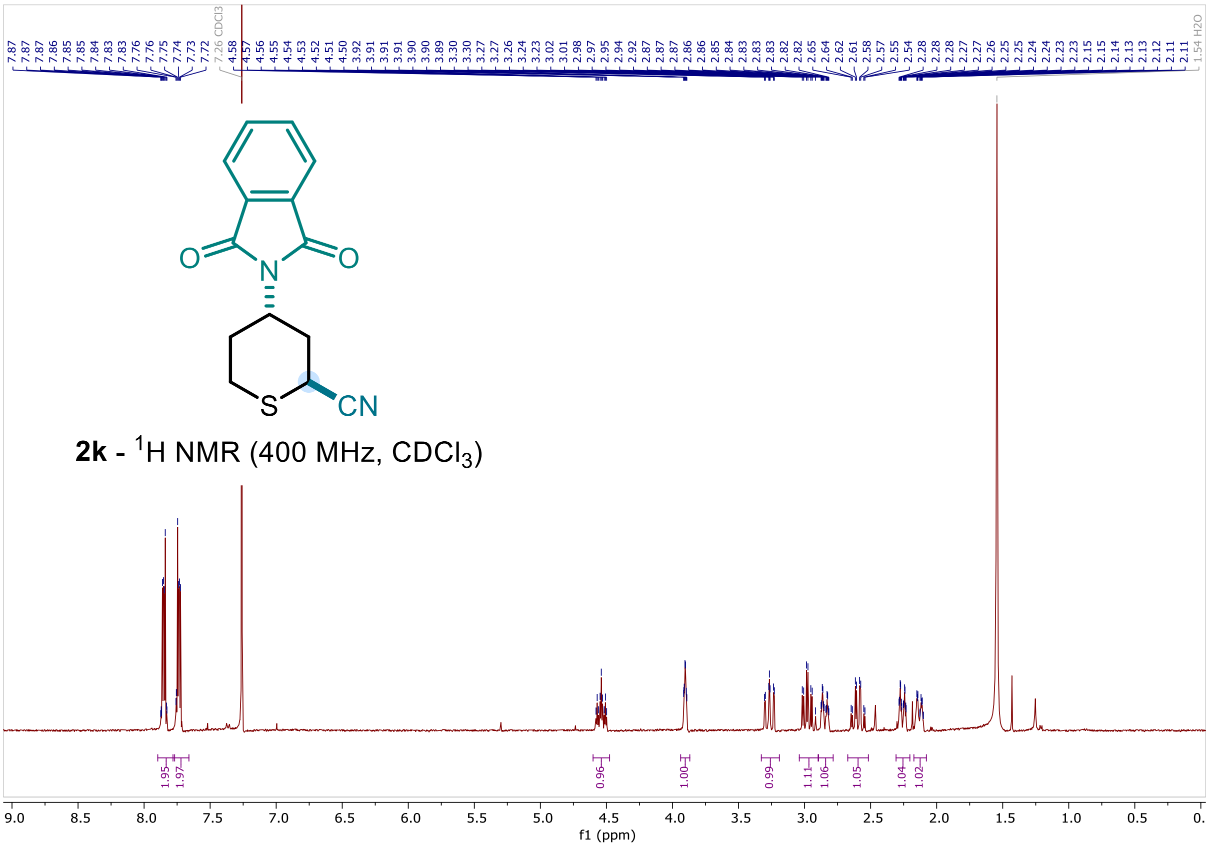

2k - ^{1}H NMR (400 MHz, $CDCl_3$)

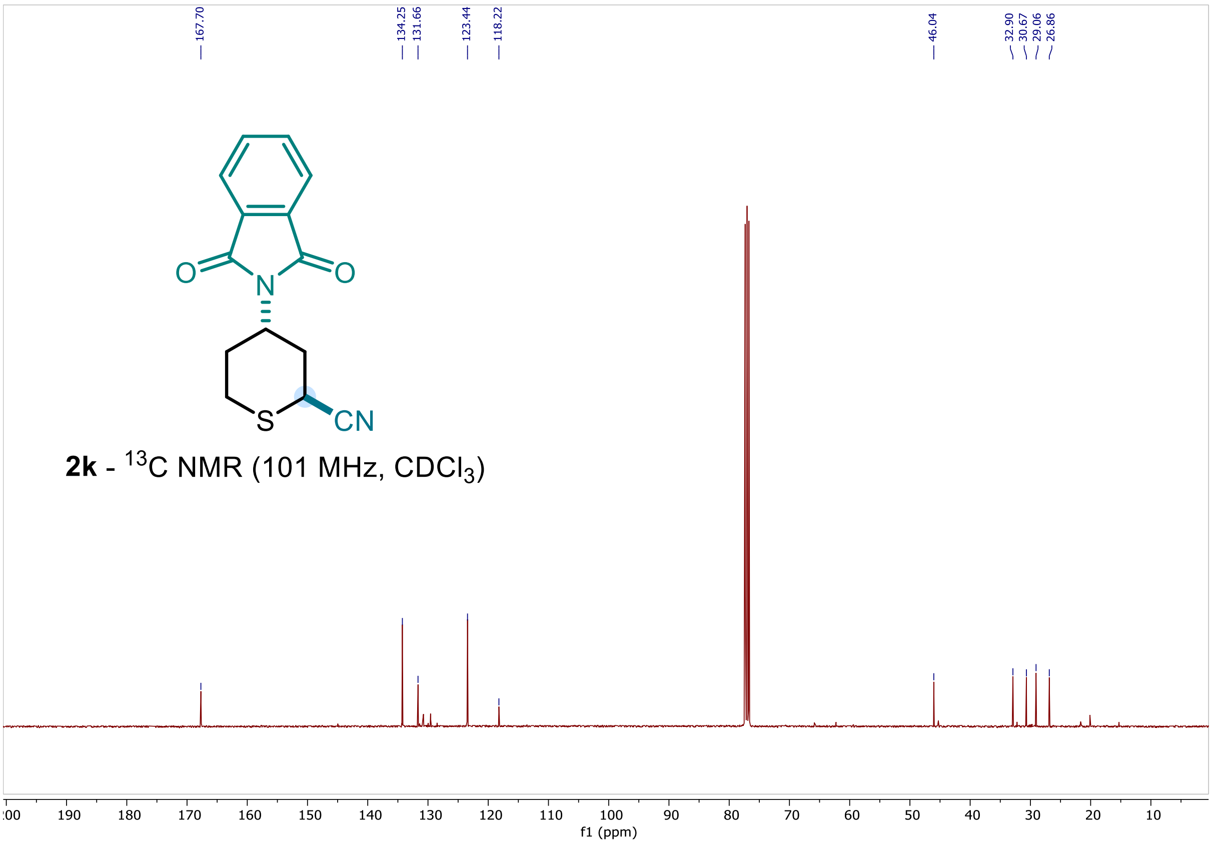

2k - ^{13}C NMR (101 MHz, $CDCl_3$)

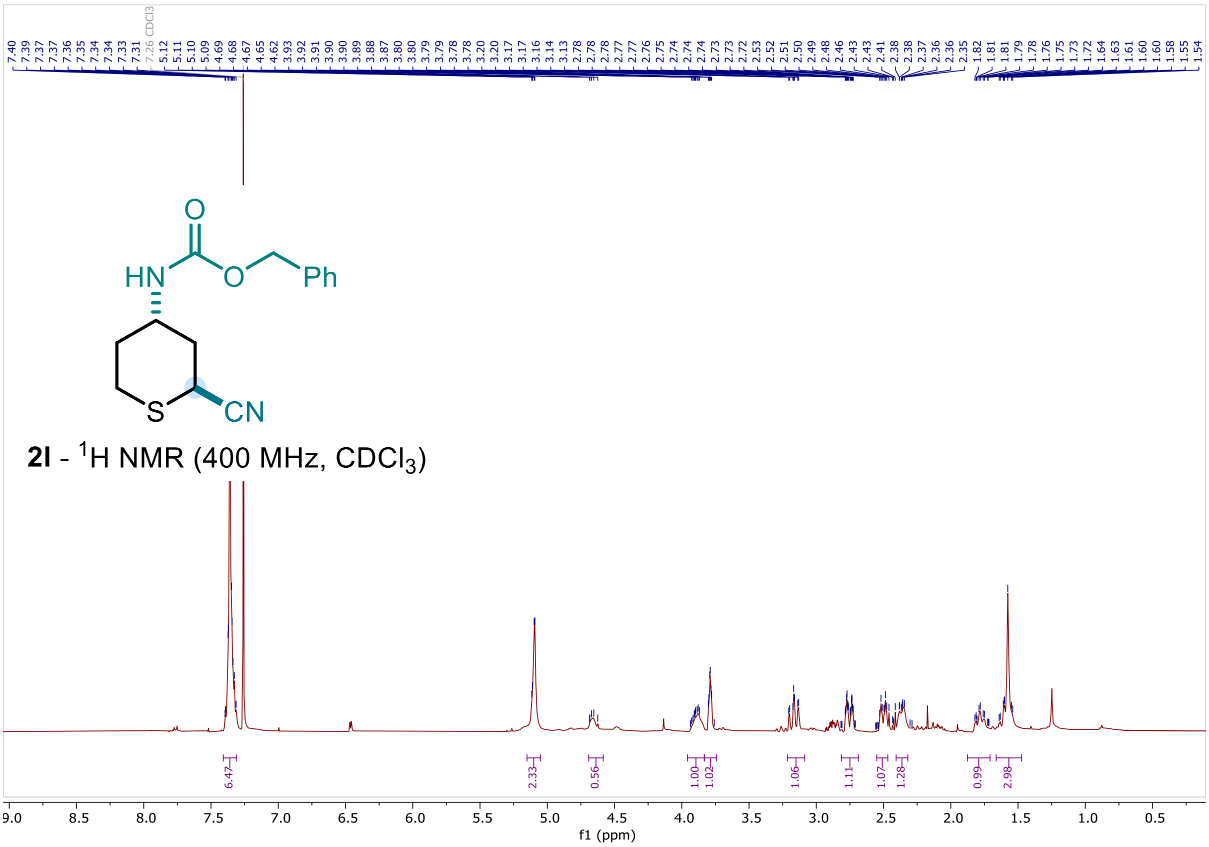

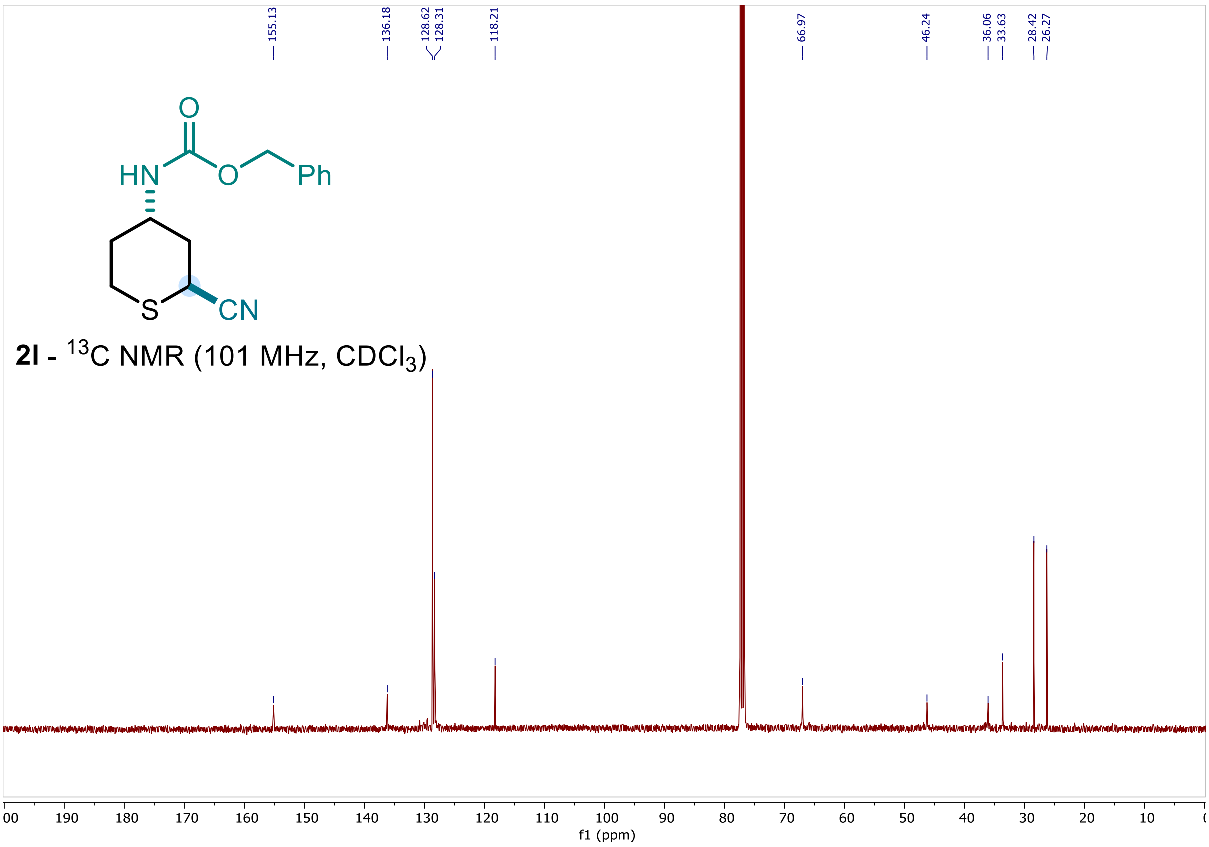

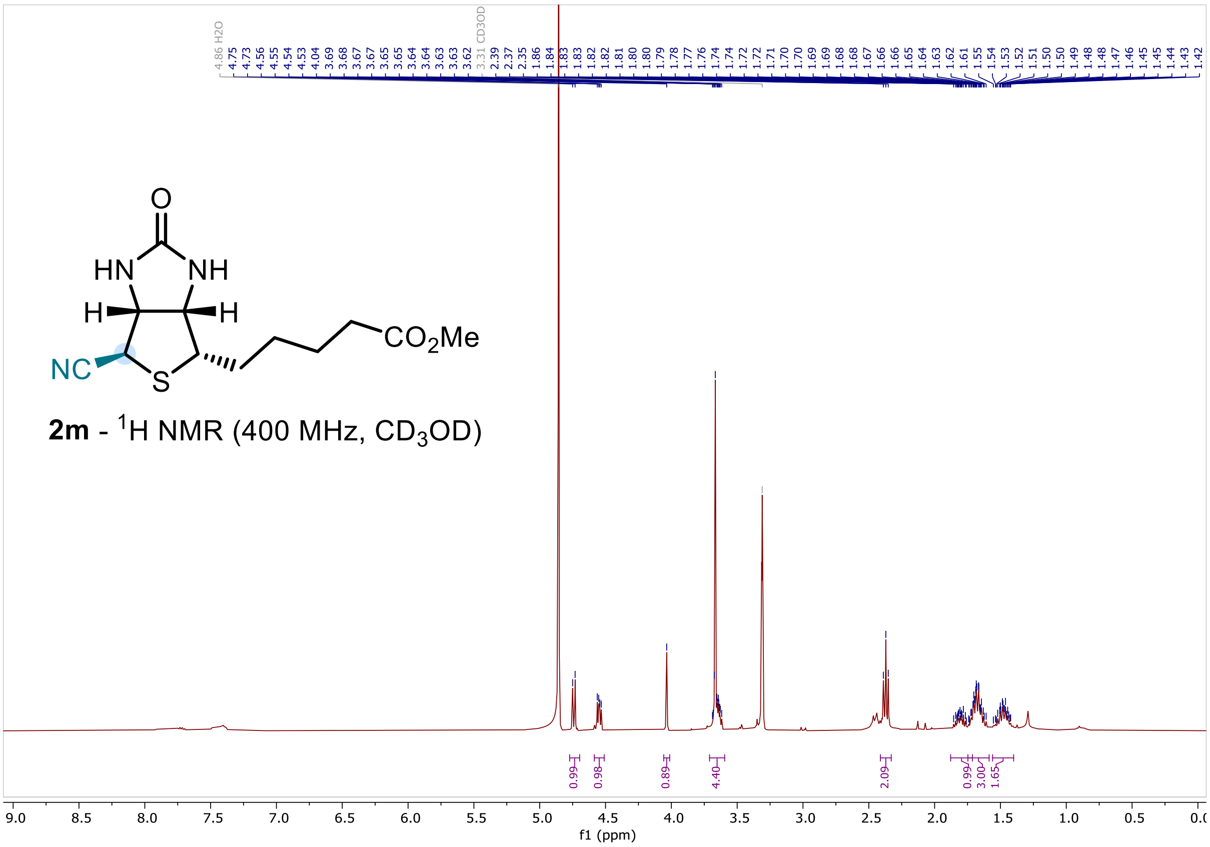

O
HN
NH
H
H
NC
S
CO2Me
2m - 1H NMR (400 MHz, CD3OD)
4.86 H2O
3.31 CD3OD
0.99
0.98
0.89
4.40
2.09
0.99
3.00
1.65
f1 (ppm)

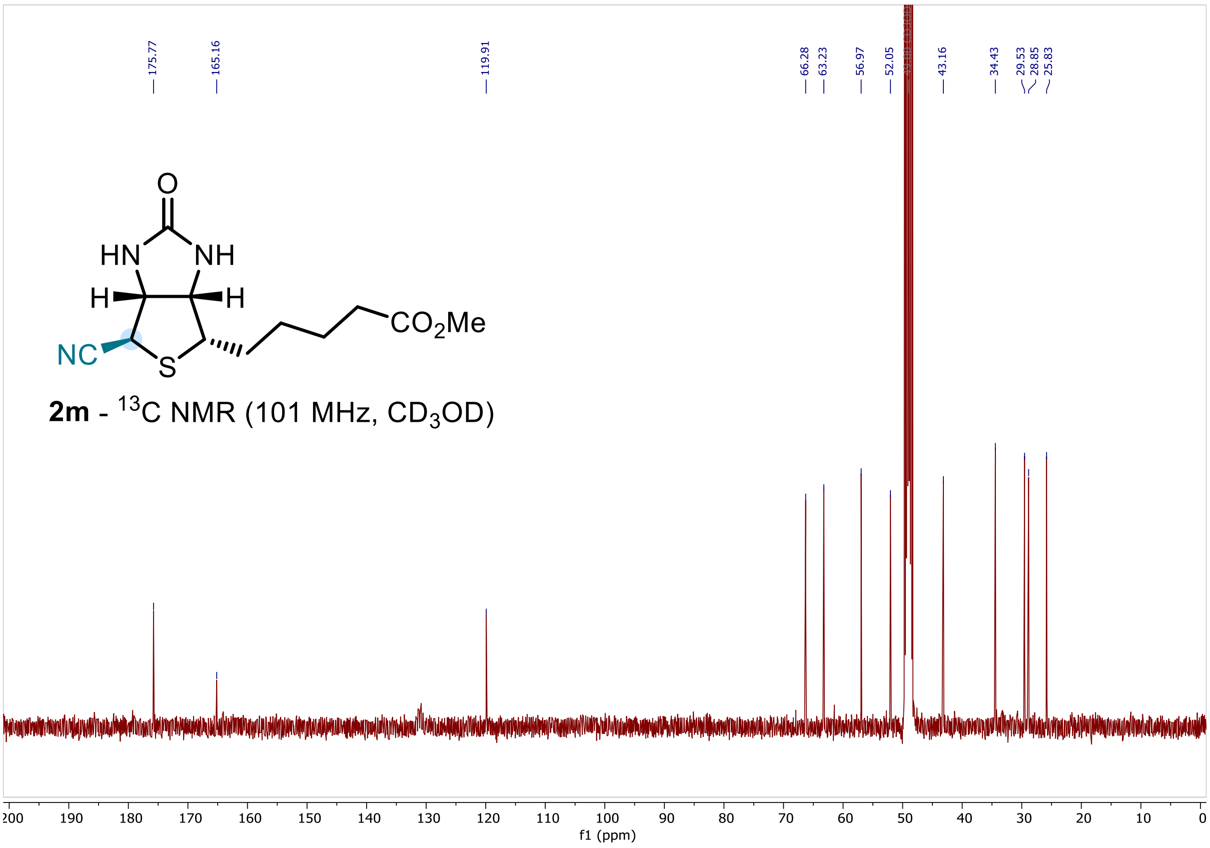

175.77
165.16
119.91
66.28
63.23
56.97
52.05
43.16
34.43
29.53
28.85
25.83
O
HN
NH
H
H
NC
S
CO2Me
2m - 13C NMR (101 MHz, CD3OD)
f1 (ppm)

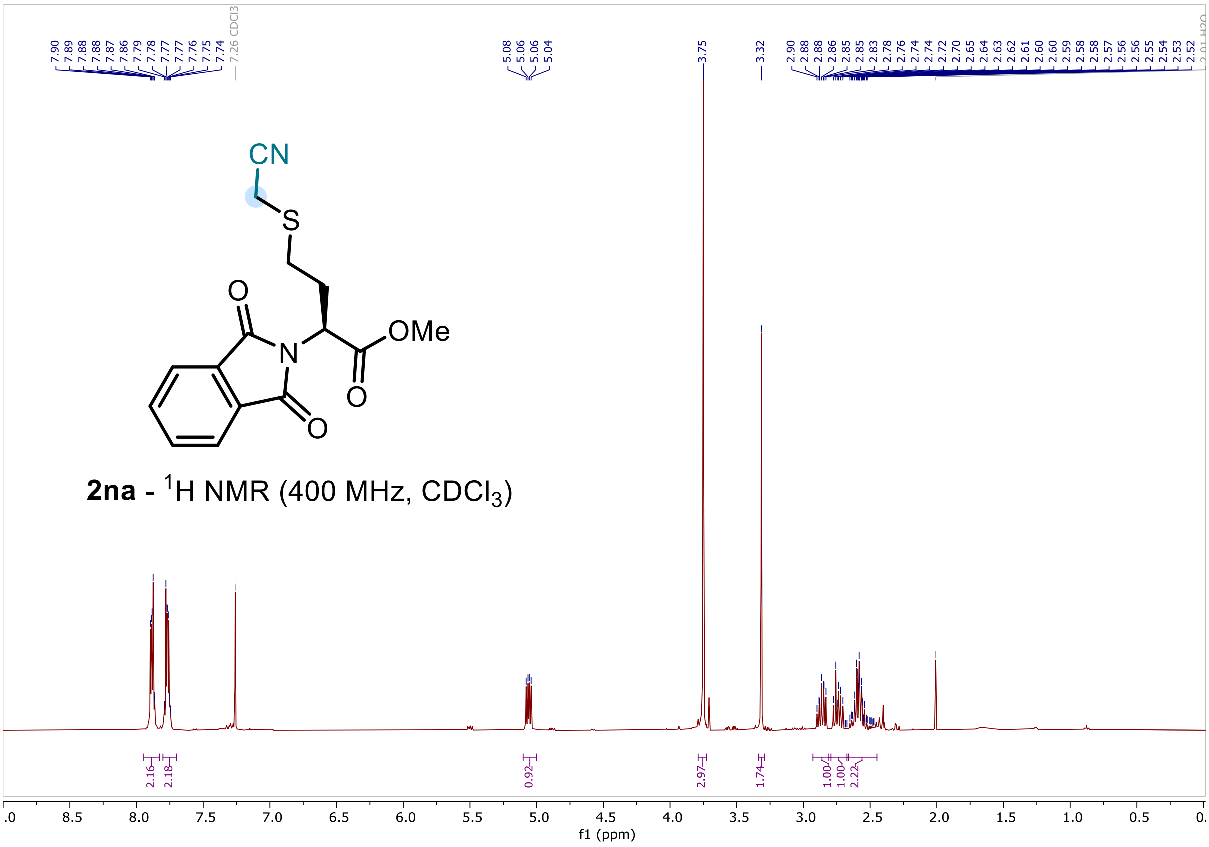

2na - ^{1}H NMR (400 MHz, $CDCl_3$)

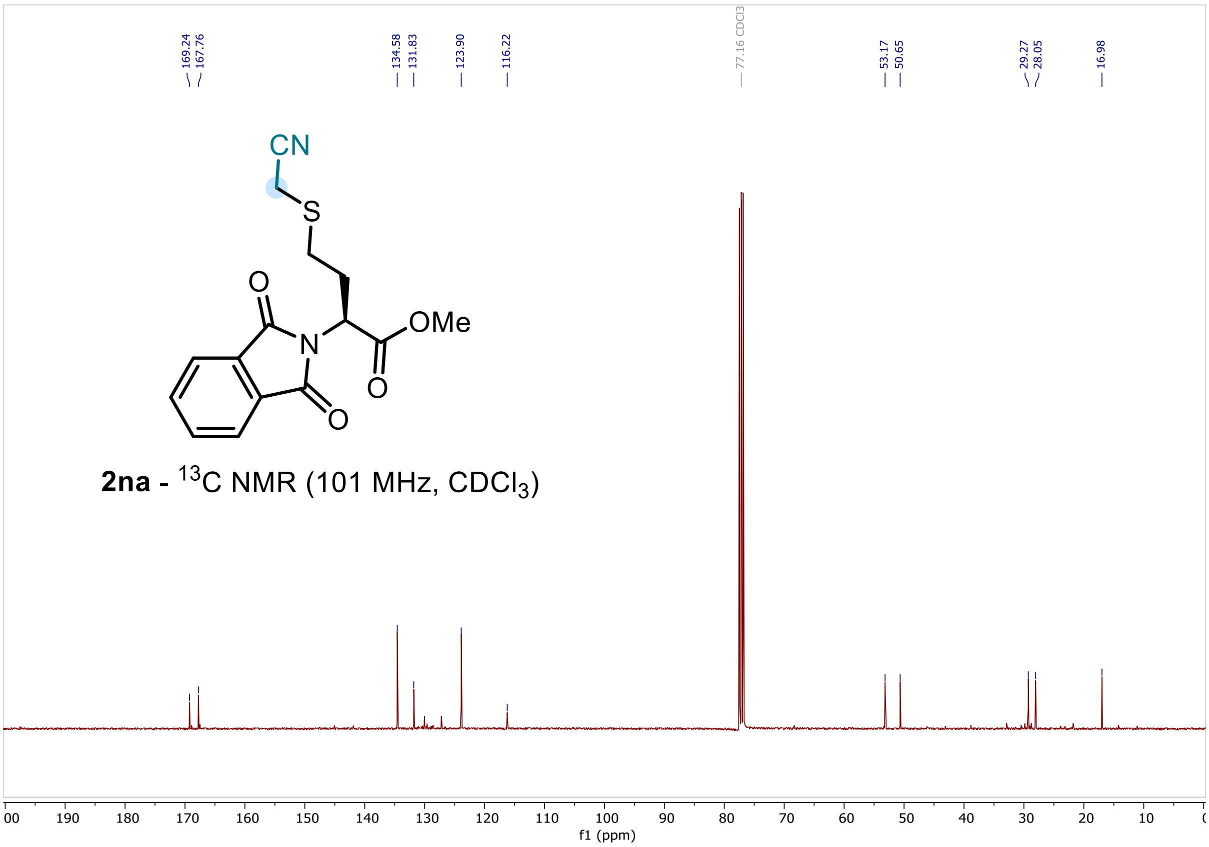

2na - ^{13}C NMR (101 MHz, $CDCl_3$)

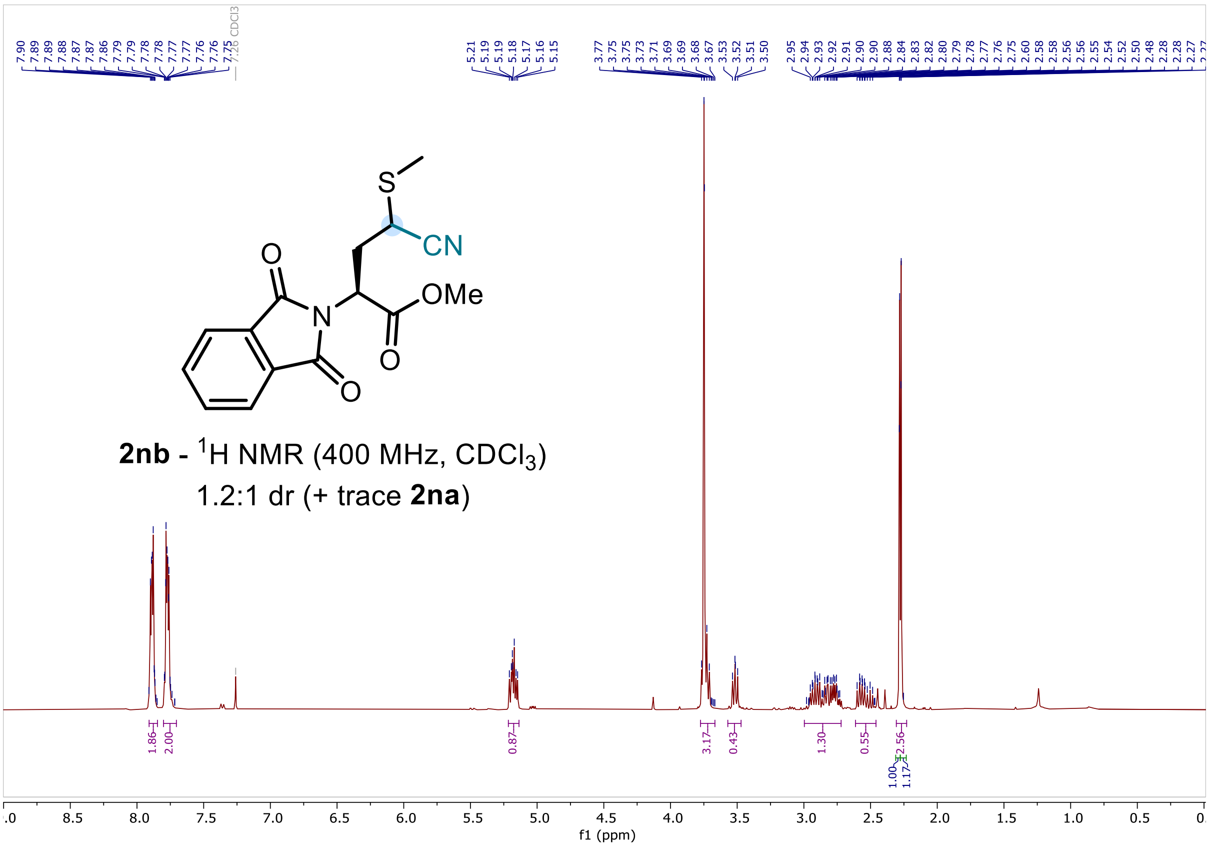
7.90 7.89 7.89 7.88 7.87 7.87 7.86 7.79 7.79 7.78 7.78 7.77 7.77 7.76 7.76 7.75 7.26 CDCl3
5.21 5.19 5.19 5.18 5.17 5.16 5.15
3.77 3.75 3.75 3.73 3.71 3.69 3.69 3.68 3.67 3.53 3.52 3.51 3.50
2.95 2.94 2.93 2.92 2.91 2.90 2.90 2.88 2.84 2.83 2.82 2.80 2.79 2.78 2.77 2.76 2.75 2.60 2.58 2.58 2.56 2.56 2.55 2.54 2.52 2.50 2.48 2.28 2.28 2.27 2.27
S
CN
O
N
OMe
O
O
2nb - ^{1}H NMR (400 MHz, $CDCl_3$)
1.2:1 dr (+ trace 2na)
1.86
2.00
0.87
3.17
0.43
1.30
0.55
2.56
1.00
1.17
f1 (ppm)

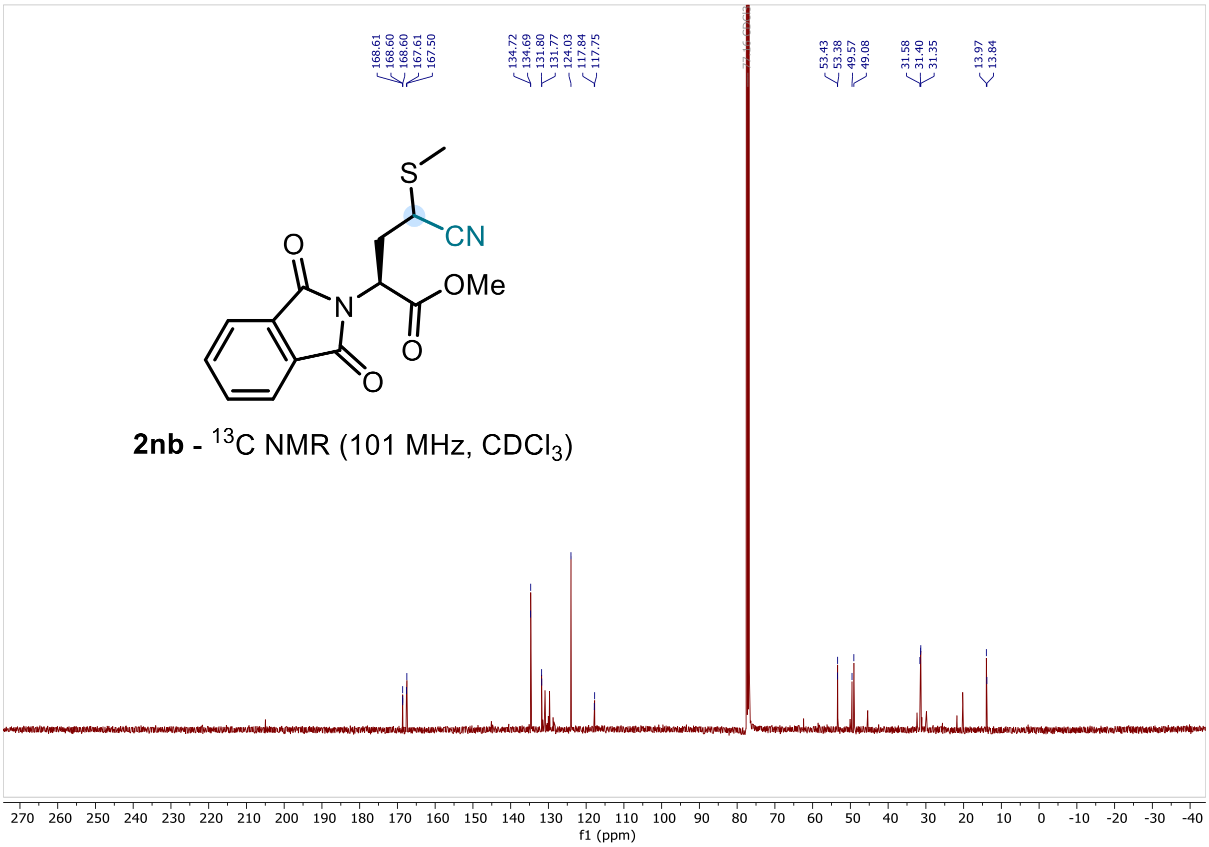
168.61 168.60 168.60 167.61 167.50
134.72 134.69 131.80 131.77 124.03 117.84 117.75
53.43 53.38 49.57 49.08
31.58 31.40 31.35
13.97 13.84
S
CN
O
N
OMe
O
O
2nb - ^{13}C NMR (101 MHz, $CDCl_3$)
f1 (ppm)

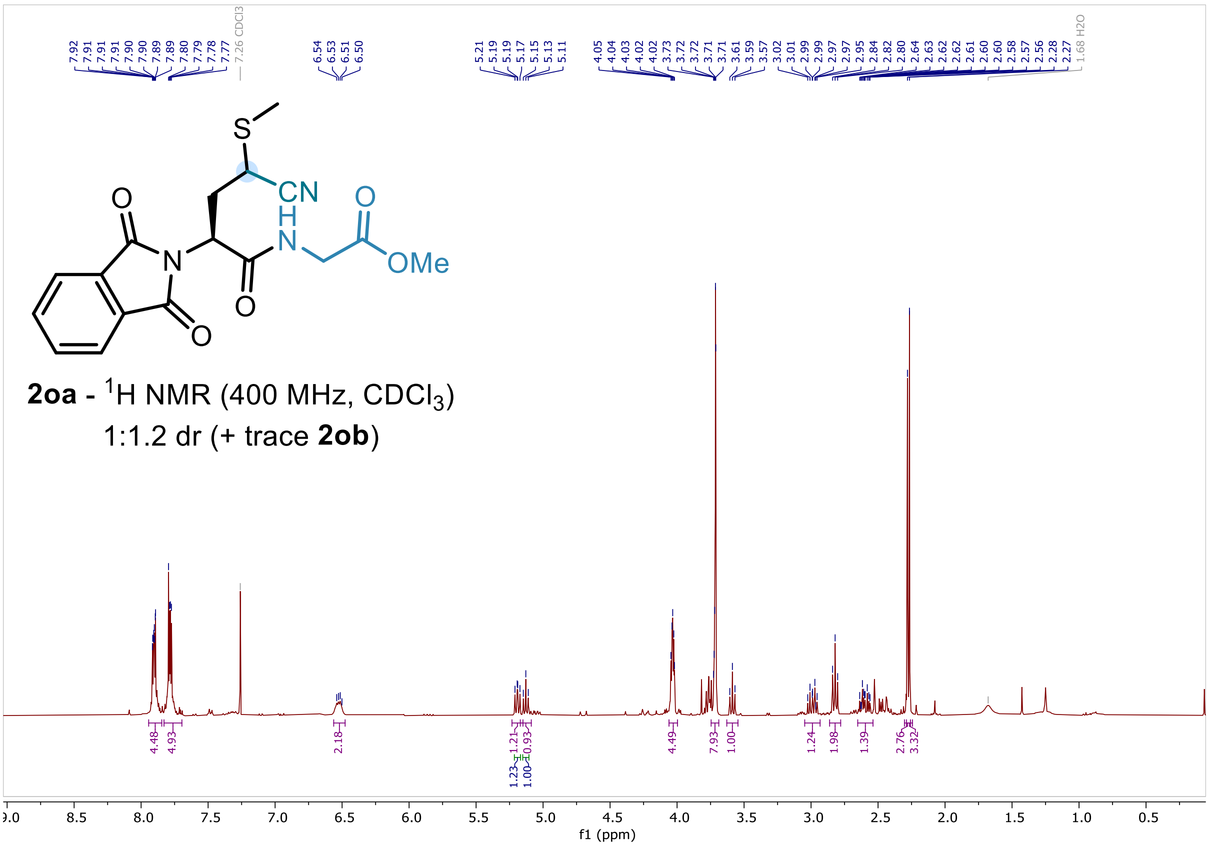

2oa - ^{1}H NMR (400 MHz, $CDCl_3$)
1:1.2 dr (+ trace 2ob)
f1 (ppm)

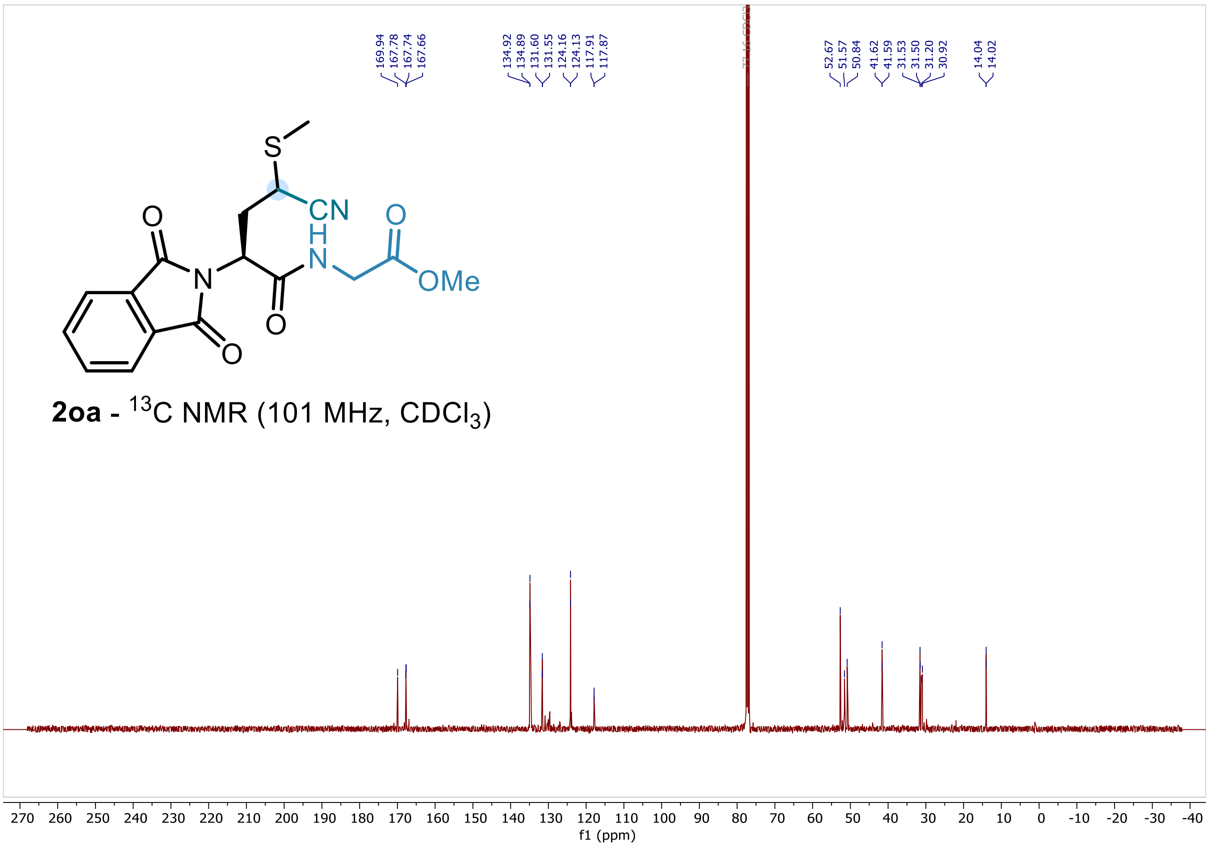

2oa - ^{13}C NMR (101 MHz, $CDCl_3$)
f1 (ppm)

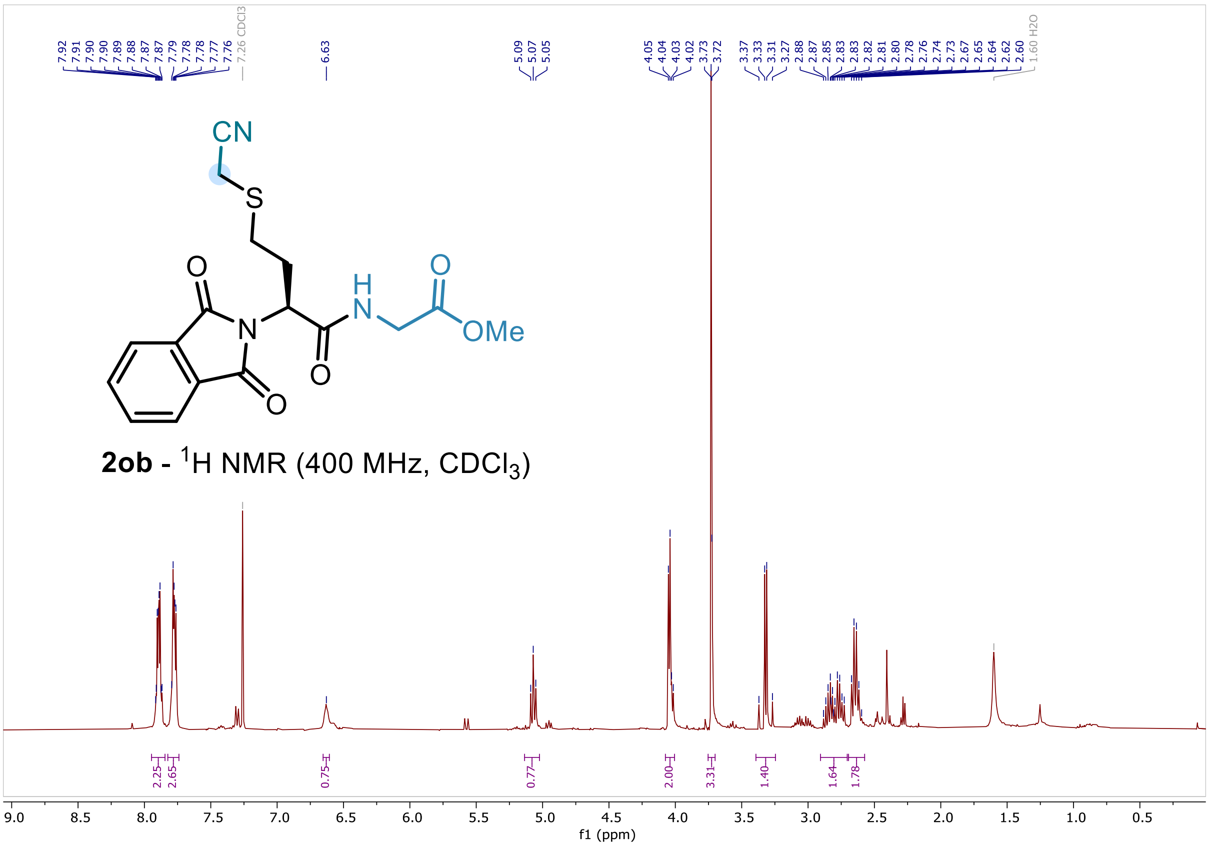

2ob - ^{1}H NMR (400 MHz, $CDCl_3$)

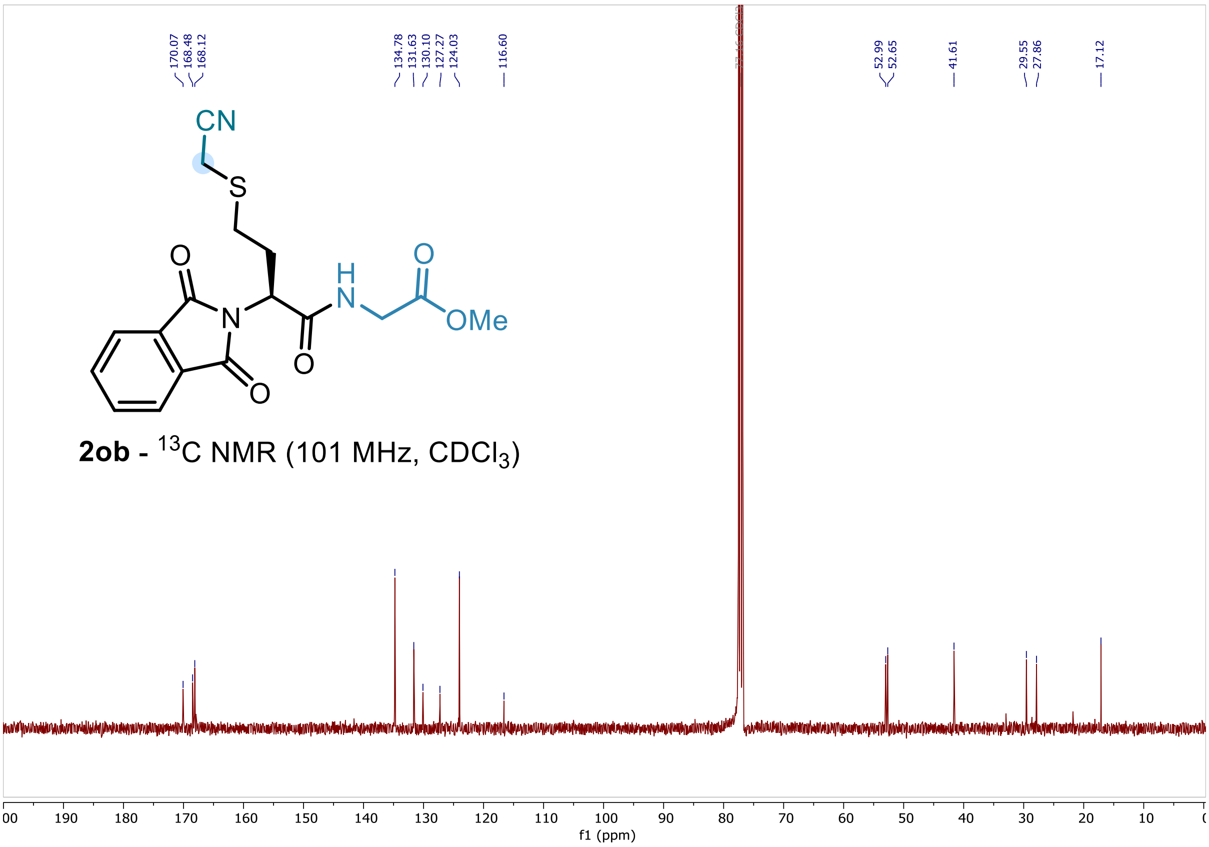

2ob - ^{13}C NMR (101 MHz, $CDCl_3$)

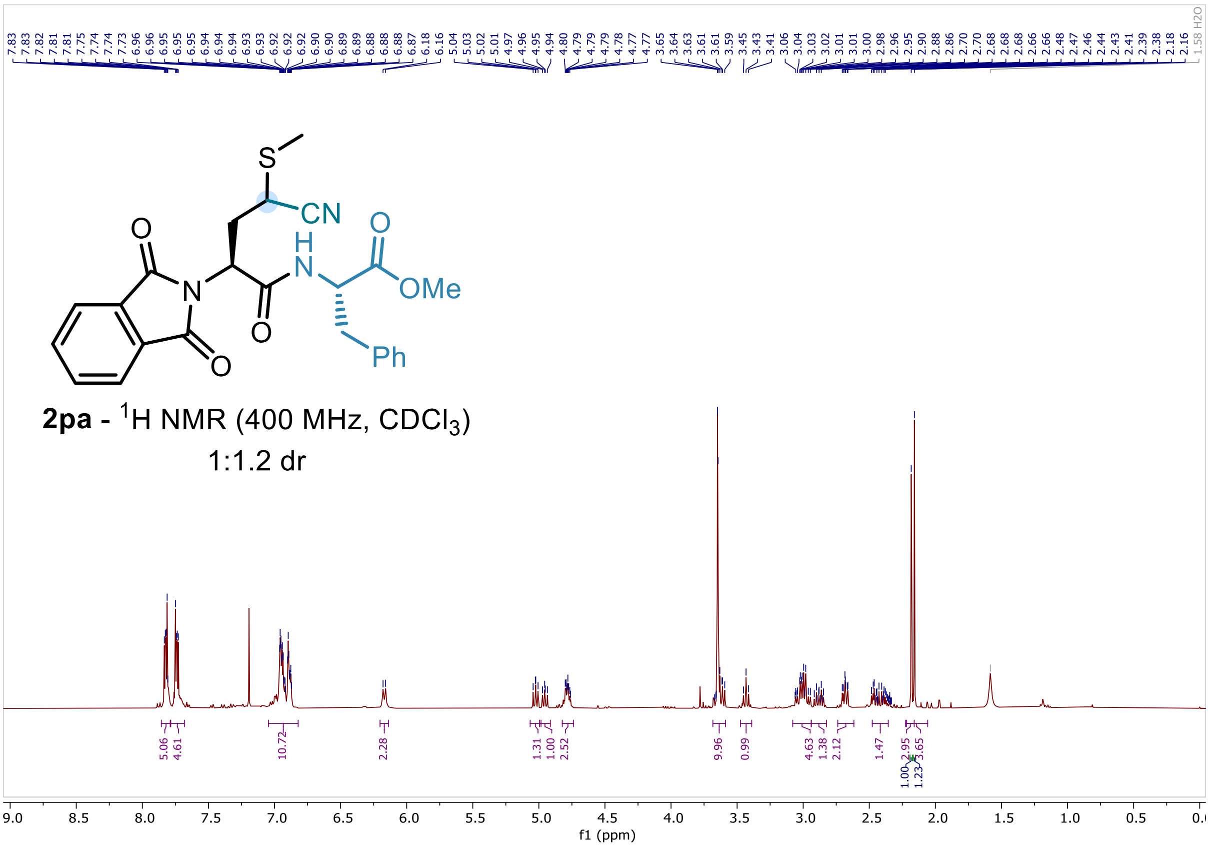
7.83 7.83 7.82 7.81 7.81 7.75 7.74 7.74 7.73 6.96 6.96 6.95 6.95 6.95 6.94 6.94 6.94 6.93 6.93 6.92 6.92 6.92 6.90 6.90 6.89 6.89 6.88 6.88 6.88 6.87 6.18 6.16 5.04 5.03 5.02 5.01 4.97 4.96 4.95 4.94 4.80 4.79 4.79 4.79 4.78 4.77 4.77 3.65 3.64 3.63 3.61 3.61 3.59 3.45 3.43 3.41 3.06 3.04 3.03 3.02 3.01 3.01 3.00 2.98 2.96 2.95 2.90 2.88 2.86 2.70 2.70 2.68 2.68 2.68 2.66 2.66 2.48 2.47 2.46 2.44 2.43 2.41 2.39 2.38 2.18 2.16 1.58 H2O
S
CN
O
H
N
O
N
OMe
O
O
Ph
2pa - ^{1}H NMR (400 MHz, $CDCl_3$)
1:1.2 dr
5.06 4.61 10.72 2.28 1.31 1.00 2.52 9.96 0.99 4.63 1.38 2.12 1.47 1.00 2.95 1.23 3.65
9.0 8.5 8.0 7.5 7.0 6.5 6.0 5.5 5.0 4.5 4.0 3.5 3.0 2.5 2.0 1.5 1.0 0.5
f1 (ppm)

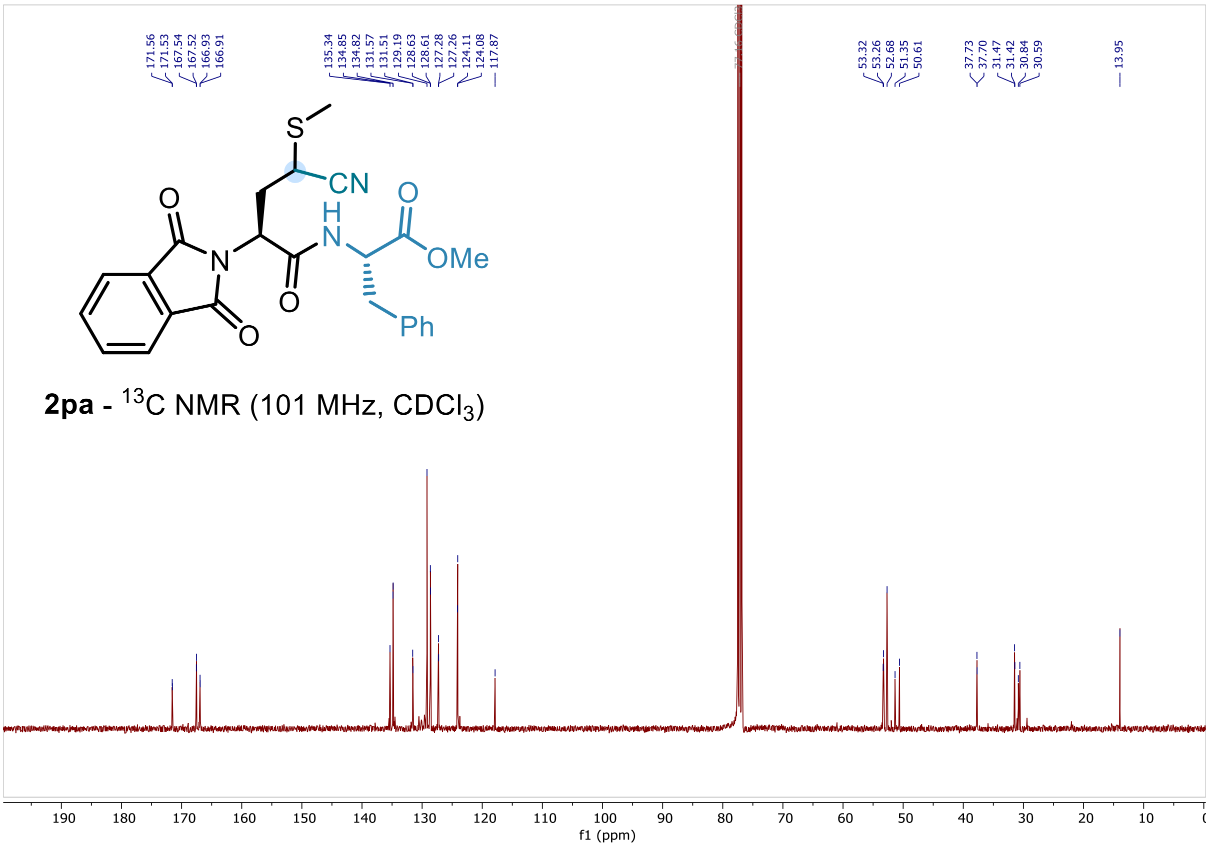
171.56 171.53 167.54 167.52 166.93 166.91
135.34 134.85 134.82 131.57 131.51 129.19 128.63 128.61 127.28 127.26 124.11 124.08 117.87
53.32 53.26 52.68 51.35 50.61
37.73 37.70 31.47 31.42 30.84 30.59
13.95
S
CN
O
H
N
O
N
OMe
O
O
Ph
2pa - ^{13}C NMR (101 MHz, $CDCl_3$)
190 180 170 160 150 140 130 120 110 100 90 80 70 60 50 40 30 20 10
f1 (ppm)

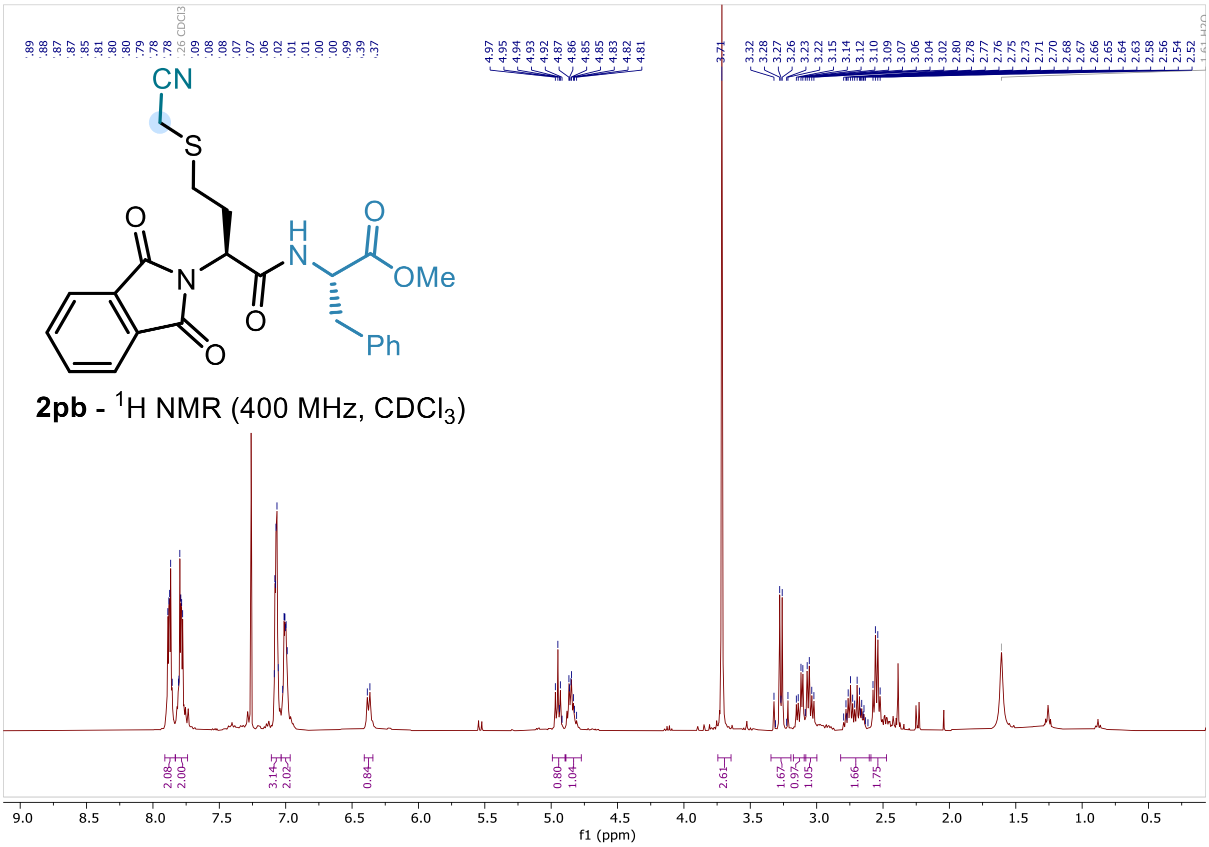
2pb - ^{1}H NMR (400 MHz, CDCl$_3$)
f1 (ppm)

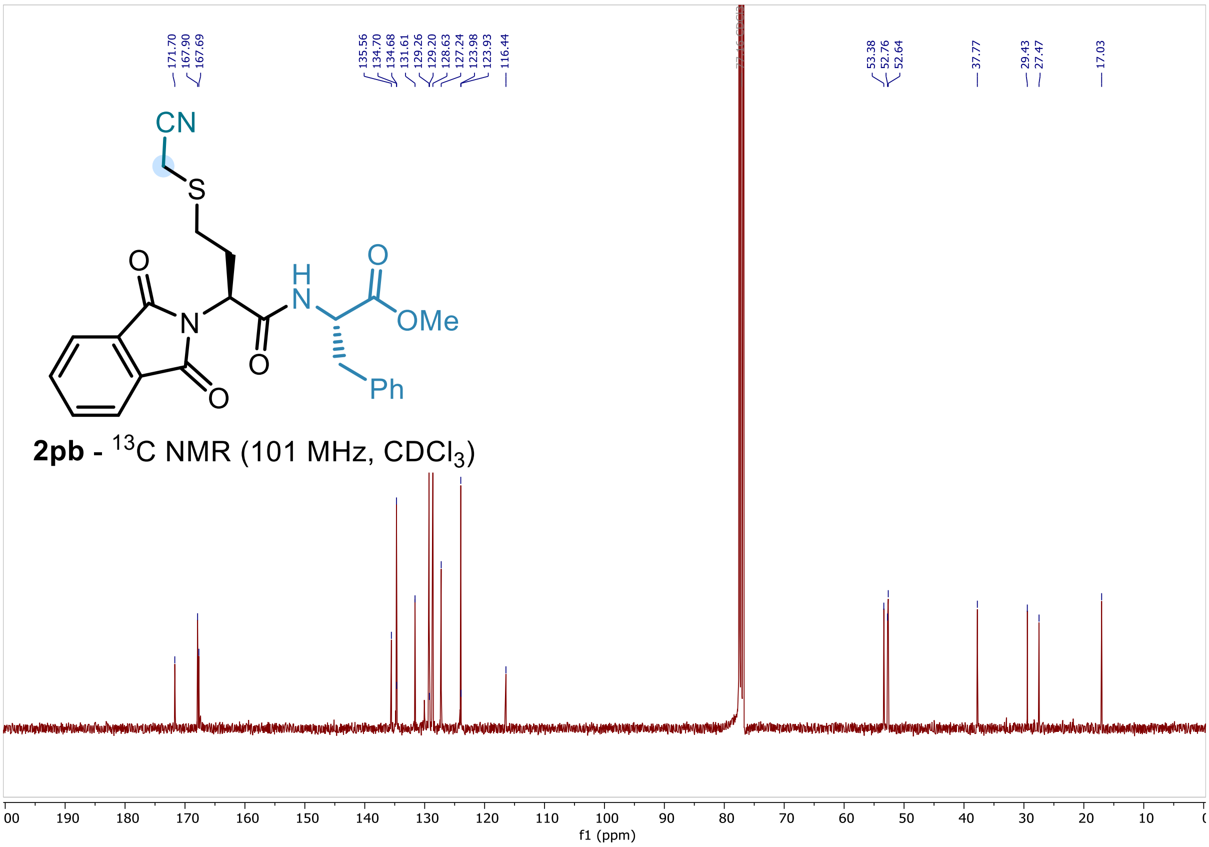
2pb - ^{13}C NMR (101 MHz, CDCl$_3$)
171.70
167.90
167.69
135.56
134.70
134.68
131.61
129.26
129.20
128.63
127.24
123.98
123.93
116.44
53.38
52.76
52.64
37.77
29.43
27.47
17.03
f1 (ppm)

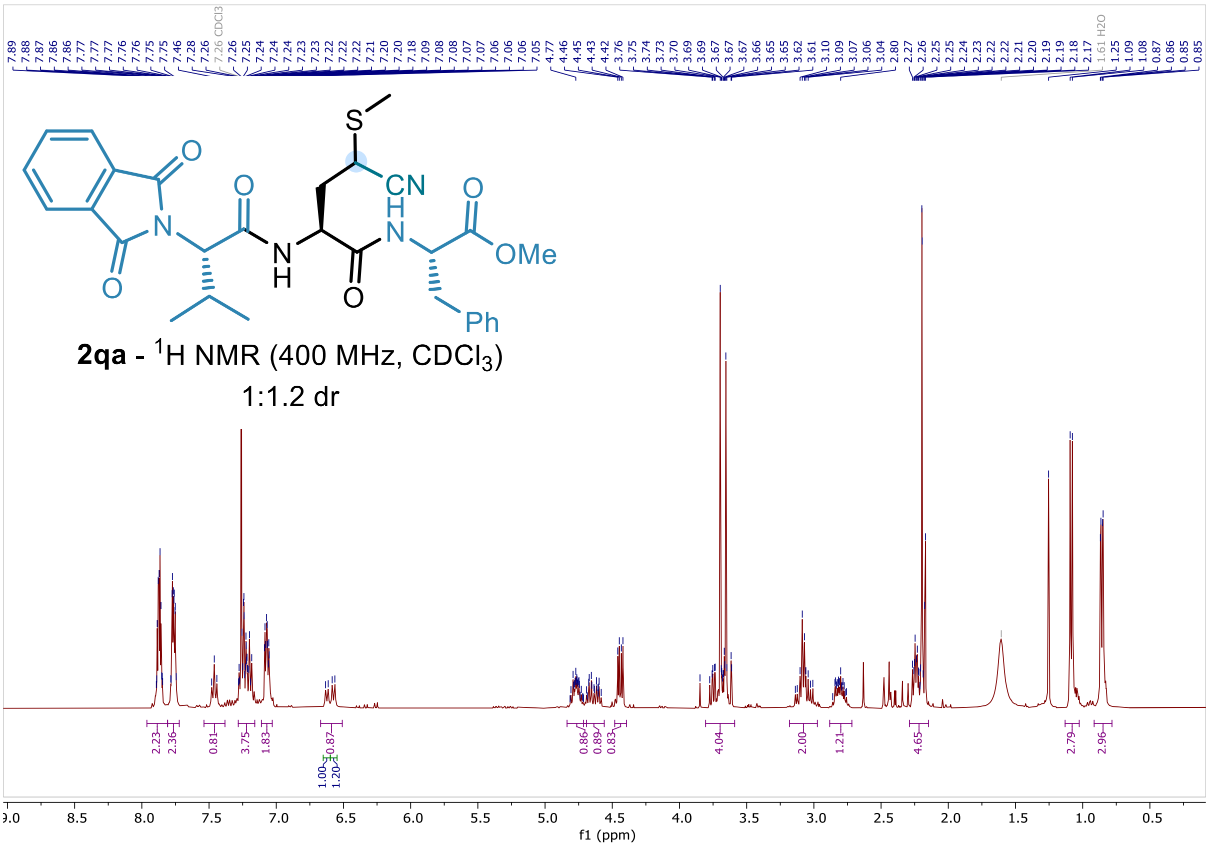
2qa - ^{1}H NMR (400 MHz, $CDCl_3$)
1:1.2 dr
f1 (ppm)

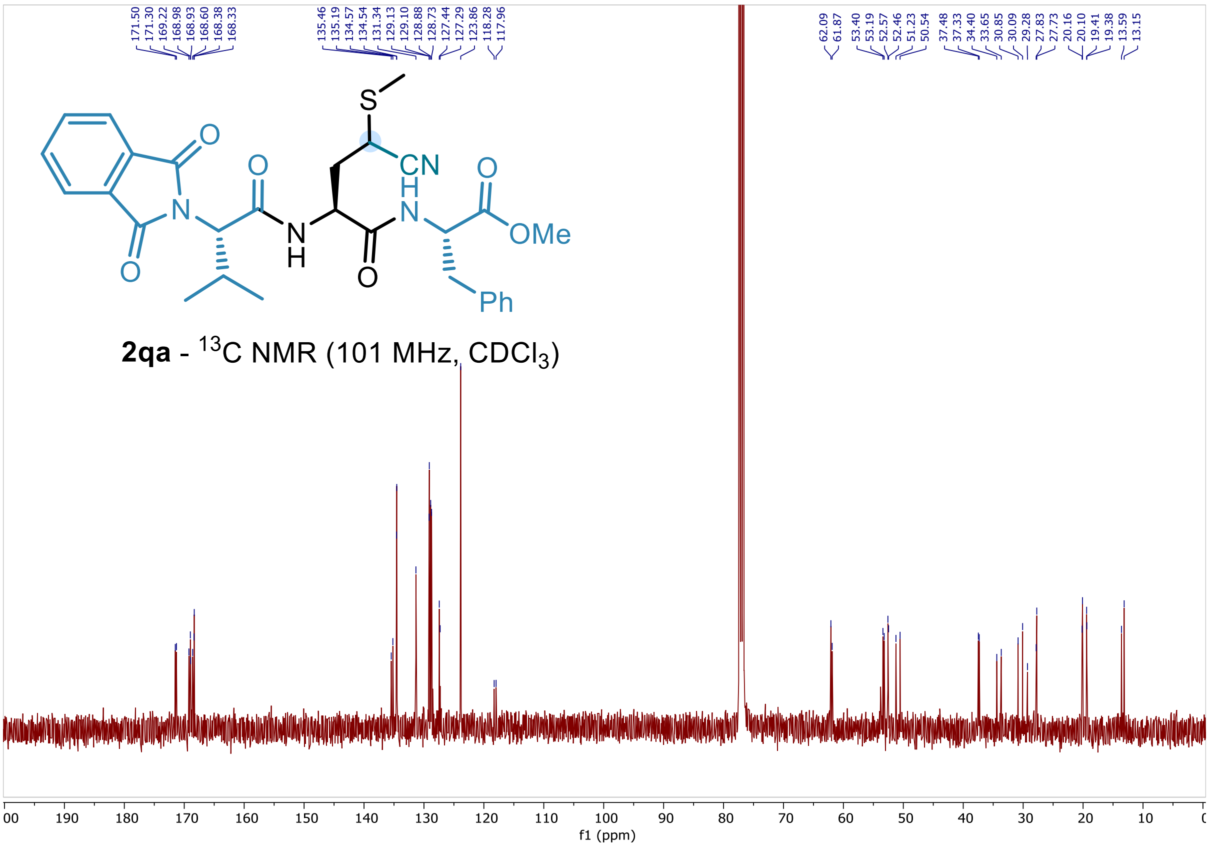
2qa - ^{13}C NMR (101 MHz, $CDCl_3$)
f1 (ppm)

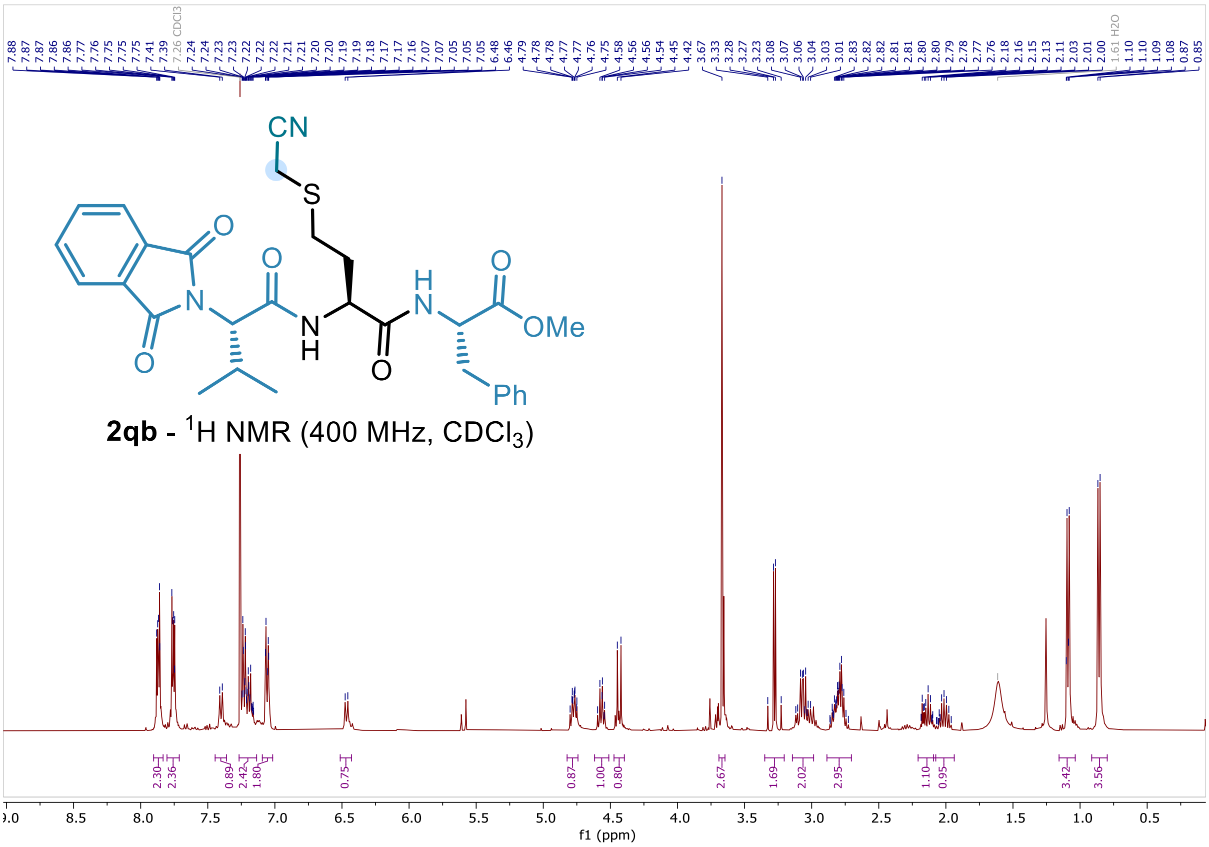

2qb - ^{1}H NMR (400 MHz, $CDCl_3$)

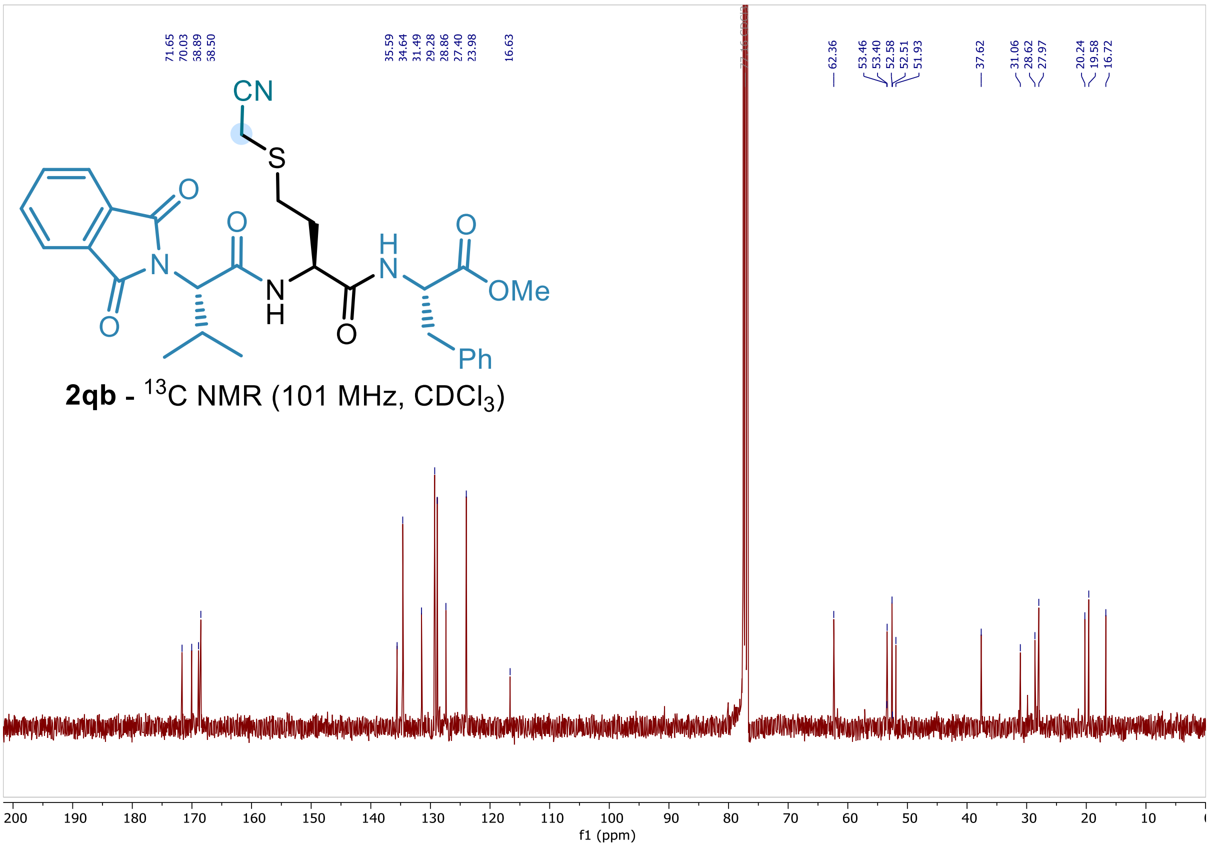

2qb - ^{13}C NMR (101 MHz, $CDCl_3$)

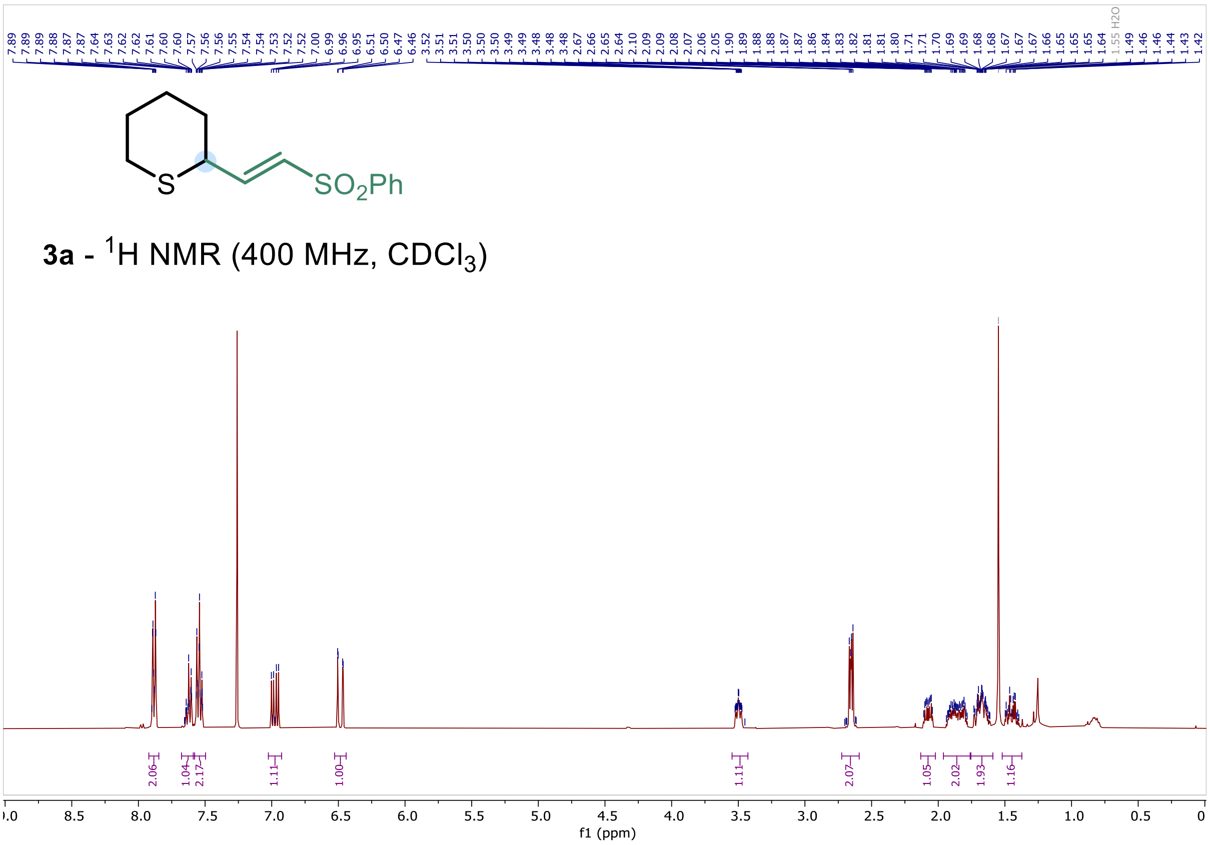
SO2Ph
3a - 1H NMR (400 MHz, CDCl3)
f1 (ppm)

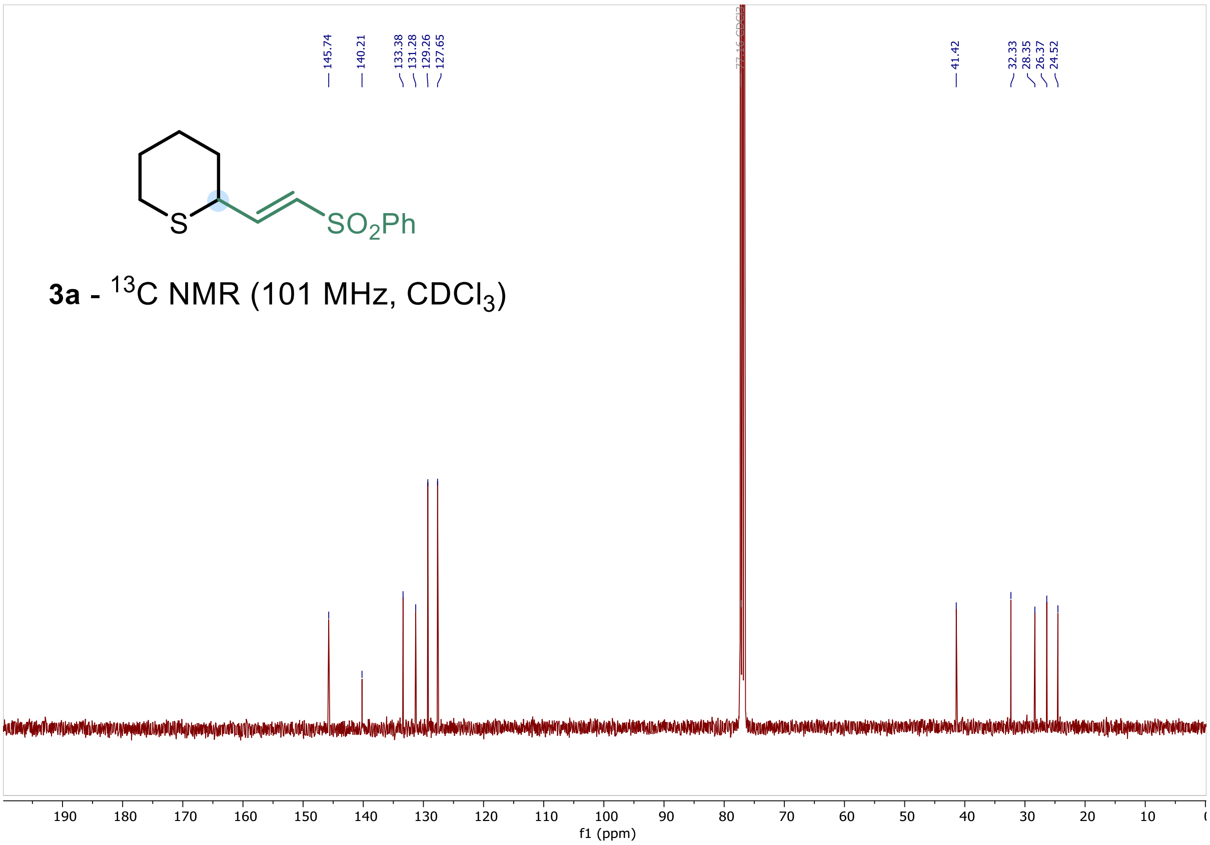
145.74
140.21
133.38
131.28
129.26
127.65
41.42
32.33
28.35
26.37
24.52
SO2Ph
3a - 13C NMR (101 MHz, CDCl3)
f1 (ppm)

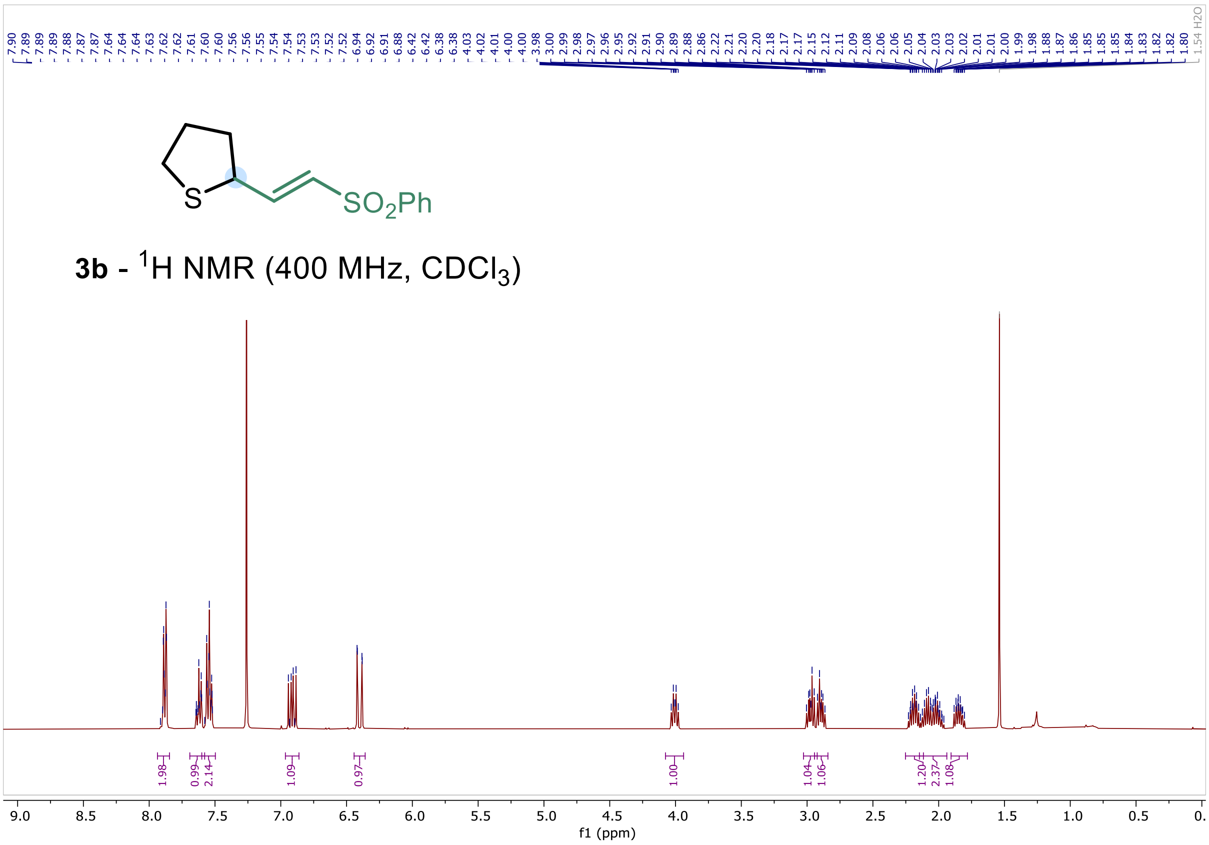
SO2Ph
S
3b - 1H NMR (400 MHz, CDCl3)
f1 (ppm)

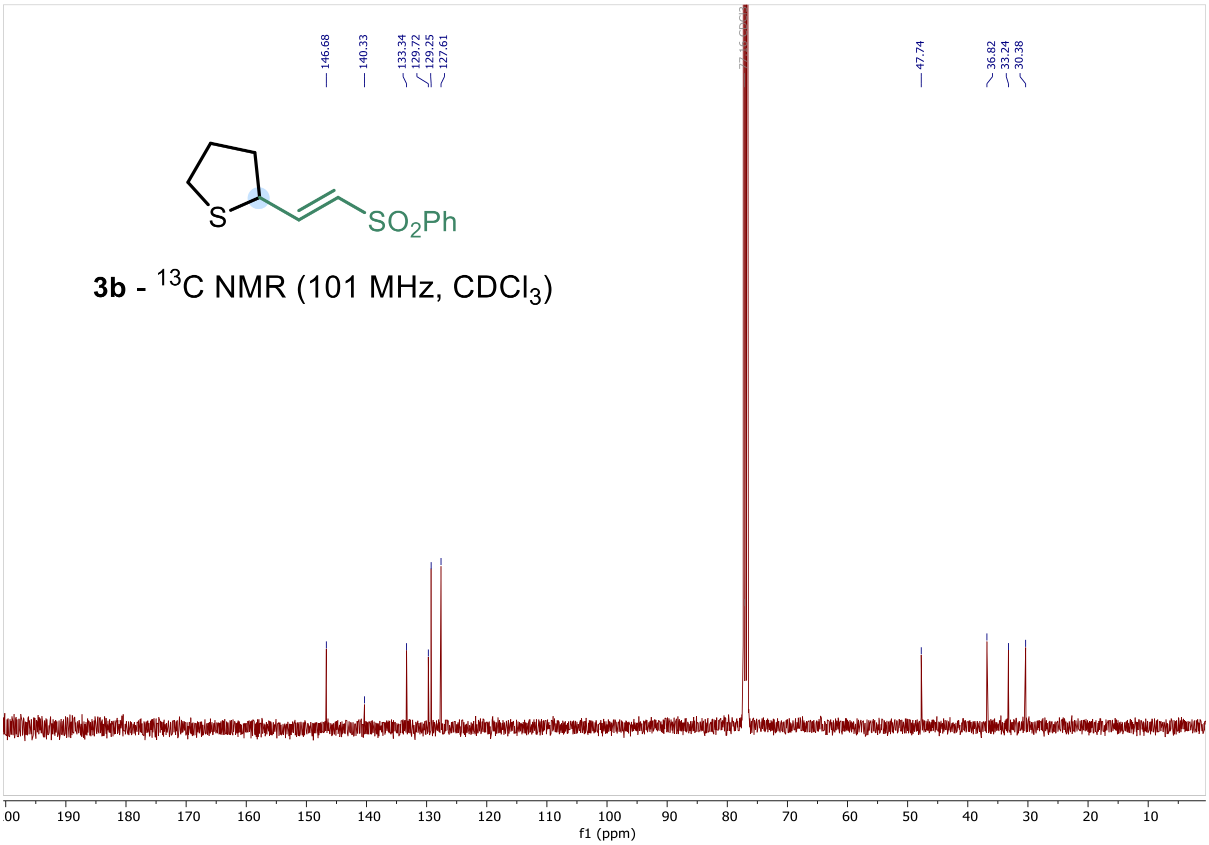
146.68
140.33
133.34
129.72
129.25
127.61
47.74
36.82
33.24
30.38
SO2Ph
S
3b - 13C NMR (101 MHz, CDCl3)
f1 (ppm)

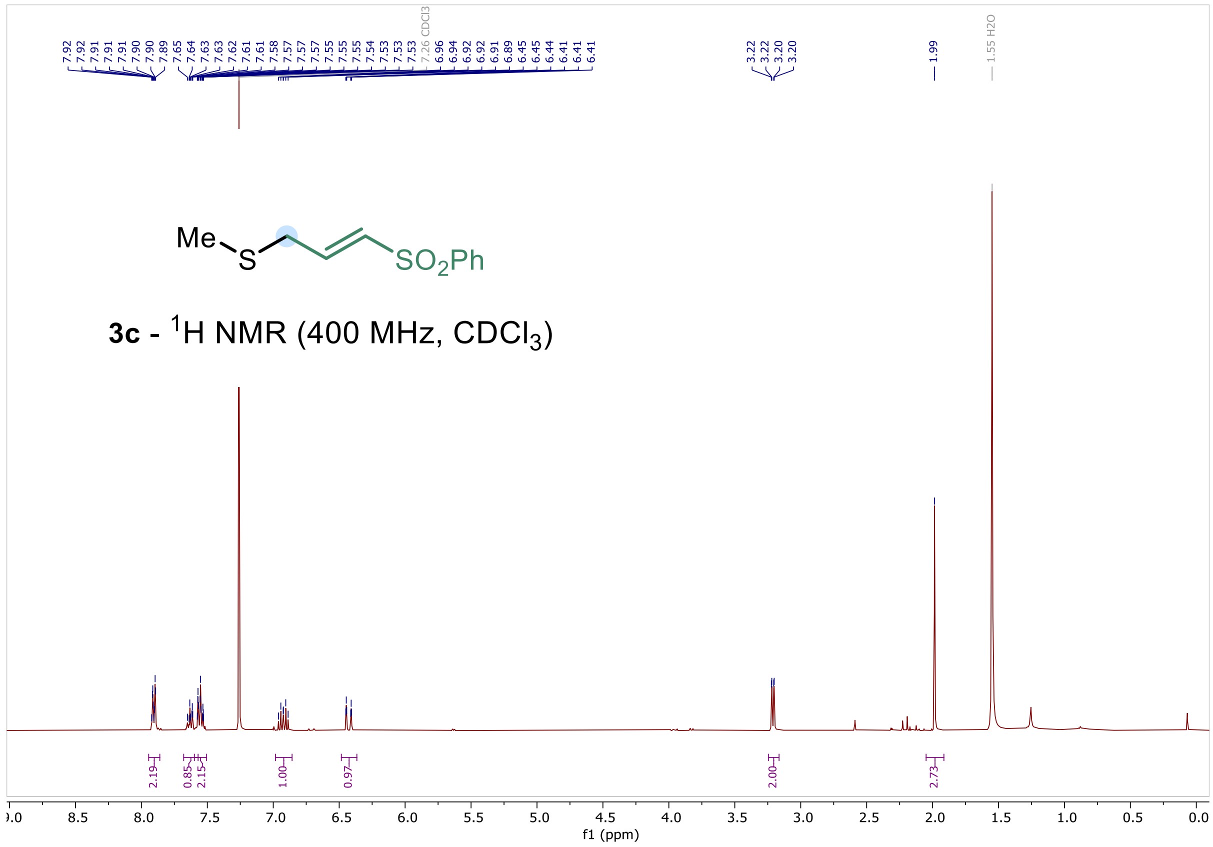
Me
S
SO2Ph
3c - 1H NMR (400 MHz, CDCl3)
7.92
7.92
7.91
7.91
7.91
7.90
7.90
7.89
7.65
7.64
7.63
7.63
7.62
7.61
7.61
7.58
7.57
7.57
7.57
7.55
7.55
7.55
7.54
7.53
7.53
7.53
7.26 CDCl3
6.96
6.94
6.92
6.92
6.91
6.89
6.45
6.45
6.44
6.41
6.41
6.41
3.22
3.22
3.20
3.20
1.99
1.55 H2O
2.19
0.85
2.15
1.00
0.97
2.00
2.73
f1 (ppm)

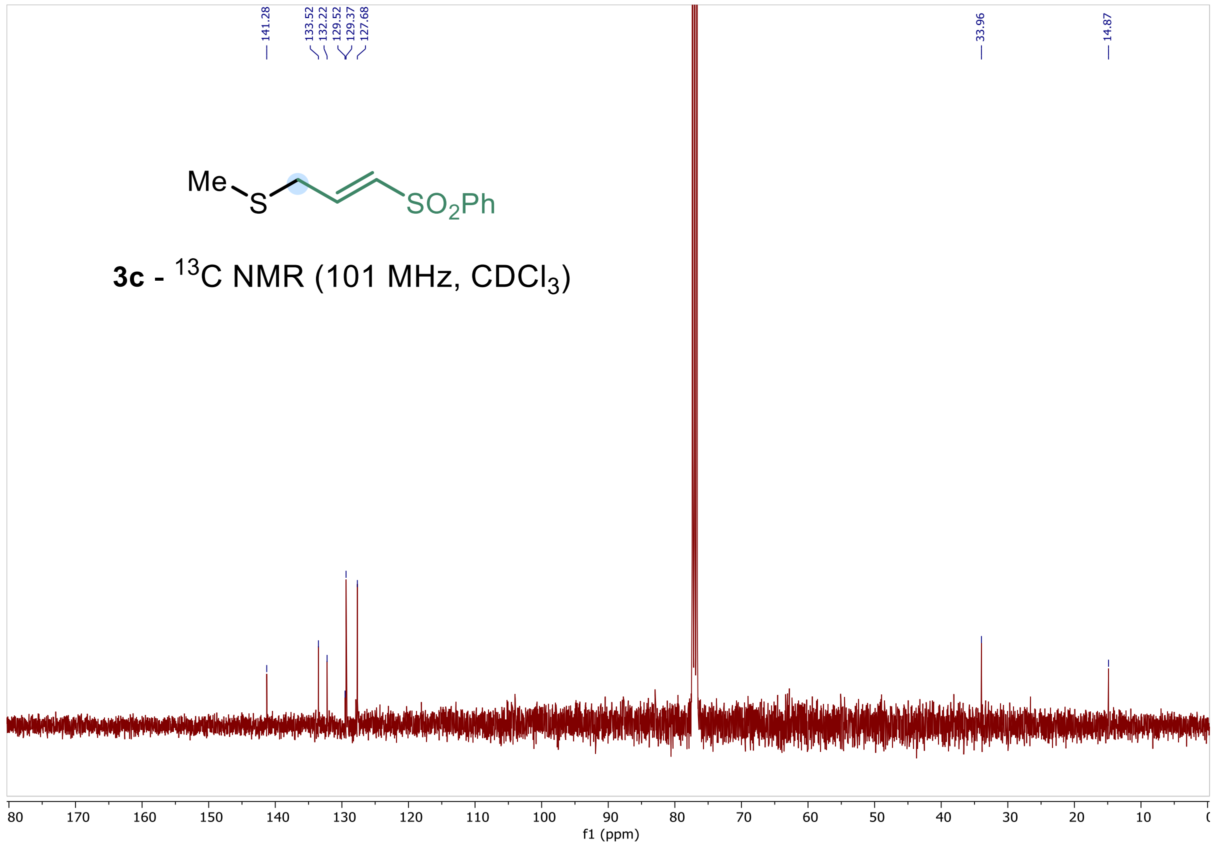
141.28
133.52
132.22
129.52
129.37
127.68
33.96
14.87
Me
S
SO2Ph
3c - 13C NMR (101 MHz, CDCl3)
f1 (ppm)

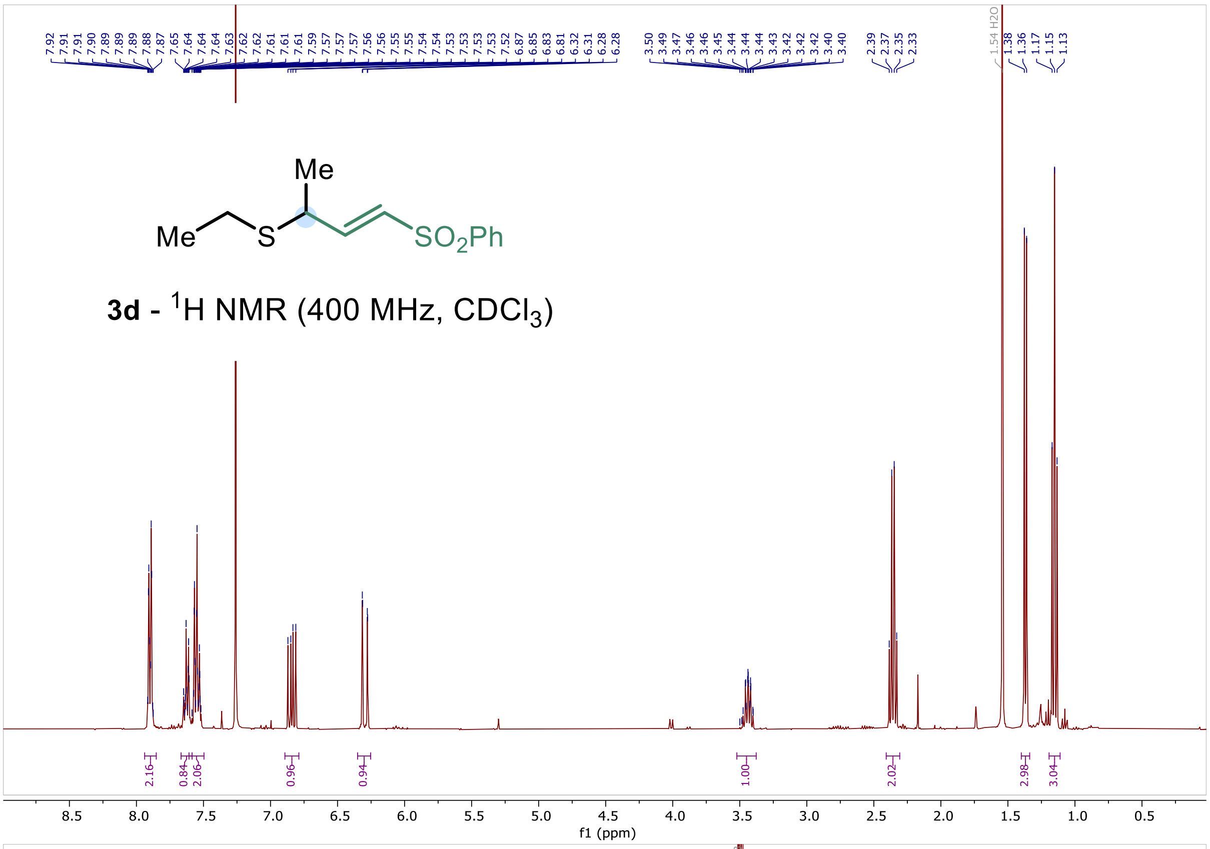
Me
Me
S
SO2Ph
3d - 1H NMR (400 MHz, CDCl3)
f1 (ppm)

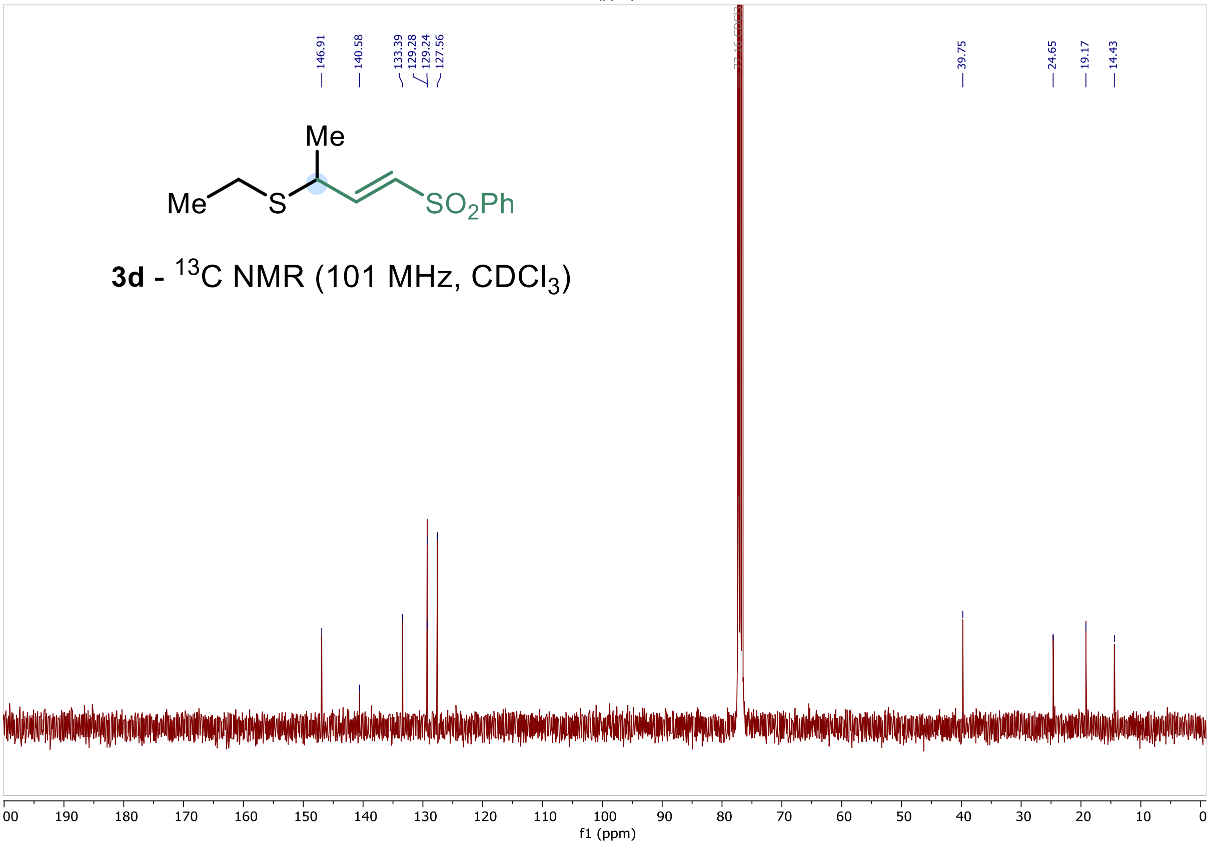
Me
Me
S
SO2Ph
3d - 13C NMR (101 MHz, CDCl3)
f1 (ppm)

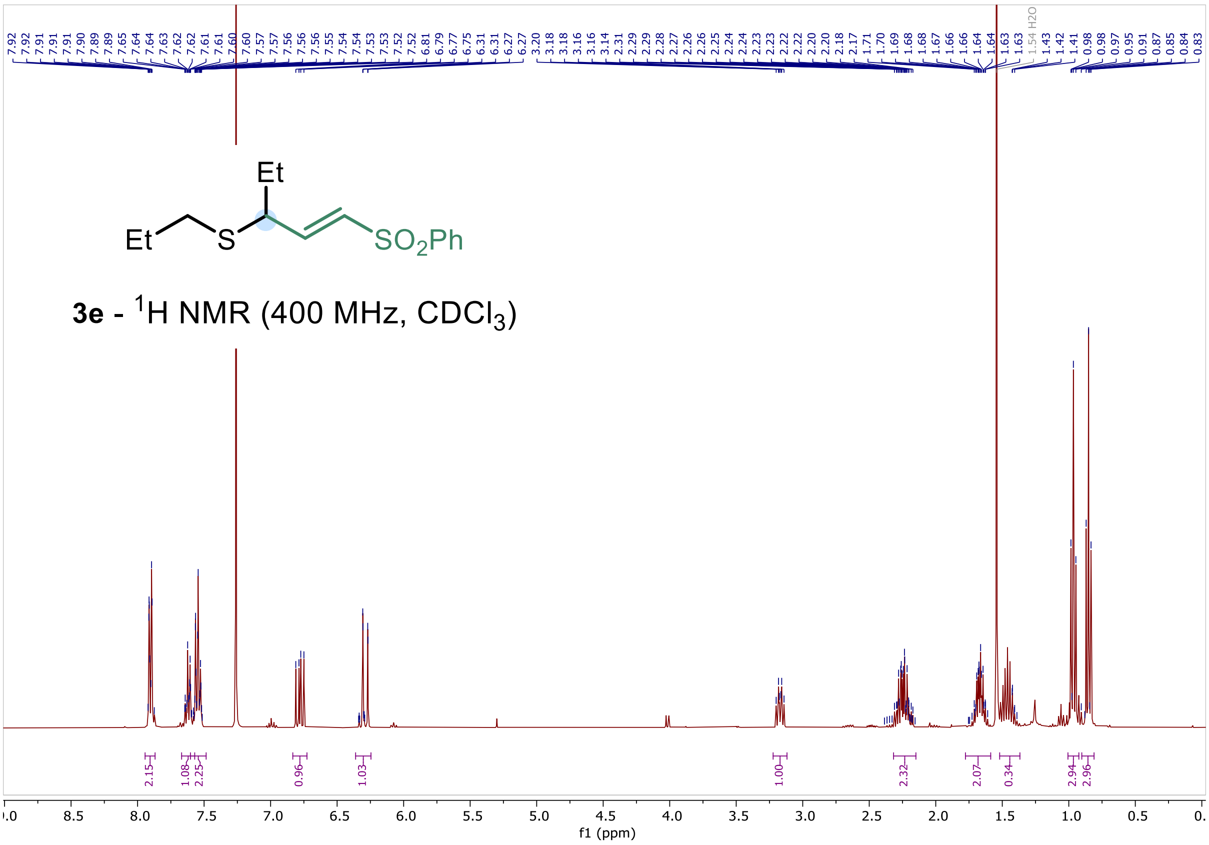

3e - ^{1}H NMR (400 MHz, $CDCl_3$)

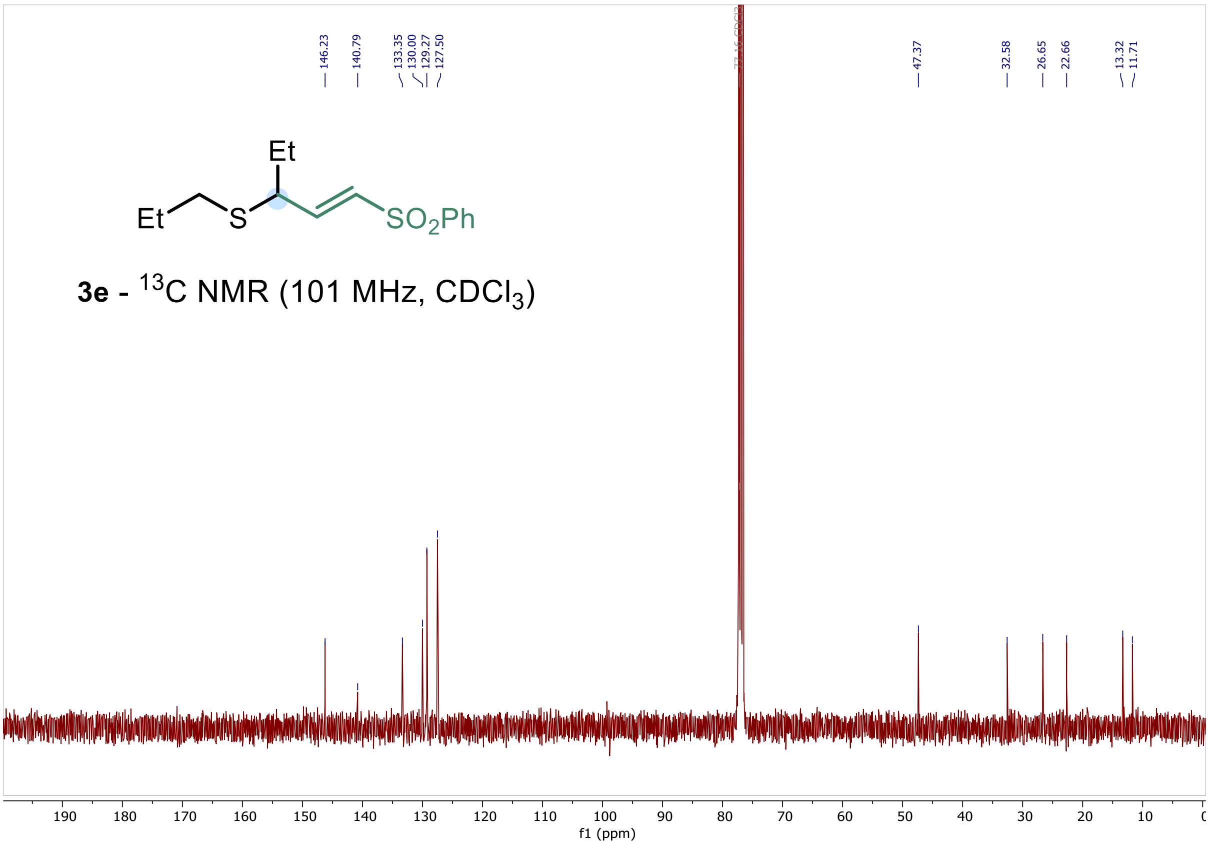

3e - ^{13}C NMR (101 MHz, $CDCl_3$)

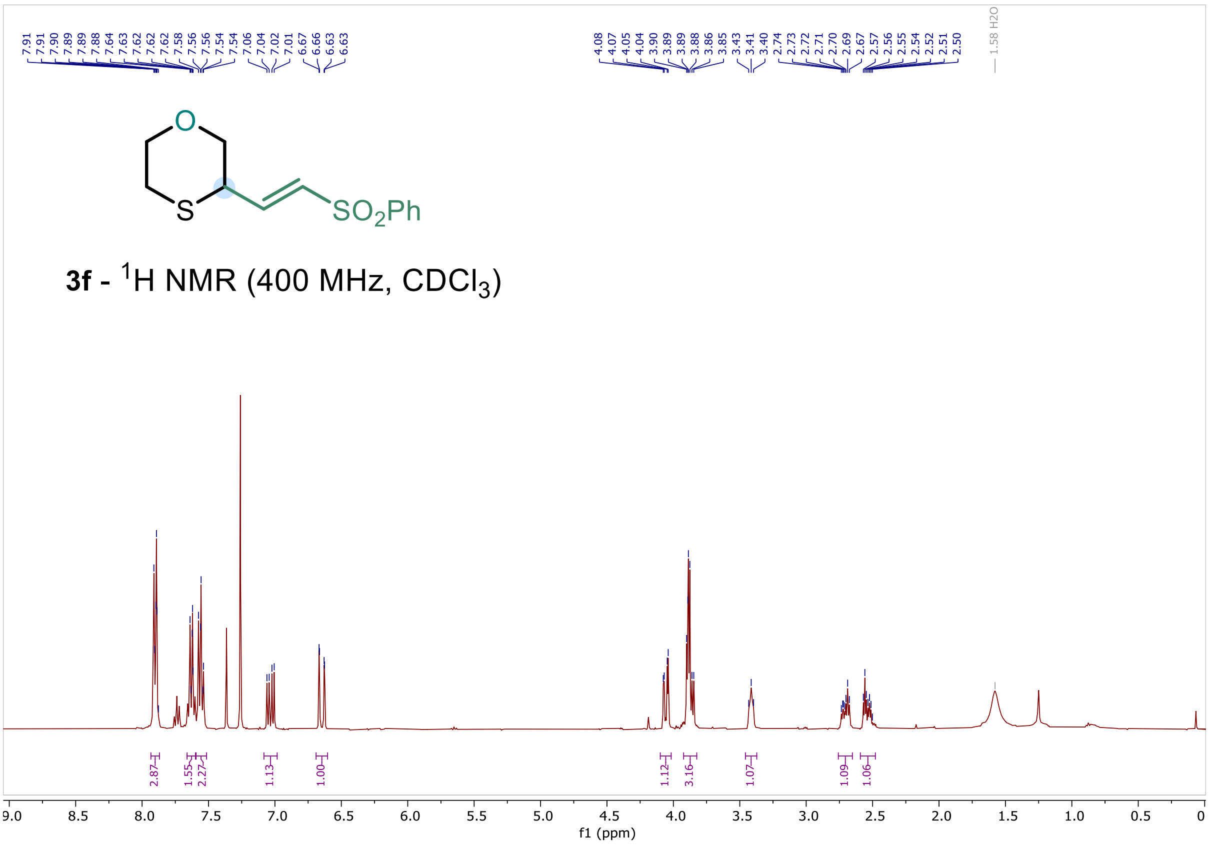

3f - ^{1}H NMR (400 MHz, $CDCl_3$)

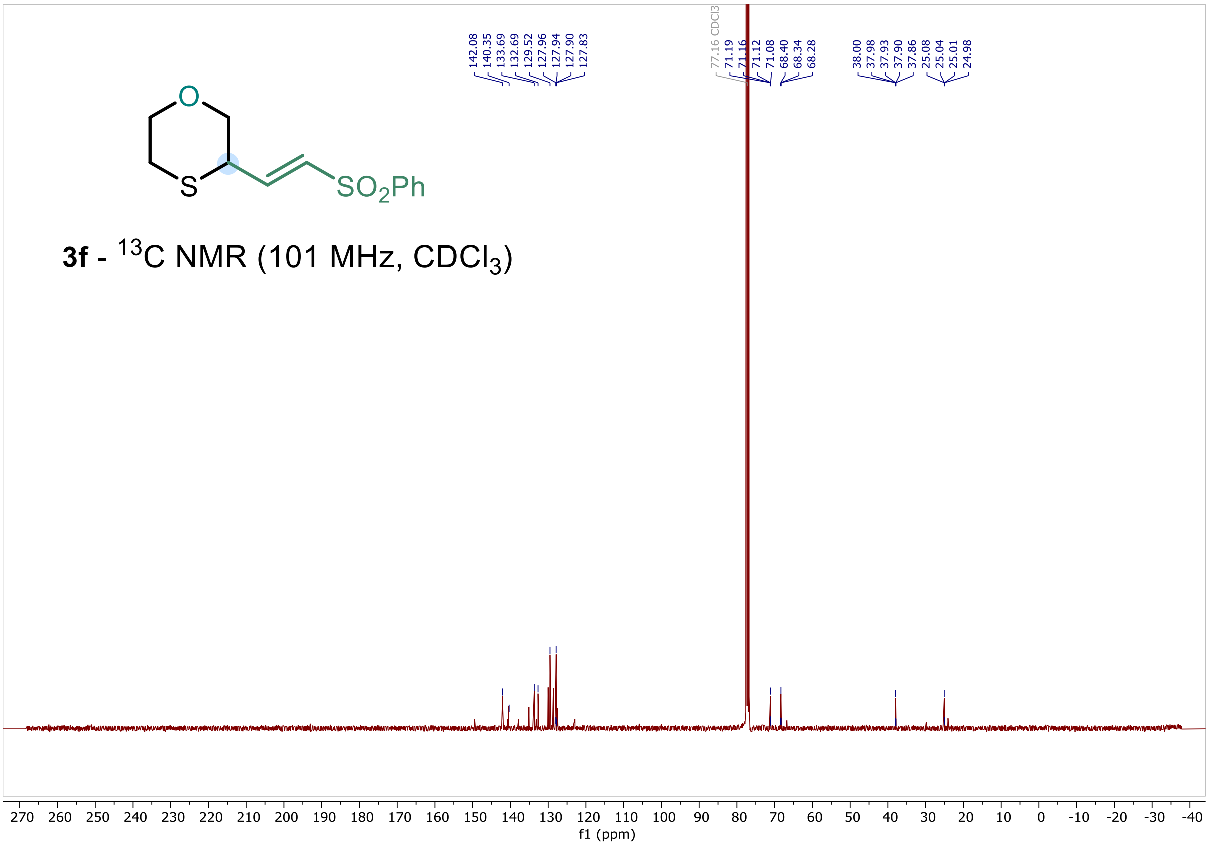

3f - ^{13}C NMR (101 MHz, $CDCl_3$)

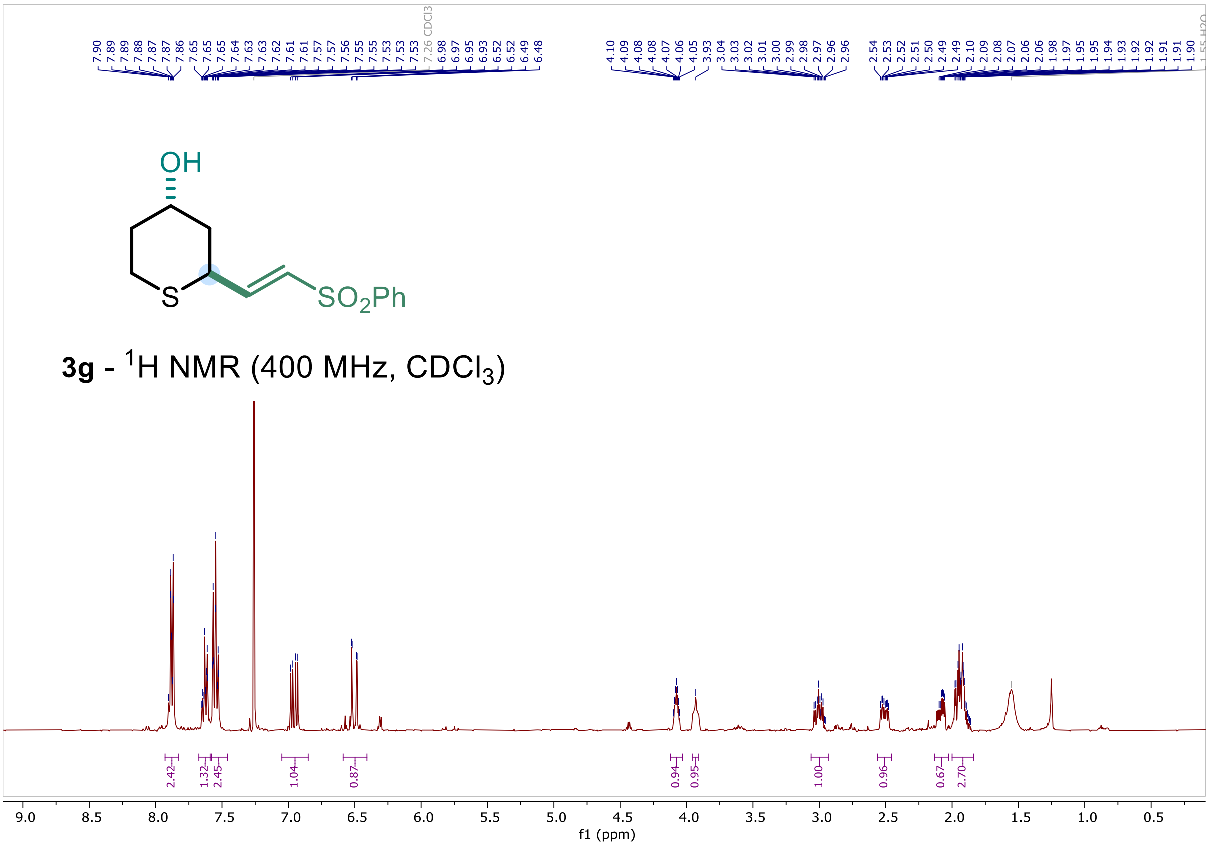
OH
S
SO2Ph
3g - 1H NMR (400 MHz, CDCl3)
7.90 7.89 7.89 7.88 7.87 7.87 7.86 7.65 7.65 7.65 7.64 7.63 7.63 7.62 7.61 7.61 7.57 7.57 7.56 7.55 7.55 7.53 7.53 7.53 7.26 CDCl3 6.98 6.97 6.95 6.93 6.52 6.52 6.49 6.48
4.10 4.09 4.08 4.07 4.06 4.05 3.93 3.04 3.03 3.02 3.01 3.00 2.99 2.98 2.97 2.96 2.96
2.54 2.53 2.52 2.51 2.50 2.49 2.49 2.10 2.09 2.08 2.07 2.06 2.06 1.98 1.97 1.95 1.95 1.94 1.93 1.93 1.92 1.91 1.91 1.90
2.42 1.32 2.45 1.04 0.87 0.94 0.95 1.00 0.96 0.67 2.70
f1 (ppm)

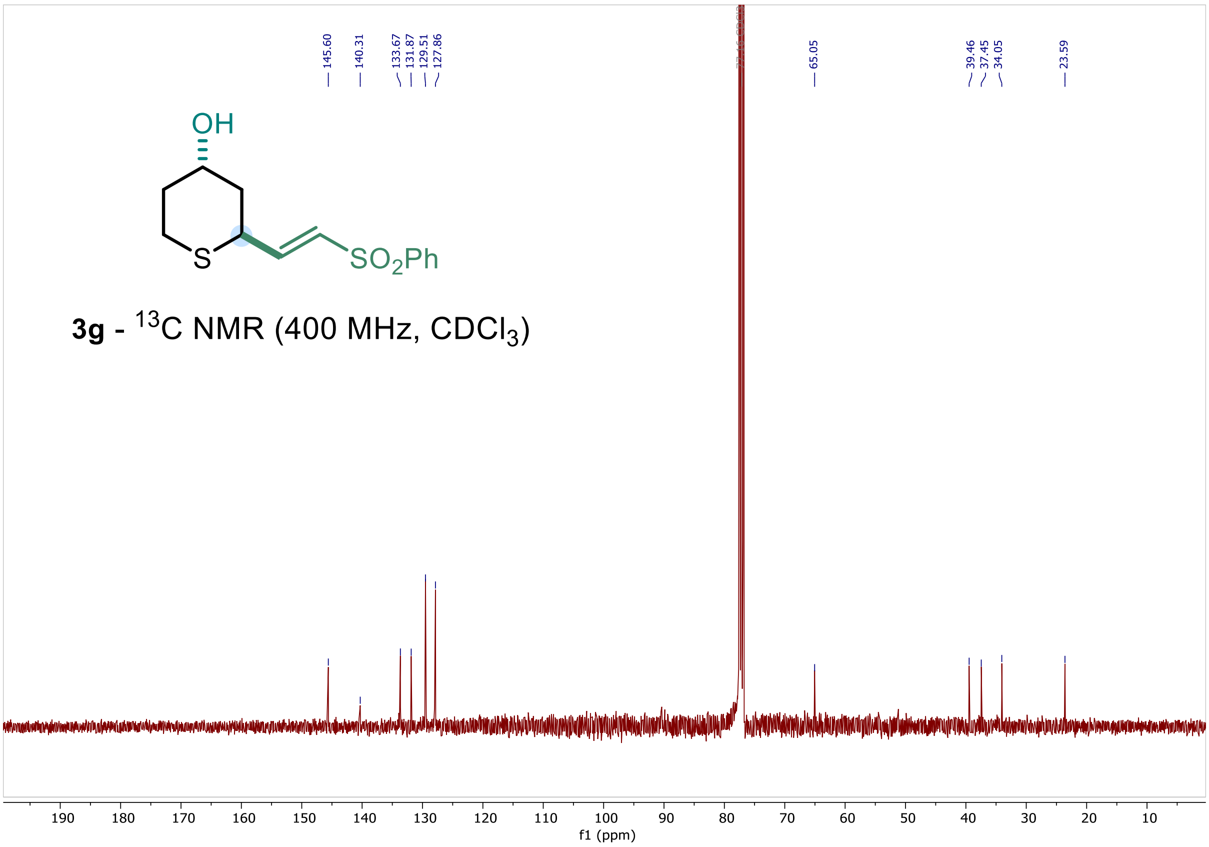
145.60 140.31 133.67 131.87 129.51 127.86 65.05 39.46 37.45 34.05 23.59
OH
S
SO2Ph
3g - 13C NMR (400 MHz, CDCl3)
f1 (ppm)

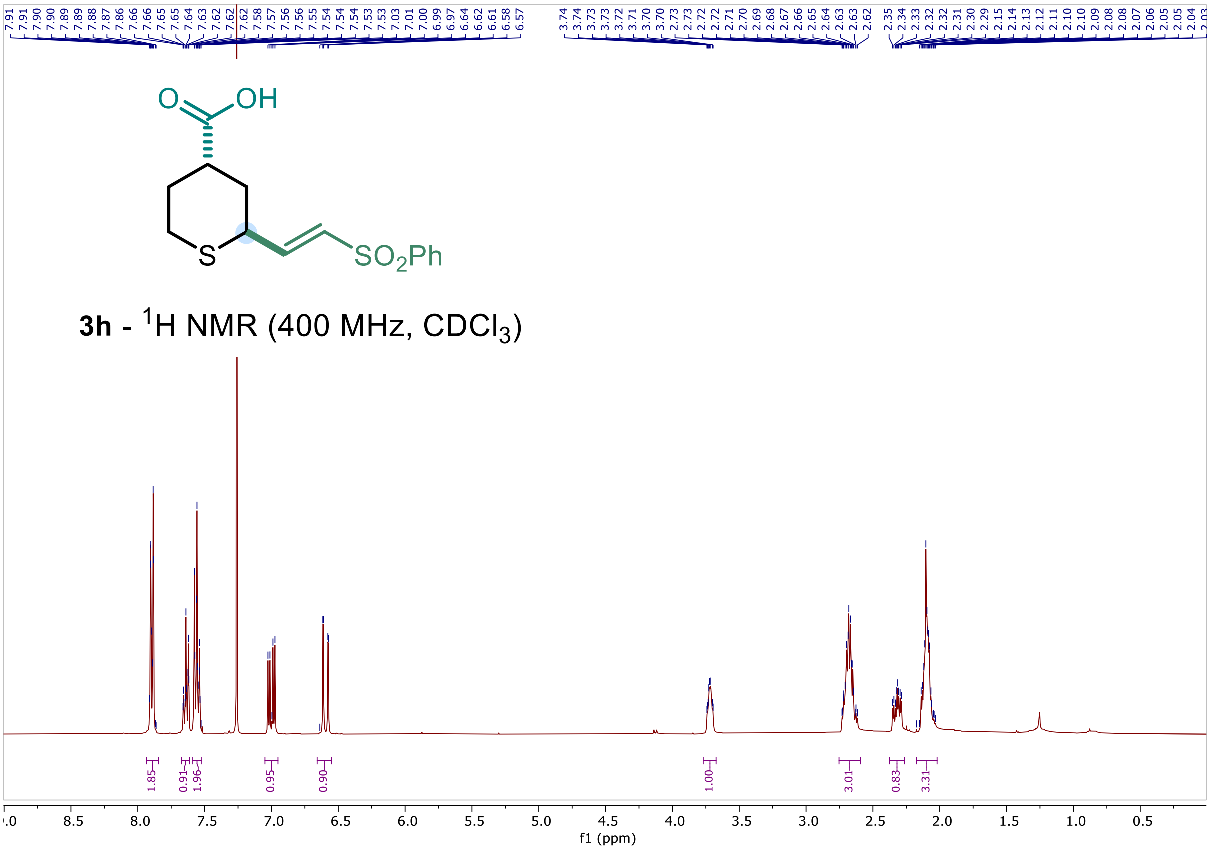

O
OH
S
SO2Ph
3h - 1H NMR (400 MHz, CDCl3)
f1 (ppm)

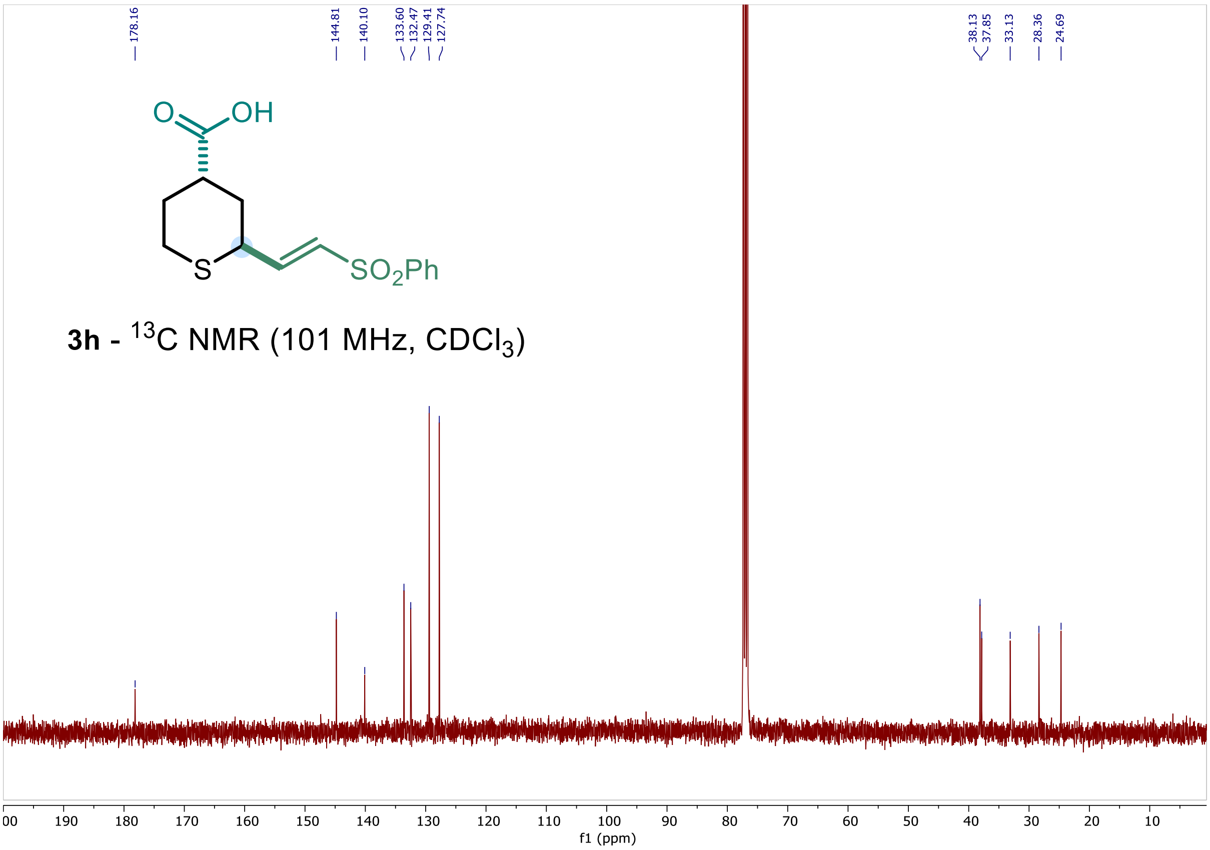

O
OH
S
SO2Ph
3h - 13C NMR (101 MHz, CDCl3)
f1 (ppm)

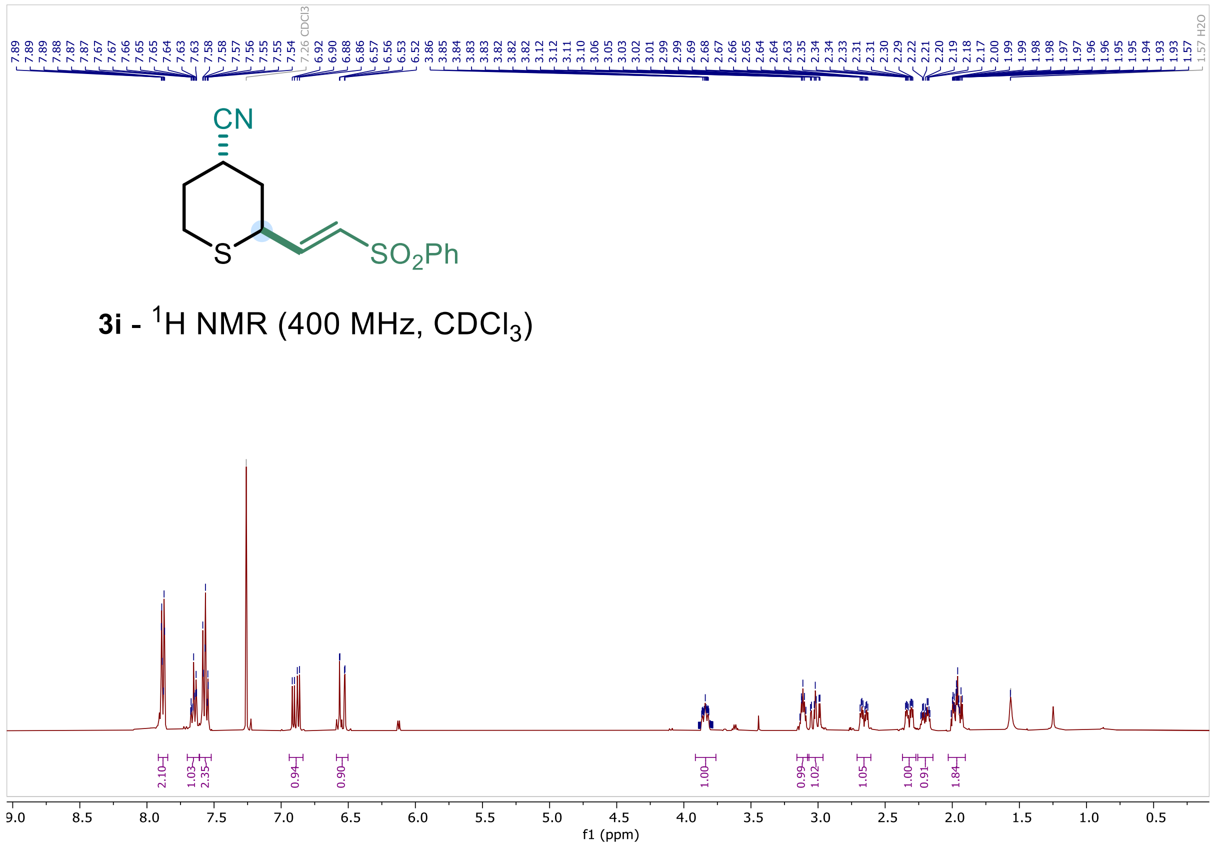
CN
S
SO2Ph
3i - 1H NMR (400 MHz, CDCl3)
f1 (ppm)

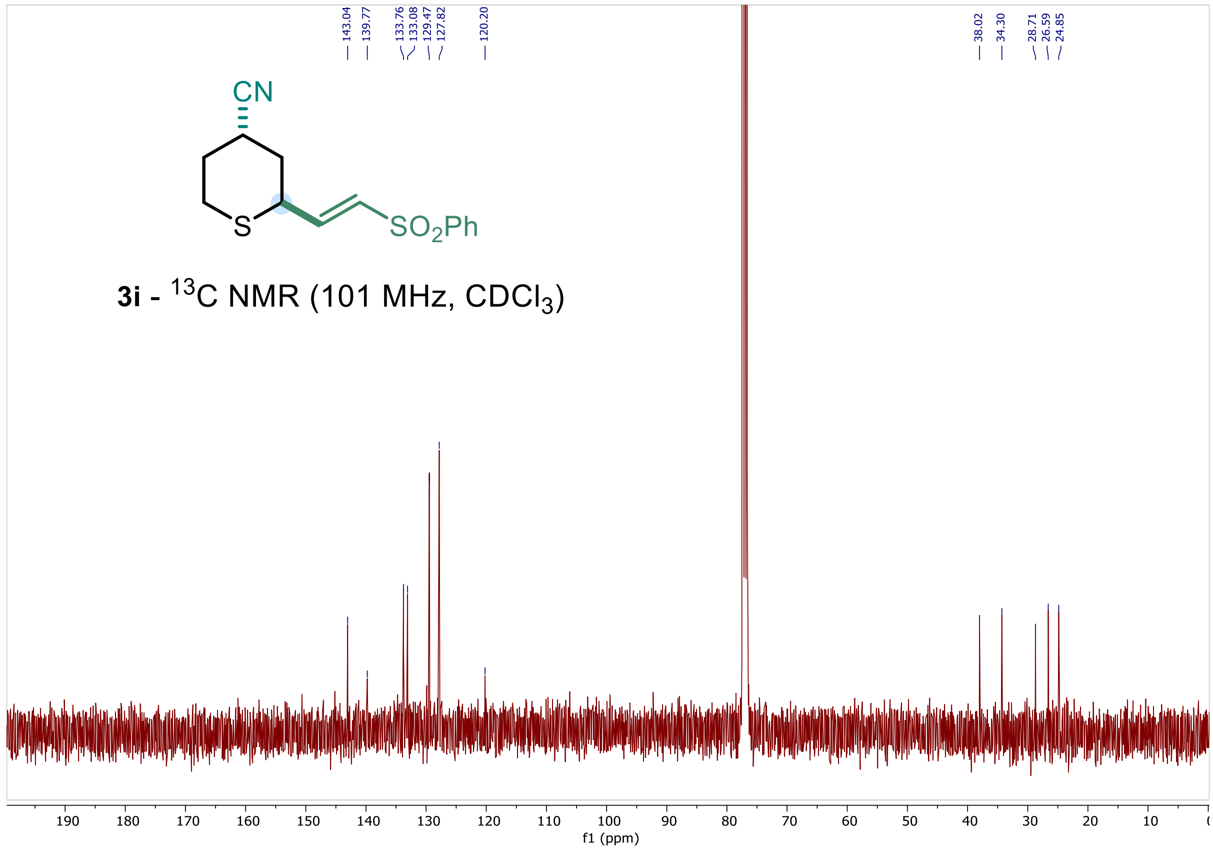
143.04
139.77
133.76
133.08
129.47
127.82
120.20
38.02
34.30
28.71
26.59
24.85
CN
S
SO2Ph
3i - 13C NMR (101 MHz, CDCl3)
f1 (ppm)

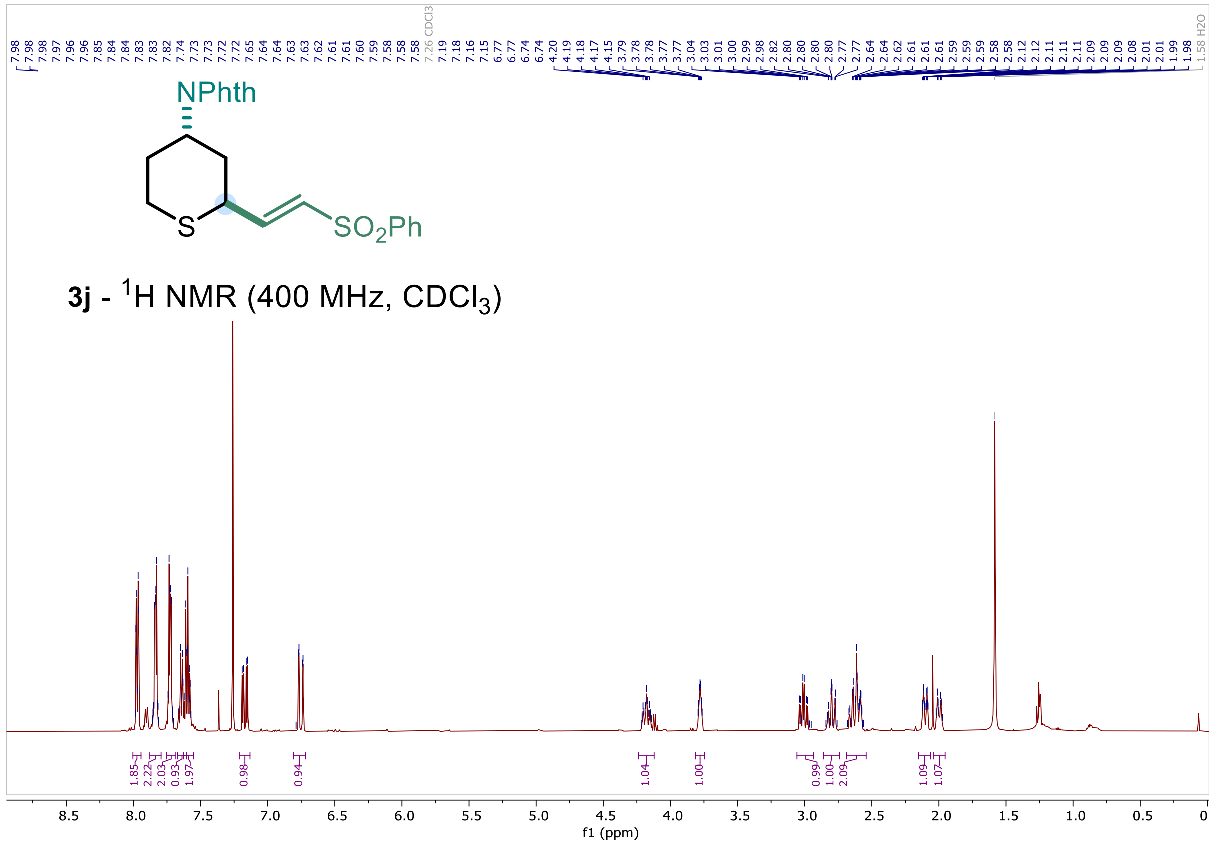
NPhth
S
SO2Ph
3j - 1H NMR (400 MHz, CDCl3)
f1 (ppm)

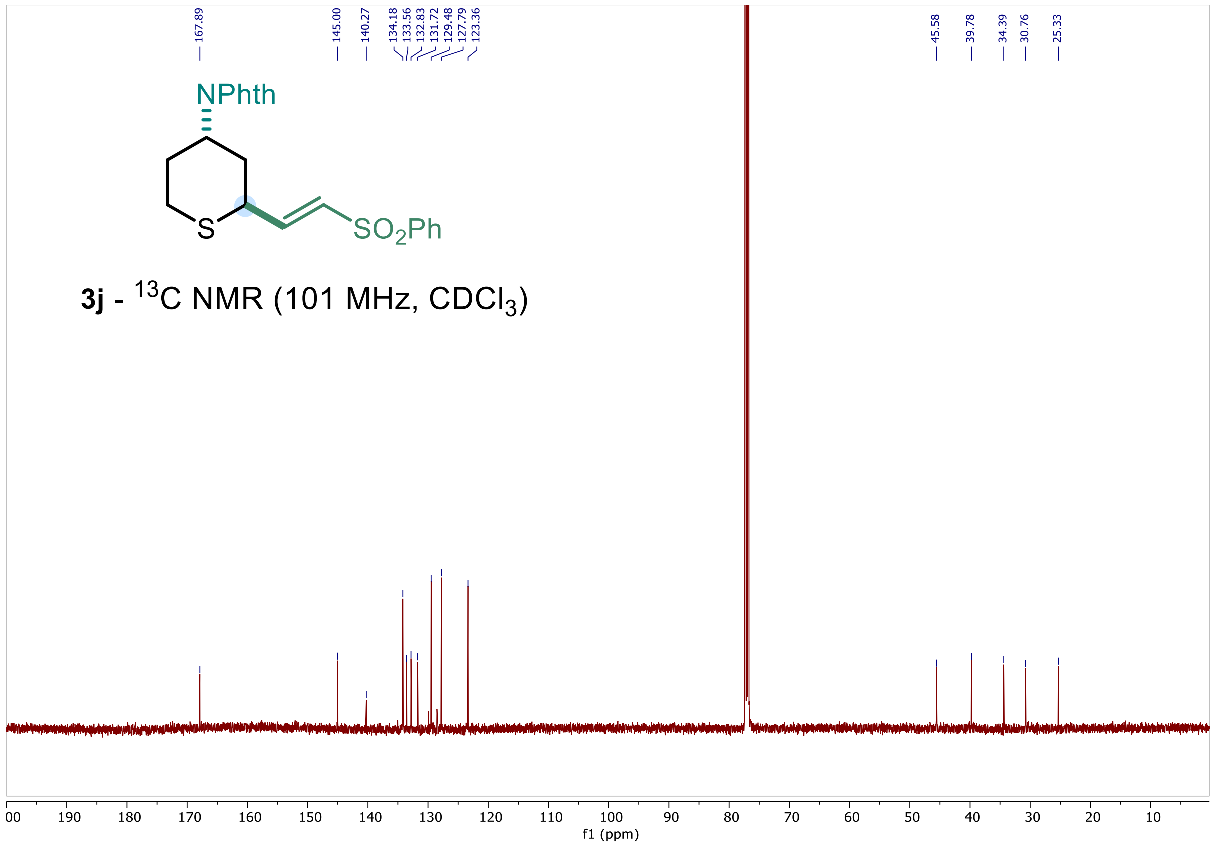
NPhth
S
SO2Ph
3j - 13C NMR (101 MHz, CDCl3)
167.89
145.00
140.27
134.18
133.56
132.83
131.72
129.48
127.79
123.36
45.58
39.78
34.39
30.76
25.33
f1 (ppm)

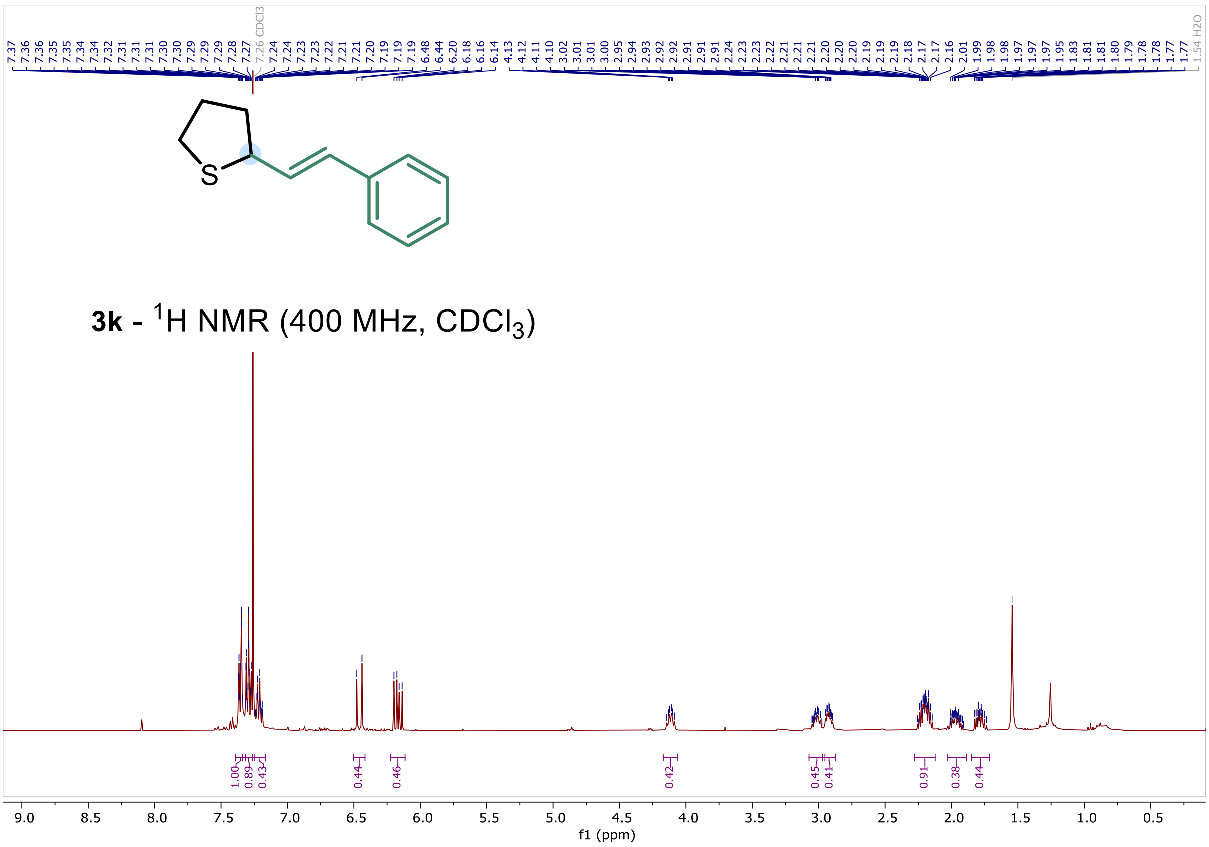
S
3k - ^{1}H NMR (400 MHz, $CDCl_3$)
f1 (ppm)

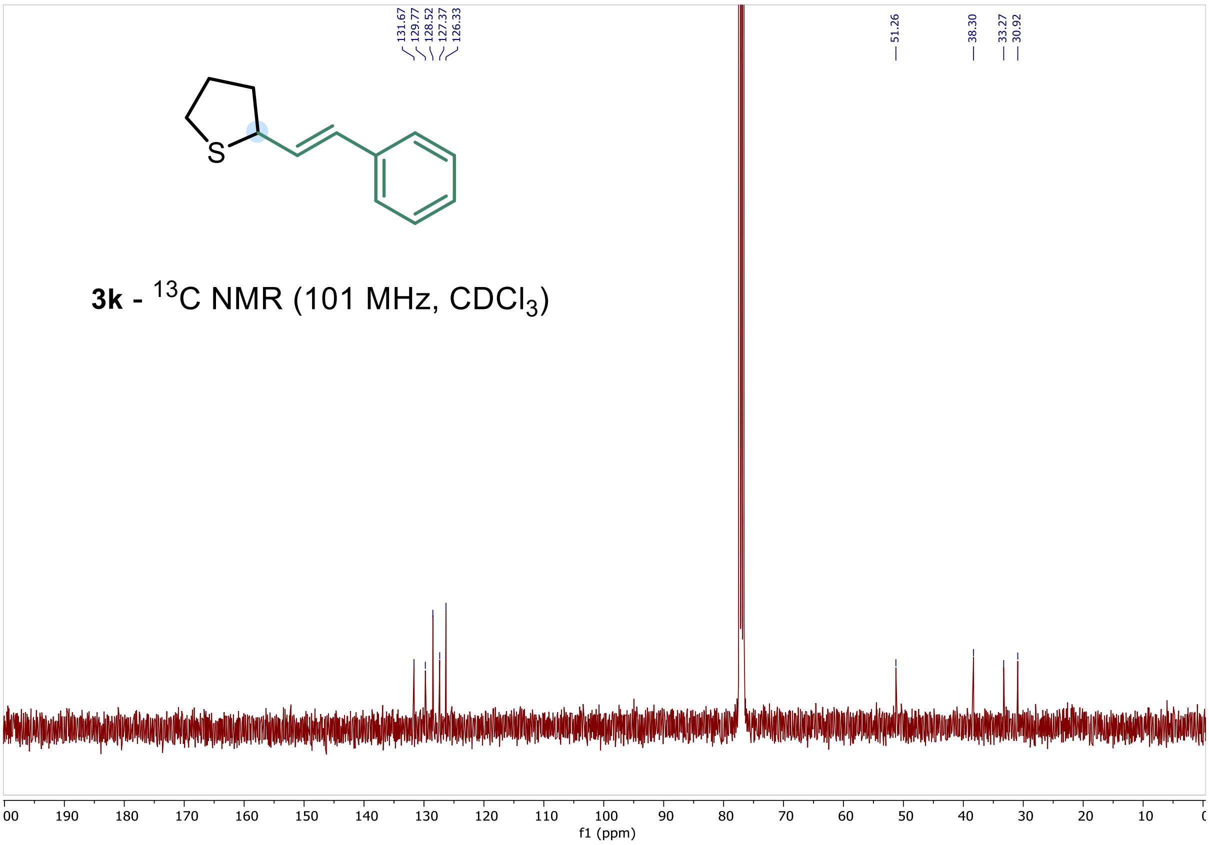
131.67
129.77
128.52
127.37
126.33
51.26
38.30
33.27
30.92
S
3k - ^{13}C NMR (101 MHz, $CDCl_3$)
f1 (ppm)

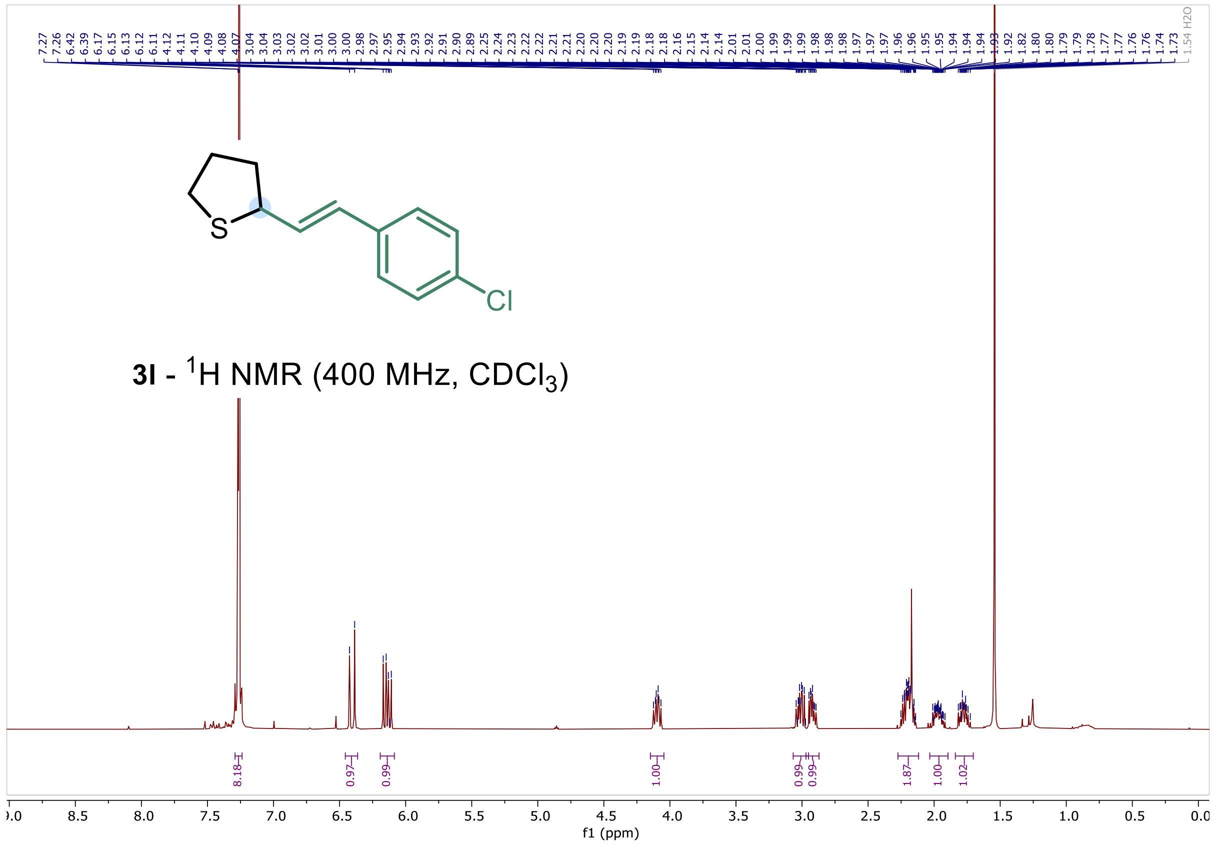

3l - ^{1}H NMR (400 MHz, $CDCl_3$)

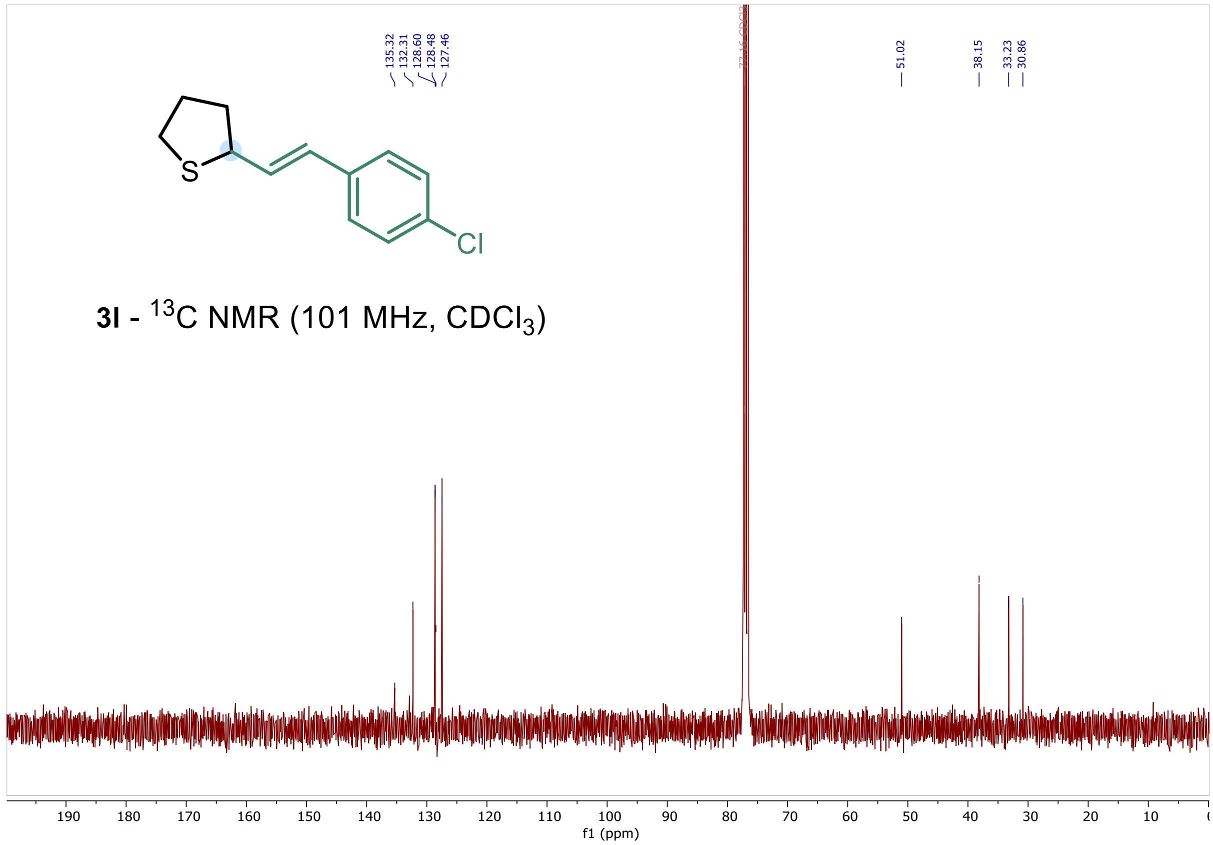

3l - ^{13}C NMR (101 MHz, $CDCl_3$)

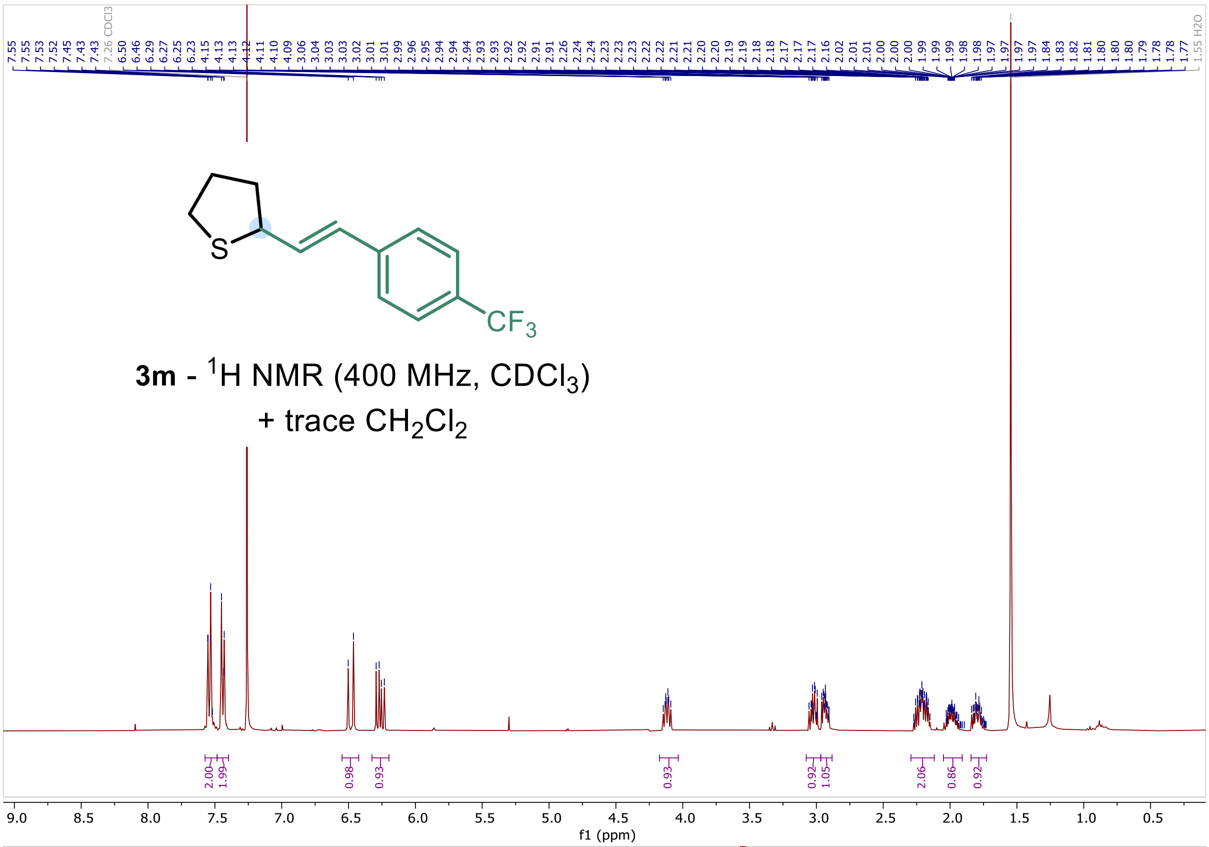
7.55 7.55 7.53 7.52 7.45 7.43 7.43 7.26 CDCl3 6.50 6.46 6.29 6.27 6.25 6.23 4.15 4.13 4.13 4.12 4.11 4.10 4.09 3.06 3.04 3.03 3.03 3.02 3.01 3.01 2.99 2.96 2.95 2.94 2.94 2.94 2.93 2.93 2.92 2.92 2.91 2.91 2.26 2.24 2.24 2.23 2.23 2.23 2.22 2.22 2.21 2.21 2.20 2.20 2.19 2.19 2.18 2.18 2.17 2.17 2.16 2.02 2.01 2.01 2.00 2.00 1.99 1.99 1.99 1.98 1.98 1.97 1.97 1.97 1.97 1.84 1.83 1.82 1.81 1.80 1.80 1.79 1.78 1.78 1.77 1.55 H2O
S
CF3
3m - ^{1}H NMR (400 MHz, $CDCl_3$)
+ trace CH_2Cl_2
2.00 1.99 0.98 0.93 0.93 0.92 1.05 2.06 0.86 0.92
f1 (ppm)

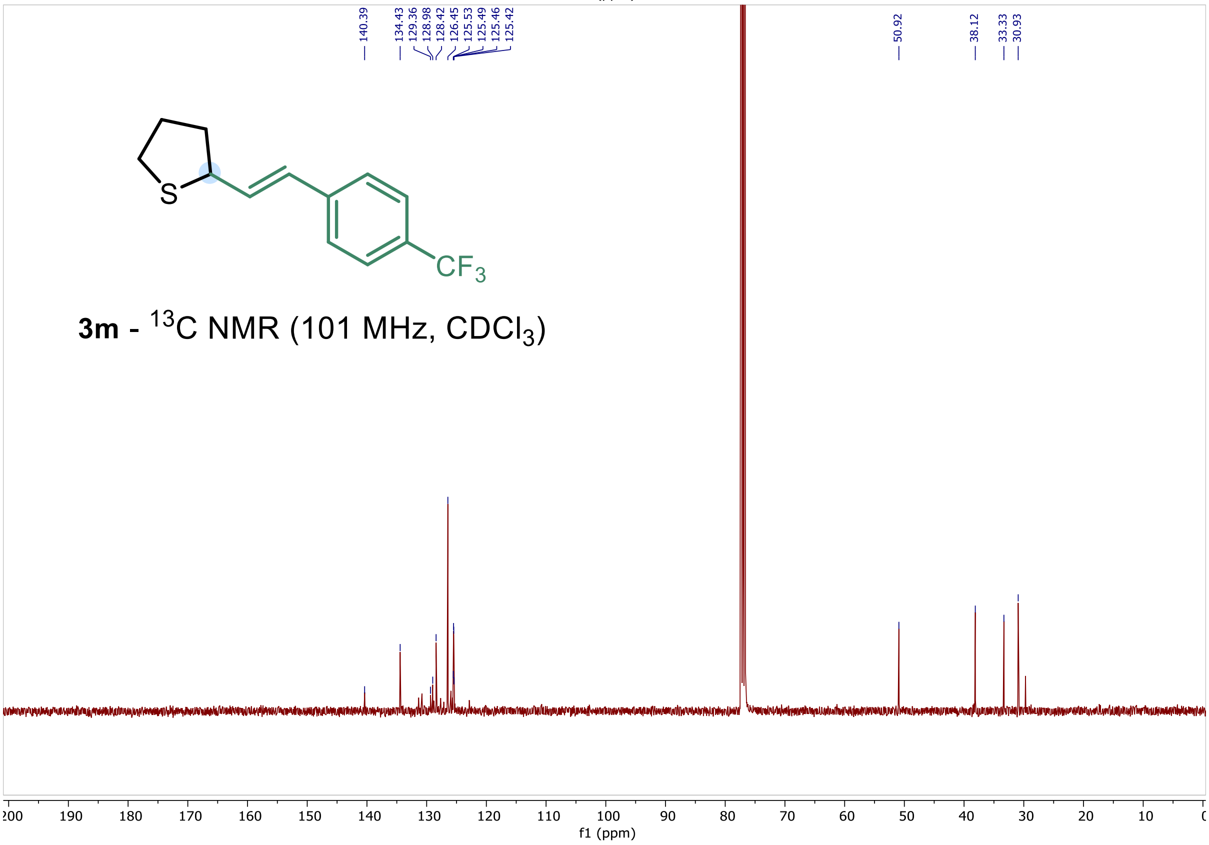
140.39 134.43 129.36 128.98 128.42 126.45 125.53 125.49 125.46 125.42 50.92 38.12 33.33 30.93
S
CF3
3m - ^{13}C NMR (101 MHz, $CDCl_3$)
f1 (ppm)

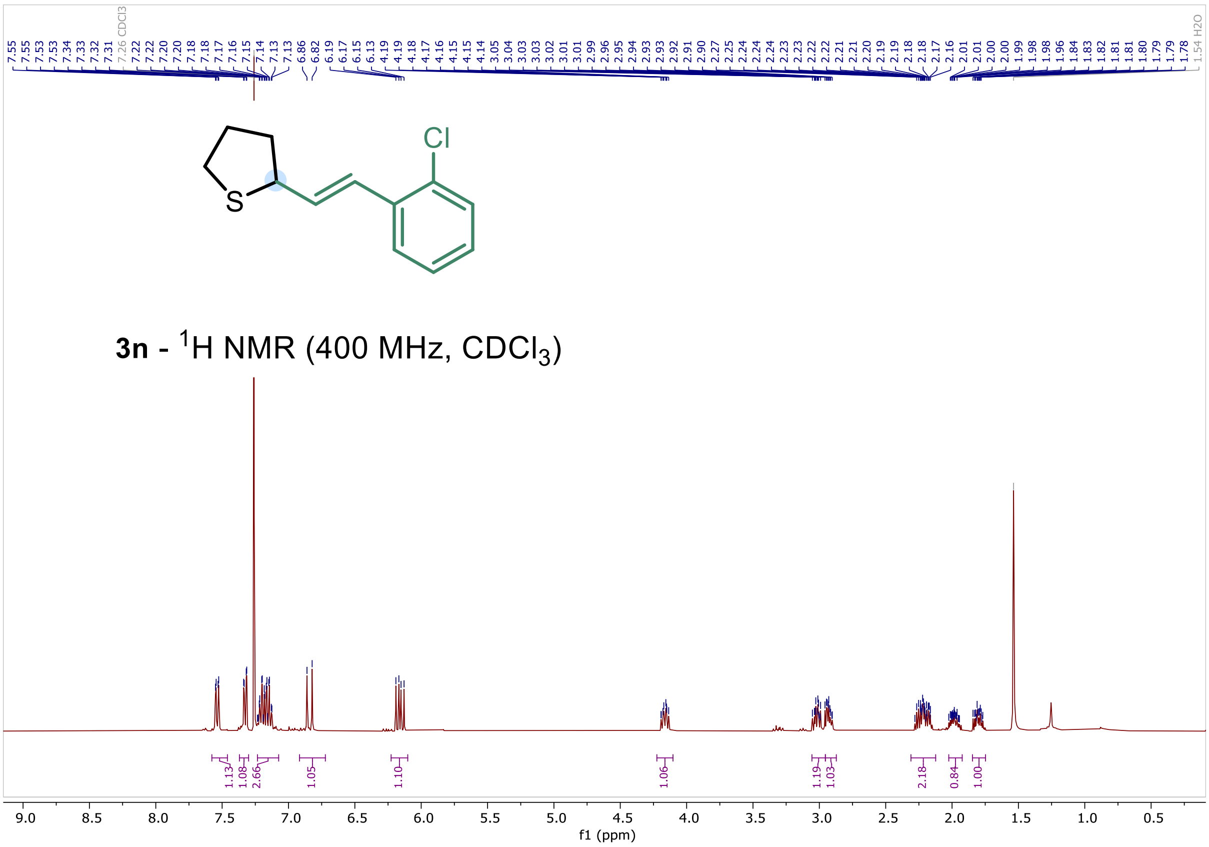
3n - ^{1}H NMR (400 MHz, $CDCl_3$)
f1 (ppm)

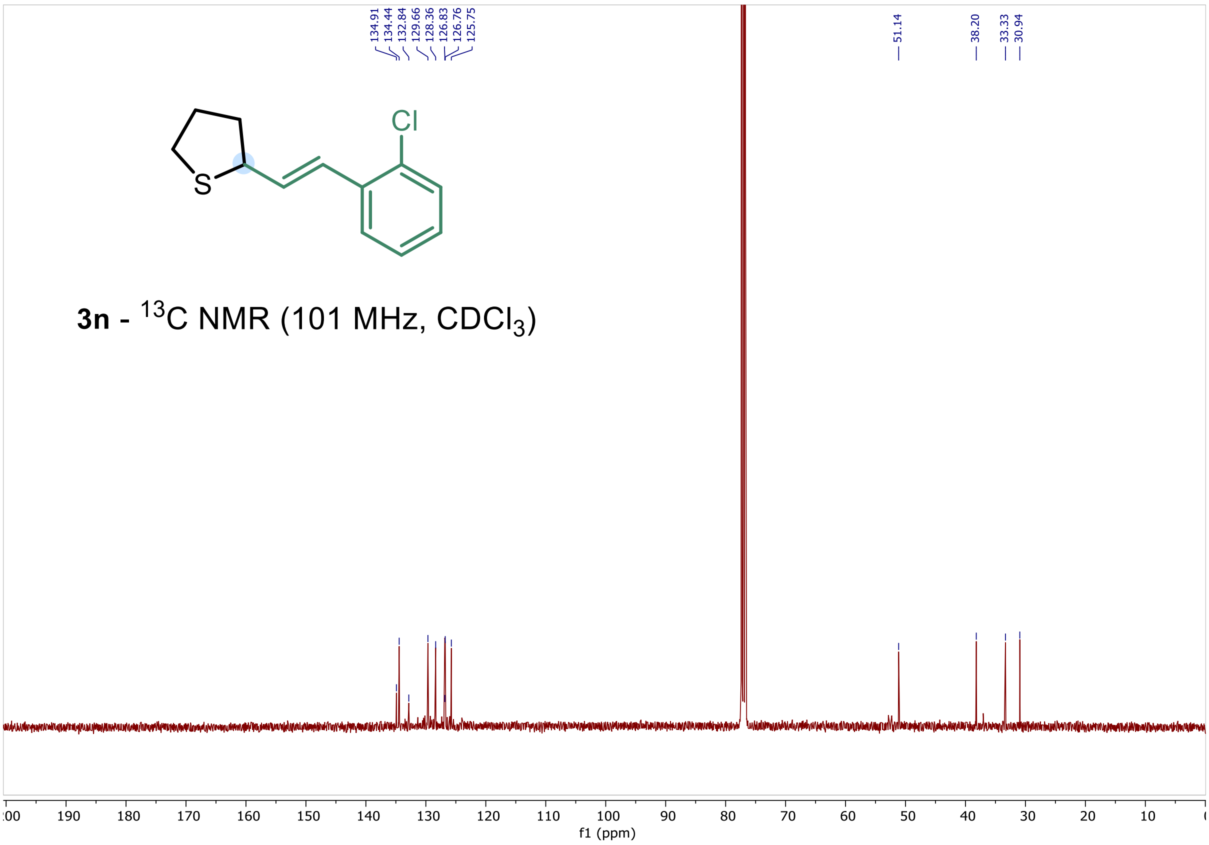
3n - ^{13}C NMR (101 MHz, $CDCl_3$)
134.91
134.44
132.84
129.66
128.36
126.83
126.76
125.75
51.14
38.20
33.33
30.94
f1 (ppm)

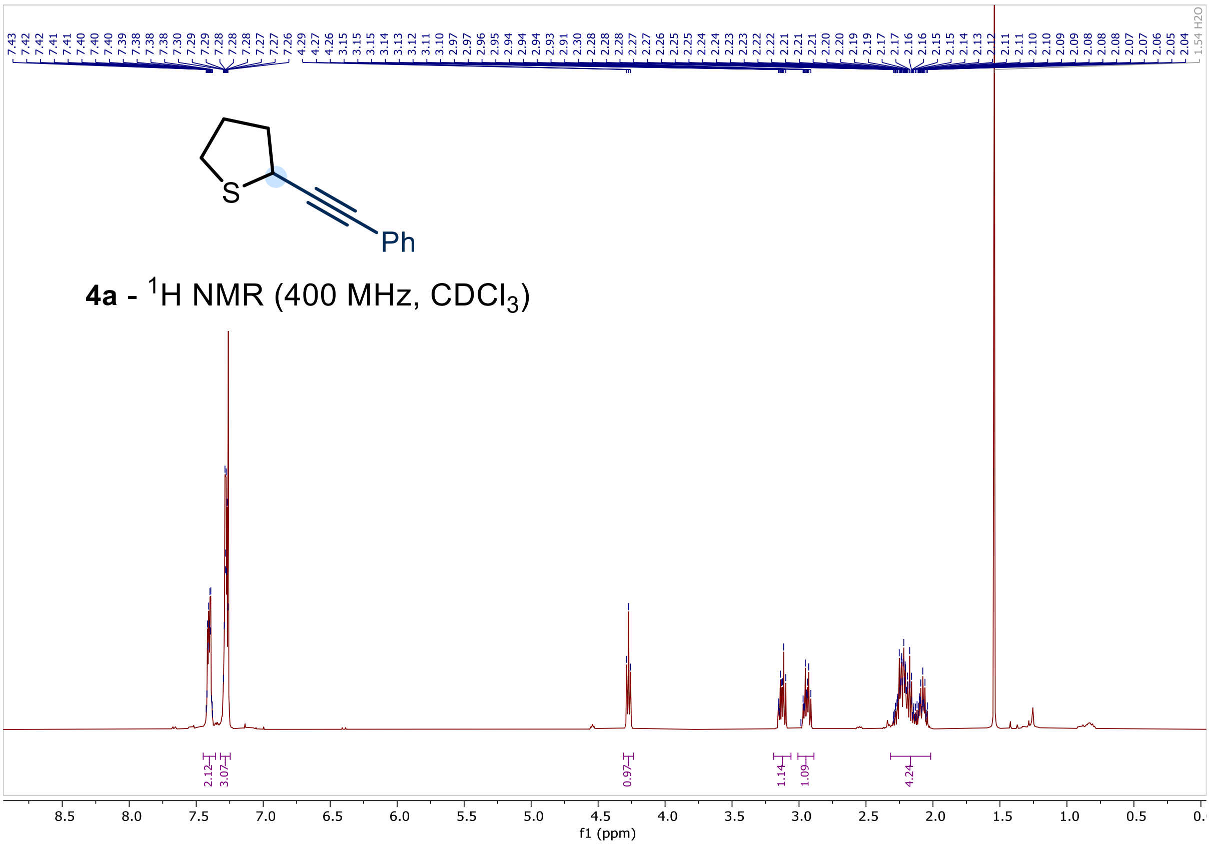
S
Ph
4a - ^{1}H NMR (400 MHz, $CDCl_3$)
f1 (ppm)

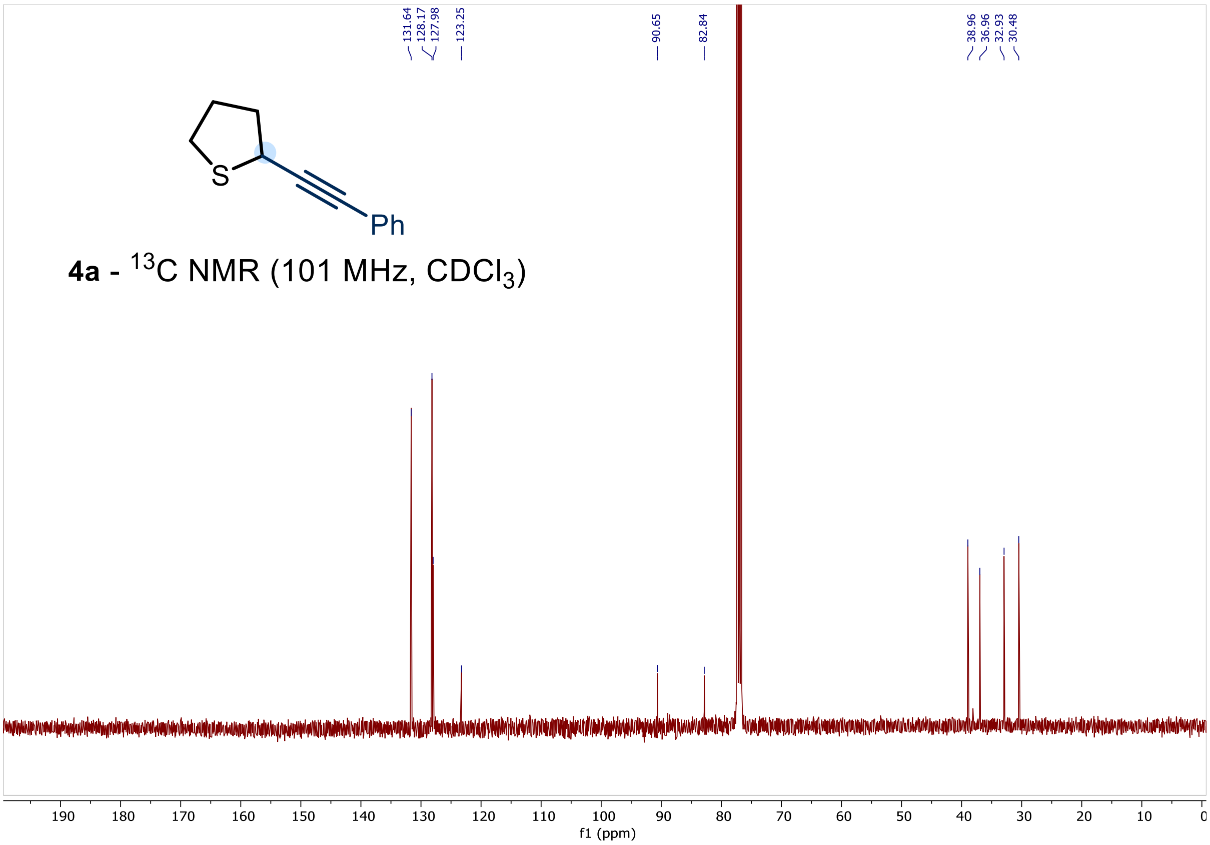
S
Ph
4a - ^{13}C NMR (101 MHz, $CDCl_3$)
131.64
128.17
127.98
123.25
90.65
82.84
38.96
36.96
32.93
30.48
f1 (ppm)

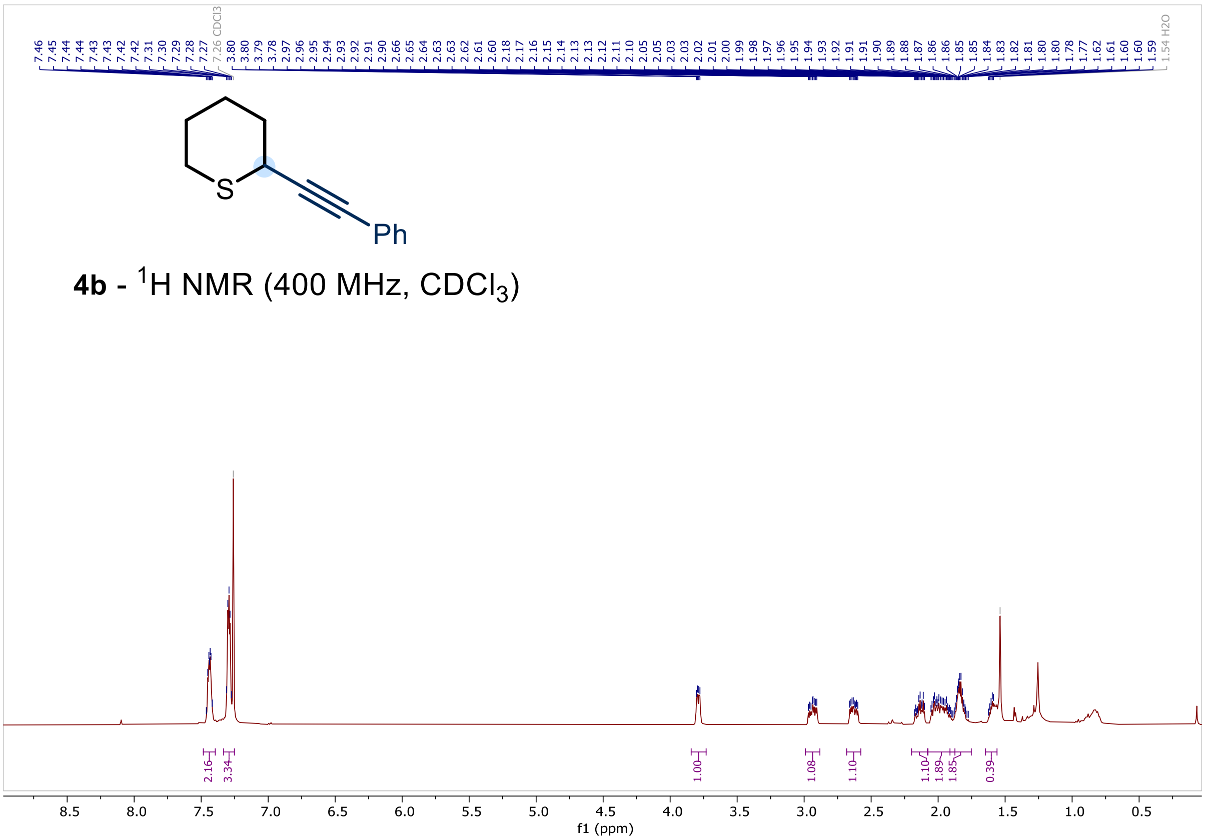
4b - ^{1}H NMR (400 MHz, $CDCl_3$)
f1 (ppm)

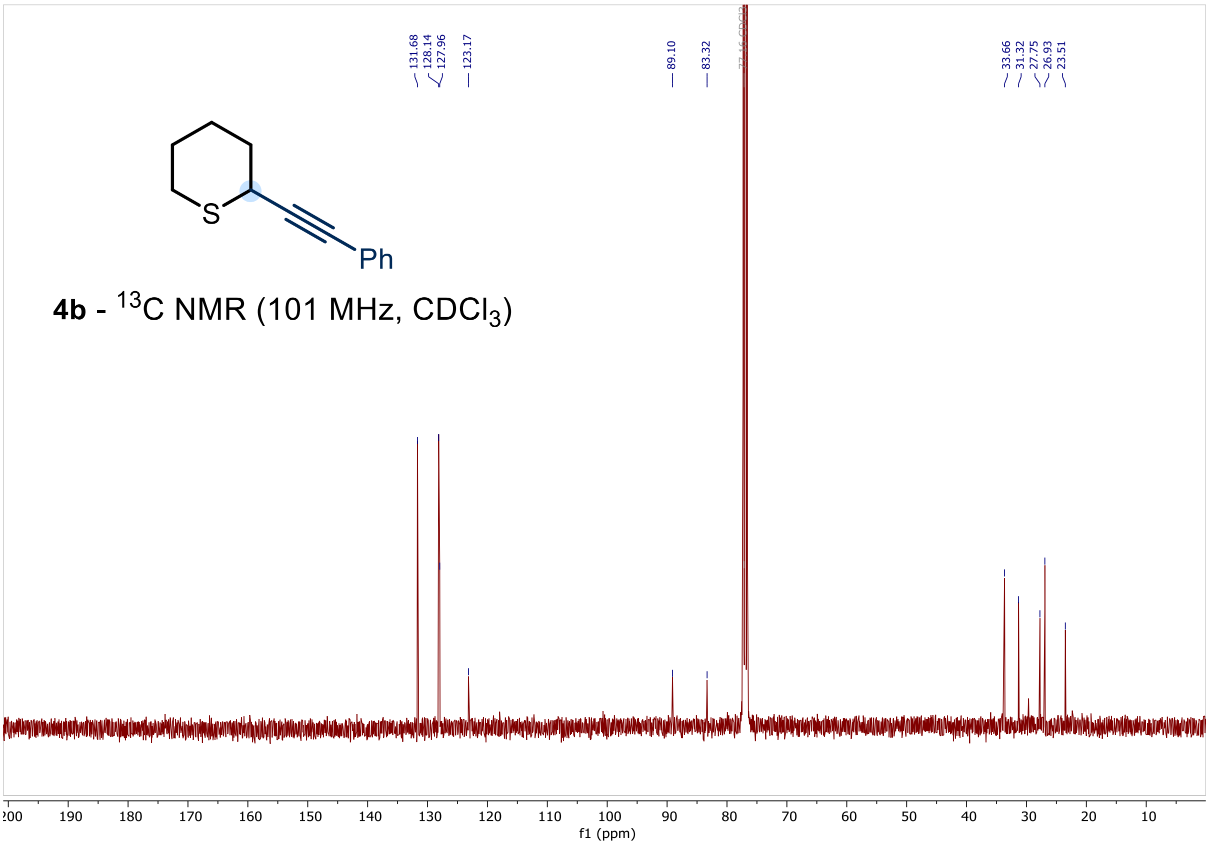
4b - ^{13}C NMR (101 MHz, $CDCl_3$)
131.68
128.14
127.96
123.17
89.10
83.32
33.66
31.32
27.75
26.93
23.51
f1 (ppm)

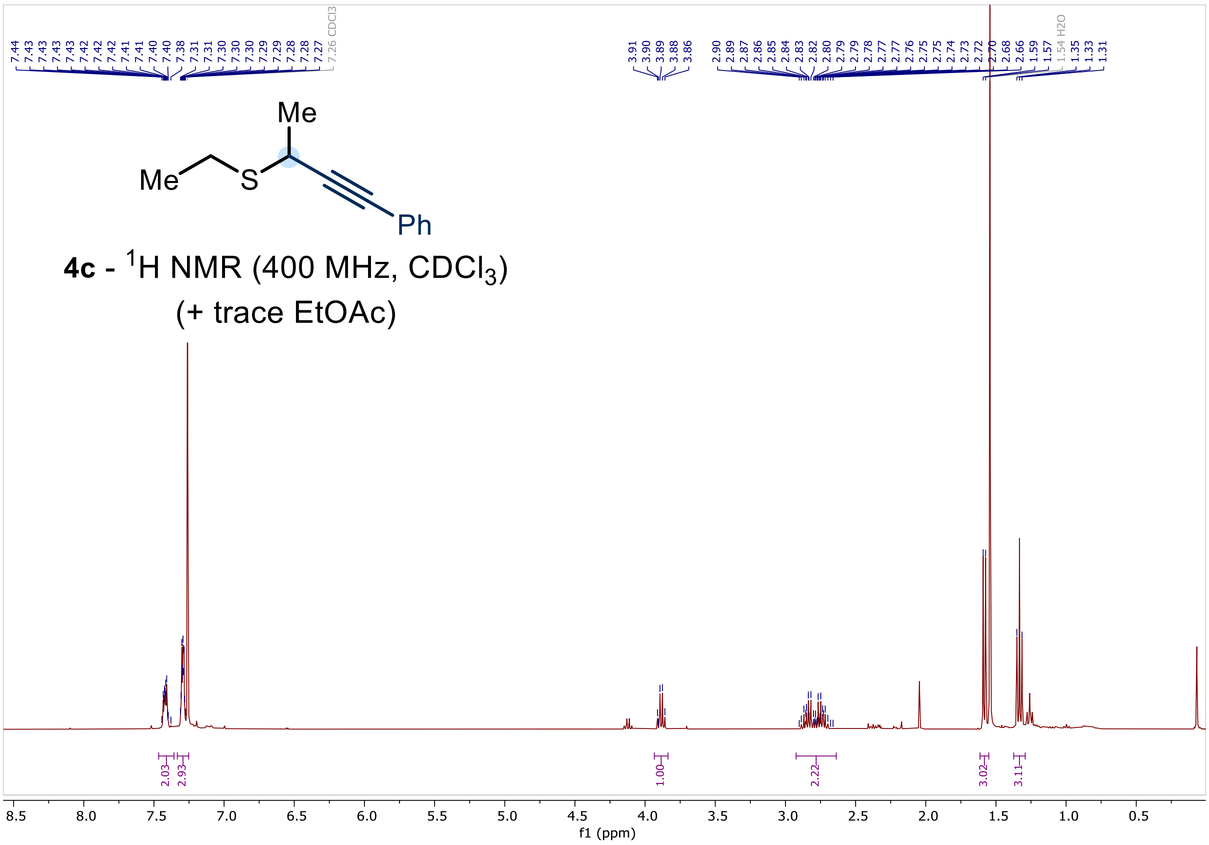
Me
Me
S
Ph
4c - ¹H NMR (400 MHz, CDCl₃)
(+ trace EtOAc)
f1 (ppm)

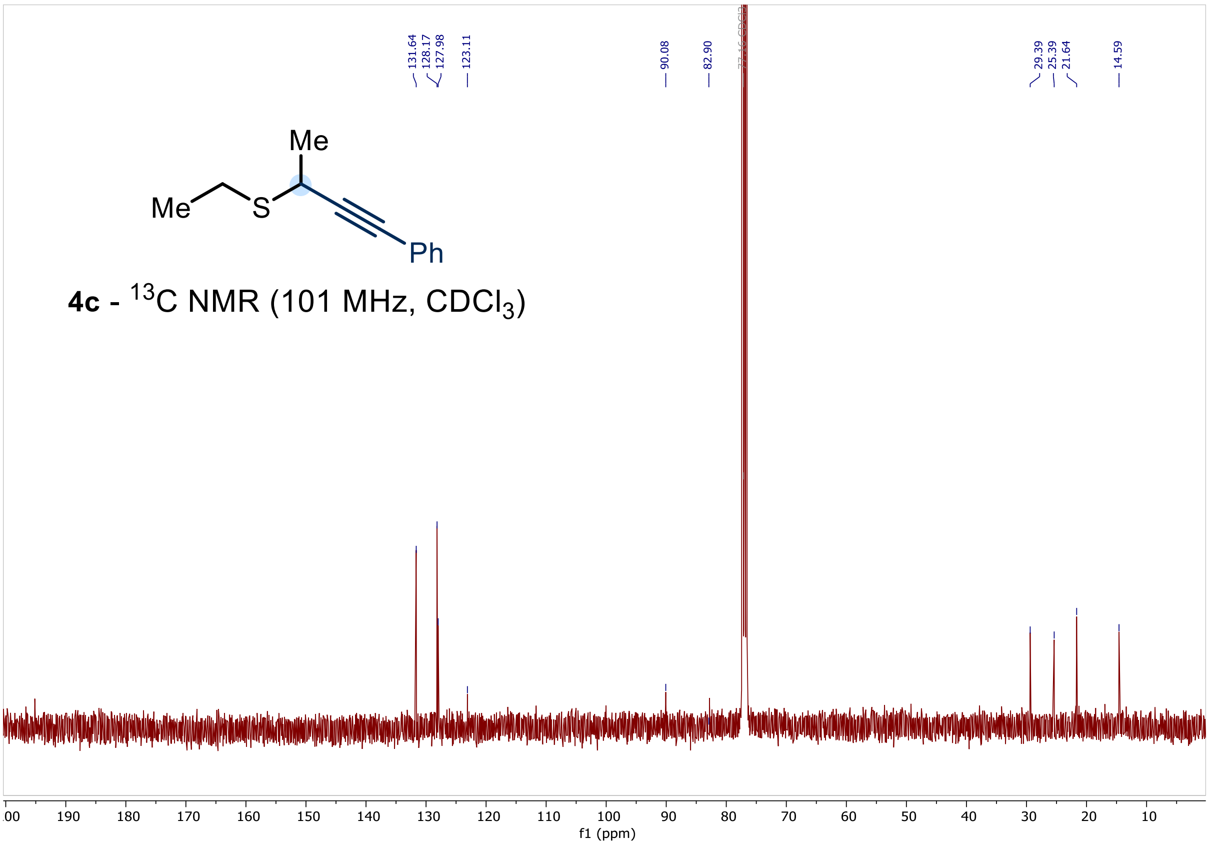
Me
Me
S
Ph
4c - ¹³C NMR (101 MHz, CDCl₃)
131.64
128.17
127.98
123.11
90.08
82.90
29.39
25.39
21.64
14.59
f1 (ppm)

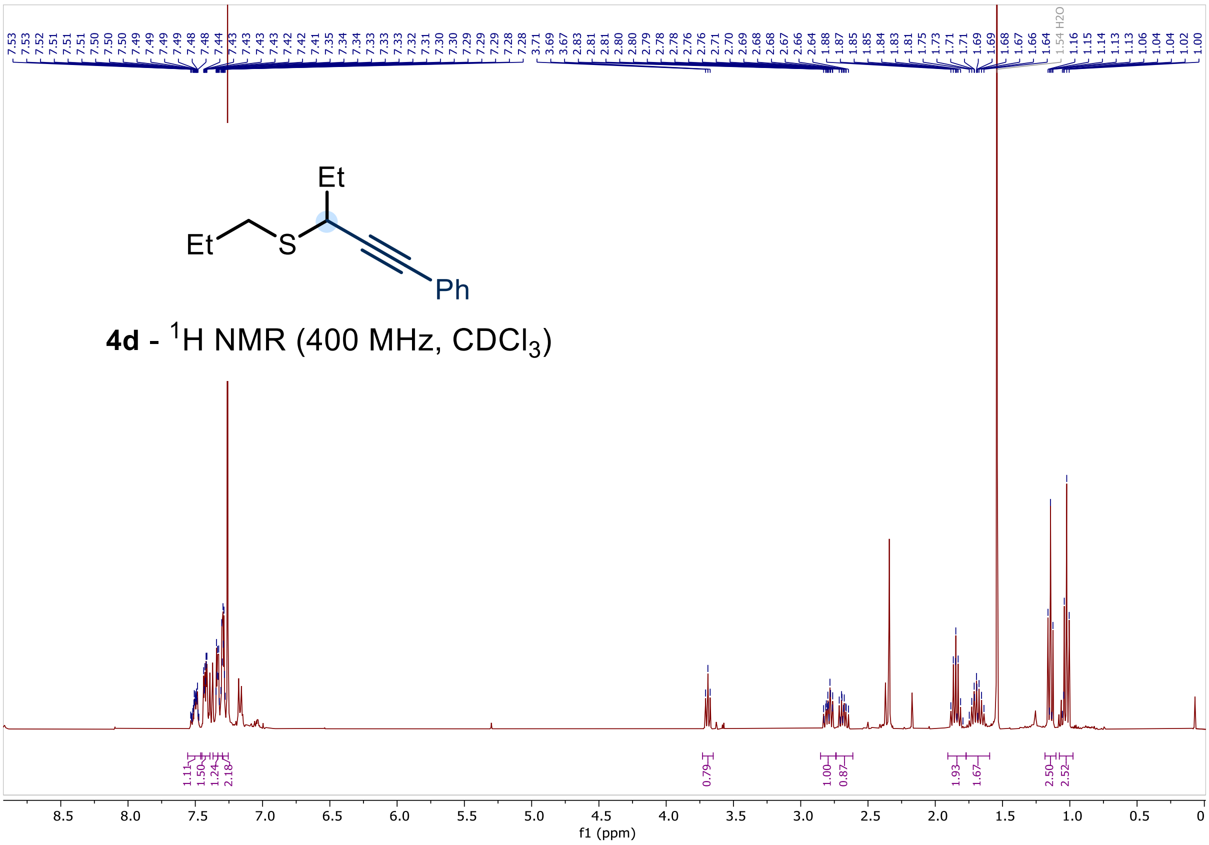
Et
Et
S
Ph
4d - ¹H NMR (400 MHz, CDCl₃)
f1 (ppm)

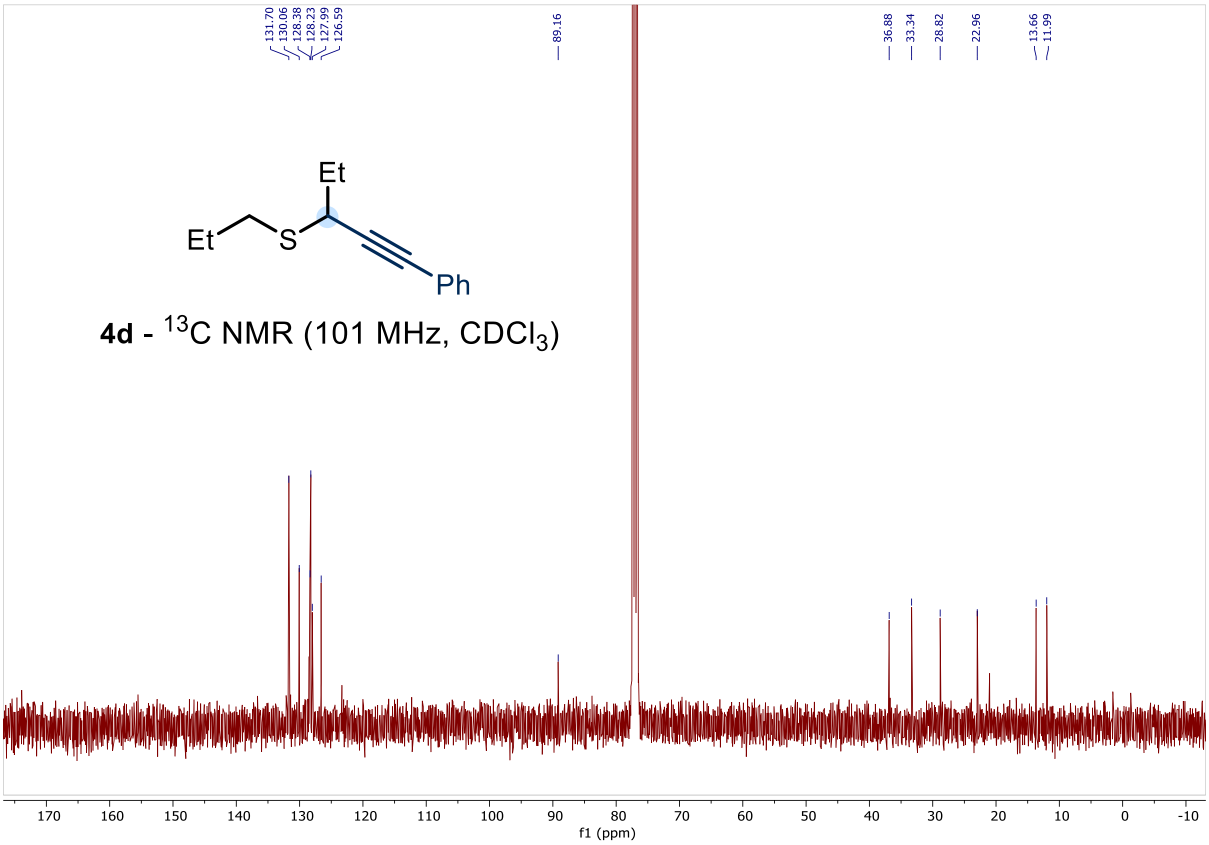
Et
Et
S
Ph
4d - ¹³C NMR (101 MHz, CDCl₃)
131.70
130.06
128.38
128.23
127.99
126.59
89.16
36.88
33.34
28.82
22.96
13.66
11.99
f1 (ppm)

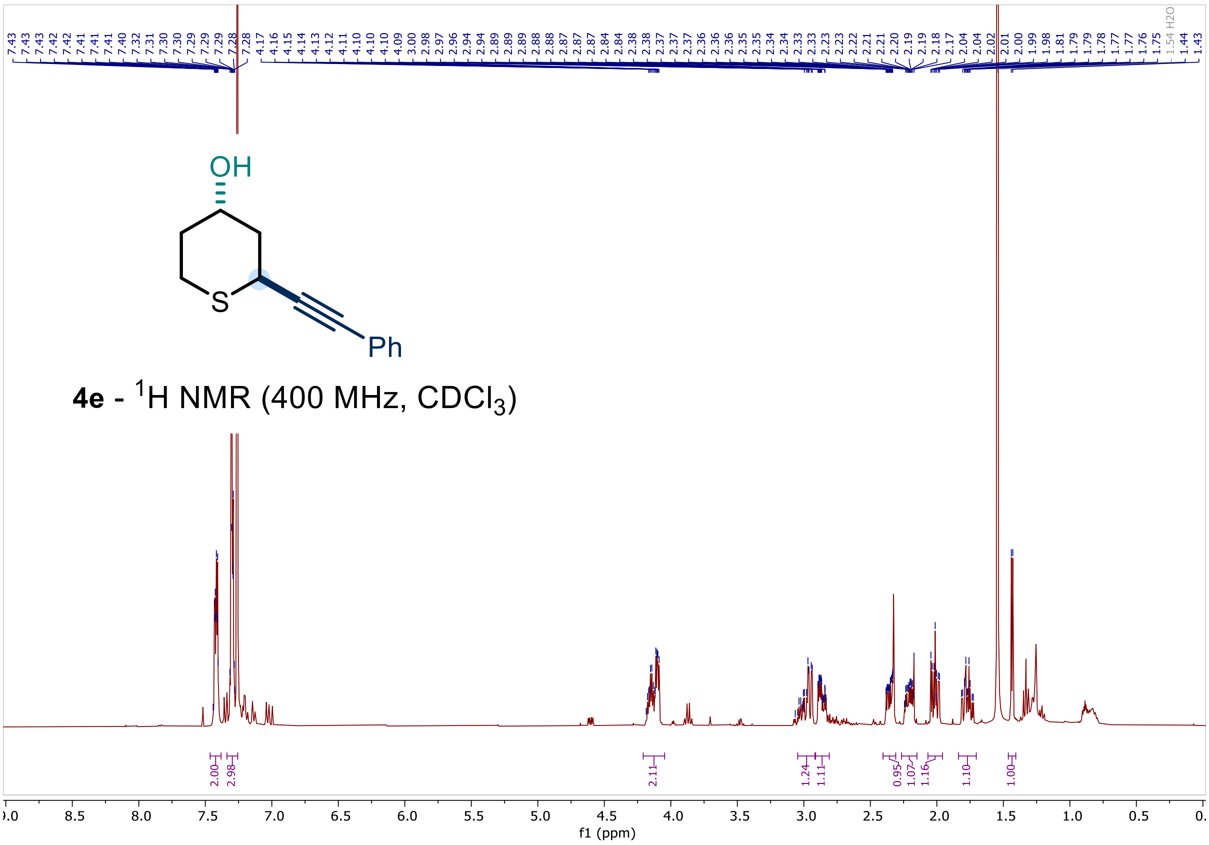
OH
S
Ph
4e - 1H NMR (400 MHz, CDCl3)
f1 (ppm)

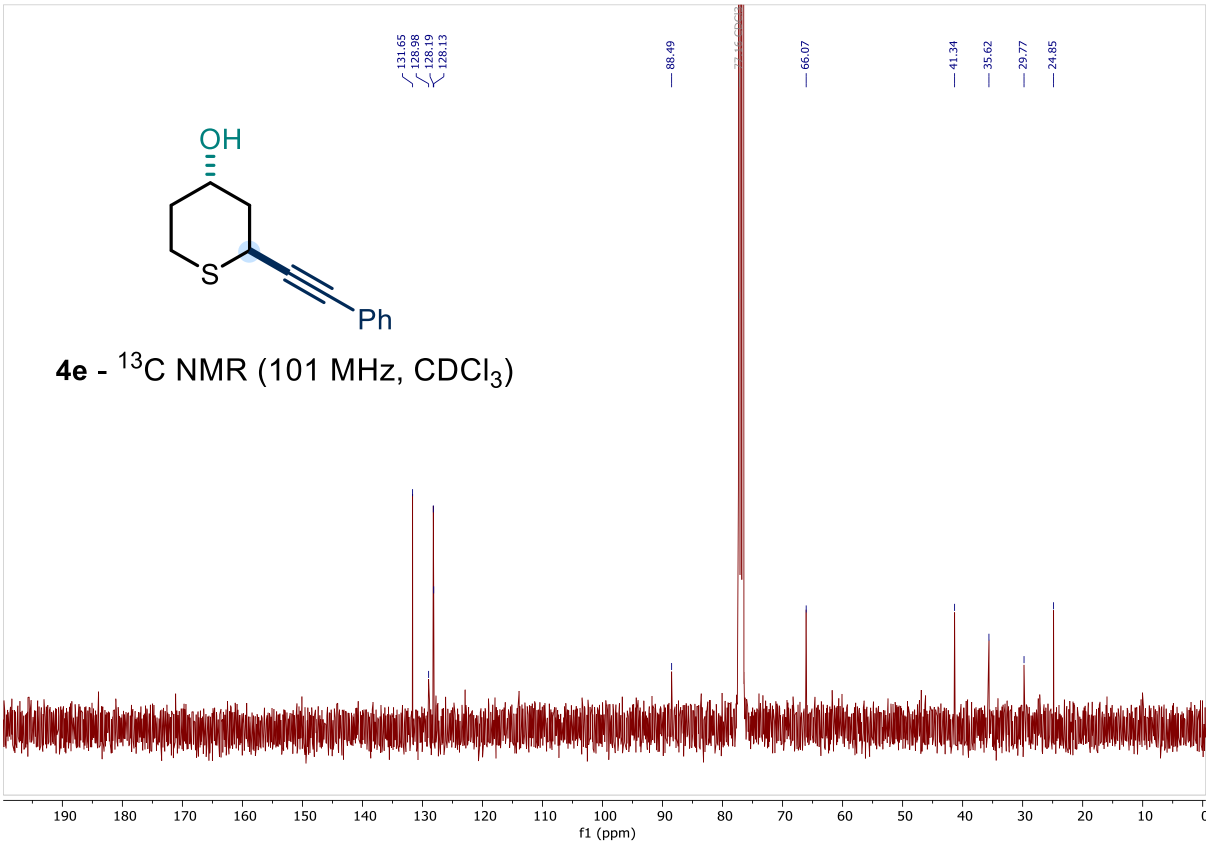
131.65
128.98
128.19
128.13
88.49
66.07
41.34
35.62
29.77
24.85
OH
S
Ph
4e - 13C NMR (101 MHz, CDCl3)
f1 (ppm)

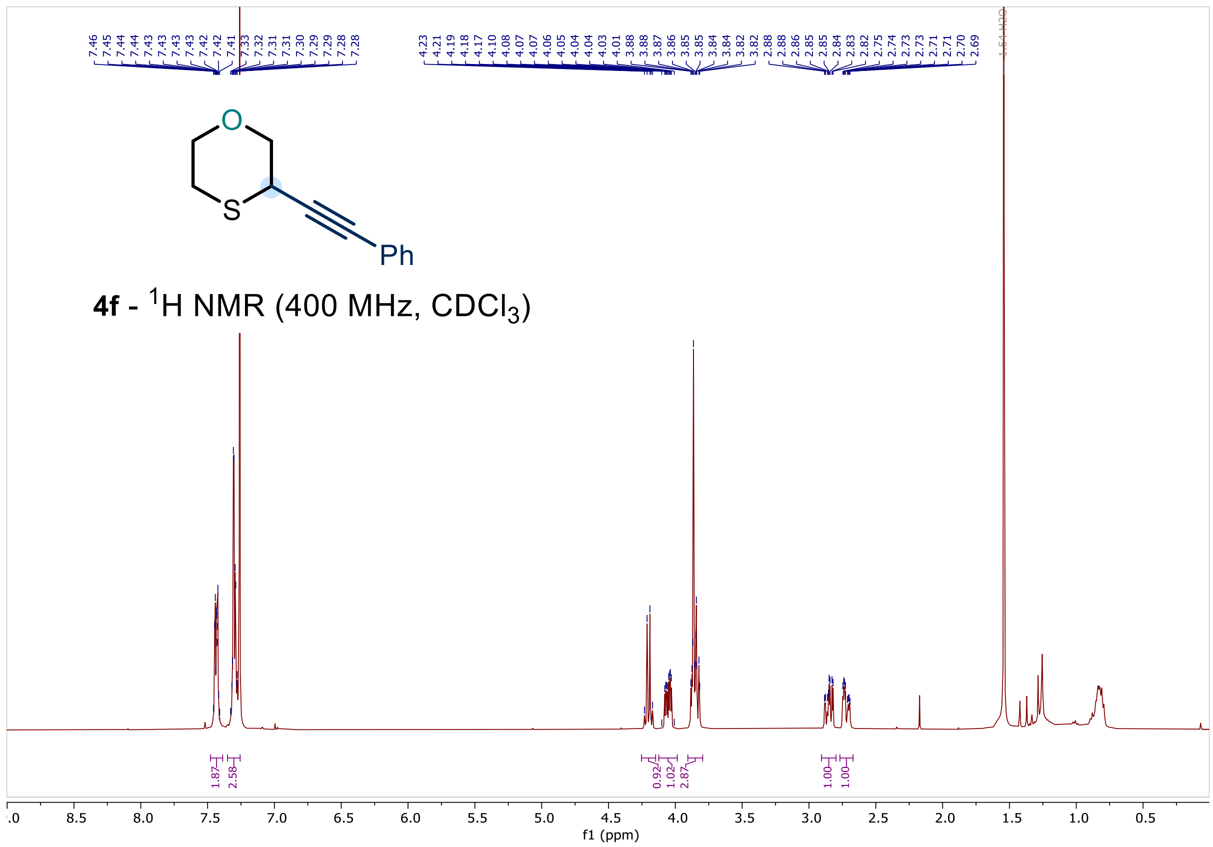

O
S
Ph
4f - 1H NMR (400 MHz, CDCl3)
f1 (ppm)

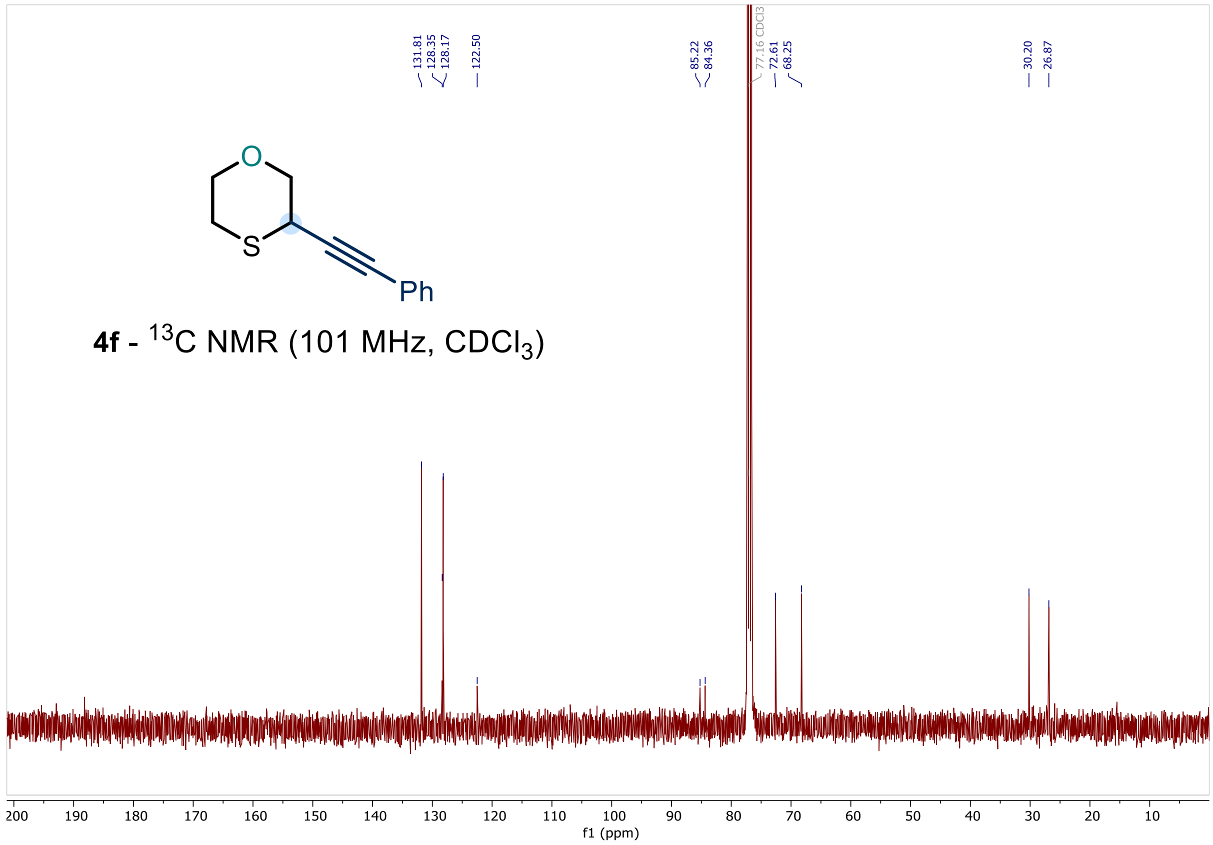

131.81
128.35
128.17
122.50
85.22
84.36
77.16 CDCl3
72.61
68.25
30.20
26.87
O
S
Ph
4f - 13C NMR (101 MHz, CDCl3)
f1 (ppm)

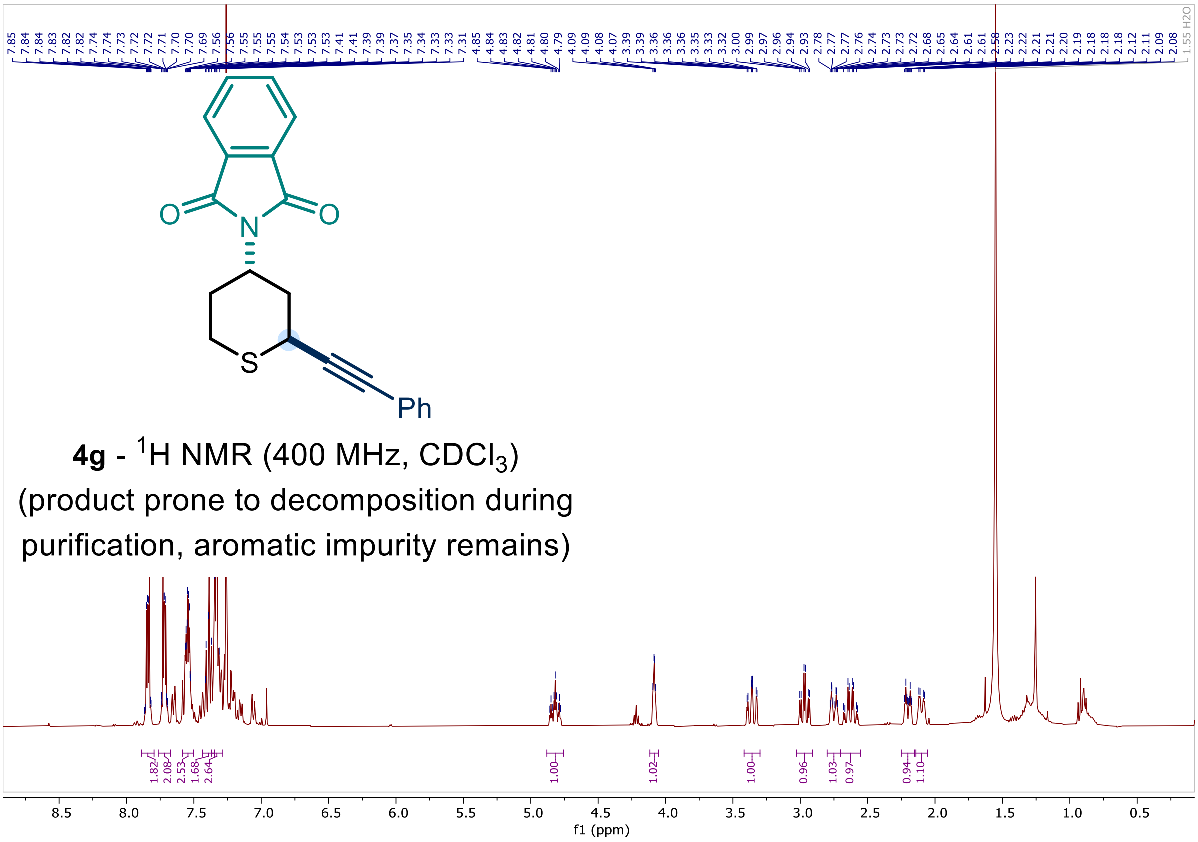
O
N
O
S
Ph
4g - 1H NMR (400 MHz, CDCl3)
(product prone to decomposition during
purification, aromatic impurity remains)
f1 (ppm)

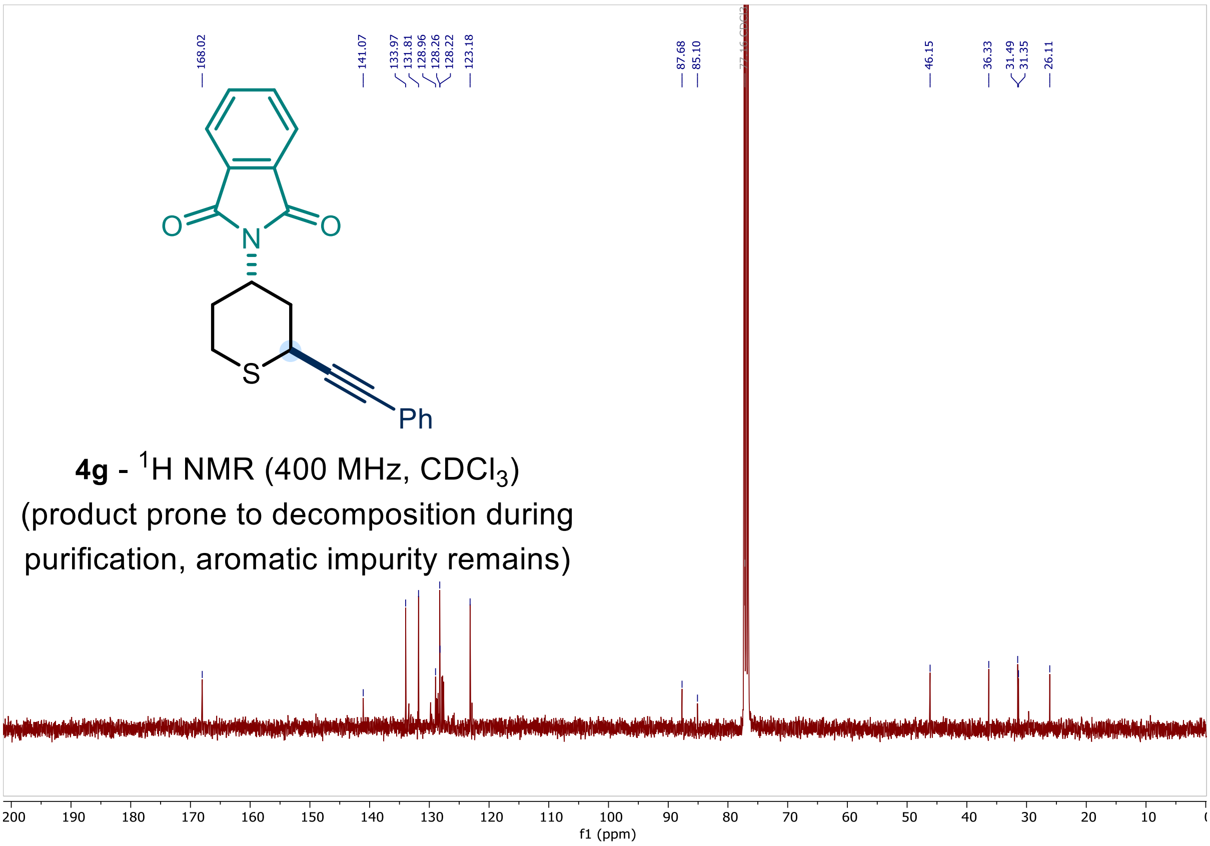
O
N
O
S
Ph
4g - 1H NMR (400 MHz, CDCl3)
(product prone to decomposition during
purification, aromatic impurity remains)
f1 (ppm)

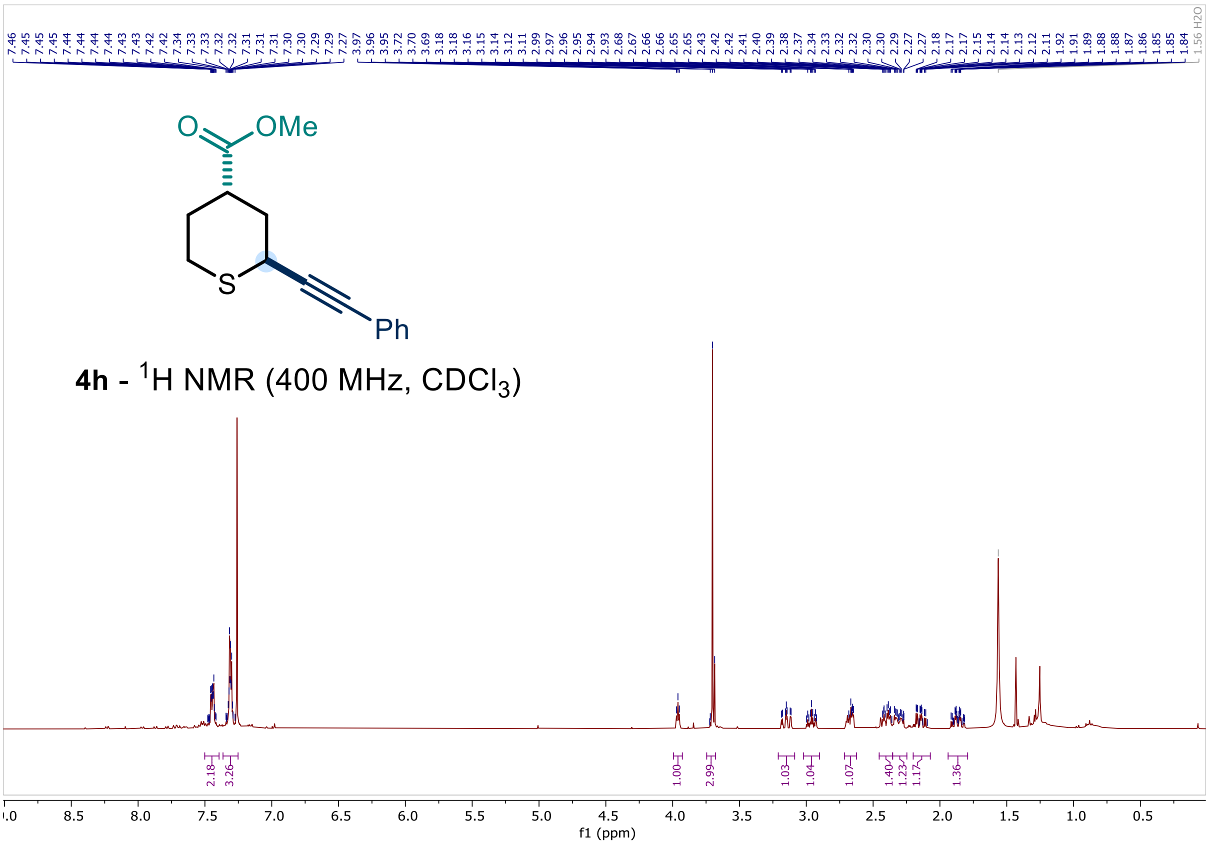
OMe
O
S
Ph
4h - ¹H NMR (400 MHz, CDCl₃)
f1 (ppm)

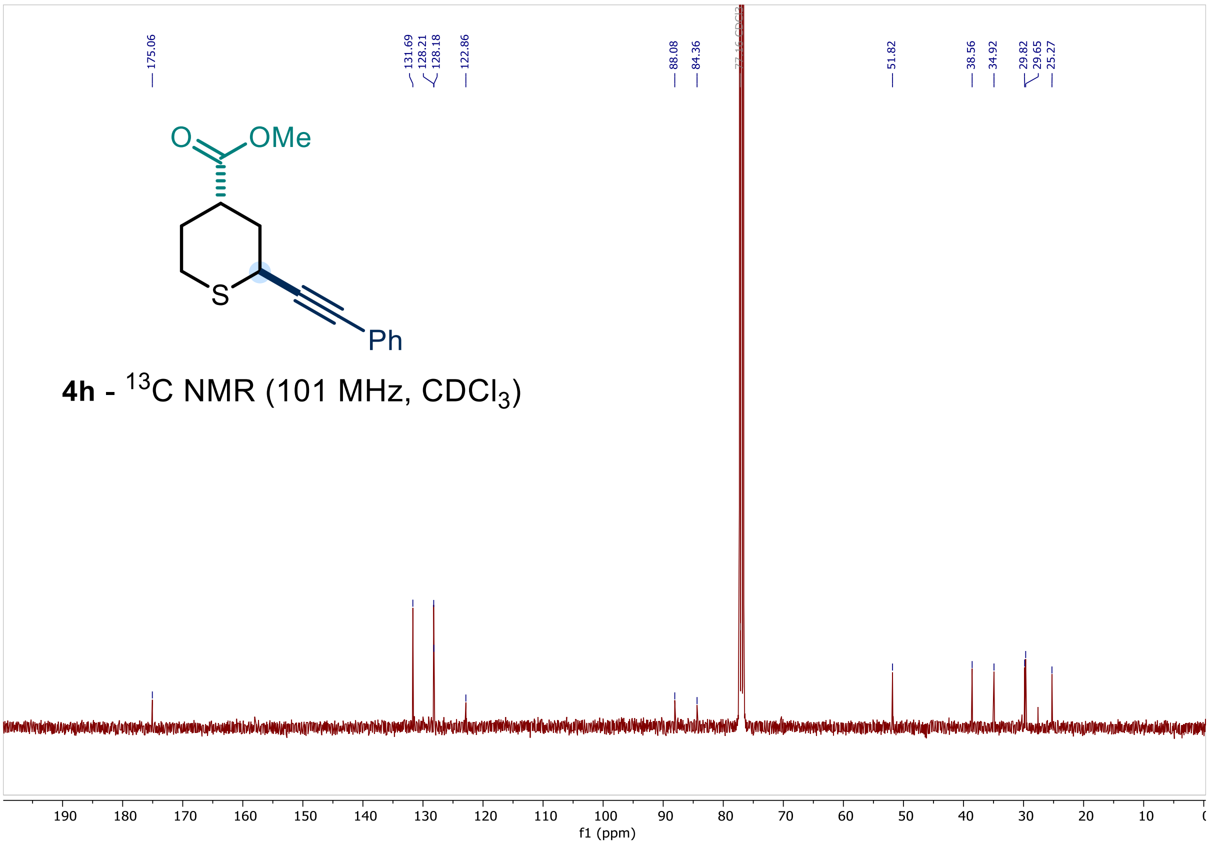
175.06
131.69
128.21
128.18
122.86
88.08
84.36
51.82
38.56
34.92
29.82
29.65
25.27
OMe
O
S
Ph
4h - ¹³C NMR (101 MHz, CDCl₃)
f1 (ppm)

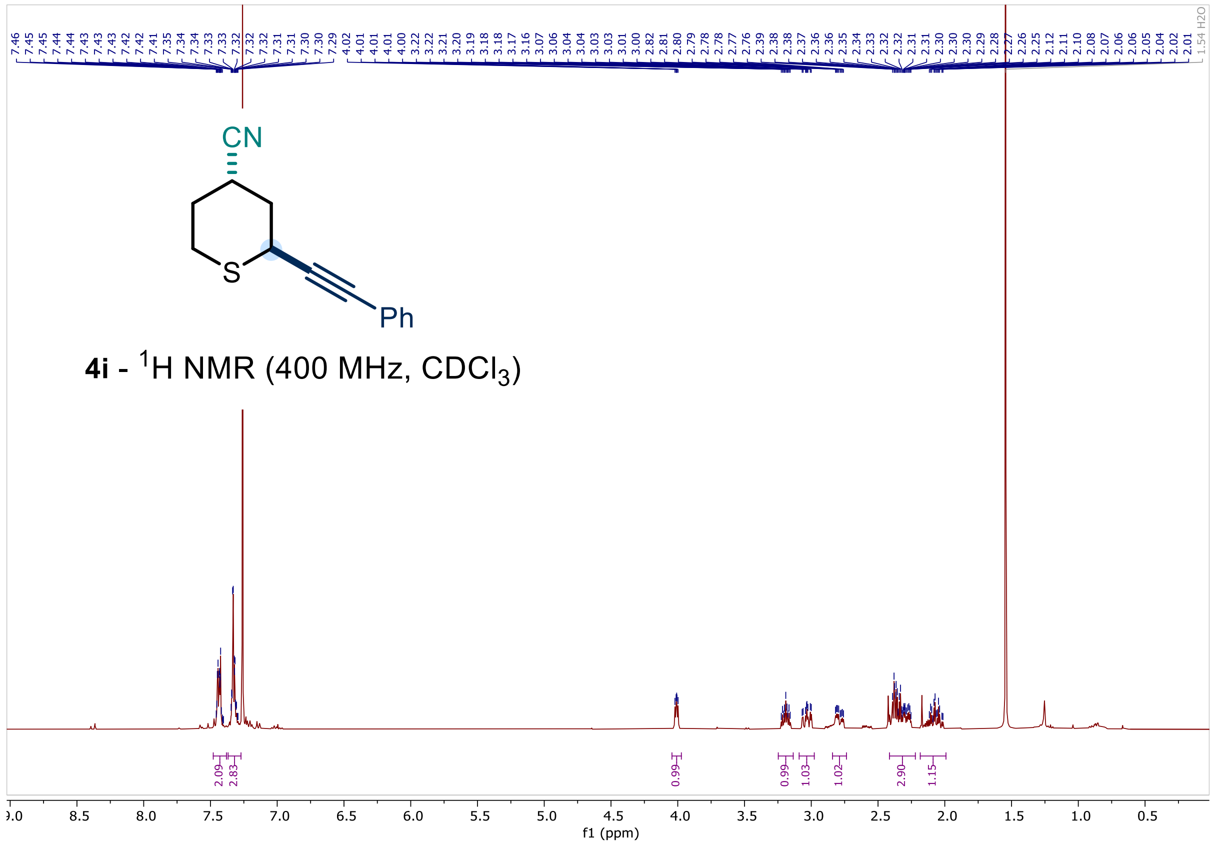
CN
S
Ph
4i - ¹H NMR (400 MHz, CDCl₃)
2.09
2.83
0.99
0.99
1.03
1.02
2.90
1.15
1.54 H2O
f1 (ppm)

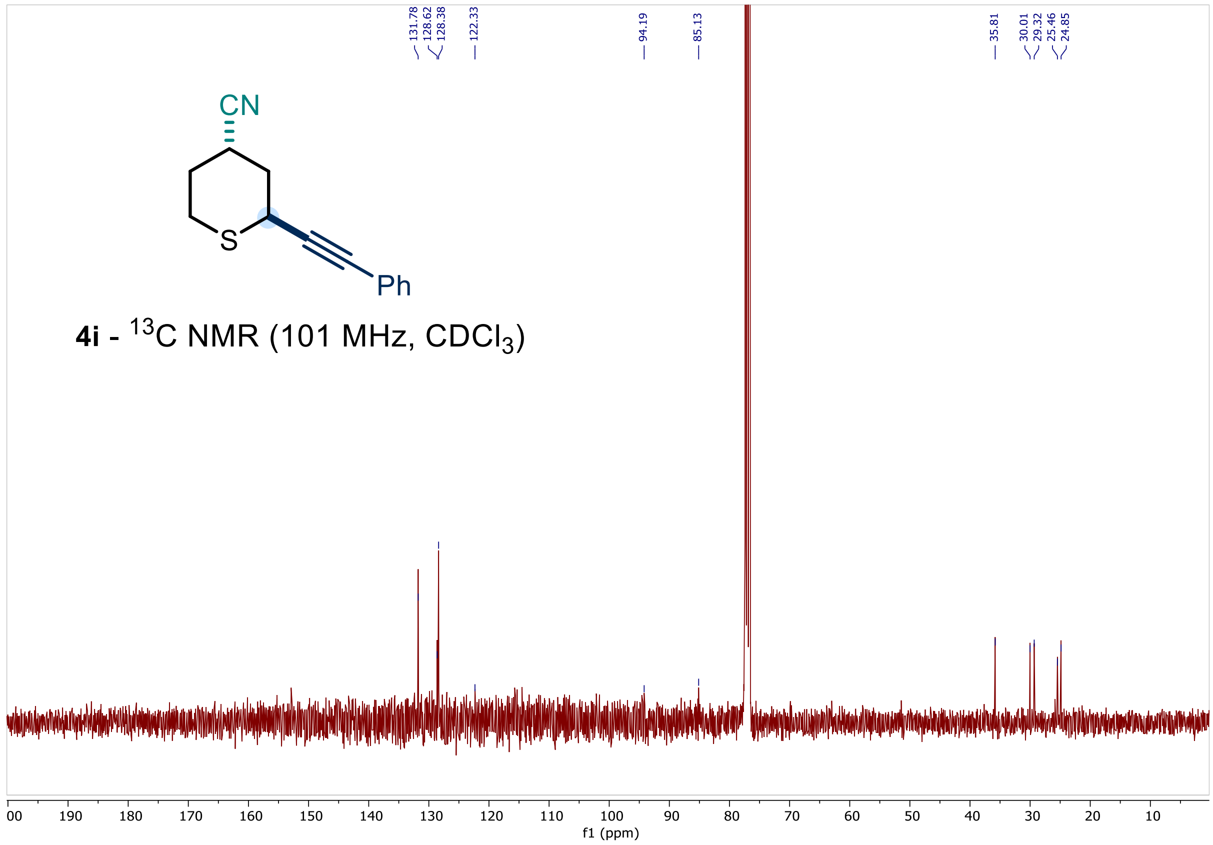
131.78
128.62
128.38
122.33
94.19
85.13
35.81
30.01
29.32
25.46
24.85
CN
S
Ph
4i - ¹³C NMR (101 MHz, CDCl₃)
f1 (ppm)

References

(1) Jhulki, I.; Chanani, P. K.; Abdelwahed, S. H.; Begley, T. P. A Remarkable Oxidative Cascade That Replaces the Riboflavin C8 Methyl with an Amino Group during Roseoflavin Biosynthesis. *J. Am. Chem. Soc.* **2016**, *138* (27), 8324–8327. https://doi.org/10.1021/jacs.6b02469.

(2) Paul, S.; Guin, J. Radical C(Sp3)–H Alkenylation, Alkynylation and Allylation of Ethers and Amides Enabled by Photocatalysis. *Green Chem.* **2017**, *19* (11), 2530–2534. https://doi.org/10.1039/C7GC00840F.

(3) Sawangphon, T.; Katrun, P.; Chaisiwamongkhol, K.; Pohmakotr, M.; Reutrakul, V.; Jaipetch, T.; Soorukram, D.; Kuhakarn, C. An Improved Synthesis of Vinyl- and β-Iodovinyl Sulfones by a Molecular Iodine-Mediated One-Pot Iodosulfonation-Dehydroiodination Reaction. *Synth. Commun.* **2013**, *43* (12), 1692–1707. https://doi.org/10.1080/00397911.2012.663448.

(4) Cai, S.; Xu, Y.; Chen, D.; Li, L.; Chen, Q.; Huang, M.; Weng, W. Visible-Light-Enabled Decarboxylative Sulfonylation of Cinnamic Acids with Sulfonylhydrazides under Transition-Metal-Free Conditions. *Org. Lett.* **2016**, *18* (12), 2990–2993. https://doi.org/10.1021/acs.orglett.6b01353.

(5) Hayes, T. O. P.; Slater, B.; Horan, R. A. J.; Radigois, M.; Wilden, J. D. A Novel Sulfonamide Non-Classical Carbenoid: A Mechanistic Study for the Synthesis of Enediynes. *Org. Biomol. Chem.* **2017**, *15* (46), 9895–9902. https://doi.org/10.1039/C7OB02437A.

(6) He, D.; Zhong, W.; Zhou, M.; Wang, B.; Li, M.; Jiang, H.; Wu, W. Palladium-Catalyzed Regio- and Stereoselective Coupling of Alkynylsulfones with Alkenes: Access to Dichlorinated Vinyl Sulfones. *Org. Lett.* **2022**, *24* (31), 5802–5806. https://doi.org/10.1021/acs.orglett.2c02324.

(7) Luque, A.; Groß, J.; Zähringer, T. J. B.; Kerzig, C.; Opatz, T. Vinylcyclopropane [3+2] Cycloaddition with Acetylenic Sulfones Based on Visible Light Photocatalysis. *Chem. Eur J.* **2022**, *28* (18), e202104329. https://doi.org/https://doi.org/10.1002/chem.202104329.

(8) Barber, C.; Blakemore, D. Triazolylpiperidine Derivatives and Use Thereof in Therapy. WO 2006/136917, 2006.

(9) Das, B. G.; Ghorai, P. Stereoselective Direct Reductive Amination of Ketones with Electron-Deficient Amines Using Re2O7/NaPF6 Catalyst. *Org. Biomol. Chem.* **2013**, *11* (26), 4379–4382. https://doi.org/10.1039/C3OB40918J.

(10) Broussard, T. C.; Pakhomova, S.; Neau, D. B.; Bonnot, R.; Waldrop, G. L. Structural Analysis of Substrate, Reaction Intermediate, and Product Binding in Haemophilus Influenzae Biotin Carboxylase. *Biochem.* **2015**, *54* (24), 3860–3870. https://doi.org/10.1021/acs.biochem.5b00340.

(11) Knowles , O. J.; Johannissen, L. O.; Crisenza, G. E. M.; Hay, S.; Leys, D.; Procter, D. J. A Vitamin B2-Photocatalysed Approach to Methionine Analogues. *Angew. Chem. Int. Ed.* **2022**, *61* (50), e202212158. https://doi.org/https://doi.org/10.1002/anie.202212158.

(12) Enßle, M.; Buck, S.; Werz, R.; Maas, G. Intramolecular Carbenoid Ylide Forming Reactions of 2-Diazo-3-Keto-4-Phthalimidocarboxylic Esters Derived from Methionine

and Cysteine. *Beilstein J. Org. Chem.* **2012**, *8*, 433–440. https://doi.org/10.3762/bjoc.8.49.

(13) Scharinger, F.; Pálvölgyi, Á. M.; Weisz, M.; Weil, M.; Stanetty, C.; Schnürch, M.; Bica-Schröder, K. Sterically Demanding Flexible Phosphoric Acids for Constructing Efficient and Multi-Purpose Asymmetric Organocatalysts. *Angew. Chem. Int. Ed.* **2022**, *61* (26), e202202189. https://doi.org/https://doi.org/10.1002/anie.202202189.

(14) Crich, D.; Banerjee, A. Native Chemical Ligation at Phenylalanine. *J. Am. Chem. Soc.* **2007**, *129* (33), 10064–10065. https://doi.org/10.1021/ja072804l.

(15) Raza, A. R.; Saddiqa, A.; Çakmak, O. Chiral Pool-Based Synthesis of Naphtho-Fused Isocoumarins. *Chirality* **2015**, *27* (12), 951–957. https://doi.org/https://doi.org/10.1002/chir.22530.

(16) Okazaki, Y.; Asai, T.; Ando, F.; Koketsu, J. The Stevens Rearrangement of Sulfur Ylide Generated by Electrochemical Reduction of Sulfonium Salt. *Chem. Lett.* **2005**, *35* (1), 98–99. https://doi.org/10.1246/cl.2006.98.

(17) Wakaki, T.; Sakai, K.; Enomoto, T.; Kondo, M.; Masaoka, S.; Oisaki, K.; Kanai, M. C(Sp3)−H Cyanation Promoted by Visible-Light Photoredox/Phosphate Hybrid Catalysis. *Chem. Eur. J.* **2018**, *24* (32), 8051–8055. https://doi.org/https://doi.org/10.1002/chem.201801746.

(18) Papadopoulos, G. N.; Kokotou, M. G.; Spiliopoulou, N.; Nikitas, N. F.; Voutyritsa, E.; Tzaras, D. I.; Kaplaneris, N.; Kokotos, C. G. Phenylglyoxylic Acid: An Efficient Initiator for the Photochemical Hydrogen Atom Transfer C−H Functionalization of Heterocycles. *ChemSusChem* **2020**, *13* (22), 5934–5944. https://doi.org/https://doi.org/10.1002/cssc.202001892.

(19) Wang, Y.-T.; Shih, Y.-L.; Wu, Y.-K.; Ryu, I. Site-Selective C(Sp3)-H Alkenylation Using Decatungstate Anion as Photocatalyst. *Adv. Synth. Catal.* **2022**, *364* (5), 1039–1043. https://doi.org/https://doi.org/10.1002/adsc.202101374.

(20) Ahmed, N.; Spears, R. J.; Sheppard, T. D.; Chudasama, V. Functionalisation of Ethereal-Based Saturated Heterocycles with Concomitant Aerobic C–H Activation and C–C Bond Formation. *Chem. Sci.* **2022**, *13* (29), 8626–8633. https://doi.org/10.1039/D2SC01626E.
